# A systematic review of studies that estimated the burden of chronic non-communicable rare diseases using disability-adjusted life years

**DOI:** 10.1186/s13023-024-03342-3

**Published:** 2024-09-09

**Authors:** Claudia Cruz Oliveira, Periklis Charalampous, Julien Delaye, Diana Alecsandra Grad, Pavel Kolkhir, Enkeleint A. Mechili, Brigid Unim, Brecht Devleesschauwer, Juanita A. Haagsma

**Affiliations:** 1 Department of Public Health, Erasmus MC University Medical Center, Rotterdam, The Netherlands; 2 European Organisation for Rare Diseases (EURORDIS), Paris, France; 3 Department of Public Health, Babes-Bolyai University, Cluj-Napoca, Romania; 4 Institute of Allergology, Charité - Universitätsmedizin Berlin, Corporate Member of Freie Universität Berlin, Humboldt-Universität zu Berlin, Berlin, Germany; 5 Fraunhofer Institute for Translational Medicine and Pharmacology ITMP, Allergology and Immunology, Berlin, Germany; 6 Clinic of Social and Family Medicine, School of Medicine, University of Crete, Crete, Greece; 7 Department of Healthcare, Faculty of Public Health, University of Vlora, Vlora, Albania; 8 Department of Cardiovascular, Endocrine-Metabolic Diseases and Aging, Istituto Superiore Di Sanità, Rome, Italy; 9 Department of Epidemiology and Public Health, Sciensano, Brussels, Belgium; 10 Department of Translational Physiology, Infectiology and Public Health, Ghent University, Merelbeke, Belgium; 11 Netherlands Institute for Health Sciences, Erasmus University Rotterdam, Rotterdam, The Netherlands

**Keywords:** Disability-adjusted life years, Rare diseases, Methods, Population health, Review

## Abstract

**Background:**

Initiatives aiming to assess the impact of rare diseases on population health might be hampered due to the complexity of disability-adjusted life years (DALYs) estimation. This study aimed to give insight into the epidemiological data sources and methodological approaches used in studies that estimated DALYs for chronic non-communicable rare diseases (CNCRD), and compare its results.

**Methods:**

A literature strategy was developed for peer-review search in Embase and Medline, and also performed on grey literature databases and population health and/or rare disease-focused websites. We included studies that determined the burden of CNCRD listed on the Orphanet’s and/or the Genetic and Rare Diseases information center (GARD) websites. We excluded communicable and occupational diseases, rare cancers, and cost-effectiveness/benefit studies. Two researchers independently screened the identified records and extracted data from the final included studies. We used the Guidelines for Accurate and Transparent Health Estimates Reporting (GATHER) statement to assess the quality of reporting of the included studies. The data synthesis depicted the studies’ characteristics, their distribution by geographic coverage and the group of disease(s) they focused on, the methods and data input sources used and estimated DALY per case.

**Results:**

In total, 533 titles were screened, and 18 studies were included. These studies covered 19 different CNCRDs, of which most fell in the disease category “Diseases of the nervous system”. Diverse methodological approaches and data input sources were observed among burden of CNCRD studies. A wide range of DALY per case was observed across the different studies and diseases included.

**Conclusions:**

A low number of burden of CNCRD studies was observed and most estimates resulted from multi-country studies, underlining the importance of international cooperation to further CNCRD research. This study revealed a lack of epidemiological data and harmonization of methods which hampers comparisons across burden of CNCRD studies.

**Supplementary Information:**

The online version contains supplementary material available at 10.1186/s13023-024-03342-3.

## Background

Rare diseases are a heterogeneous group of diseases that affect a small proportion of the population. The criteria that are used to define a rare disease vary worldwide. In the European Union (EU), a disease is defined as rare if less than 5 in 10,000 people are affected, in China when it affects less than 1 in 10,000 people, and in the United States (US) when it affects fewer than 200,000 people in the country (around 6 in 10,000) [1–3]. Currently, after combining different sources, more than 10,000 rare diseases have been identified globally, including communicable and non-communicable diseases (NCDs). However, most of these diseases are chronic non-communicable diseases and are often progressive and/or degenerative, life-threatening, and often associated with significantly reduced quality of life [4–7]. For this reason, estimates of prevalence, incidence and mortality might not fully capture the population health impact of chronic non-communicable rare diseases, as they do not reflect their associated morbidity and disability.

The concept of disability-adjusted life year (DALY) was introduced by Murray in 1994 [8]. The DALY is a population health metric that summarizes healthy time lost due to morbidity, disability, and premature mortality into a single metric [9, 10]. Because of this key feature, the DALY metric allows comparison of the health impact of causes of diseases across populations, and as such provides imperative information for priority setting, equitable resource allocation, and monitoring population health [11]. Moreover, DALYs might go beyond other health metrics by enabling the representation of the individual health impact of a disease through the average DALY per affected individual, namely, DALY per case. Specifically, expressing the DALY per individual case provides a more equitable opportunity for comparing the health impact between rare diseases and with other common diseases as it is not influenced by the prevalence of the disease.

The DALY consists of two components, namely Years of Life Lost due to premature mortality (YLL), reflecting the mortality component, and Years Lived with Disability (YLD), reflecting the morbidity component. To calculate YLL, data on mortality, cause of death and age at death per sex are required. On the other hand, the calculation of YLD requires information on the incidence or prevalence of the disease and its disease stages and patterns over time by age and sex [11–13].

Initiatives aiming to assess DALYs of a rare disease might be hampered due to the difficulty to find the required epidemiological data and the complexities of the approach to calculate DALYs. Since the 1990s, the DALY has been used in the Global Burden of Disease (GBD) study [14]. The GBD 2019 study provides the most recent epidemiological estimates and DALY calculations for 369 causes of disease and injuries and 87 risk factors for 204 countries and territories [13, 15]. However, estimates for rare diseases are frequently not reported separately, as most are reported in broader GBD cause of disease categories, such as the category “Other neurological disorders” or “Other chronic respiratory diseases” [15]. Additionally, the GBD study provides estimates for common causes of disease, including its variants. However, these variants might affect populations at a different scale and differ in aetiology, clinical presentation and progression, and/or clinical management (e.g., urticaria and myocarditis) [16, 17].

An overview of studies that have estimated DALYs for rare diseases can give insight into the epidemiological data sources and methodological approaches that have been used to assess the burden of rare diseases. Therefore, the aim of this systematic literature review was to provide an overview of the studies that estimated DALYs resulting from chronic non-communicable rare diseases (CNCRD). The following research questions were addressed:

- How many burden of CNCRD studies have been conducted since 1990, and in which region/country and for which CNCRDs have these studies been performed?
- Which mortality and morbidity data input sources have been used in burden of CNCRD studies?
- Which methodological approaches have been used in order to calculate YLL, YLD, and/or DALY for burden of CNCRD?
- What was the DALY per case estimated in each burden of CNCRD study?

## Methods

This systematic literature review was performed according to the Preferred Reporting Items for Systematic Reviews and Meta-Analyses (PRISMA) guidelines [18]. We registered the protocol of the present systematic review in the International Prospective Register of Systematic Reviews (PROSPERO) database [19], under the ID number CRD42022324960.

### Search strategy and data sources

A librarian from the Erasmus MC medical library was involved in the development of the search strategy, on 10 March 2022. We performed the search in two electronic bibliographic databases, namely, Embase and Medline. The search strategy and its search strings can be found in the supplementary materials.

In addition, we performed a grey literature search on 24 March 2022. The grey literature search involved databases and websites based on literature recommendations [20] as well as organization’s websites focused on population health and/or rare diseases, namely the World Health Organization (WHO), the European Medicines Agency (EMA), the U.S Food and Drug Administration (FDA), the National Organization for Rare Diseases (NORD), Genetic and Rare Diseases information center (GARD), European Organisation for Rare Diseases (Eurordis) and the European Reference Network for Hereditary Metabolic Rare Diseases (MetabERN). Finally, we screened the references of the included papers and the references of studies that determined the impact of all rare diseases, including communicable and non-communicable diseases, in a certain region.

Conference and paper abstracts, editorials, and general correspondence presenting disease burden findings from CNCRD were also included. For the identified abstracts, we searched for the corresponding full-text. If the same study was both recognized as an abstract and full text, we only included the full-text of the study. Furthermore, no geographic and language restrictions were applied. We included studies if they had been published between January 1990, the decade that the DALY concept was introduced, and 10 March 2022, when the search strategy was developed.

### Inclusion and exclusion criteria

In this systematic literature review we included studies that assessed the burden of rare diseases using DALY, and/or YLD and YLL as defined within the DALY framework. We considered studies in which the rare disease was listed on the Orphanet’s and/or GARD’s websites as a recognized rare disease. We limited this systematic literature review to studies that assessed the burden of CNCRD. We included chronic diseases that matched the chronic disease definition according to the Friedman et al., 2008 study [21], which defined chronic diseases as “*lasting 12 months or longer*,* imposing limitations on self-care*,* independent living and social interactions*,* and/or resulting in the need for ongoing medical intervention”*. We excluded studies that assessed the burden of communicable or occupational diseases, and diseases related to injuries or risk factors, because the affected proportion of the population, and consequently meeting the definition of rare diseases, may vary widely over time and by geographical region. We excluded studies estimating the burden of rare cancers as most of them have a sub-acute clinical course (characterized by a rapid progression and high mortality rates) due to the lack of treatments and delayed diagnosis [22, 23], not matching Friedman’s et al. definition of a chronic disease. However, proliferative but non-malignant disorders were included. In total, 3,888 diseases met our eligibility criterion and were included in the search strategy.

We included cost of illness studies that followed the burden of disease approach, assessing DALYs for rare diseases. However, we excluded cost-effectiveness and cost-benefit analysis due to insufficient information on burden of disease methods. We also excluded studies that assessed DALYs due to delayed or lack of healthcare access, as those were beyond the scope of this study. Finally, we excluded studies that mentioned DALY, YLL or YLD but did not follow the burden of disease approach, using other formulae to obtain their estimates.

### Data screening and extraction

The screening of titles, abstracts, and full texts was carried out by two independent researchers, (CCO and PC). The same researchers (CCO and PC) extracted data independently from the included studies using an adjusted data extraction excel spreadsheet form based on a previous systematic literature review [24]. Finally, the completed data extraction forms were compared, and the final version of the data extraction form was obtained, in mutual agreement. Any disagreements regarding the selection and extraction steps were solved through debate and, if needed, by the study supervisor (JAH). The data extraction form, including the extracted data, and the definitions of each extracted item are provided as supplementary material. The software used for compiling, screening, and selecting the studies to be included for data synthesis was EndNote version 20.0.1.

### Data synthesis

The data synthesis of the included studies depicts the study characteristics, methods and data input sources using tables. Moreover, figures were presented to capture the studies distribution by geographic coverage, publication year, and group of disease(s) it focused on, according to the chapter names of the 11th revision of the International Statistical Classification of Diseases and Related Health Problems (ICD-11) [25]. The extracted information on the general study characteristics that was not displayed in the data synthesis was included in the supplementary material (e.g. related to study design, study population and detailed methodological choices). Studies were classified as independent or linked studies. The term ‘independent study’ reflects a single or multi-country study for which researchers independently calculated and analyzed YLL, YLD and/or DALY caused by CNCRDs. On the other hand, ‘linked studies’ refers to any single or multi-country study that presented estimates or secondary analyses from collaborative initiatives, such as the GBD study.

The data extraction and synthesis were managed with Microsoft Excel 2016. The figure displaying the geographical distribution of the included studies was created using the online tool, Datawrapper [26].

### DALY per case calculation

For each study, we determined the DALY per incident or prevalent case and displayed these in a figure.

The DALY per case was calculated by using information provided in the methods and/or results section of the study. If the study provided references for further consultation on sample size and/or prevalence/incidence estimates on which the study was based on, we extracted that information for our DALY per case estimation. To following formulas were used to calculate DALY per case, depending on the information provided:

$$\:\frac{Crude\:DALY}{Number\:of\:affected\:individuals}=DALY\:per\:case$$

OR

$$\:\frac{DALY\:rate\:\left(per\:100\:000\right)}{Prevalence\:or\:Incidence\:rate\:\left(per\:100\:000\right)}=DALY\:per\:case$$

If the same study reported DALYs for multiple years separately, then only the most recent DALY estimates were used for the DALY per case calculation. Additionally, if estimates for multiple diseases were provided separately, DALY per case was calculated per each disease included in the study.

### Quality of reporting assessment

We used the Guidelines for Accurate and Transparent Health Estimates Reporting (GATHER) statement to assess the quality of reporting of the included full-text studies. The GATHER statement was developed to motivate best reporting practices for studies that calculate health estimates [27]. The quality assessment table can be found in the supplementary material.

## Results

### Literature search

**Fig. 1:**
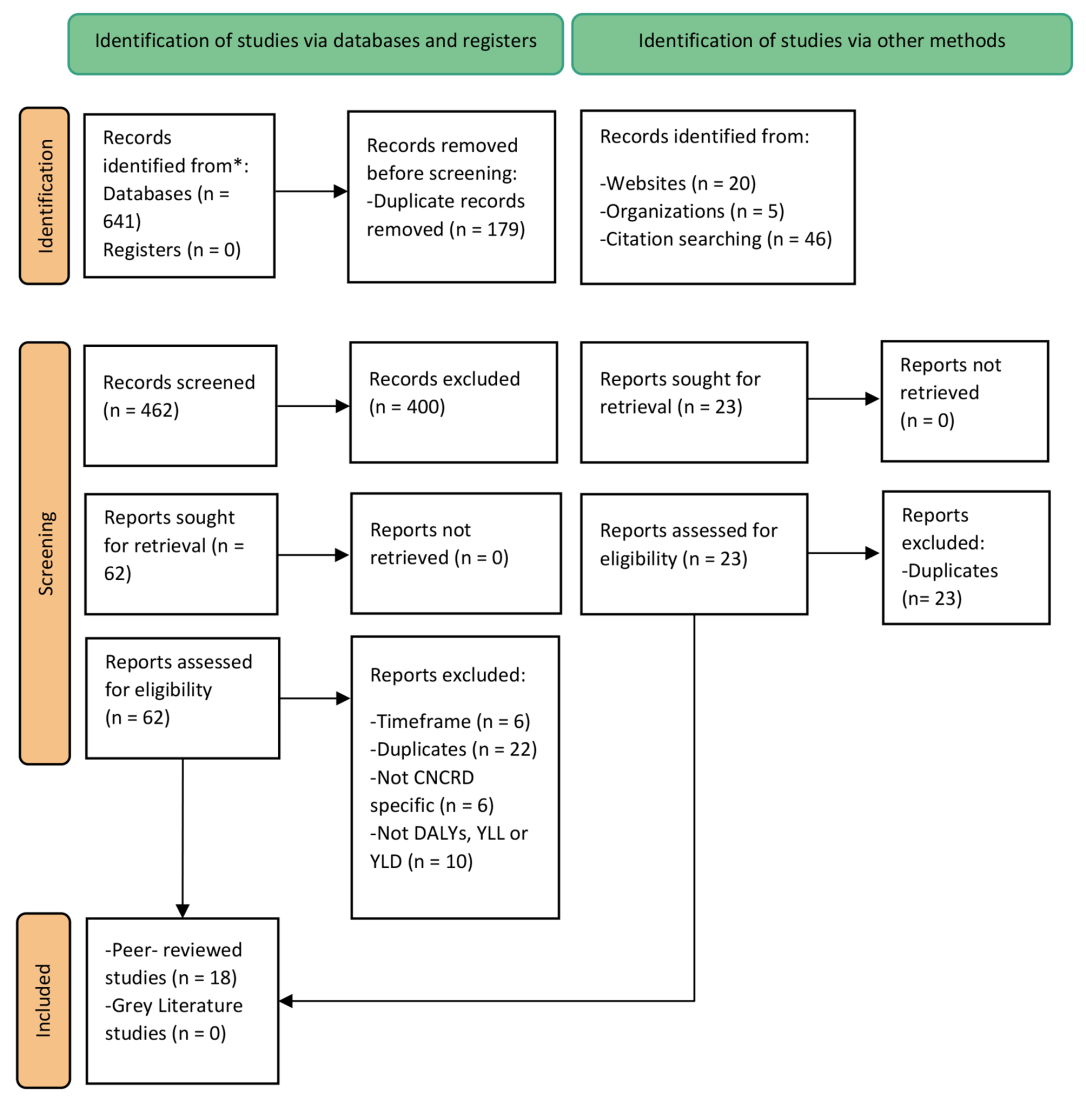
Flowchart of the literature search (PRISMA)

Figure 1 presents an overview of the search and screening strategy performed in this systematic literature review, including the main reasons for exclusion based on eligibility criteria. We identified a total of 641 records via searches in databases Embase and Medline and 71 records via other methods. We screened 533 titles and abstracts. We then assessed 85 studies for eligibility according to the study inclusion and exclusion criteria. The main reasons for exclusion were due to duplicates, publication date previous than 1990 (“timeframe”) [28–33], burden of disease studies not focused in CNCRDs (“Not CNCRD specific”) [34–39], and studies that reported different metrics than DALY, or YLD and YLL according to the DALY framework (“Not DALYs, YLL or YLD”) [40–49]. In total, we included 18 peer-reviewed studies [13, 50–66]. Out of the 18 included studies, five did not have the full-text available at the at the inclusion time criteria and thus, these were included as abstracts [50–52, 56, 60].

### Study characteristics

Table 1 presents an overview of the characteristics of the 18 included studies that provided 26 different DALY and/or YLD and YLL estimates. Two out of the 18 studies were limited to YLLs [60, 61]. On the other hand, five studies provided DALYs, but not YLLs or YLDs [52, 55, 57, 63, 65]. Furthermore, the GBD study reported DALYs, YLLs and YLDs for two non-fatal CNCRD diseases (Klinefelter and Turner syndrome), meaning YLL was provided as a null estimate. Out of 18 studies, 16 were independent studies while only two were linked studies [13, 55]. However, out of 26 DALY and/or YLD and YLL estimates, 10 (38.5%) were the result of linked studies, while 16 were derived by independent studies.

**Table 1:** Overview of burden of CNCRD studies

Author(s)	Reference time period	Geographic coverage	Disease(s) included	Reported metric(s)	
			Name(s)		
Abolhassani et al. [50]	1985–2008	Iran	Common variable immunodeficiency disorders	YLL, YLD, DALY	
Acuna et al. [51]	August 2015 to mid-December 2016	Roxas City Capiz, Philippines	X-Linked dystonia-parkinsonism	YLL, YLD, DALY	
Café et al. [52]	2017	Portugal	Hemophilia A	YLL, YLD, DALY	
Chung et al. [54]	2008	Korea	Multiple sclerosis	YLL, YLD, DALY	
Costa et al. [56]	2019	Portugal	Spinal Muscular Atrophy	YLL, YLD, DALY	
GBD 2016 Motor Neuron Disease collaborators [55]	2016	Global	Motor neuron diseases*	DALY	
	2019*¹	Global	Down syndrome	YLL, YLD, DALY	
	2019*¹	Global	Klinefelter syndrome	YLD, DALY	
	2019*¹	Global	Motor Neuron Disease	YLL, YLD, DALY	
	2019*¹	Global	Multiple sclerosis	YLL, YLD, DALY	
	2019*¹	Global	Neural tube defects	YLL, YLD, DALY	
	2019*¹	Global	Orofacial cleft	YLL, YLD, DALY	
	2019*¹	Global	Sickle cell disease	YLL, YLD, DALY	
	2019*¹	Global	Thalassemia	YLL, YLD, DALY	
	2019*¹	Global	Turner syndrome	YLD, DALY	
Guojun et al. [57]	2017–2018	China	Multiple sclerosis	DALY	
Henrard et al. [58]	2011	Belgium	Hemophilia (A and B)	YLL, YLD, DALY	
Inês et al. [59]	2016	Portugal	Hereditary transthyretin amyloidosis polyneuropathy	YLL, YLD, DALY	
Janphram et al. [60]	2011–2020	Thailand	Glomerulonephritis *²	YLL	
Kansal et al. [61]	NA	Global	Frontotemporal dementia	YLL	
Liu et al. [62]	2013	Shandong Province, China	Multiple sclerosis	YLL, YLD, DALY	
Odnoletkova et al. [63]	2004–2014	Western Europe	Common variable immunodeficiency disorders	DALY	
Pinto et al. [52]	2018	Brazil	Sickle cell disease	DALY	
Siddiqi et al. [64]	2007	USA	Hemophilia (A and B)	YLL, YLD, DALY	
Villaquiran-Torres et al. [65]	NR	Colombia	Pulmonary arterial hypertension	DALY	
	NR	Colombia	Chronic thromboembolic pulmonary hypertension	DALY	
Villaverde-Hueso et al. [66]	2001	Spain	Scleroderma	YLL, YLD, DALY	

*Amyotrophic lateral sclerosis, spinal muscular atrophy, hereditary spastic paraplegia, primary lateral sclerosis, progressive muscular atrophy and pseudobulbar palsy

*¹ Only the latest estimates were included (yearly measures are available from 1990). However, GBD 2016 study was also included in the analysis as it provided additional systematic analysis on severity and YLD estimations, which are rare disease-specific

*²Primary glomerulonephritis (IgA nephropathy, focal segmental glomerulosclerosis, membranous nephropathy and minimal change disease) and secondary GN (Lupus nephritis and Anti-neutrophil cytoplasmic antibody-associated glomerulonephritis)

Note^1^: Reference time period refers to the months/year(s) the information used in the study was collected. Geographic coverage refers to where the information used in the study was collected

Note^2^: YLL (Years of Life Lost); YLD (Years Lived with Disability); DALY (Disability-Adjusted Life Year); NA (Not applicable)

Seven of the 13 studies with full-text available reported receiving funding. Out of these, six relied on non-profit organizations (NPO) as a funding source [13, 55, 61, 63] and/or research fellowships [61, 62] and one was funded by pharmaceutical industries [53].

Most of the studies were performed as observational studies, with three exceptions: two studies estimated DALYs through simulation studies [58, 65] and another study was a Delphi study [53].

#### Number of studies over time

**Fig. 2:**
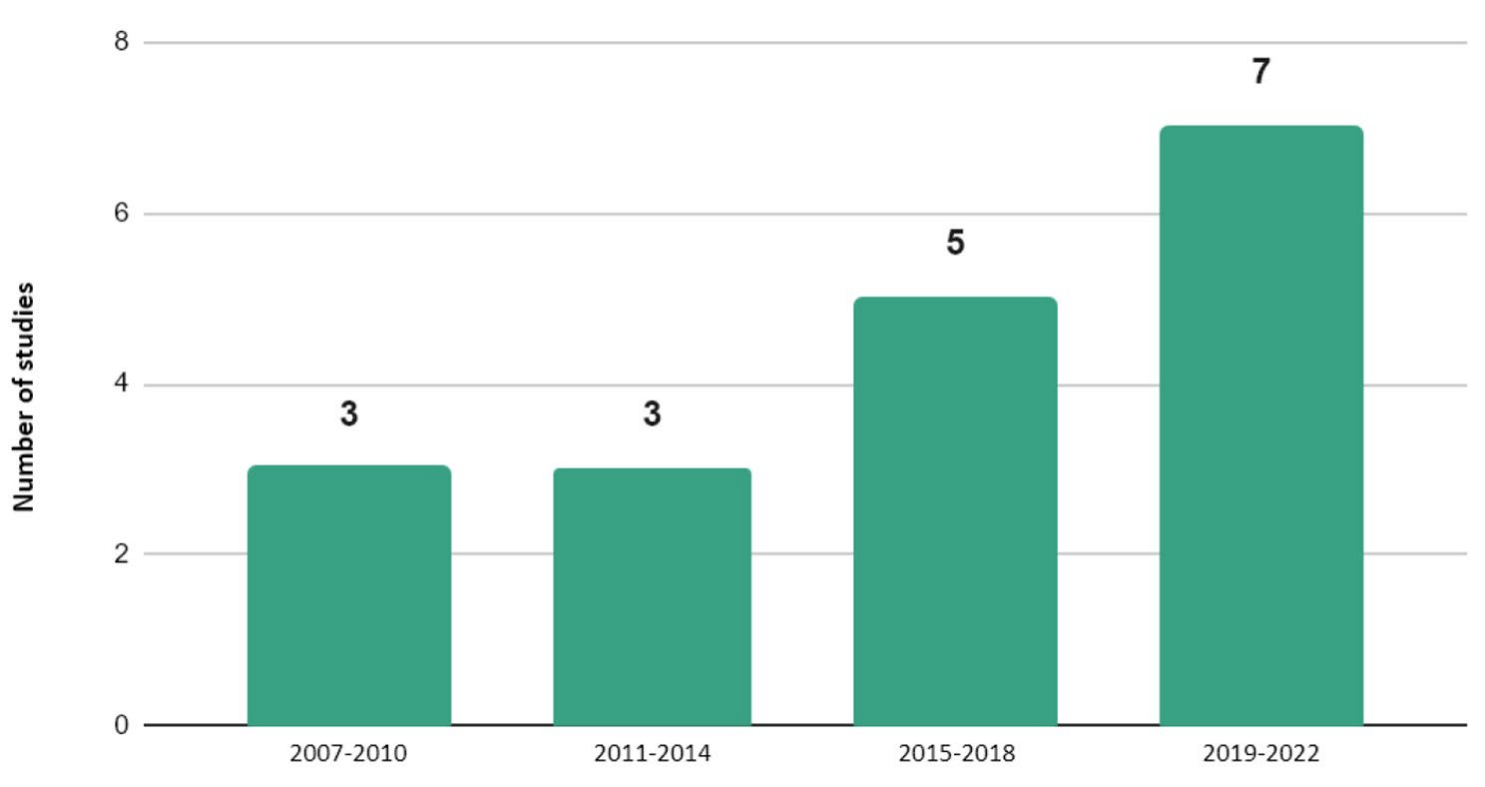
Number of burden of CNCRD disease studies, per 4-year time interval* *Only independent studies are presented in the figure

Figure 2 depicts the number of studies by year of publication, aggregated into 4-year time-periods. The time intervals displayed in Fig. 2 start in 2007, as no independent CNCRD studies were published before this date, till early 2022, when the search was conducted. Overall, the number of studies estimating DALYs associated with CNCRD has increased over time, varying from the lowest number from 2007 to 2010 and 2011–2014 (*n* = 3) to the highest, from 2019 to 2022 period (*n* = 7).

#### Number of studies per geographical region

**Fig. 3:**
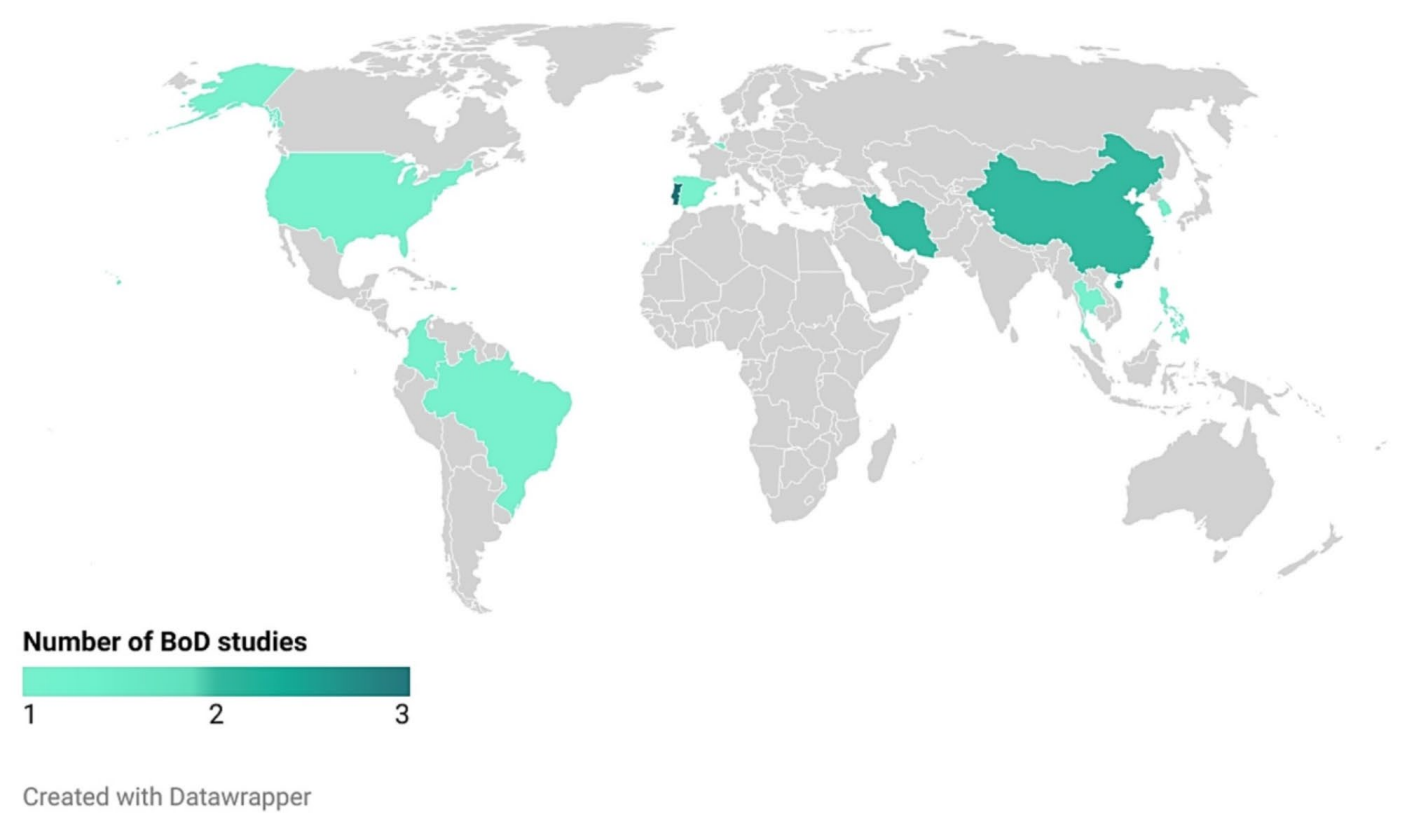
Number of burden of CNCRD disease studies, per geographic region* *Solely single country studies are presented in the figure

Figure 3 shows the geographic distribution of the included CNCRD studies. Of the 18 studies included in this review, four studies were multi-country studies [13, 55, 61, 63]. The remaining studies (*n* = 14) were performed across 11 countries, namely Belgium, Brazil, China, Colombia, Iran, Korea, Philippines, Portugal, Spain, Thailand and the United States of America [50–54, 56–60, 62, 64–66]. The country with highest number of studies was Portugal (*n* = 3) [51, 53, 59]. Additionally, all single country studies were performed in middle (*n* = 7) [50, 52, 54, 57, 60, 62, 65] or high (*n* = 7) [51, 53, 56, 58, 59, 64, 66] income countries.

#### Rare disease categories

**Fig. 4:**
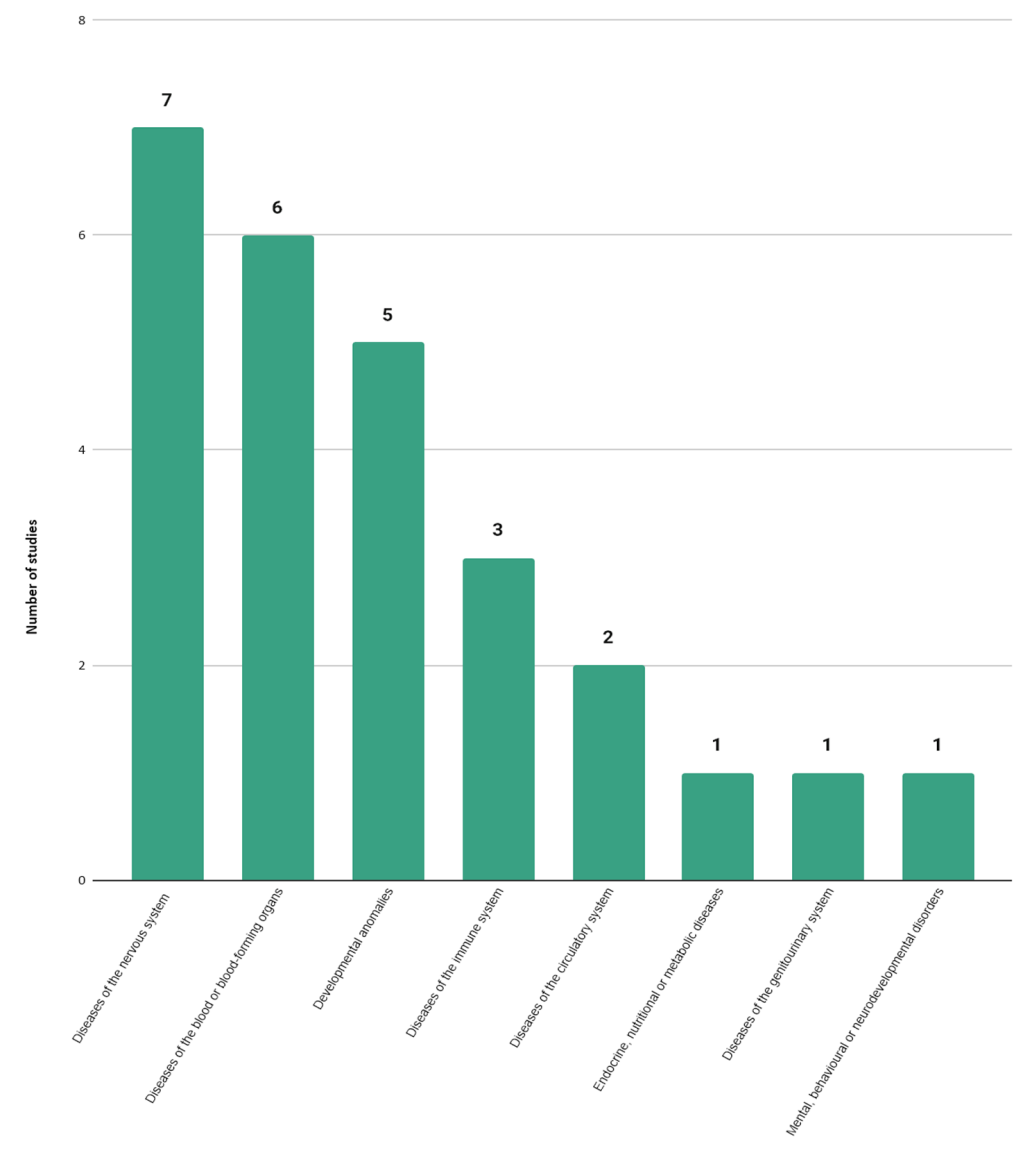
Number of burden of CNCRD disease studies e, per cause of disease category *According to the chapters of the 11th revision of the International Classification of Diseases (ICD)

The 18 included studies covered 19 different rare diseases (see Table 1). Common variable immunodeficiency disorders [50, 63], hemophilia [53, 58, 64], sickle cell disease [13, 52], motor neuron diseases [13, 55] and multiple sclerosis [54, 55, 62] were covered in more than one study. Figure 4 shows that rare diseases falling in the disease category “Diseases of the nervous system” were most frequently studied (n = 7) [13, 51, 54–56, 62], followed by “Diseases of the blood or blood-forming organs” (n = 6) [13, 52, 53, 58, 64] and ‘Developmental anomalies” (n = 5) [13, 56].

### Data input sources

Table 2 presents the data input sources used to inform on incidence/prevalence and morbidity, and mortality associated with the rare disease. According to our findings, independent studies relied on a lower number of data sources than linked studies (Table 2). Literature was the most common input source to inform on incidence/prevalence and mortality (*n* = 10) [13, 52, 54, 55, 58, 59, 61, 64–66], followed by population-based registries (patient or diseases registries) (*n* = 8) [13, 50, 55, 58–60, 62, 63], national vital statistics/census (*n* = 8) [13, 52, 55, 60, 64–66], expert opinion (*n* = 5) [52, 53, 56, 57, 66] and convenience samples (hospital records or surveys) (*n* = 4) [51, 56, 57, 62].

**Table 2:** Data input sources used to calculate YLL, YLD and DALY in burden of CNCRD studies

Author(s)	Disease(s) included	Data input sources	
		For prevalence/incidence and morbidity	For mortality
Abolhassani et al. [50]	Common variable immunodeficiency disorders	Disease Registry (Iranian Primary Immunodeficiency Registry at the Children’s Medical Center)	Disease Registry (Iranian Primary Immunodeficiency Registry at the Children’s Medical Center)
Acuna et al. [51]	X-Linked dystonia-parkinsonism	Hospital Records (Health Centrum, Movement Disorders Clinic)	Hospital Records (Health Centrum, Movement Disorders Clinic)
Café et al. [53]	Hemophilia A	Delphi method (Expert Delbecq panel) and Survey	Delphi method (Expert Delbecq panel)
Chung et al. [54]	Multiple sclerosis	Literature (Kim et al. 2010; Torisu et al. 2010, Granieri et al. 2007)	Literature (Ekestern et al. 2004)
Costa et al. [56]	Spinal Muscular Atrophy	Hospital Records (NR) and expert’s opinion	Hospital Records (NR) and expert’s opinion
GBD 2016 Motor Neuron Disease collaborators [55]	Motor neuron diseases *	Literature (systematic review), Insurance claims (USA), disease registry (ALS Clinical Trials (PRO-ACT))	National vital statistics and verbal autopsy (GBD)
GBD 2019 Diseases and Injuries Collaborators [13]	Down syndrome, klinefelter syndrome, motor neuron diseases*, multiple sclerosis, neural tube defects, orofacial cleft, thalassemia and sickle cell disease	Literature, censuses, national vital statistics, disease registries, health service use, satellite imaging, disease notifications	Literature, censuses, national vital vital statistics, disease registries, satellite imaging, disease notifications
Guojun et al. [57]	Multiple sclerosis	Surveys (50 centers across China) and expert’s opinion	NR
Henrard et al. [58]	Hemophilia (A and B)	Disease Registry (Belgian Haemophilia Association) and Literature (Taruscio et al. 1990, Soucie et al. 1998, Stonebrakeret al 2010 and Stonebraker et al. 2012)	Literature (Plug et al. 2006)
Inês et al. [59]	Hereditary transthyretin amyloidosis polyneuropathy	Literature (Ines et al. 2018, Coelho et al. 2018) and Disease Registries (2 national reference centers)	Literature (Ines et al. 2018, Coelho et al. 2018)
Janphram et al. [60]	Glomerulonephritis *^1^	Disease Registry (Ramathibodi Hospital Glomerular Registry)	Disease Registry (Ramathibodi Hospital Glomerular Registry) and National vital statistics
Kansal et al. [61]	Frontotemporal dementia	Literature (systematic review)	Literature (systematic review)
Liu et al. [62]	Multiple sclerosis	Hospital Records (university-affiliated hospitals, and hospitals from 17 cities in Shandong Province)	Patient Registry (Chinese Center for Disease Control and Prevention)
Odnoletkova et al. [63]	Common variable immunodeficiency disorders	Disease Registry (European Society for Immunodeficiencies registry data- ESID)	Disease Registry (European Society for Immunodeficiencies registry data- ESID)
Pinto et al. [52]	Sickle cell disease	Literature, National vital statistics (Brazilian governmental healthcare public database) and expert’s opinion	National vital statistics (Brazilian governmental healthcare public database)
Siddiqi et al. [64]	Hemophilia (A and B)	Literature (Soucie et al. 1998), Patient Registry (CDC) and censuses (U.S. Census Bureau)	Literature (Soucie et al. 1998)
Villaquiran-Torres et al. [65]	Pulmonary arterial hypertension and Chronic thromboembolic pulmonary hypertension	Literature (NR), GBD 2015 and National vital statistics	Literature (NR), GBD 2015 and National vital statistics
Villaverde-Hueso et al. [66]	Scleroderma	Literature (Silman et al. 1988), National Vital Statistics and expert’s opinion	National Statistics Institute

* Amyotrophic lateral sclerosis, spinal muscular atrophy, hereditary spastic paraplegia, primary lateral sclerosis, progressive muscular atrophy and pseudobulbar palsy

*^1^ Primary glomerulonephritis (IgA nephropathy, focal segmental glomerulosclerosis, membranous nephropathy and minimal change disease) and secondary GN (Lupus nephritis and Anti-neutrophil cytoplasmic antibody-associated glomerulonephritis)

Note: NR (Not Reported)

Some studies that covered the same disease used the same type of data input sources. This is the case for studies that have estimated DALYs for common variable immunodeficiency disorders as both used data from disease registries. Moreover, both motor neuron diseases and sickle cell diseases studies used multiple data sources. These studies were conducted more recently (from 2016) and/or correspond to linked studies [13, 52, 55]. On the contrary, burden of disease studies for multiple sclerosis and hemophilia relied on distinct data sources, namely hospital records, a disease registry and surveys [13, 54, 57, 62], and literature, experts and a disease registry [53, 58, 64], respectively.

### Methodological design choices

Table 3 provides an overview of the methodological design choices made to estimate YLL, YLD and/or DALYs in the included CNCRD studies.

**Table 3:** Methods used to calculate YLL, YLD and DALY in Burden of CNCRD disease studies

Author(s)	Disease(s) included	Full-text	YLL methods	YLD methods			Age weighting	Time discounting
			Life expectancy source	Perspective of YLD estimates	Study developed own DWs: Yes/No [Source]	Severity distribution		
Abolhassani et al. [50]	Common variable immunodeficiency disorders	✓	National Life Table	Incidence-based	No [Dutch DWs]	☓	✓	3%
Acuna et al. [51]	X-Linked dystonia-parkinsonism	☓	NR	Prevalence-based	No [NR]	☓	NR	NR
Café et al. [53]	Hemophilia A	✓	Aspirational table (West level 26 standard life table)	Incidence-based	Yes [SF-36 (Carvalhosa et al. 2014)]	National	✓	☓/3%
Chung et al. [54]	Multiple sclerosis	✓	NR	Incidence-based	No [Korean DWs]	National	✓	3%
Costa et al. [56]	Spinal Muscular Atrophy	☓	NR	Prevalence-based	NR	NR	NR	NR
GBD 2016 Motor Neuron Disease collaborators [55]	Motor neuron diseases *	✓	Aspirational table (GBD model life table)	Prevalence-based	Yes [ALSFRS-R]	Global	☓	☓
GBD 2019 Diseases and Injuries Collaborators [13]	Down syndrome, klinefelter syndrome, motor neuron diseases*, multiple sclerosis, neural tube defects, orofacial cleft, thalassemia and sickle cell disease	✓	Aspirational table (GBD model life table)	Prevalence-based	Yes [GBD DWs]	Global	☓	☓
Guojun et al. [57]	Multiple sclerosis	✓	Aspirational table (WHO standard life expectancy)	Incidence-based	No [GBD DWs]	☓	✓	3%
Henrard et al. [58]	Hemophilia (A and B)	✓	Aspirational table (Coale-Demeny West model life table)	Incidence-based	Yes [SF-36 (carvalhosa et al. 2014) and KINDL score (khair et al. 2012)]	National	✓/ ☓*^2^	1,5%/ 3%*^2^
Inês et al. [59]	Hereditary transthyretin amyloidosis polyneuropathy	✓	National Life Table	Prevalence-based	Yes [EQ-5D-3 L (Monica et al. 2015)]	☓	☓	☓
Janphram et al. [60]	Glomerulonephritis *^3^	☓	National Life Table	NA	NA	NA	NA	NA
Kansal et al. [61]	Frontotemporal dementia	✓	National and Aspirational tables (Human Life-Table Database; WHO life tables; National Life table (Canada and India))	NA	NA	NA	NA	NA
Liu et al. [62]	Multiple sclerosis	✓	Aspirational table (Coale-Demeny West model life table)	Incidence-based	No [Korean DWs]	☓	✓	3%
Odnoletkova et al. [63]	Common variable immunodeficiency disorders	✓	Aspirational table (GBD model life table)	Prevalence-based	No [GBD DWs]	☓	☓	☓
Pinto et al. [52]	Sickle cell disease	☓	NR	Prevalence-based	NR	NR	NR	NR
Siddiqi et al. [64]	Hemophilia (A and B)	✓	NR	Incidence-based	Yes [EQ-5D (Universal Data Collection project (UDC))]	National	✓	3%
Villaquiran-Torres et al. [65]	Pulmonary arterial hypertension and Chronic thromboembolic pulmonary hypertension	☓	NR	Incidence-based	No [GBD DWs]	NR	NR	NR
Villaverde-Hueso et al. [66]	Scleroderma	✓	Aspirational table (Princeton Model Life Table with Level West 26 modified)	Incidence-based	No [Dutch DWs]	National	✓	3%

* Amyotrophic lateral sclerosis, spinal muscular atrophy, hereditary spastic paraplegia, primary lateral sclerosis, progressive muscular atrophy and pseudobulbar palsy

*^1^ The study provided estimates with and without age weighing and with different time discounting rates (scenario analysis)

*^2^ Primary glomerulonephritis (IgA nephropathy, focal segmental glomerulosclerosis, membranous nephropathy and minimal change disease) and secondary GN (Lupus nephritis and Anti-neutrophil cytoplasmic antibody-associated glomerulonephritis)

Note^1^: Age-weighing places a lower weight on a year of healthy life lived at younger and older ages, and a higher value on working ages, implying the value of life is age-dependent. Similarly, time discounting places a higher value on life years closer to the present than the life years lived in the future [14, 87, 88].

Note^2^: YLL (Years of Life Lost); YLD (Years Lived with Disability); DALY (Disability-Adjusted Life Year); DW (Disability-Weights); GBD (Global Burden of Disease); NR (Not Reported); NA (Not applicable)

#### Years of life lost

From 18 studies, four studies performed their YLL calculations using national life tables (representative of the corresponding population) [50, 59–61], while nine studies used aspirational life tables (representing the ideal standard) [13, 53, 55, 57, 58, 61–63, 66]. Exceptionally, one multiple-country study used different types of life tables to estimate YLL per included country [61]. Life tables were not reported in six out of the 18 included for YLL estimation However, of these studies, four were abstracts [51, 52, 56, 65] and two full-text studies failed to report the life table used for their calculations [54, 64].

#### Years lived with disability

Of the 18 studies, 16 calculated YLDs. In nine studies (56.3%), incidence data were used to calculate YLDs [50, 53, 54, 57, 58, 62, 64–66], whereas in seven studies (43.7%) prevalence data were used [13, 51, 52, 55, 56, 59, 63]. Out of these 16 studies, two abstracts did not include any further methodological information used for YLD calculation [52, 65]. Of the remaining 14 studies, six developed their own disability weights, based on health-related quality of life data [13, 53, 55, 58, 59, 64]. Eight studies used existing disability weights, such as the GBD disability weights (*n* = 3) [57, 63, 65], the Dutch disability weights (*n* = 2) [50, 66] or Korean disability weights (*n* = 2) [54, 62]. The remaining study was an abstract that did not report the disability weight source used [51]. Out of the 14 studies which have reported YLD methods, seven used severity distributions, taking into account different levels of severity of the disease and using disability weights that reflected these difference in severity level [13, 53–55, 64, 66].

#### Age-weighting and time discounting

Age-weighting and time discounting was applied in eight of the 16 studies [50, 53, 54, 57, 58, 62, 64, 66]. Moreover, of these eight studies, two reported both DALY estimates with and without age weighting and/or with different time discounting rates (0% and 3% or 1.5% and 3%) [53, 58].

### DALY per case

**Fig. 5:**
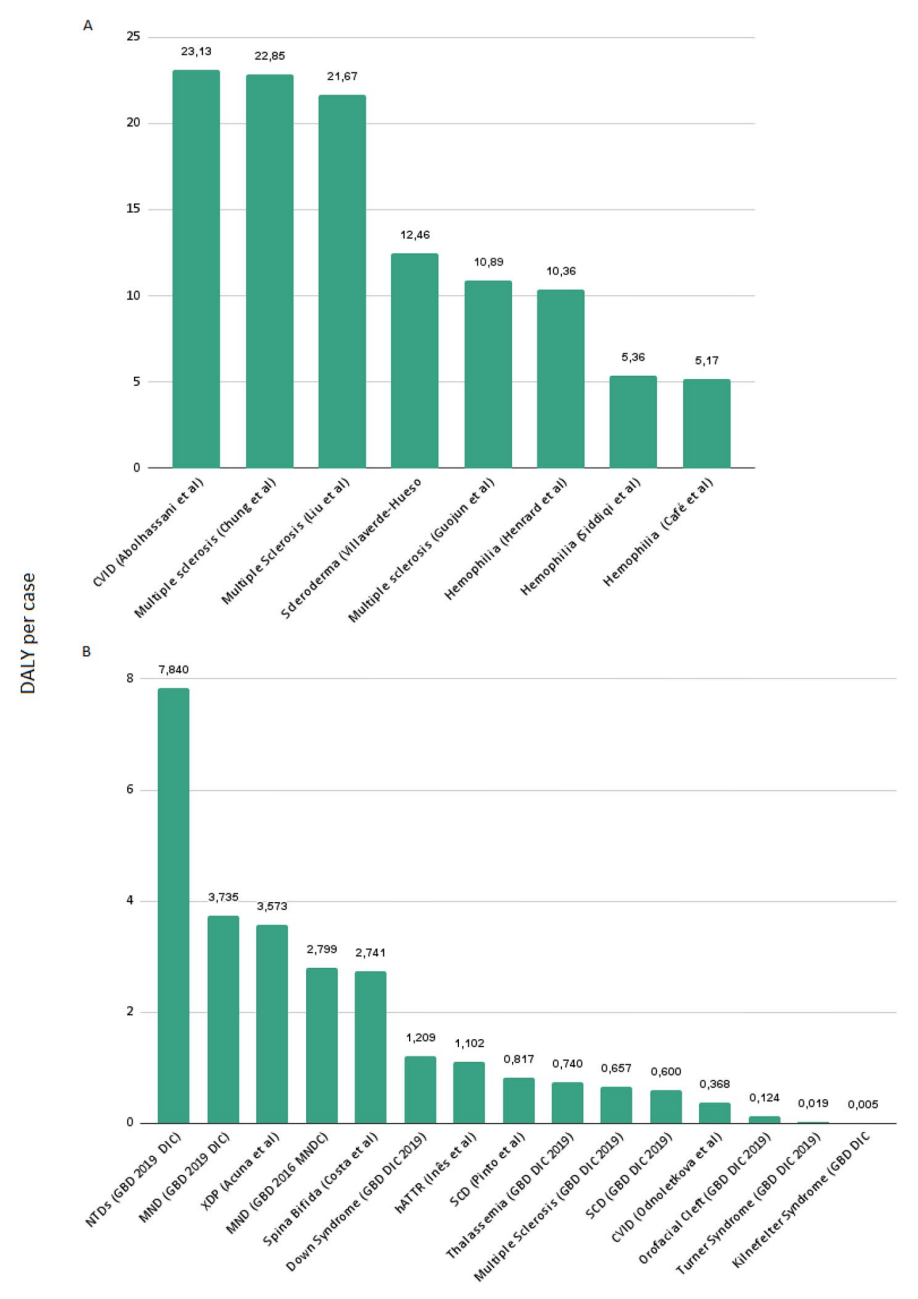
DALY per case*¹ *¹ (**A**) DALY per incident case (lifetime); (**B**) DALY per prevalent case (annual)

Figure 5 displays the DALY per incident case (A) and prevalent case (B) of each study. The DALY per case varied from 5.2 to 23.1 in studies that used incidence data to determine DALYs, and from 0.005 to 7.8 in studies that used a prevalence data to determine DALYs. Some studies calculated DALYs for the same disease using the similar methods, yet DALY per cases varied, namely for multiple sclerosis (10.9, 21,7 and 22.9 DALY per incident case) [54, 57, 62], hemophilia (5.2, 5.4 and 10.4 DALY per incident case) [53, 58, 64], motor neuron diseases (2.8 and 3.7 DALY per prevalent case) [13, 55] and sickle cell disease (0.6 and 0.8 DALY per prevalent case) [13, 52].

The diseases with the highest DALY per incident case (higher than 20 DALY per affected individual) were common variable immune disorders (23.1 DALY per case) [50] and multiple sclerosis (22.9 and 21.7 DALY per case) [54, 62], using an incidence-based approach.

The diseases with the highest estimated DALY per prevalent case were neural tube defects disorders, accounting for almost 8 DALY per year [13], followed by motor neuron diseases (3.7 DALY per case) [13] and linked dystonia-parkinsonism (3.6 DALY per case) [51].

### Quality of the reporting assessment

Six out of the 12 included studies with full text available have covered between 55% and 70% of all the recommended GATHER items. Additionally, four studies have achieved around 72% of the reporting requirements. Two studies covered between 75 and 85% of all items. Finally, two studies have reported all the items in the list, both were linked to the GBD study. We observed that the items most frequently not reported were related to providing secondary data sources (life tables for YLL calculation and disability weights data source for YLD calculation), file formats that could be efficiently extracted and methods and estimates of uncertainty analysis.

## Discussion

This systematic literature review provided an overview of the number of burden of CNCRD studies and their studies’ characteristics, data input sources and methodological approaches. It was found that the number of burden of disease studies for rare diseases is low as compared with the number of studies observed in previous systematic reviews of burden of non-communicable diseases, infectious diseases or injuries [67–69]. Furthermore, the distribution of rare diseases per disease group showed that many cause of disease categories were not represented in the included studies. This finding may indicate the predominance of rare diseases in certain disease groups, but could also be an indication of greater awareness of certain rare diseases. Reasons for the low number of studies may be the lack of epidemiological data collection, challenges in rare disease case reporting, lack of knowledge regarding the rare disease clinical presentation and progression, patients’ quality of life, lack of familiarity of researchers in the field of rare disease with the DALY concept, and lack of funding for rare disease research [70–72]. Foremost, challenges in case reporting and data collection have been linked to the lack of disease codes (e.g., ICD codes) as basis to classify and record diseases in medical records or registries references [73]. For these reasons, data on incidence and prevalence of rare diseases might be difficult to obtain. On the other hand, the lack of knowledge among medical professionals regarding the existence and/or clinical presentation of a rare disease may lead to the underdiagnoses of rare disease patients which, in its turn, may lead to underestimation of incidence or prevalence of a rare disease, which can then result in underestimation of DALY rates for rare diseases in the population [73–76]. Possible strategies to tackle the low number of burden of CNCRD studies are the harmonization of rare disease terminology and case reporting, and the systematic collection of health-related quality of life data through scaled assessments (e.g., patient reported outcome measurements-PROMs) [77].

The majority of the studies identified in this systematic literature review were performed in middle or high income countries, while the remaining were performed at a global level. Moreover, some diseases included in this study met the definition of a rare disease in high/middle income regions but not in lower income regions. This is the case for sickle cell disease with a prevalence that ranges from 50 to 990 cases per 100,000 individuals in lower income countries, which is almost three times higher than the prevalence of Parkinson’s disease around the world [15]. Thus, these findings may reflect that researchers from low income countries face even more challenges in performing research on rare or uncommon diseases compared with researchers from middle and high income countries [78]. In this study, 12 out of 26 CNCRD DALY estimates (46%) covered multiple countries (Global or Western Europe). This finding may be explained by the fact that for research on rare diseases, the availability of a sample of rare disease patients which represents its rare disease population might require the recruitment of patients from multiple countries and/or the involvement of rare disease experts that are spread across different countries [79]. Even though the DALY calculation does not require a minimum sample size, it is important to achieve generalizable and relevant DALY estimates of rare diseases, suitable for comparisons between countries and over time, economic evaluations, and other outputs. For all the reasons mentioned above, this study’s finding underlines the importance of international cooperation to further rare disease research, globally [80, 81]. At the basis of this is the need to facilitate data sharing across borders [71, 82].

The overview of data input sources revealed that, unlike burden of disease studies for common diseases, determination of the burden of rare disease relies more on literature and expert opinion, which could lead to inaccurate estimations [68, 83]. This conclusion is consistent with previous reports on the difficulty of gathering data in the field of rare diseases [71, 75].

In contrast to data input sources, we observed considerable variations across CNCRD studies estimating YLL, and/or YLD and DALY. Different life tables were used to calculate YLL. Most studies used aspirational life tables (e.g. GBD or WHO life tables) which assume an ideal standard for life expectancy [84]. This ideal standard is derived from the highest life expectancy achieved, approximating the potential biological life expectancy per population [85]. In contrast, national life tables reflect current mortality and reflective of a country’s past and current circumstances. However, YLL refer to the future years lost due to past or current premature mortality [68, 84]. In addition, the use of different life tables hampers international comparisons of DALYs due to rare disease. Therefore we recommend the use of aspirational life tables when calculating YLLs, in order to facilitate international comparisons, and calculate YLL that reflect future years of life lost.

Different methodologies were observed for YLD calculations. To start, the disability weights may influence the accuracy of YLD estimates whenever they do not reflect the health problems that are experienced across life or at different stages [86]. Furthermore, YLD can either assume an incidence perspective or a prevalence perspective, while YLL always assume an incidence perspective (inform on years lost in the future). These YLD and YLL perspectives influence the information provided in DALY estimates. Namely, while the DALY per incident case informs on the average healthy life years a person loses in a lifetime, the DALY per prevalent case provides an overview of healthy life year(s) that a person with the disease loses per year, on average. Thus, the DALY per prevalent case (yearly estimate) can account for values higher than one as they include information on time lost in the future (YLL).

When it comes to DALY calculations, these can be heavily influenced by applying age weighting and/or time discounting. Age weighting was initially used by the GBD study after studies demonstrating a social preference to value a year lived by a young adult more highly than a year lived by a young child or an older adult [14, 87, 88]. Additionally, previous experts have argued in favor of using future time discounting to prevent the current generation from making excessive sacrifices to the point of dedicating all available resources to future health [14, 87, 88]. However, both methods were criticized, as it was argued that the DALY was defined as quantifying loss of health, rather than the social value of loss of health and that health cannot be measured with money or reinvested elsewhere [14, 87, 88]. For these reasons and to avoid extra complexity, in 2013 both methods were discontinued by the GBD [83, 89]. In the studies identified in this systematic review, the application of age weighing or time discounting was common, which is explained either by the year of publication - before 2013 - or the fact that the authors aimed to determine both health as economic burden. Nevertheless, whenever applying such methods, unweighted and undiscounted DALY estimates must also be presented in addition, to ensure comparability with other estimates/studies.

Ultimately, the heterogeneity of methodological choices observed in burden of CNCRD studies highlight the need to improve and harmonize burden of rare disease research, by developing a framework and a suitable checklist for assessing the quality of reporting of these studies. Such measures might improve the relevance of DALY estimates which can then be used for comparisons and be retrieved by policymakers and researchers.

In this study, we determined DALYs per case to facilitate comparison of findings across studies and diseases, including common diseases. According to the GBD 2019 study, DALY per case estimates for Diabetes mellitus type 2 are around 0.15 DALY per prevalent case, annually [15]. In this systematic review, however, most of the annual DALY per prevalent case estimates for rare diseases largely surpassed the DALY per prevalent diabetes case with up to 52 times higher for neural tube defects. Notably, some differences were observed for different studies that have calculated DALYs for the same rare diseases using the same method. Namely, both multiple sclerosis and hemophilia include different DALY estimates from three different initiatives. The same was observed for two studies that focused on sickle cell disease. As mentioned in previous sections, the difference in disease burden estimates between studies can be explained by differences in data sources and disease variants distribution and methods used and/or progress of knowledge of the disease, screening and new treatments. This was the case for Hemophilia, as two studies focused in both disease types A and B, while the third study focused merely on type A. Additionally, another likely explanation for hemophilia’s different estimates might be that two studies decided to apply different time discount rates (1.5% versus 3%) while another decided not to apply time discount rates. For multiple sclerosis, progress in new treatments might present a possible explanation for the different estimates obtained, as lower DALYs are observed in most recent studies.

### Strength and limitations

To the best of our knowledge, this systematic literature review is the first of its kind in assembling existing burden of CNCRD studies and identifying methodological design choices that have been used to estimate YLL, YLD, and DALY in these studies over the period from 1990 to 2022.

A limitation of our study was that it was limited to CNCDs and excluded cancer and occupational diseases. Similarly, we excluded studies which focused on diseases that did not meet Friedman’s chronicity criteria [21]. As the definition on chronicity might change per medical field, some diseases labeled as chronic to some professionals might not be present in this review. Another limitation of this study stands with the lack of harmonization of terminology of rare diseases which might have led to the omission of some rare disease names in the search strategy. At last, due to the extension of the search terms, the search was only performed in Medline and Embase, which might have led to missing studies present in different databases.

## Conclusion

In our systematic literature review a low number of burden of CNCRD studies was observed, most estimates resulted from multi-country studies, and a lack of epidemiological data and harmonization of data input sources and methodological choices was observed. These results highlight the importance of international cooperation to further CNCRD research, especially in low and middle income countries. Moreover, collaborative initiatives should structure rare diseases focus groups and develop a framework on the burden of rare disease research. Such actions might improve the visibility of the DALY concept among rare disease experts, allowing for more burden of rare disease studies. Consequently, more DALY estimates might raise awareness to the need of funding rare disease research and social support, reducing rare disease patients burden and inequalities.

## Electronic supplementary material

Below is the link to the electronic supplementary material.

**Supplementary Material 1**: The file includes further information on the search strategy (such as search terms and grey literature search procedure), the definitions of each included extracted item, a table including the result of the quality of reporting assessment of included studies with full-text available, the PRISMA checklist stating the locations of the reported items, and references to the included studies and additional sources

**Supplementary Material 2**: The excel file named “CNCRD list” contains the eligible diseases to the present study that were included in the search strategy

**Supplementary Material 3**: The excel file named “complete version of the data extraction” presents all the information extracted from the included studies

## Data Availability

Not applicable.
