# Supplementary material for: A systematic review of studies that estimated the burden of chronic non-communicable rare diseases using disability-adjusted life years
Source: Orphanet J Rare Dis. 2024 Sep 9;19:333. doi: 10.1186/s13023-024-03342-3 (PMC11384705; doi:10.1186/s13023-024-03342-3)
Supplement: Supplementary file 1 — Supplementary Material 1: The file includes further information on the search strategy (such as search terms and grey literature search procedure), the definitions of each included extracted item, a table including the result of the quality of reporting assessment of included studies with full-text available, the PRISMA checklist stating the locations of the reported items, and references to the included studies and additional sources [file 13023_2024_3342_MOESM1_ESM.docx]

Supplementary material

A systematic review of studies that estimated the burden of chronic non-communicable rare diseases using disability-adjusted life years

Contents

[Chapter 1 2](#_Toc126054741)

[Search Strategy 2](#_Toc126054742)

[Rare disease list 2](#_Toc126054743)

[Peer-review databases 2](#_Toc126054744)

[Grey literature search 136](#_Toc126054745)

[Complete version of the data extraction 139](#_Toc126054746)

[Definitions of the items included in the data extraction 140](#_Toc126054747)

[The 11th revision of the International Statistical Classification of Diseases and Related Health Problems (ICD-11) 145](#_Toc126054748)

[Chapter 2 148](#_Toc126054749)

[Quality of reporting assessment of included studies with full-text available 148](#_Toc126054750)

[Chapter 3 155](#_Toc126054751)

[The Preferred Reporting Items for Systematic Reviews and Meta-Analyses (PRISMA) checklist 155](#_Toc126054752)

[Abstract checklist 155](#_Toc126054753)

[PRISMA 2020 checklist 156](#_Toc126054754)

[Supplementary material, Definitions of the items included in the data extraction 157](#_Toc126054755)

[Chapter 4 160](#_Toc126054756)

[References 160](#_Toc126054757)

[A. Reference list of the included burden chronic non-communicable rare disease studies 160](#_Toc126054758)

[B. Additional sources 161](#_Toc126054759)

[Rare diseases list sources: search strategy 161](#_Toc126054760)

# Chapter 1

## Search Strategy

### Rare disease list

The list of chronic non-communicable rare diseases included in the search strategy is provided as an excel file format attachment, under the name “CNCRD list”. The same list was obtained through the Orphanet and the Genetic and Rare Diseases information center (GARD) websites (reference section).

### Peer-review databases

**Embase 437**

('disability-adjusted life year'/de OR 'years of life lost'/de OR (DALY OR DALYs OR ((disabil*) NEAR/4 (adjust*) NEAR/4 (life*) NEAR/4 (year*)) OR YLL OR YLLs OR ((year*) NEXT/2 (life*) NEXT/1 (lost*)) OR YLD OR YLDs OR ((year*) NEAR/3 (lived) NEAR/3 (disabil*))):ab,ti,kw) **AND** ('rare disease'/de OR 'orphan disease'/de OR 'hemophilia'/exp OR 'sarcoidosis'/de OR 'congenital heart disease'/exp OR (((rare OR orphan) NEAR/3 (diseas* OR disorder* OR syndrom* OR condition*)) OR hemophilia* OR haemophilia* OR sarcoidosis* OR besnier-boeck* OR ((congenital) NEAR/3 (heart* OR cardiac*) NEAR/3 (diseas*))):ab,ti,kw OR ((Addison NEAR/3 disease) OR (Autoimmune-gastrointestinal-dysmotility) OR (Autoimmune-hemolytic-anemia) OR (Autoimmune-hepatitis) OR (Autoimmune-Inner-Ear NEAR/3 diseas*) OR (Autoimmune-lymphoproliferative NEAR/3 syndrom*) OR (Evans NEAR/3 syndrom*) OR (Warm-antibody-hemolytic-anemia) OR (Conversion-disorder) OR (Gardner-Diamond NEAR/3 syndrom*) OR (Mietens-Weber NEAR/3 syndrom*) OR (Presenile-dementia NEAR/3 -Kraepelin) OR (15q13.3-microdeletion NEAR/3 syndrom*) OR (16p11.2-deletion NEAR/3 syndrom*) OR (17q23.1q23.2-microdeletion NEAR/3 syndrom*) OR (1q-duplication*) OR (1q21.1-microdeletion NEAR/3 syndrom*) OR (22q11.2-deletion NEAR/3 syndrom*) OR (22q11.2-duplication NEAR/3 syndrom*) OR (2q23.1-microdeletion NEAR/3 syndrom*) OR (2q37-deletion NEAR/3 syndrom*) OR (47-XXX NEAR/3 syndrom*) OR (47 NEAR/3 XYY NEAR/3 syndrom*) OR (49 NEAR/3 XXXXX NEAR/3 syndrom*) OR (Cat-eye NEAR/3 syndrom*) OR (Chromosome-1 NEAR/3 uniparental-disomy-1q12-q21) OR (Chromosome-10p-deletion) OR (Chromosome-10p-duplication) OR (Chromosome-10q-deletion) OR (Chromosome-10q-duplication) OR (Chromosome-11p-deletion) OR (Chromosome-11p-duplication) OR (Chromosome-11q-deletion) OR (Chromosome-11q-duplication) OR (Chromosome-12p-deletion) OR (Chromosome-12p-duplication) OR (Chromosome-12q-deletion) OR (Chromosome-12q-duplication) OR (Chromosome-13q-deletion) OR (Chromosome-13q-duplication) OR (Chromosome-14q-deletion) OR (Chromosome-14q-duplication) OR (Chromosome-15q-deletion) OR (Chromosome-15q-duplication) OR (Chromosome-16-trisomy) OR (Chromosome-16p-deletion) OR (Chromosome-16p-duplication) OR (Chromosome-16q-deletion) OR (Chromosome-17p-deletion) OR (Chromosome-17p-duplication) OR (Chromosome-17q-duplication) OR (Chromosome-18p-deletion) OR (Chromosome-18p-tetrasomy) OR (Chromosome-19p-deletion) OR (Chromosome-19p-duplication) OR (Chromosome-19q-deletion) OR (Chromosome-19q-duplication) OR (Chromosome-1p-deletion) OR (Chromosome-1p-duplication) OR (Chromosome-1p36-deletion NEAR/3 syndrom*) OR (Chromosome-1q-deletion) OR (Chromosome-1q21.1-duplication NEAR/3 syndrom*) OR (Chromosome-20-trisomy) OR (Chromosome-20p-deletion) OR (Chromosome-20p-duplication) OR (Chromosome-20q-deletion) OR (Chromosome-20q-duplication) OR (Chromosome-21q-deletion) OR (Chromosome-21q-duplication) OR (Chromosome-22q-deletion) OR (Chromosome-2p-deletion) OR (Chromosome-2p-duplication) OR (Chromosome-2q-deletion) OR (Chromosome-2q-duplication) OR (Chromosome-2q24-microdeletion NEAR/3 syndrom*) OR (Chromosome-3p-duplication) OR (Chromosome-3p- NEAR/3 syndrom*) OR (Chromosome-3q-deletion) OR (Chromosome-3q-duplication) OR (Chromosome-3q29-microduplication NEAR/3 syndrom*) OR (Chromosome-4p-deletion) OR (Chromosome-4p-duplication) OR (Chromosome-4q-deletion) OR (Chromosome-4q-duplication) OR (Chromosome-5p-deletion) OR (Chromosome-5p-duplication) OR (Chromosome-5q-deletion) OR (Chromosome-5q-duplication) OR (Chromosome-6p-deletion) OR (Chromosome-6p-duplication) OR (Chromosome-6q-deletion) OR (Chromosome-6q-duplication) OR (Chromosome-6q25-microdeletion NEAR/3 syndrom*) OR (Chromosome-7p-deletion) OR (Chromosome-7p-duplication) OR (Chromosome-7q-deletion) OR (Chromosome-7q-duplication) OR (Chromosome-8p-deletion) OR (Chromosome-8p-duplication) OR (Chromosome-8p23.1-deletion) OR (Chromosome-8q-deletion) OR (Chromosome-8q-duplication) OR (Chromosome-9p-deletion) OR (Chromosome-9p-duplication) OR (Chromosome-9q-deletion) OR (Chromosome-9q-duplication) OR (Chromosome-Xq-duplication) OR (Diploid-triploid-mosaicism) OR (Distal-chromosome-18q-deletion NEAR/3 syndrom*) OR (Emanuel NEAR/3 syndrom*) OR (Kleefstra NEAR/3 syndrom*) OR (Koolen-de-Vries NEAR/3 syndrom*) OR (Mosaic-monosomy-18) OR (Mosaic-monosomy-22) OR (Mosaic-trisomy-13) OR (Mosaic-trisomy-14) OR (Mosaic-trisomy-22) OR (Mosaic-trisomy-7) OR (Mosaic-trisomy-8) OR (Mosaic-trisomy-9) OR (Nablus-mask-like-facial NEAR/3 syndrom*) OR (Pallister-Killian-mosaic NEAR/3 syndrom*) OR (Partial-deletion NEAR/3 the-short-arm NEAR/3 chromosome-3) OR (Partial-deletion NEAR/3 Y) OR (Potocki-Shaffer NEAR/3 syndrom*) OR (Proximal-chromosome-18q-deletion NEAR/3 syndrom*) OR (Recombinant-chromosome-8 NEAR/3 syndrom*) OR (Ring-chromosome-1) OR (Ring-chromosome-10) OR (Ring-chromosome-11) OR (Ring-chromosome-12) OR (Ring-chromosome-13) OR (Ring-chromosome-14) OR (Ring-chromosome-15) OR (Ring-chromosome-16) OR (Ring-chromosome-17) OR (Ring-chromosome-18) OR (Ring-chromosome-19) OR (Ring-chromosome-2) OR (Ring-chromosome-20) OR (Ring-chromosome-21) OR (Ring-chromosome-22) OR (Ring-chromosome-3) OR (Ring-chromosome-4) OR (Ring-chromosome-5) OR (Ring-chromosome-6) OR (Ring-chromosome-7) OR (Ring-chromosome-8) OR (Ring-chromosome-9) OR (Smith-Magenis NEAR/3 syndrom*) OR (Tetrasomy-9p) OR (Tetrasomy-X) OR (Triploidy) OR (Trisomy-13) OR (Trisomy-17-mosaicism) OR (Trisomy-2-mosaicism) OR (Turner NEAR/3 syndrom*) OR (Wolf-Hirschhorn NEAR/3 syndrom*) OR (X-linked-susceptibility-to-autism-4) OR (Y-chromosome-infertility) OR (5q NEAR/3 syndrome*) OR (Aagenaes NEAR/3 syndrome*) OR (Abdominal-aortic-aneurysm) OR (Abetalipoprotein*) OR (Acatalas*) OR (Aceruloplasmin*) OR (Acquir* NEAR/3 agranulocytos*) OR (Acquir* NEAR/3 hemophilia) OR (Acquir* NEAR/3 hemophilia-A) OR (Acquir* NEAR/3 pure-red-cell-aplasia) OR (Acquir* NEAR/3 Von-Willebrand NEAR/3 syndrome*) OR (Adenosine-Deaminase NEAR/3 deficiency) OR (Adrenocortical-carcinoma) OR (Adult-T-cell-leukemia NEAR/3 lymphoma) OR (Afibrinogen*) OR (ALK-histiocytos*) OR (Alpha-thalass*-x-linked-intellectual-disability NEAR/3 syndrome*) OR (AML NEAR/3 myelodysplasia-related-features) OR (Anemia NEAR/3 Adenosine-triphosphatase NEAR/3 deficienc*) OR (An*-sideroblastic NEAR/3 spinocerebellar-ataxia) OR (Aneurysm NEAR/3 sinus NEAR/3 Valsalva) OR (Angioimmunoblastic-T-cell-lymphoma) OR (Angioma-hereditary-neurocutaneous) OR (Angioma-serpiginosum) OR (Antiphospholipid NEAR/3 syndrome*) OR (Aplasia-cut*-congenita-intestinal-lymphangiectasia) OR (Aplastic-an*) OR (Arterial-calcification NEAR/3 infancy) OR (Arterial-tortuosity NEAR/3 syndrome*) OR (Atransferrin*) OR (Atypical-hemolytic-uremic NEAR/3 syndrome*) OR (Autosomal NEAR/3 recessive*-protein-C NEAR/3 deficienc*) OR (Bannayan-Riley-Ruvalcaba NEAR/3 syndrome*) OR (Behcet NEAR/3 disease*) OR (Beta-thalass*) OR (Blastic-plasmacytoid-dendritic-cell) OR (Bleeding NEAR/3 disorder* NEAR/3 P2RY12-defect) OR (Bloom NEAR/3 syndrome*) OR (Blue-rubber-bleb-nevus NEAR/3 syndrome*) OR (Buerger NEAR/3 disease*) OR (Burkitt-lymphoma) OR (Campomelia-Cumming NEAR/3 type) OR (Castleman NEAR/3 disease*) OR (Chediak-Higashi NEAR/3 syndrome*) OR (Chromosome-17q11.2-deletion NEAR/3 syndrome*) OR (Chronic-myeloid-leuk*) OR (Chylous-ascites) OR (CLOVES NEAR/3 syndrome*) OR (Cobb NEAR/3 syndrome*) OR (Cold-agglutinin NEAR/3 disease*) OR (Congenital-amegakaryocytic-thrombocytopenia) OR (Congenital-analbumin*) OR (Congenital-dyserythropoietic-anemia NEAR/3 type-1) OR (Congenital-dyserythropoietic-anemia NEAR/3 type-2) OR (Congenital-dyserythropoietic-anemia NEAR/3 type-3) OR (Congenital-erythropoietic-porphyria) OR (Congenital-myasthenic NEAR/3 syndrome* NEAR/3 episodic-apnea) OR (Congenital-pulmonary-lymphangiectasia) OR (Congenital-thrombotic-thrombocytopenic-purpura) OR (Cutaneous-mastocytoma) OR (Cut*-laxa NEAR/3 autosomal* NEAR/3 recessive* NEAR/3 type-1) OR (Cut*-marmorata-telangiectatica-congenita) OR (Cyclic-neutropenia) OR (Cyclic-thrombocytopenia) OR (Cystic-medial-necrosis NEAR/3 aorta) OR (Dahlberg-Borer-Newcomer NEAR/3 syndrome*) OR (Deafness-lymphedema-leukemia NEAR/3 syndrome*) OR (Dehydrated-hereditary-stomatocytos*) OR (Diamond-Blackfan-an*) OR (Diamond-Blackfan-an*-2) OR (Diamond-Blackfan-an*-3) OR (Dysfibrinogen*) OR (Dyskeratos*-congenita) OR (Dyskeratos*-congenita NEAR/3 autosomal* NEAR/3 dominant*) OR (Dyskeratos*-congenita NEAR/3 autosomal* NEAR/3 recessive*) OR (Dyskeratos*-congenita-X-linked) OR (Ehlers-Danlos NEAR/3 syndrome*-dysfibronectinemic NEAR/3 type) OR (Eosinophilic-granulomatosis NEAR/3 polyangiit*) OR (Erythema-elevatum-diutinum) OR (Essential-thrombocyth*) OR (Extranodal-nasal-NK NEAR/3 T-cell-lymphoma) OR (Fabry NEAR/3 disease*) OR (Factor-V NEAR/3 deficienc*) OR (Factor-VII NEAR/3 deficienc*) OR (Factor-X NEAR/3 deficienc*) OR (Factor-XI NEAR/3 deficienc*) OR (Factor-XII NEAR/3 deficienc*) OR (Factor-XIII NEAR/3 deficienc*) OR (Familial-hyperthyroidism NEAR/3 mutations NEAR/3 TSH-receptor) OR (Familial-LCAT NEAR/3 deficienc*) OR (Familial-platelet NEAR/3 disorder* NEAR/3 associated-myeloid-malignancy) OR (Familial-thoracic-aortic-aneurysm NEAR/3 aortic-dissection) OR (Fanconi-an*) OR (Fetal NEAR/3 neonatal-alloimmune-thrombocytopenia) OR (Follicular-lymphoma) OR (Genuine-diffuse-phlebectasia) OR (Giant-cell-arterit*) OR (Giant-platelet NEAR/3 syndrome*) OR (Glanzmann-thrombasthenia) OR (Glucocorticoid-remediable-aldosteronism) OR (Glutamate-formiminotransferase NEAR/3 deficienc*) OR (Glycogen-storage NEAR/3 disease* NEAR/3 type-12) OR (Glycogen-storage NEAR/3 disease* NEAR/3 type-7) OR (Glycoprotein-VI NEAR/3 deficienc*) OR (Goodpasture NEAR/3 syndrome*) OR (Gorham* NEAR/3 disease*) OR (Granulomatosis NEAR/3 polyangiit*) OR (Granulomatous-slack-skin NEAR/3 disease*) OR (Gray-platelet NEAR/3 syndrome*) OR (Hairy-cell-leuk*) OR (Hashimoto-Pritzker NEAR/3 syndrome*) OR (Heinz-body-anemias) OR (Hemangioma-thrombocytopenia NEAR/3 syndrome*) OR (Hemochromatosis NEAR/3 type-2) OR (Hemochromatosis NEAR/3 type-3) OR (Hemochromatosis NEAR/3 type-4) OR (Hemoglobin-C NEAR/3 disease*) OR (Hemoglobin-E NEAR/3 disease*) OR (Hemoglobin-SC NEAR/3 disease*) OR (Hemoglobin-SE NEAR/3 disease*) OR (Hemolytic-an*-lethal-congenital-nonspherocytic NEAR/3 genital NEAR/3 other-abnormalities) OR (Hemolytic-uremic NEAR/3 syndrome*) OR (Hemophilia-A) OR (Hemophilia-B) OR (Hemorrhagic-shock NEAR/3 encephalopathy NEAR/3 syndrome*) OR (Hennekam NEAR/3 syndrome*) OR (Henoch-Schonlein-purpura) OR (Heparin-induced-thrombocytopenia) OR (Hereditary-antithrombin NEAR/3 deficienc*) OR (Hereditary-elliptocytos*) OR (Hereditary-folate-malabsorption) OR (Hereditary-hemorrhagic-telangiectasia) OR (Hereditary-hemorrhagic-telangiectasia NEAR/3 type-2) OR (Hereditary-hemorrhagic-telangiectasia NEAR/3 type-3) OR (Hereditary-hemorrhagic-telangiectasia NEAR/3 type-4) OR (Hereditary-lymphedema NEAR/3 type-II) OR (Hereditary-methemoglobin*) OR (Hereditary-paraganglioma-pheochromocytoma) OR (Hereditary-spherocytos*) OR (Hermansky-Pudlak NEAR/3 syndrome*-2) OR (High-molecular-weight-kininogen NEAR/3 deficienc*) OR (Histiocytos*-lymphadenopathy NEAR/3 syndrome*) OR (Hoyeraal-Hreidarsson NEAR/3 syndrome*) OR (Hypercoagulability NEAR/3 syndrome* NEAR/3 glycosylphosphatidylinositol NEAR/3 deficienc*) OR (Hypereosinophilic NEAR/3 syndrome*) OR (Hypersensitivity-vasculit*) OR (Hypocomplementemic-urticarial-vasculit*) OR (Hypofibrinogenemia NEAR/3 familial*) OR (Hypotrichos*-lymphedema-telangiectasia NEAR/3 syndrome*) OR (Idiopathic-thrombocytopenic-purpura) OR (Imerslund-Grasbeck NEAR/3 syndrome*) OR (Internal-carotid-agenes*) OR (Intrinsic-factor NEAR/3 deficienc*) OR (Iron-refractory-iron NEAR/3 deficienc*-an*) OR (Jacobsen NEAR/3 syndrome*) OR (Juvenile-myelomonocytic-leuk*) OR (Juvenile-temporal-arterit*) OR (Kanzaki NEAR/3 disease*) OR (Kaposi-sarcoma) OR (Kaposiform-Hemangioendothelioma) OR (Kaposiform-lymphangiomatos*) OR (Kawasaki NEAR/3 disease*) OR (Klippel-Trenaunay NEAR/3 syndrome*) OR (Langerhans-cell-sarcoma) OR (Lesch-Nyhan NEAR/3 syndrome*) OR (Liddle NEAR/3 syndrome*) OR (Lissencephaly-2) OR (Loeys-Dietz NEAR/3 syndrome*) OR (Loeys-Dietz NEAR/3 syndrome* NEAR/3 type-1) OR (Loeys-Dietz NEAR/3 syndrome* NEAR/3 type-2) OR (Loeys-Dietz NEAR/3 syndrome* NEAR/3 type-3) OR (Loeys-Dietz NEAR/3 syndrome* NEAR/3 type-4) OR (Lymphedema NEAR/3 cerebral-arteriovenous-anomaly) OR (Lymphedema-distichiasis NEAR/3 syndrome*) OR (Lymphomatoid-papulos*) OR (Maffucci NEAR/3 syndrome*) OR (Majeed NEAR/3 syndrome*) OR (Mantle-cell-lymphoma) OR (McLeod-neuroacanthocytosis NEAR/3 syndrome*) OR (Megalencephaly-capillary-malformation NEAR/3 syndrome*) OR (Megaloblastic-anemia NEAR/3 dihydrofolate-reductase NEAR/3 deficienc*) OR (Methemoglobin*-beta-globin NEAR/3 type) OR (Methylcobalamin NEAR/3 deficienc*-cbl-G NEAR/3 type) OR (Microcystic-lymphatic NEAR/3 malformation*) OR (Microscopic-polyangiit*) OR (Milroy NEAR/3 disease*) OR (Mitochondrial-myopathy NEAR/3 sideroblastic-an*) OR (MPI-CDG-CDG-Ib) OR (Multicentric-Castleman NEAR/3 disease*) OR (Multifocal-lymphangioendotheliomatosis NEAR/3 thrombocytopenia) OR (Multiple-myeloma) OR (Multisystemic-smooth-muscle-dysfunction NEAR/3 syndrome*) OR (Myelodysplastic NEAR/3 syndrome* NEAR/3 single-lineage-dysplasia) OR (Myelodysplastic NEAR/3 syndrome*s) OR (Myeloid-sarcoma) OR (MYH9-related-thrombocytopenia) OR (Neonatal-hemochromatos*) OR (Neutropenia NEAR/3 chronic* NEAR/3 familial*) OR (Neutropenia-lethal-congenital NEAR/3 eosinophilia) OR (Non-involuting-congenital-hemangioma) OR (Nonspherocytic-hemolytic-anemia NEAR/3 hexokinase NEAR/3 deficienc*) OR (Noonan NEAR/3 syndrome*) OR (Orotic-aciduria NEAR/3 type-1) OR (Overhydrated-hereditary-stomatocytos*) OR (Par*-Trousseau-thrombocytopenia) OR (Parkes-Weber NEAR/3 syndrome*) OR (Paroxysmal-cold-hemoglobinuria) OR (Paroxysmal-nocturnal-hemoglobinuria) OR (Pearson NEAR/3 syndrome*) OR (PEHO NEAR/3 syndrome*) OR (PHACE NEAR/3 syndrome*) OR (Pheochromocytoma) OR (Phosphoglycerate-kinase NEAR/3 deficienc*) OR (Plasmablastic-lymphoma) OR (Plasminogen-activator-inhibitor NEAR/3 type-1 NEAR/3 deficienc*) OR (Platelet-storage-pool NEAR/3 deficienc*) OR (Plummer-Vinson NEAR/3 syndrome*) OR (POEMS NEAR/3 syndrome*) OR (Poikiloderma NEAR/3 neutropenia) OR (Polycyth*-vera) OR (Prekallikrein NEAR/3 deficienc* NEAR/3 congenital*) OR (Primary-angiitis NEAR/3 central-nervous-system) OR (Primary-central-nervous-system-lymphoma) OR (Primary NEAR/3 familial* NEAR/3 congenital-polycyth*) OR (Primary-intestinal-lymphangiectasia) OR (Primary-myelofibros*) OR (Primary-release NEAR/3 disorder* NEAR/3 platelets) OR (Prolidase NEAR/3 deficienc*) OR (Protein-S NEAR/3 deficienc*) OR (Proteus NEAR/3 syndrome*) OR (Prothrombin NEAR/3 deficienc*) OR (Pseudo-Von-Willebrand NEAR/3 disease*) OR (Pseudohyperkal*-Cardiff) OR (Pseudoxanthoma-elasticum) OR (Pulmonary-arterio-veinous-fistula) OR (Pulmonary-atresia NEAR/3 intact-ventricular-septum) OR (Pulmonary-vein-stenos*) OR (Pyropoikilocytos*-hereditary) OR (Pyruvate-kinase NEAR/3 deficienc*) OR (Quebec-platelet NEAR/3 disorder*) OR (Red-cell-phospholipid-defect NEAR/3 hemolys*) OR (Revesz NEAR/3 syndrome*) OR (Reynolds NEAR/3 syndrome*) OR (Rh NEAR/3 deficienc* NEAR/3 syndrome*) OR (Rosai-Dorfman NEAR/3 disease*) OR (Rotor NEAR/3 syndrome*) OR (Scott NEAR/3 syndrome*) OR (Severe NEAR/3 congenital*-neutropenia NEAR/3 autosomal* NEAR/3 dominant*) OR (Severe NEAR/3 congenital*-neutropenia NEAR/3 autosomal* NEAR/3 recessive*-3) OR (Sezary NEAR/3 syndrome*) OR (Shwachman-Diamond NEAR/3 syndrome*) OR (Sickle-beta-thalass*) OR (Sickle-cell--hemoglobin-D NEAR/3 disease*) OR (Sickle-cell-an*) OR (Sideroblastic-an*-pyridoxine-refractory NEAR/3 autosomal* NEAR/3 recessive*) OR (Sideroblastic-an*-pyridoxine-responsive NEAR/3 autosomal* NEAR/3 recessive*) OR (Slow-channel NEAR/3 congenital*-myasthenic NEAR/3 syndrome*) OR (Sneddon NEAR/3 syndrome*) OR (Sturge-Weber NEAR/3 syndrome*) OR (Supraumbilical-midabdominal-raphe NEAR/3 facial-cavernous-hemangiomas) OR (Supravalvular-aortic-stenos*) OR (Susac NEAR/3 syndrome*) OR (Swyer NEAR/3 syndrome*) OR (Systemic-mastocytos*) OR (T-cell-large-granular-lymphocyte-leuk*) OR (T-cell NEAR/3 histiocyte-rich-large-B-cell-lymphoma) OR (Takayasu-arterit*) OR (TAR NEAR/3 syndrome*) OR (Thalass*) OR (Thiamine-responsive-megaloblastic-anemia NEAR/3 syndrome*) OR (Thoracolaryngopelvic-dysplasia) OR (Thrombocytopathy-asplenia-mios*) OR (Thrombocytopenia-2) OR (Thrombocytopenia NEAR/3 elevated-serum-IgA NEAR/3 renal NEAR/3 disease*) OR (Thrombomodulin-anomalies NEAR/3 familial*) OR (Thrombotic-thrombocytopenic-purpura-acquired) OR (Transient-erythroblastopenia NEAR/3 childhood) OR (Transient-myeloproliferative NEAR/3 syndrome*) OR (Triosephosphate-isomerase NEAR/3 deficienc*) OR (Tuberous-scleros*-complex) OR (Tufted-angioma) OR (Twin-to-twin-transfusion NEAR/3 syndrome*) OR (Type-1-plasminogen NEAR/3 deficienc*) OR (Unicentric-Castleman NEAR/3 disease*) OR (Vascular-Ehlers-Danlos NEAR/3 syndrome*) OR (Vein NEAR/3 Galen-aneurysm) OR (Von-Hippel-Lindau NEAR/3 disease*) OR (White-platelet NEAR/3 syndrome*) OR (Williams NEAR/3 syndrome*) OR (Wiskott-Aldrich NEAR/3 syndrome*) OR (WT-limb-blood NEAR/3 syndrome*) OR (Wyburn-Mason NEAR/3 syndrome*) OR (X-linked-sideroblastic-an*) OR (X-linked-thrombocytopenia) OR (Yellow-nail NEAR/3 syndrome*) OR (Parastremmatic-dwarfism) OR (Bilateral-parasagittal-parieto-occipital-polymicrogyria) OR (11-beta-hydroxylase NEAR/3 deficien*) OR (12q14 NEAR/3 microdeletion* NEAR/3 syndrom*) OR (15q11.2 NEAR/3 microdeletion*) OR (15q24 NEAR/3 microdeletion* NEAR/3 syndrom*) OR (16p13.11-microduplication NEAR/3 syndrom*) OR (16q24.3 NEAR/3 microdeletion* NEAR/3 syndrom*) OR (17-alpha-hydroxylase NEAR/3 deficien*) OR (17-beta-hydroxysteroid-dehydrogenase-3 NEAR/3 deficien*) OR (17q12-deletion NEAR/3 syndrom*) OR (17q12 NEAR/3 duplicat*) OR (18-Hydroxylase NEAR/3 deficien*) OR (19p13.12 NEAR/3 microdeletion* NEAR/3 syndrom*) OR (1q44 NEAR/3 microdeletion* NEAR/3 syndrom*) OR (2-methylbutyryl-CoA-dehydrogenase NEAR/3 deficien*) OR (20p12.3 NEAR/3 microdeletion* NEAR/3 syndrom*) OR (22q13.3-deletion NEAR/3 syndrom*) OR (2p15p16.1 NEAR/3 microdeletion* NEAR/3 syndrom*) OR (3-alpha-hydroxyacyl-CoA-dehydrogenase NEAR/3 deficien*) OR (3-beta-hydroxysteroid-dehydrogenase NEAR/3 deficien*) OR (3-Hydroxyisobutyric-aciduria) OR (3-methylcrotonyl-CoA-carboxylase NEAR/3 deficien*) OR (3-methylglutaconyl-CoA-hydratase NEAR/3 deficien*-) OR (AUH NEAR/3 defect*) OR (3M NEAR/3 syndrom*) OR (3MC NEAR/3 syndrom*) OR (3q29 NEAR/3 microdeletion* NEAR/3 syndrom*) OR (46 NEAR/3 XX-testicular-disorder NEAR/3 sex-development) OR (48 NEAR/3 XXXY NEAR/3 syndrom*) OR (48 NEAR/3 XYYY) OR (49 NEAR/3 XXXXY NEAR/3 syndrom*) OR (49 NEAR/3 XXXYY NEAR/3 syndrom*) OR (5-alpha-reductase NEAR/3 deficien*) OR (5-oxoprolinase NEAR/3 deficien*) OR (5q14.3 NEAR/3 microdeletion* NEAR/3 syndrom*) OR (6-pyruvoyl-tetrahydropterin-synthase NEAR/3 deficien*) OR (7q11.23 NEAR/3 duplicat* NEAR/3 syndrom*) OR (8p23.1 NEAR/3 duplicat* NEAR/3 syndrom*) OR (8q12-microduplication NEAR/3 syndrom*) OR (Aarskog NEAR/3 syndrom*) OR (Ablepharon-macrostomia NEAR/3 syndrom*) OR (ABri-amyloidosis) OR (Abruzzo-Erickson NEAR/3 syndrom*) OR (Absence NEAR/3 fingerprints-congenital-milia) OR (Absence NEAR/3 gluteal-muscle) OR (Absence NEAR/3 Tibia) OR (Absence NEAR/3 tibia NEAR/3 polydactyly) OR (Absent-breasts NEAR/3 nipples) OR (Absent-patella) OR (Acalvaria) OR (Acanthosis-nigricans-muscle-cramps-acral-enlargement) OR (Acardia) OR (Accessory-deep-peroneal-nerve) OR (Accessory-pancreas) OR (Achalasia-microcephaly NEAR/3 syndrom*) OR (Achard NEAR/3 syndrom*) OR (Acheiropody) OR (Achondrogenesis) OR (Achondrogenesis-type-1A) OR (Achondrogenesis-type-1B) OR (Achondrogenesis-type-2) OR (Achondroplasia) OR (Acitretin-embryopathy) OR (Acral-dysostosis-dyserythropoiesis NEAR/3 syndrom*) OR (Acral-peeling-skin NEAR/3 syndrom*) OR (Acro-pectoro-renal-field NEAR/3 defect*) OR (Acrocallosal NEAR/3 syndrom* NEAR/3 Schinzel) OR (Acrocapitofemoral-dysplasia) OR (Acrocephalopolydactyly) OR (Acrodermatitis-enteropathica) OR (Acrodysostosis) OR (Acrodysplasia-scoliosis) OR (Acrodysplasia NEAR/3 ossification-abnormalities NEAR/3 short-stature NEAR/3 fibular-hypoplasia) OR (Acrofacial-dysostosis-Catania) OR (Acrofacial-dysostosis-Palagonia) OR (Acrofacial-dysostosis-Rodriguez) OR (Acrofrontofacionasal-dysostosis NEAR/3 syndrom*) OR (Acrogeria NEAR/3 Gottron) OR (Acrokeratoelastoidosis NEAR/3 Costa) OR (Acromegaloid-facial-appearance NEAR/3 syndrom*) OR (Acromegaloid-features NEAR/3 overgrowth NEAR/3 cleft-palate NEAR/3 hernia) OR (Acromegaloid-hypertrichosis NEAR/3 syndrom*) OR (Acromelic-frontonasal-dysostosis) OR (Acromesomelic-dysplasia-Campailla-Martinelli) OR (Acromesomelic-dysplasia-Hunter-Thompson) OR (Acromesomelic-dysplasia-Maroteaux) OR (Acromicric-dysplasia) OR (Acroosteolysis-dominant) OR (Acropectoral NEAR/3 syndrom*) OR (Acropectorovertebral-dysplasia-F-form) OR (Acrorenal-mandibular NEAR/3 syndrom*) OR (ACTH-independent-macronodular-adrenal-hyperplasia) OR (Adactylia-unilateral) OR (Adams-Oliver NEAR/3 syndrom*) OR (ADCY5-related-dyskinesia) OR (Adenine-phosphoribosyltransferase NEAR/3 deficien*) OR (Adenosine-deaminase NEAR/3 deficien*) OR (Adenosine-monophosphate-deaminase-1 NEAR/3 deficien*) OR (Adenylosuccinase NEAR/3 deficien*) OR (Adermatoglyphia) OR (Adrenomyeloneuropathy) OR (Adrenomyodystroph*) OR (Adult-polyglucosan-body NEAR/3 diseas*) OR (ADULT NEAR/3 syndrom*) OR (Adult-onset-nemaline-myopathy) OR (Adult-onset-vitelliform-macular-dystroph*) OR (Advanced-sleep-phase NEAR/3 syndrom* NEAR/3 familial) OR (Agammaglobulinemia NEAR/3 microcephaly NEAR/3 -severe-dermatitis) OR (Agammaglobulinemia NEAR/3 non-Bruton) OR (Agenesis NEAR/3 dorsal-pancreas) OR (Agnathia-microstomia-synotia) OR (Aicardi NEAR/3 syndrom*) OR (Aicardi-Goutieres NEAR/3 syndrom*) OR (Akesson NEAR/3 syndrom*) OR (Al-Gazali-Aziz-Salem NEAR/3 syndrom*) OR (Al-Gazali-Khidr-Prem-Chandran NEAR/3 syndrom*) OR (Al-Gazali-Sabrinathan-Nair NEAR/3 syndrom*) OR (Al-Gazali NEAR/3 syndrom*) OR (Al-Gazali-Donnai-Mueller NEAR/3 syndrom*) OR (Alagille NEAR/3 syndrom*) OR (Alaninuria NEAR/3 microcephaly NEAR/3 dwarfism NEAR/3 enamel-hypoplasia NEAR/3 diabetes-mellitus) OR (Albinism) OR (Albinism-deafness NEAR/3 syndrom*) OR (Albinism-ocular-late-onset-sensorineural-deafness) OR (Albright NEAR/3 -hereditary-osteodystroph*) OR (Alexander NEAR/3 diseas*) OR (ALG1-CDG-) OR (CDG-Ik) OR (ALG11-CDG-) OR (CDG-Ip) OR (ALG12-CDG-) OR (CDG-Ig) OR (ALG13-CDG) OR (ALG2-CDG-) OR (CDG-Ii) OR (ALG3-CDG-) OR (CDG-Id) OR (ALG6-CDG-) OR (CDG-Ic) OR (ALG8-CDG-) OR (CDG-Ih) OR (ALG9-CDG-) OR (CDG-IL) OR (Alkaptonuria) OR (Allain-Babin-Demarquez NEAR/3 syndrom*) OR (Allan-Herndon-Dudley NEAR/3 syndrom*) OR (Alopecia-epilepsy-oligophrenia NEAR/3 syndrom* NEAR/3 Moynahan) OR (Alopecia-intellectual-disability NEAR/3 syndrom*-2) OR (Alopecia-totalis) OR (Alopecia-universalis) OR (Alopecia-universalis-onychodystroph*-vitiligo) OR (Alopecia NEAR/3 epilepsy NEAR/3 pyorrhea NEAR/3 mental-subnormality) OR (Alopecia-contractures-dwarfism-intellectual-disability NEAR/3 syndrom*) OR (Alopecia-intellectual-disability NEAR/3 syndrom*) OR (Alpers NEAR/3 syndrom*) OR (Alpha-1-antitrypsin NEAR/3 deficien*) OR (Alpha-ketoglutarate-dehydrogenase NEAR/3 deficien*) OR (Alpha-mannosidosis) OR (Alport NEAR/3 syndrom*) OR (Alstrom NEAR/3 syndrom*) OR (Alternating-hemiplegia NEAR/3 childhood) OR (Alveolar-capillary-dysplasia) OR (Amaurosis-congenita-cone-rod-type NEAR/3 congenital-hypertrichosis) OR (Ambras NEAR/3 syndrom*) OR (Amelogenesis-imperfecta) OR (Amelogenesis-imperfecta-hypoplastic NEAR/3 hypomaturation-X-linked-1) OR (Amelogenesis-imperfecta-local-hypoplastic) OR (Amelogenesis-imperfecta-nephrocalcinosis) OR (Ameloonychohypohidrotic NEAR/3 syndrom*) OR (Amino-aciduria NEAR/3 mental NEAR/3 deficien* NEAR/3 dwarfism NEAR/3 muscular-dystroph* NEAR/3 osteoporosis NEAR/3 acidosis) OR (Aminoacylase-1 NEAR/3 deficien*) OR (Aminolevulinate-dehydratase NEAR/3 deficien*-porphyria) OR (Amish-lethal-microcephaly) OR (Amish-Nemaline-Myopathy) OR (Amniotic-band NEAR/3 syndrom*) OR (Amyloidosis-corneal) OR (Amyloidosis NEAR/3 gingiva NEAR/3 conjunctiva NEAR/3 intellectual-disability) OR (Amyotonia-congenita) OR (Anal-sphincter-dysplasia) OR (Anauxetic-dysplasia) OR (Andermann NEAR/3 syndrom*) OR (Andersen-Tawil NEAR/3 syndrom*) OR (Anencephaly) OR (Angel-shaped-phalangoepiphyseal-dysplasia) OR (Angelman NEAR/3 syndrom*) OR (Aniridia---ptosis---intellectual-disability---familial-obesity) OR (Aniridia-absent-patella) OR (Aniridia-renal-agenesis-psychomotor-retardation) OR (Ankyloblepharon-filiforme-adnatum-cleft-palate) OR (Ankyloblepharon-filiforme-imperforate-anus) OR (Ankyloblepharon-ectodermal NEAR/3 defect*s-cleft-lip NEAR/3 palate NEAR/3 syndrom*) OR (Ankylosing-vertebral-hyperostosis NEAR/3 tylosis) OR (Ankylosis NEAR/3 teeth) OR (Annular-pancreas) OR (Anodontia) OR (Anomalous-origin NEAR/3 right-pulmonary-artery-familial) OR (Anonychia-ectrodactyly) OR (Anonychia-onychodystroph* NEAR/3 brachydactyly-type-B NEAR/3 ectrodactyly) OR (Anonychia-onychodystroph* NEAR/3 hypoplasia-) OR (Anonychia-onychodystroph*-absence NEAR/3 distal-phalanges) OR (Anophthalmia-plus NEAR/3 syndrom*) OR (Anophthalmos NEAR/3 limb-anomalies) OR (Anorchia) OR (Antecubital-pterygium) OR (Anterior-segment-dysgenesis) OR (Antley-Bixler NEAR/3 syndrom*) OR (Aortic-arch-anomaly---peculiar-facies---intellectual-disability) OR (Aortic-coarctation) OR (Aortopulmonary-window) OR (Apert NEAR/3 syndrom*) OR (Aphalangia-partial NEAR/3 syndactyly NEAR/3 duplication NEAR/3 metatarsal-IV) OR (Aplasia-cutis-congenita) OR (Aplasia-cutis-congenita NEAR/3 limbs-recessive) OR (Arachnodactyly---intellectual-disability---dysmorphism) OR (Arachnoid-cysts) OR (AREDYLD) OR (Arginase NEAR/3 deficien*) OR (Argininosuccinic-aciduria) OR (Arhinia-choanal-atresia-microphthalmia) OR (Aromatase NEAR/3 deficien*) OR (Aromatase-excess NEAR/3 syndrom*) OR (Aromatic-L-amino-acid-decarboxylase NEAR/3 deficien*) OR (Arrhinia) OR (Arthrochalasia-Ehlers-Danlos NEAR/3 syndrom*) OR (Arthrogryposis NEAR/3 ectodermal-dysplasia) OR (Arthrogryposis-epileptic-seizures-migrational-brain-disorder) OR (Arthrogryposis-multiplex-congenita-neurogenic) OR (Arthrogryposis-multiplex-congenita-whistling-face) OR (Arthrogryposis-multiplex-congenita NEAR/3 distal NEAR/3 X-linked) OR (Arthrogryposis-renal-dysfunction-cholestasis NEAR/3 syndrom*) OR (Arthrogryposis NEAR/3 ectodermal-dysplasia NEAR/3 cleft NEAR/3 -developmental-delay) OR (Arthrogryposis-like-hand-anomaly NEAR/3 sensorineural-deafness) OR (Arts NEAR/3 syndrom*) OR (Ascher NEAR/3 syndrom*) OR (Aspartylglycosaminuria) OR (Asternia) OR (Ataxia---hypogonadism---choroidal-dystroph*) OR (Ataxia-telangiectasia) OR (Ataxia NEAR/3 oculomotor-apraxia-type-1) OR (Ataxia NEAR/3 Oculomotor-Apraxia-Type-2) OR (Ataxia NEAR/3 oculomotor-apraxia-type-4) OR (Ataxia NEAR/3 vitamin-E NEAR/3 deficien*) OR (Atelosteogenesis-type-1) OR (Atelosteogenesis-type-2) OR (Atelosteogenesis-type-3) OR (Atkin NEAR/3 syndrom*) OR (Atresia NEAR/3 small-intestine) OR (Atrial-myxoma NEAR/3 familial) OR (Atrial-septal NEAR/3 defect*-coronary-sinus) OR (Atrial-septal NEAR/3 defect*-ostium-primum) OR (Atrial-septal NEAR/3 defect*-sinus-venosus) OR (Atypical-Gaucher NEAR/3 diseas*-due-to-saposin-C NEAR/3 deficien*) OR (Atypical-Rett NEAR/3 syndrom*) OR (Atypical-Werner NEAR/3 syndrom*) OR (Auralcephalosyndactyly) OR (Auriculo-condylar NEAR/3 syndrom*) OR (Auriculoosteodysplasia) OR (Ausems-Wittebol-Post-Hennekam NEAR/3 syndrom*) OR (Autism NEAR/3 port-wine-stain) OR (Autoimmune-lymphoproliferative NEAR/3 syndrom*-due-to-CTLA4-haploinsuffiency) OR (Autoimmune-polyglandular NEAR/3 syndrom*-type-1) OR (Autoimmune-polyglandular NEAR/3 syndrom*-type-2) OR (Autosomal-dominant-Alport NEAR/3 syndrom*) OR (Autosomal-dominant-centronuclear-myopathy) OR (Autosomal-dominant-cerebellar-ataxia NEAR/3 deafness NEAR/3 -narcolepsy) OR (Autosomal-dominant-Charcot-Marie-Tooth NEAR/3 diseas*-type-2 NEAR/3 giant-axons) OR (Autosomal-dominant-deafness-onychodystroph* NEAR/3 syndrom*) OR (Autosomal-dominant-distal-renal-tubular-acidosis) OR (Autosomal-dominant-hyper-IgE NEAR/3 syndrom*) OR (Autosomal-dominant-intermediate-Charcot-Marie-Tooth NEAR/3 diseas*-type-A) OR (Autosomal-dominant-intermediate-Charcot-Marie-Tooth NEAR/3 diseas*-type-B) OR (Autosomal-dominant-intermediate-Charcot-Marie-Tooth NEAR/3 diseas*-type-C) OR (Autosomal-dominant-intermediate-Charcot-Marie-Tooth NEAR/3 diseas*-type-D) OR (Autosomal-dominant-intermediate-Charcot-Marie-Tooth NEAR/3 diseas*-type-E) OR (Autosomal-dominant-intermediate-Charcot-Marie-Tooth NEAR/3 diseas*-type-F) OR (Autosomal-dominant-leukodystroph* NEAR/3 autonomic NEAR/3 diseas*) OR (Autosomal-dominant-multiple-pterygium NEAR/3 syndrom*) OR (Autosomal-dominant-neuronal-ceroid-lipofuscinosis-4B) OR (Autosomal-dominant-nocturnal-frontal-lobe-epilepsy) OR (Autosomal-dominant-non-syndromic-intellectual-disability) OR (Autosomal-dominant-optic-atrophy NEAR/3 cataract) OR (Autosomal-dominant-optic-atrophy-plus NEAR/3 syndrom*) OR (Autosomal-dominant-palmoplantar-keratoderma NEAR/3 congenital-alopecia) OR (Autosomal-dominant-partial-epilepsy NEAR/3 auditory-features) OR (Autosomal-dominant-pseudohypoaldosteronism-type-1) OR (Autosomal-dominant-spinal-muscular-atrophy NEAR/3 lower-extremity-predominant-1) OR (Autosomal-dominant-spondyloepiphyseal-dysplasia-tarda) OR (Autosomal-dominant-tubulointerstitial-kidney NEAR/3 diseas*-due-to-REN-mutations) OR (Autosomal-dominant-tubulointerstitial-kidney NEAR/3 diseas*-due-to-UMOD-mutations) OR (Autosomal-dominant-vitreoretinochoroidopathy) OR (Autosomal-erythropoietic-protoporphyria) OR (Autosomal-recessive-Alport NEAR/3 syndrom*) OR (Autosomal-recessive-axonal-neuropathy NEAR/3 neuromyotonia) OR (Autosomal-recessive-candidiasis-familial-chronic-mucocutaneous) OR (Autosomal-recessive-centronuclear-myopathy) OR (Autosomal-recessive-Charcot-Marie-Tooth NEAR/3 diseas* NEAR/3 hoarseness) OR (Autosomal-recessive-distal-osteolysis NEAR/3 syndrom*) OR (Autosomal-recessive-early-onset-inflammatory-bowel NEAR/3 diseas*) OR (Autosomal-recessive-intermediate-Charcot-Marie-Tooth NEAR/3 diseas*-type-A) OR (Autosomal-recessive-intermediate-Charcot-Marie-Tooth NEAR/3 diseas*-type-B) OR (Autosomal-recessive-neuronal-ceroid-lipofuscinosis-4A) OR (Autosomal-recessive-palmoplantar-keratoderma NEAR/3 congenital-alopecia) OR (Autosomal-recessive-polycystic-kidney NEAR/3 diseas*) OR (Autosomal-recessive-primary-microcephaly) OR (Autosomal-recessive-pseudohypoaldosteronism-type-1) OR (Autosomal-recessive-spastic-ataxia-4) OR (Autosomal-recessive-spastic-paraplegia-type-49) OR (Autosomal-recessive-spinocerebellar-ataxia-9) OR (Axenfeld-Rieger NEAR/3 syndrom*) OR (Axial-mesodermal-dysplasia-spectrum) OR (Axial-spondylometaphyseal-dysplasia) OR (Ayazi NEAR/3 syndrom*) OR (B4GALT1-CDG-) OR (CDG-IId) OR (Baetz-Greenwalt NEAR/3 syndrom*) OR (Bagatelle-Cassidy NEAR/3 syndrom*) OR (Baller-Gerold NEAR/3 syndrom*) OR (Bamforth NEAR/3 syndrom*) OR (Bangstad NEAR/3 syndrom*) OR (Banki NEAR/3 syndrom*) OR (Bantu-siderosis) OR (BAP1-tumor-predisposition NEAR/3 syndrom*) OR (Baraitser-Winter NEAR/3 syndrom*) OR (Barakat NEAR/3 syndrom*) OR (Barber-Say NEAR/3 syndrom*) OR (Bardet-Biedl NEAR/3 syndrom*) OR (Bardet-Biedl NEAR/3 syndrom*-1) OR (Bardet-Biedl NEAR/3 syndrom*-10) OR (Bardet-Biedl NEAR/3 syndrom*-11) OR (Bardet-Biedl NEAR/3 syndrom*-12) OR (Bardet-Biedl NEAR/3 syndrom*-2) OR (Bardet-Biedl NEAR/3 syndrom*-3) OR (Bardet-Biedl NEAR/3 syndrom*-4) OR (Bare-lymphocyte NEAR/3 syndrom*-2) OR (Barraquer-Simons NEAR/3 syndrom*) OR (Barth NEAR/3 syndrom*) OR (Bartter NEAR/3 syndrom*-type-3) OR (Bartter NEAR/3 syndrom*-type-4) OR (Battaglia-Neri NEAR/3 syndrom*) OR (Bazex-Dupre-Christol NEAR/3 syndrom*) OR (Beare-Stevenson-cutis-gyrata NEAR/3 syndrom*) OR (Becker-muscular-dystroph*) OR (Becker-nevus NEAR/3 syndrom*) OR (Beckwith-Wiedemann NEAR/3 syndrom*) OR (Beemer-Ertbruggen NEAR/3 syndrom*) OR (Behr NEAR/3 syndrom*) OR (Benallegue-Lacete NEAR/3 syndrom*) OR (Benign-essential-blepharospasm) OR (Benign-familial-neonatal-epilepsy) OR (Benign-familial-neonatal-infantile-seizures) OR (Benign-hereditary-chorea) OR (Berk-Tabatznik NEAR/3 syndrom*) OR (Best-vitelliform-macular-dystroph*) OR (Beta-ketothiolase NEAR/3 deficien*) OR (Beta-Propeller-Protein-Associated-Neurodegeneration) OR (Bethlem-myopathy) OR (Beukes-familial-hip-dysplasia) OR (Biemond NEAR/3 syndrom*) OR (Biemond NEAR/3 syndrom*-2) OR (Bietti-crystalline-corneoretinal-dystroph*) OR (Bifid-nose) OR (Bifid-nose NEAR/3 anorectal NEAR/3 renal-anomalies) OR (Bilateral-frontal-polymicrogyria) OR (Bilateral-frontoparietal-polymicrogyria) OR (Bilateral-generalized-polymicrogyria) OR (Bilateral-perisylvian-polymicrogyria) OR (Bile-acid-synthesis NEAR/3 defect* NEAR/3 congenital NEAR/3 4) OR (Biliary-atresia) OR (Biotin-thiamine-responsive-basal-ganglia NEAR/3 diseas*) OR (Biotinidase NEAR/3 deficien*) OR (Birk-Barel NEAR/3 syndrom*) OR (Birt-Hogg-Dube NEAR/3 syndrom*) OR (Bixler-Christian-Gorlin NEAR/3 syndrom*) OR (Bjornstad NEAR/3 syndrom*) OR (Blau NEAR/3 syndrom*) OR (Blepharo-cheilo-odontic NEAR/3 syndrom*) OR (Blepharonasofacial-malformation NEAR/3 syndrom*) OR (Blepharophimosis NEAR/3 ptosis NEAR/3 syndactyly NEAR/3 -short-stature) OR (Blepharoptosis-myopia-ectopia-lentis) OR (Blount NEAR/3 diseas*) OR (Blue-cone-monochromatism) OR (Blue-diaper NEAR/3 syndrom*) OR (BOD NEAR/3 syndrom*) OR (Bohring-Opitz NEAR/3 syndrom*) OR (Bone-dysplasia-Azouz) OR (Bone-dysplasia-lethal-Holmgren) OR (Book NEAR/3 syndrom*) OR (Boomerang-dysplasia) OR (BOR-Duane-hydrocephalus-contiguous-gene NEAR/3 syndrom*) OR (Borjeson-Forssman-Lehmann NEAR/3 syndrom*) OR (Bork-Stender-Schmidt NEAR/3 syndrom*) OR (Bowen-Conradi NEAR/3 syndrom*) OR (Bowing NEAR/3 legs NEAR/3 anterior NEAR/3 dwarfism) OR (Boylan-Dew-Greco NEAR/3 syndrom*) OR (Brachioskeletogenital NEAR/3 syndrom*) OR (Brachycephalofrontonasal-dysplasia) OR (Brachydactylous-dwarfism-Mseleni) OR (Brachydactyly-elbow-wrist-dysplasia) OR (Brachydactyly-long-thumb) OR (Brachydactyly-Mononen) OR (Brachydactyly-preaxial NEAR/3 hallux-varus NEAR/3 thumb-abduction) OR (Brachydactyly-tibial-hypoplasia) OR (Brachydactyly-type-A1) OR (Brachydactyly-type-A2) OR (Brachydactyly-type-A3) OR (Brachydactyly-type-A4) OR (Brachydactyly-type-A5) OR (Brachydactyly-type-A6) OR (Brachydactyly-type-A7) OR (Brachydactyly-type-B) OR (Brachydactyly-type-C) OR (Brachydactyly-type-E) OR (Brachydactyly-types-B NEAR/3 E-combined) OR (Brachydactyly NEAR/3 hypertension) OR (Brachydactyly-mesomelia-intellectual-disability-heart NEAR/3 defect*s NEAR/3 syndrom*) OR (Brachyolmia-type-3) OR (Brachyphalangy NEAR/3 polydactyly NEAR/3 -tibial-aplasia) OR (Brachyphalangy NEAR/3 polydactyly NEAR/3 -tibial-hypoplasia) OR (Bradyopsia) OR (Brain-dopamine-serotonin-vesicular-transport NEAR/3 diseas*) OR (Brain-lung-thyroid NEAR/3 syndrom*) OR (Branchial-arch NEAR/3 syndrom*-X-linked) OR (Branchiooculofacial NEAR/3 syndrom*) OR (Branchiootic NEAR/3 syndrom*) OR (Branchiootorenal NEAR/3 syndrom*) OR (BRCA1-hereditary-breast NEAR/3 ovarian-cancer NEAR/3 syndrom*) OR (BRCA2-hereditary-breast NEAR/3 ovarian-cancer NEAR/3 syndrom*) OR (Brittle-cornea NEAR/3 syndrom*) OR (Brody-myopathy) OR (Bronchogenic-cyst) OR (Bronchopulmonary-dysplasia) OR (Brooks-Wisniewski-Brown NEAR/3 syndrom*) OR (Bruck NEAR/3 syndrom*-1) OR (Bruck NEAR/3 syndrom*-2) OR (Brugada NEAR/3 syndrom*) OR (Bullous-dystroph*-hereditary-macular) OR (Buschke-Ollendorff NEAR/3 syndrom*) OR (C NEAR/3 syndrom*) OR (C1q NEAR/3 deficien*) OR (Cabezas NEAR/3 syndrom*) OR (CAD-CDG) OR (CADASIL) OR (Caffey NEAR/3 diseas*) OR (Calabro NEAR/3 syndrom*) OR (Calloso-genital-dysplasia) OR (Camera-Marugo-Cohen NEAR/3 syndrom*) OR (Campomelic-dysplasia) OR (Camptobrachydactyly) OR (Camptodactyly-arthropathy-coxa-vara-pericarditis NEAR/3 syndrom*) OR (Camptodactyly NEAR/3 syndrom*-Guadalajara-type-1) OR (Camptodactyly NEAR/3 syndrom*-Guadalajara-type-2) OR (Camptodactyly NEAR/3 syndrom*-Guadalajara-type-3) OR (Camptodactyly NEAR/3 fibrous-tissue-hyperplasia NEAR/3 -skeletal-dysplasia) OR (Camptodactyly NEAR/3 tall-stature NEAR/3 -hearing-loss NEAR/3 syndrom*) OR (Camptodactyly-ichthyosis NEAR/3 syndrom*) OR (Camptomelic NEAR/3 syndrom*-long-limb) OR (Camurati-Engelmann NEAR/3 diseas* NEAR/3 type-2) OR (Camurati-Engelmann NEAR/3 diseas*) OR (Canavan NEAR/3 diseas*) OR (Cantu-Sanchez-Corona-Fragoso NEAR/3 syndrom*) OR (Cantu NEAR/3 syndrom*) OR (Cap-myopathy) OR (Carbamoyl-phosphate-synthetase-1 NEAR/3 deficien*) OR (Carbonic-anhydrase-VA NEAR/3 deficien*) OR (Cardiac-Valvular-Ehlers-Danlos NEAR/3 syndrom*) OR (Cardioauditory NEAR/3 syndrom* NEAR/3 Sanchez-Cascos) OR (Cardioencephalomyopathy) OR (Cardiofaciocutaneous NEAR/3 syndrom*) OR (Cardiomelic NEAR/3 syndrom*-Stratton-Koehler) OR (Cardiomyopathy NEAR/3 deafness-due-to-tRNA-lysine-gene-mutation) OR (Cardiomyopathy-cataract-hip-spine NEAR/3 diseas*) OR (Cardiomyopathy-dilated NEAR/3 woolly-hair NEAR/3 keratoderma) OR (Cardioskeletal NEAR/3 syndrom*-Kuwaiti) OR (Carey-Fineman-Ziter NEAR/3 syndrom*) OR (Carney-complex) OR (Carney-triad) OR (Carnitine-palmitoyl-transferase-1A NEAR/3 deficien*) OR (Carnitine-acylcarnitine-translocase NEAR/3 deficien*) OR (Carnosinemia) OR (Caroli NEAR/3 diseas*) OR (Carpenter NEAR/3 syndrom*) OR (Carpotarsal-osteochondromatosis) OR (Cartilage-hair-hypoplasia) OR (Cataract-ataxia-deafness) OR (Cataract-congenital-Volkmann) OR (Cataract-microcornea NEAR/3 syndrom*) OR (Cataract NEAR/3 total-congenital) OR (Catatrichy) OR (Catecholaminergic-polymorphic-ventricular-tachycardia) OR (Catel-Manzke NEAR/3 syndrom*) OR (Caudal-appendage-deafness) OR (Caudal-regression-sequence) OR (Central-core NEAR/3 diseas*) OR (Cerebellar-ataxia NEAR/3 hypogonadotropic-hypogonadism) OR (Cerebellar-ataxia-ectodermal-dysplasia) OR (Cerebellar-ataxia NEAR/3 areflexia NEAR/3 pes-cavus NEAR/3 optic-atrophy NEAR/3 sensorinural-hearing-loss) OR (Cerebellar-hypoplasia) OR (Cerebellar-hypoplasia-tapetoretinal-degeneration) OR (Cerebellar-hypoplasia NEAR/3 endosteal-sclerosis) OR (Cerebelloparenchymal-disorder-3) OR (Cerebellum-agenesis-hydrocephaly) OR (Cerebral-autosomal-recessive-arteriopathy NEAR/3 subcortical-infarcts NEAR/3 leukoencephalopathy) OR (Cerebral-dysgenesis NEAR/3 neuropathy NEAR/3 ichthyosis NEAR/3 -palmoplantar-keratoderma NEAR/3 syndrom*) OR (Cerebral-folate NEAR/3 deficien*) OR (Cerebral-gigantism-jaw-cysts) OR (Cerebro-costo-mandibular NEAR/3 syndrom*) OR (Cerebro-facio-articular NEAR/3 syndrom*) OR (Cerebro-oculo-facio-skeletal NEAR/3 syndrom*) OR (Cerebrocostomandibular-like NEAR/3 syndrom*) OR (Cerebrooculonasal NEAR/3 syndrom*) OR (Cerebrotendinous-xanthomatosis) OR (Ceroid-lipofuscinosis-neuronal-1) OR (Cerulean-cataract) OR (Cervical-hypertrichosis-peripheral-neuropathy) OR (Chanarin-Dorfman NEAR/3 syndrom*) OR (Char NEAR/3 syndrom*) OR (Charcot-Marie-Tooth NEAR/3 diseas*-type-1A) OR (Charcot-Marie-Tooth NEAR/3 diseas*-type-1B) OR (Charcot-Marie-Tooth NEAR/3 diseas*-type-1C) OR (Charcot-Marie-Tooth NEAR/3 diseas*-type-1D) OR (Charcot-Marie-Tooth NEAR/3 diseas*-type-1E) OR (Charcot-Marie-Tooth NEAR/3 diseas*-type-1F) OR (Charcot-Marie-Tooth NEAR/3 diseas*-type-2B) OR (Charcot-Marie-Tooth NEAR/3 diseas*-type-2B1) OR (Charcot-Marie-Tooth NEAR/3 diseas*-type-2B2) OR (Charcot-Marie-Tooth NEAR/3 diseas*-type-2D) OR (Charcot-Marie-Tooth NEAR/3 diseas*-type-2E) OR (Charcot-Marie-Tooth NEAR/3 diseas*-type-2F) OR (Charcot-Marie-Tooth NEAR/3 diseas*-type-2G) OR (Charcot-Marie-Tooth NEAR/3 diseas*-type-2H) OR (Charcot-Marie-Tooth NEAR/3 diseas*-type-2I) OR (Charcot-Marie-Tooth NEAR/3 diseas*-type-2J) OR (Charcot-Marie-Tooth NEAR/3 diseas*-type-2K) OR (Charcot-Marie-Tooth NEAR/3 diseas*-type-2N) OR (Charcot-Marie-Tooth NEAR/3 diseas*-type-2O) OR (Charcot-Marie-Tooth NEAR/3 diseas*-type-2P) OR (Charcot-Marie-Tooth NEAR/3 diseas*-type-2Q) OR (Charcot-Marie-Tooth NEAR/3 diseas*-type-2R) OR (Charcot-Marie-Tooth NEAR/3 diseas*-type-4A) OR (Charcot-Marie-Tooth NEAR/3 diseas*-type-4B1) OR (Charcot-Marie-Tooth NEAR/3 diseas*-type-4B2) OR (Charcot-Marie-Tooth NEAR/3 diseas*-type-4C) OR (Charcot-Marie-Tooth NEAR/3 diseas*-type-4D) OR (Charcot-Marie-Tooth NEAR/3 diseas*-type-4E) OR (Charcot-Marie-Tooth NEAR/3 diseas*-type-4H) OR (CHARGE NEAR/3 syndrom*) OR (Charlie-M NEAR/3 syndrom*) OR (Cherubism) OR (Chiari-malformation-type-2) OR (Chiari-malformation-type-3) OR (CHILD NEAR/3 syndrom*) OR (Childhood-apraxia NEAR/3 speech) OR (Childhood-encephalopathy-due-to-thiamine-pyrophosphokinase NEAR/3 deficien*) OR (Childhood-hypophosphatasia) OR (Childhood-onset-nemaline-myopathy) OR (Chitayat-Meunier-Hodgkinson NEAR/3 syndrom*) OR (Choanal-atresia-hearing-loss-cardiac NEAR/3 defect*s-craniofacial-dysmorphism NEAR/3 syndrom*) OR (Cholesteryl-ester-storage NEAR/3 diseas*) OR (Chondrocalcinosis-2) OR (Chondrodysplasia-acromesomelic NEAR/3 genital-anomalies) OR (Chondrodysplasia-Blomstrand) OR (Chondrodysplasia-calcificans-metaphysealis) OR (Chondrodysplasia-punctata-1 NEAR/3 X-linked-recessive) OR (Chondrodysplasia-punctata-Sheffield) OR (Chondrodysplasia NEAR/3 joint-dislocations NEAR/3 GPAPP) OR (Chondrodysplasia NEAR/3 Grebe) OR (Chordoma) OR (Chorea-acanthocytosis) OR (Choroidal-dystroph*-central-areolar) OR (Choroideremia) OR (Christianson NEAR/3 syndrom*) OR (Chromosome-15 NEAR/3 trisomy-mosaicism) OR (Chromosome-16p13.3-deletion NEAR/3 syndrom*) OR (Chromosome-16p13.3 NEAR/3 duplicat*) OR (Chromosome-17p13.1-deletion NEAR/3 syndrom*) OR (Chromosome-17q-deletion) OR (Chromosome-18p NEAR/3 duplicat*) OR (Chromosome-19q13.11-deletion NEAR/3 syndrom*) OR (Chromosome-1q41-q42-deletion NEAR/3 syndrom*) OR (Chromosome-21 NEAR/3 uniparental-disomy) OR (Chronic-atypical-neutrophilic-dermatosis NEAR/3 lipodystroph* NEAR/3 elevated-temperature) OR (Chronic-granulomatous NEAR/3 diseas*) OR (Chronic-progressive-external-ophthalmoplegia) OR (Chudley-Rozdilsky NEAR/3 syndrom*) OR (Chylomicron-retention NEAR/3 diseas*) OR (Chylothorax NEAR/3 congenital) OR (Circumferential-skin-creases-Kunze) OR (Citrullinemia-type-II) OR (Clark-Baraitser NEAR/3 syndrom*) OR (Clasped-thumbs NEAR/3 congenital) OR (Classical-like-Ehlers-Danlos NEAR/3 syndrom*) OR (Cleft-hand-absent-tibia) OR (Cleft-palate-short-stature-vertebral-anomalies) OR (Cleft-palate NEAR/3 midfacial-hypoplasia NEAR/3 triangular-facies NEAR/3 -sensorineural-hearing-loss) OR (Cleidocranial-dysplasia) OR (Cleidocranial-dysplasia-recessive-form) OR (Cleidorhizomelic NEAR/3 syndrom*) OR (Clouston NEAR/3 syndrom*) OR (COACH NEAR/3 syndrom*) OR (COASY-Protein-Associated-Neurodegeneration) OR (Coats NEAR/3 diseas*) OR (Cockayne NEAR/3 syndrom*) OR (Cockayne NEAR/3 syndrom*-type-I) OR (Cockayne NEAR/3 syndrom*-type-II) OR (Cockayne NEAR/3 syndrom*-type-III) OR (CODAS NEAR/3 syndrom*) OR (Coffin-Lowry NEAR/3 syndrom*) OR (Coffin-Siris NEAR/3 syndrom*) OR (COG1-CDG-) OR (CDG-IIg) OR (COG4-CDG-) OR (CDG-IIj) OR (COG5-CDG-) OR (CDG-IIi) OR (COG7-CDG-) OR (CDG-IIe) OR (COG8-CDG-) OR (CDG-IIh) OR (Cogan-Reese NEAR/3 syndrom*) OR (Cohen NEAR/3 syndrom*) OR (Cold-induced-sweating NEAR/3 syndrom*) OR (Cole-Carpenter NEAR/3 syndrom*) OR (Collins-Pope NEAR/3 syndrom*) OR (Coloboma NEAR/3 alar-nasal-cartilages NEAR/3 telecanthus) OR (Coloboma NEAR/3 macula) OR (Coloboma NEAR/3 macula NEAR/3 type-B-brachydactyly) OR (Colpocephaly) OR (Combined-immunodeficiency NEAR/3 skin-granulomas) OR (Combined-oxidative-phosphorylation NEAR/3 deficien*-16) OR (Combined-pituitary-hormone-deficiencies NEAR/3 genetic-forms) OR (Common-variable-immunodeficiency) OR (Complement-component-2 NEAR/3 deficien*) OR (Complete-androgen-insensitivity NEAR/3 syndrom*) OR (Condensing-osteitis NEAR/3 clavicle) OR (Conductive-deafness NEAR/3 malformed-external-ear) OR (Cone-dystroph*) OR (Cone-rod-dystroph*) OR (Cone-rod-dystroph*-3) OR (Cone-rod-dystroph*-5) OR (Cone-rod-dystroph*-6) OR (Cone-rod-dystroph*-amelogenesis-imperfecta) OR (Cone-rod-dystroph*-X-linked-1) OR (Cone-rod-dystroph*-X-linked-2) OR (Cone-rod-dystroph*-X-linked-3) OR (Congenital-absence NEAR/3 sternocleidomastoid-muscle) OR (Congenital-adrenal-hyperplasia-due-to-cytochrome-P450-oxidoreductase NEAR/3 deficien*) OR (Congenital-anosmia) OR (Congenital-bilateral-absence NEAR/3 vas-deferens) OR (Congenital-bile-acid-synthesis NEAR/3 defect* NEAR/3 type-1) OR (Congenital-bile-acid-synthesis NEAR/3 defect* NEAR/3 type-2) OR (Congenital-central-hypoventilation NEAR/3 syndrom*) OR (Congenital-chloride-diarrhea) OR (Congenital-contractural-arachnodactyly) OR (Congenital-cytomegalovirus) OR (Congenital-deafness NEAR/3 vitiligo NEAR/3 achalasia) OR (Congenital-diaphragmatic-hernia) OR (Congenital-ectodermal-dysplasia NEAR/3 hearing-loss) OR (Congenital-extrahepatic-portosystemic-shunt) OR (Congenital-femoral NEAR/3 deficien*) OR (Congenital-fiber-type-disproportion) OR (Congenital-fibrosis NEAR/3 extraocular-muscles) OR (Congenital-generalized-lipodystroph*) OR (Congenital-generalized-lipodystroph*-type-2) OR (Congenital-generalized-lipodystroph*-type-4) OR (Congenital-heart-block) OR (Congenital-hydrocephalus) OR (Congenital-hyperinsulinism) OR (Congenital-insensitivity-to-pain) OR (Congenital-insensitivity-to-pain NEAR/3 anhidrosis) OR (Congenital-lactase NEAR/3 deficien*) OR (Congenital-laryngeal-palsy) OR (Congenital-lipoid-adrenal-hyperplasia) OR (Congenital-lobar-emphysema) OR (Congenital-microcoria) OR (Congenital-mirror-movement-disorder) OR (Congenital-muscular-dystroph*-due-to-LMNA-mutation) OR (Congenital-muscular-dystroph*-type-1A) OR (Congenital-muscular-dystroph* NEAR/3 integrin-alpha-7 NEAR/3 deficien*) OR (Congenital-muscular-dystroph*-dystroglycanopathy NEAR/3 intellectual-disability-) OR (Congenital-myasthenic NEAR/3 syndrom*-associated NEAR/3 acetylcholine-receptor NEAR/3 deficien*) OR (Congenital-nephrotic NEAR/3 syndrom*-Finnish) OR (Congenital-primary-aphakia) OR (Congenital-pseudoarthrosis) OR (Congenital-pulmonary-alveolar-proteinosis) OR (Congenital-rubella) OR (Congenital-sucrase-isomaltase NEAR/3 deficien*) OR (Congenital-tracheal-stenosis) OR (Congenital-tracheomalacia) OR (Congenital-varicella NEAR/3 syndrom*) OR (Congenital-vertical-talus) OR (Congenitally-corrected-transposition NEAR/3 great-arteries) OR (Continuous-spike-wave-during-slow-sleep NEAR/3 syndrom*) OR (Convulsions NEAR/3 benign-familial-infantile NEAR/3 1) OR (Copper NEAR/3 deficien* NEAR/3 familial-benign) OR (Cor-triatriatum-dexter) OR (Cor-triatriatum-sinister) OR (Corneal-dystroph* NEAR/3 perceptive-deafness) OR (Corneal-dystroph*-Avellino) OR (Corneal-dystroph*-crystalline NEAR/3 Schnyder) OR (Corneal-dystroph*-Thiel-Behnke) OR (Corneal-endothelial-dystroph*-type-2) OR (Corneal-hypesthesia NEAR/3 familial) OR (Cornelia-de-Lange NEAR/3 syndrom*) OR (Corneodermatoosseous NEAR/3 syndrom*) OR (Corpus-callosum-agenesis-double-urinary-collecting) OR (Cortical-blindness-intellectual-disability-polydactyly NEAR/3 syndrom*) OR (Cortical NEAR/3 defect*s-wormian-bones NEAR/3 dentinogenesis-imperfecta) OR (Cortical-dysgenesis NEAR/3 pontocerebellar-hypoplasia-due-to-TUBB3-mutation) OR (Corticobasal-degeneration) OR (Corticosteroid-binding-globulin NEAR/3 deficien*) OR (Costello NEAR/3 syndrom*) OR (Cousin NEAR/3 syndrom*) OR (Cowden NEAR/3 syndrom*) OR (Crandall NEAR/3 syndrom*) OR (Crane-Heise NEAR/3 syndrom*) OR (Craniodiaphyseal-dysplasia) OR (Cranioectodermal-dysplasia) OR (Craniofacial-deafness-hand NEAR/3 syndrom*) OR (Craniofacial-dysostosis NEAR/3 diaphyseal-hyperplasia) OR (Craniofacial-dyssynostosis) OR (Craniofrontonasal-dysplasia) OR (Craniometaphyseal-dysplasia NEAR/3 autosomal-dominant) OR (Craniometaphyseal-dysplasia NEAR/3 autosomal-recessive) OR (Craniopharyngioma) OR (Craniorachischisis) OR (Craniosynostosis) OR (Craniosynostosis NEAR/3 anal-anomalies NEAR/3 -porokeratosis) OR (Craniotelencephalic-dysplasia) OR (Cri-du-chat NEAR/3 syndrom*) OR (Crigler-Najjar NEAR/3 syndrom* NEAR/3 type-1) OR (Crigler-Najjar NEAR/3 syndrom*-type-2) OR (Crome NEAR/3 syndrom*) OR (Cronkhite-Canada NEAR/3 diseas*) OR (Crouzon NEAR/3 syndrom*) OR (Crumpled-helices NEAR/3 small-mouth) OR (Cryptomicrotia-brachydactyly NEAR/3 syndrom*) OR (Cryptophthalmos) OR (Culler-Jones NEAR/3 syndrom*) OR (Curly-hair-acral-keratoderma-caries NEAR/3 syndrom*) OR (Currarino-triad) OR (Curry-Jones NEAR/3 syndrom*) OR (Cutis-laxa NEAR/3 autosomal-dominant) OR (Cylindrical-spirals-myopathy) OR (Cyprus-facial-neuromusculoskeletal NEAR/3 syndrom*) OR (Cystic-fibrosis) OR (Cystic-hygroma) OR (Cystinosis) OR (Czech-dysplasia-metatarsal) OR (D-ercole NEAR/3 syndrom*) OR (D-2-hydroxyglutaric-aciduria) OR (D-bifunctional-protein NEAR/3 deficien*) OR (D-glycericacidemia) OR (Daentl-Towsend-Siegel NEAR/3 syndrom*) OR (Daish-Hardman-Lamont NEAR/3 syndrom*) OR (Dandy-Walker-complex) OR (Dandy-Walker-cyst NEAR/3 Renal-Hepatic-Pancreatic-dysplasia) OR (Dandy-Walker-like-malformation NEAR/3 atrioventricular-septal NEAR/3 defect*) OR (Dandy-Walker-malformation NEAR/3 intellectual-disability NEAR/3 basal-ganglia NEAR/3 diseas* NEAR/3 seizures) OR (Dandy-Walker-malformation NEAR/3 nasopharyngeal-teratoma NEAR/3 diaphragmatic-hernia) OR (Dandy-Walker-malformation NEAR/3 postaxial-polydactyly) OR (Dandy-Walker-malformation NEAR/3 sagittal-craniosynostosis NEAR/3 hydrocephalus) OR (Daneman-Davy-Mancer NEAR/3 syndrom*) OR (Danon NEAR/3 diseas*) OR (Darier NEAR/3 diseas*) OR (Dauwerse-Peters NEAR/3 syndrom*) OR (Davenport-Donlan NEAR/3 syndrom*) OR (DCMA NEAR/3 syndrom*) OR (DDOST-CDG-) OR (CDG-Ir) OR (De-Barsy NEAR/3 syndrom*) OR (De-Sanctis-Cacchione NEAR/3 syndrom*) OR (DEAF1-associated-disorders) OR (Deafness NEAR/3 myopia NEAR/3 syndrom*) OR (Deafness-conductive-ptosis-skeletal-anomalies) OR (Deafness-enamel-hypoplasia-nail NEAR/3 defect*s) OR (Deafness-hypogonadism NEAR/3 syndrom*) OR (Deafness-oligodontia NEAR/3 syndrom*) OR (Deafness NEAR/3 labyrinthine-aplasia-microtia NEAR/3 microdontia-) OR (LAMM) OR (Deafness NEAR/3 autosomal-dominant-nonsyndromic-sensorineural-17) OR (Deafness NEAR/3 autosomal-dominant-nonsyndromic-sensorineural-22) OR (Deafness NEAR/3 autosomal-dominant-nonsyndromic-sensorineural-23) OR (Deafness NEAR/3 autosomal-dominant-nonsyndromic-sensorineural-24) OR (Deafness NEAR/3 autosomal-dominant-nonsyndromic-sensorineural-3) OR (Deafness NEAR/3 autosomal-dominant-nonsyndromic-sensorineural-53) OR (Deafness NEAR/3 autosomal-recessive-51) OR (Deafness NEAR/3 dystonia NEAR/3 -cerebral-hypomyelination) OR (Deafness NEAR/3 epiphyseal-dysplasia NEAR/3 short-stature) OR (Deafness NEAR/3 X-linked-2) OR (Deafness-infertility NEAR/3 syndrom*) OR (Deficiency NEAR/3 interleukin-1-receptor-antagonist) OR (Delayed-membranous-cranial-ossification) OR (Dendritic-cell NEAR/3 monocyte NEAR/3 B-lymphocyte NEAR/3 -natural-killer-lymphocyte NEAR/3 deficien*) OR (Dense-deposit NEAR/3 diseas*) OR (Dentatorubral-pallidoluysian-atrophy) OR (Dentin-dysplasia-sclerotic-bones) OR (Dentin-dysplasia NEAR/3 coronal) OR (Dentin-dysplasia NEAR/3 type-1) OR (Dentinogenesis-imperfecta) OR (Dentinogenesis-imperfecta-type-2) OR (Dentinogenesis-imperfecta-type-3) OR (Denys-Drash NEAR/3 syndrom*) OR (Dermatofibrosarcoma-protuberans) OR (Dermatoosteolysis-Kirghizian) OR (Dermatopathia-pigmentosa-reticularis) OR (Dermatosparaxis-Ehlers-Danlos NEAR/3 syndrom*) OR (Dermochondrocorneal-dystroph* NEAR/3 Francois) OR (Dermoodontodysplasia) OR (Desbuquois NEAR/3 syndrom*) OR (Desmoid-tumor) OR (Desmosterolosis) OR (Devriendt NEAR/3 syndrom*) OR (Dextrocardia) OR (Dextrocardia NEAR/3 unusual-facies NEAR/3 microphthalmia) OR (DFNB1) OR (Diaphyseal-medullary-stenosis NEAR/3 malignant-fibrous-histiocytoma) OR (Diastrophic-dysplasia) OR (Dicarboxylic-aminoaciduria) OR (Dihydrolipoamide-dehydrogenase NEAR/3 deficien*) OR (Dihydropteridine-reductase NEAR/3 deficien*) OR (Dihydropyrimidinase NEAR/3 deficien*) OR (Dilated-cardiomyopathy) OR (Dilated-cardiomyopathy NEAR/3 hypergonadotropic-hypogonadism) OR (Diphallia) OR (Disseminated-superficial-actinic-porokeratosis) OR (Distal-arthrogryposis-type-1) OR (Distal-arthrogryposis-type-5) OR (Distal-arthrogryposis-type-5D) OR (Distal-arthrogryposis NEAR/3 hypopituitarism NEAR/3 intellectual-disability NEAR/3 facial-anomalies) OR (Distal-myopathy NEAR/3 vocal-cord-weakness) OR (DK-phocomelia NEAR/3 syndrom*) OR (DOLK-CDG-) OR (CDG-Im) OR (Dominant-dystrophic-epidermolysis-bullosa) OR (Donnai-Barrow NEAR/3 syndrom*) OR (DOOR NEAR/3 syndrom*) OR (Dopa-responsive-dystonia) OR (Dopamine-beta-hydroxylase NEAR/3 deficien*) OR (Dopamine-transporter NEAR/3 deficien* NEAR/3 syndrom*) OR (Dowling-Degos NEAR/3 diseas*) OR (DPAGT1-CDG-) OR (CDG-Ij) OR (DPM1-CDG-) OR (CDG-Ie) OR (DPM2-CDG) OR (DPM3-CDG-) OR (CDG-Io) OR (Drachtman-Weinblatt-Sitarz NEAR/3 syndrom*) OR (Dravet NEAR/3 syndrom*) OR (Duane NEAR/3 syndrom*) OR (Duane-radial-ray NEAR/3 syndrom*) OR (Dubin-Johnson NEAR/3 syndrom*) OR (Dubowitz NEAR/3 syndrom*) OR (Duchenne-muscular-dystroph*) OR (Duodenal-atresia) OR (Duplication NEAR/3 urethra) OR (Dwarfism-familial NEAR/3 muscle-spasms) OR (Dwarfism-Levi) OR (Dwarfism NEAR/3 low-birth-weight-type NEAR/3 unresponsiveness-to-growth-hormone) OR (Dwarfism NEAR/3 proportionate NEAR/3 hip-dislocation) OR (Dyggve-Melchior-Clausen NEAR/3 syndrom*) OR (Dykes-Markes-Harper NEAR/3 syndrom*) OR (Dyschondrosteosis-nephritis) OR (Dyschromatosis-symmetrica-hereditaria-1) OR (Dyschromatosis-universalis-hereditaria) OR (Dysequilibrium NEAR/3 syndrom*) OR (Dysosteosclerosis) OR (Dysplasia-epiphysealis-hemimelica) OR (Dyssegmental-dysplasia NEAR/3 glaucoma) OR (Dyssegmental-dysplasia-Rolland-Desbuquois) OR (Dyssegmental-dysplasia-Silverman-Handmaker) OR (Dystelephalangy) OR (Dystonia-2 NEAR/3 torsion NEAR/3 autosomal-recessive) OR (DYT-PRKRA) OR (DYT-THAP1) OR (DYT-TOR1A) OR (DYT-TUBB4A) OR (Early-Infantile-Epileptic-Encephalopathy) OR (Early-infantile-epileptic-encephalopathy-25) OR (Early-onset-anterior-polar-cataract) OR (Early-onset-autosomal-dominant-Alzheimer NEAR/3 diseas*) OR (Early-onset-parkinsonism-intellectual-disability NEAR/3 syndrom*) OR (Early-onset-zonular-cataract) OR (Ebstein NEAR/3 -anomaly) OR (Ectodermal-dysplasia) OR (Ectodermal-dysplasia-skin-fragility NEAR/3 syndrom*) OR (Ectodermal-dysplasia-trichoodontoonychial) OR (Ectodermal-dysplasia NEAR/3 natal-teeth-Turnpenny) OR (Ectodermal-dysplasia NEAR/3 hidrotic NEAR/3 Christianson-Fourie) OR (Ectodermal-dysplasia NEAR/3 sensorineural-hearing-loss NEAR/3 -distinctive-facial-features) OR (EEC NEAR/3 syndrom*) OR (EEM NEAR/3 syndrom*) OR (Eisenmenger NEAR/3 syndrom*) OR (Elastosis-perforans-serpiginosa) OR (Ellis-Yale-Winter NEAR/3 syndrom*) OR (Ellis-Van-Creveld NEAR/3 syndrom*) OR (Encephalocele) OR (Encephalocraniocutaneous-lipomatosis) OR (Encephalopathy-due-to-prosaposin NEAR/3 deficien*) OR (Encephalopathy-intracranial-calcification-growth-hormone NEAR/3 deficien*-microcephaly-retinal-degeneration) OR (Epidermodysplasia-verruciformis) OR (Epidermolysa-bullosa-simplex NEAR/3 muscular-dystroph*) OR (Epidermolysis-bullosa-simplex NEAR/3 mottled-pigmentation) OR (Epidermolysis-bullosa-simplex NEAR/3 Dowling-Meara) OR (Epidermolysis-bullosa-simplex NEAR/3 localized) OR (Epidermolysis-bullosa-simplex NEAR/3 Ogna) OR (Epidermolysis-bullosa NEAR/3 lethal-acantholytic) OR (Epidermolytic-ichthyosis) OR (Epidermolytic-palmoplantar-keratoderma) OR (Epilepsy-juvenile-absence) OR (Epilepsy NEAR/3 myoclonic-atonic-seizures) OR (Epiphyseal-dysplasia-hearing-loss-dysmorphism) OR (Epiphyseal-dysplasia-multiple NEAR/3 early-onset-diabetes-mellitus) OR (Episodic-ataxia NEAR/3 nystagmus) OR (Ermine-phenotype) OR (Erythrokeratoderma-en-cocardes) OR (Erythromelalgia) OR (Erythropoietic-uroporphyria-associated NEAR/3 myeloid-malignancy) OR (Esophageal-atresia) OR (Ethylmalonic-encephalopathy) OR (Eunuchoidism-familial-hypogonadotropic) OR (Exstrophy NEAR/3 bladder) OR (FACES NEAR/3 syndrom*) OR (Facial-ectodermal-dysplasia) OR (Facial-onset-sensory NEAR/3 motor-neuronopathy) OR (Facio-thoraco-genital NEAR/3 syndrom*) OR (Faciocardiorenal NEAR/3 syndrom*) OR (Facioscapulohumeral-muscular-dystroph*) OR (Fallot-complex NEAR/3 severe-mental NEAR/3 growth-retardation) OR (Familial-amyloidosis NEAR/3 Finnish) OR (Familial-atrial-fibrillation) OR (Familial-avascular-necrosis NEAR/3 femoral-head) OR (Familial-bilateral-striatal-necrosis) OR (Familial-caudal-dysgenesis) OR (Familial-cold-autoinflammatory NEAR/3 syndrom*) OR (Familial-congenital-palsy NEAR/3 trochlear-nerve) OR (Familial-cutaneous-collagenoma) OR (Familial-dilated-cardiomyopathy) OR (Familial-dysautonomia) OR (Familial-encephalopathy NEAR/3 neuroserpin-inclusion-bodies) OR (Familial-exudative-vitreoretinopathy) OR (Familial-focal-epilepsy NEAR/3 variable-foci) OR (Familial-glucocorticoid NEAR/3 deficien*) OR (Familial-HDL NEAR/3 deficien*) OR (Familial-hemiplegic-migraine) OR (Familial-hemophagocytic-lymphohistiocytosis) OR (Familial-hyperaldosteronism-type-2) OR (Familial-hyperaldosteronism-type-III) OR (Familial-hypocalciuric-hypercalcemia-type-1) OR (Familial-hypocalciuric-hypercalcemia-type-2) OR (Familial-hypocalciuric-hypercalcemia-type-3) OR (Familial-infantile-convulsions NEAR/3 paroxysmal-choreoathetosis) OR (Familial-joint-instability NEAR/3 syndrom*) OR (Familial-lipoprotein-lipase NEAR/3 deficien*) OR (Familial-Mediterranean-fever) OR (Familial-multiple-lipomatosis) OR (Familial-osteochondritis-dissecans) OR (Familial-partial-lipodystroph*-associated NEAR/3 PLIN1-mutations) OR (Familial-partial-lipodystroph*-associated NEAR/3 PPARG-mutations) OR (Familial-partial-lipodystroph*-due-to-AKT2-mutations) OR (Familial-partial-lipodystroph*-type-2) OR (Familial-partial-lipodystroph*-type-Kobberling) OR (Familial-porencephaly) OR (Familial-progressive-cardiac-conduction NEAR/3 defect*) OR (Familial-reactive-perforating-collagenosis) OR (Familial-thyroglossal-duct-cyst) OR (Familial-visceral-myopathy NEAR/3 external-ophthalmoplegia) OR (Familiar-hemiplegic-migraine) OR (Sporadic-hemiplegic-migraine) OR (Fanconi-Bickel NEAR/3 syndrom*) OR (Fanconi NEAR/3 syndrom*) OR (Farber NEAR/3 diseas*) OR (Fatal-familial-insomnia) OR (Fatty-acid-hydroxylase-associated-neurodegeneration) OR (Faye-Petersen-Ward-Carey NEAR/3 syndrom*) OR (FBXL4-related-encephalomyopathic-mitochondrial-DNA-depletion NEAR/3 syndrom*) OR (Feigenbaum-Bergeron-Richardson NEAR/3 syndrom*) OR (Feingold NEAR/3 syndrom*) OR (Femoral-facial NEAR/3 syndrom*) OR (Femur-bifid NEAR/3 monodactylous-ectrodactyly) OR (Femur-fibula-ulna NEAR/3 syndrom*) OR (Fertile-eunuch NEAR/3 syndrom*) OR (Fetal-akinesia-deformation-sequence) OR (Fetal-aminopterin NEAR/3 syndrom*) OR (Fetal-cystic-hygroma) OR (Fetal-hydantoin NEAR/3 syndrom*) OR (Fetal-methylmercury NEAR/3 syndrom*) OR (Fetal-retinoid NEAR/3 syndrom*) OR (Fetal-thalidomide NEAR/3 syndrom*) OR (Fetal-valproate NEAR/3 syndrom*) OR (FG NEAR/3 syndrom*) OR (FG NEAR/3 syndrom*-2) OR (FG NEAR/3 syndrom*-3) OR (Fibro-adipose-vascular-anomaly) OR (Fibrochondrogenesis) OR (Fibrodysplasia-ossificans-progressiva) OR (Fibrous-dysplasia) OR (Fibular-aplasia-ectrodactyly) OR (Fibular-aplasia NEAR/3 tibial-campomelia NEAR/3 -oligosyndactyly NEAR/3 syndrom*) OR (Fibular-hemimelia) OR (Fibular-hypoplasia NEAR/3 complex-brachydactyly) OR (Filippi NEAR/3 syndrom*) OR (Fine-Lubinsky NEAR/3 syndrom*) OR (Fingerprint-body-myopathy) OR (Fish-eye NEAR/3 diseas*) OR (Fitzsimmons NEAR/3 syndrom*) OR (Fitzsimmons-Walson-Mellor NEAR/3 syndrom*) OR (Fitzsimmons-Guilbert NEAR/3 syndrom*) OR (Floating-Harbor NEAR/3 syndrom*) OR (Flynn-Aird NEAR/3 syndrom*) OR (Focal-cortical-dysplasia NEAR/3 Taylor) OR (Focal-dermal-hypoplasia) OR (Focal-facial-dermal-dysplasia) OR (Focal-segmental-glomerulosclerosis) OR (Follicle-stimulating-hormone NEAR/3 deficien* NEAR/3 isolated) OR (Fountain NEAR/3 syndrom*) OR (FOXG1 NEAR/3 syndrom*) OR (Fragile-X NEAR/3 syndrom*) OR (Fragile-XE NEAR/3 syndrom*) OR (Frank-Ter-Haar NEAR/3 syndrom*) OR (Fraser NEAR/3 syndrom*) OR (Frasier NEAR/3 syndrom*) OR (Free-sialic-acid-storage NEAR/3 diseas*) OR (Freeman-Sheldon NEAR/3 syndrom*) OR (Frias NEAR/3 syndrom*) OR (Friedreich-ataxia) OR (Frints-De-Smet-Fabry-Fryns NEAR/3 syndrom*) OR (Frontofacionasal-dysplasia) OR (Frontometaphyseal-dysplasia) OR (Frontonasal-dysplasia) OR (Frontonasal-dysplasia NEAR/3 alopecia NEAR/3 genital-anomaly) OR (Frontonasal-dysplasia-severe-microphthalmia-severe-facial-clefting NEAR/3 syndrom*) OR (Frontorhiny) OR (Frontotemporal-dementia NEAR/3 ubiquitin-positive) OR (Froster-Huch NEAR/3 syndrom*) OR (Fructose-1-6-bisphosphatase NEAR/3 deficien*) OR (Fryns-Hofkens-Fabry NEAR/3 syndrom*) OR (Fryns NEAR/3 syndrom*) OR (Fucosidosis) OR (Fuhrmann NEAR/3 syndrom*) OR (Fukuyama-type-muscular-dystroph*) OR (Fumarase NEAR/3 deficien*) OR (Fused-mandibular-incisors) OR (Galactokinase NEAR/3 deficien*) OR (Galactosemia) OR (Galactosialidosis) OR (Galloway-Mowat NEAR/3 syndrom*) OR (Game-Friedman-Paradice NEAR/3 syndrom*) OR (Gamma-aminobutyric-acid-transaminase NEAR/3 deficien*) OR (Gamma-cystathionase NEAR/3 deficien*) OR (GAPO NEAR/3 syndrom*) OR (Gardner NEAR/3 syndrom*) OR (Garret-Tripp NEAR/3 syndrom*) OR (Gastrocutaneous NEAR/3 syndrom*) OR (Gastrointestinal-Stromal-Tumors) OR (Gastroschisis) OR (GATAD2B-associated-neurodevelopmental-disorder) OR (Gaucher NEAR/3 diseas*---ophthalmoplegia---cardiovascular-calcification) OR (Gaucher NEAR/3 diseas*-perinatal-lethal) OR (Gaucher NEAR/3 diseas*-type-1) OR (Gaucher NEAR/3 diseas*-type-2) OR (Gaucher NEAR/3 diseas*-type-3) OR (Gay-Feinmesser-Cohen NEAR/3 syndrom*) OR (Geleophysic-dwarfism) OR (Gemignani NEAR/3 syndrom*) OR (Generalized-junctional-epidermolysis-bullosa NEAR/3 non-Herlitz) OR (Generalized-pustular-psoriasis) OR (Genito-palato-cardiac NEAR/3 syndrom*) OR (Genitopatellar NEAR/3 syndrom*) OR (Genoa NEAR/3 syndrom*) OR (Genochondromatosis) OR (Genu-valgum NEAR/3 st-Helena-familial) OR (Geroderma-osteodysplastica) OR (Gerstmann-Straussler-Scheinker NEAR/3 diseas*) OR (Gestational-trophoblastic-tumor) OR (Ghosal-hematodiaphyseal-dysplasia NEAR/3 syndrom*) OR (Ghose-Sachdev-Kumar NEAR/3 syndrom*) OR (Giant-axonal-neuropathy) OR (Giant-congenital-nevus) OR (Gillespie NEAR/3 syndrom*) OR (Gingival-fibromatosis NEAR/3 distinctive-facies) OR (Gingival-fibromatosis NEAR/3 hypertrichosis) OR (Gitelman NEAR/3 syndrom*) OR (Glaucoma-sleep-apnea) OR (Glaucoma NEAR/3 Ectopia NEAR/3 Microspherophakia NEAR/3 Stiff-joints NEAR/3 Short-stature NEAR/3 syndrom*) OR (Globozoospermia) OR (Glomerulonephritis NEAR/3 sparse-hair NEAR/3 telangiectases) OR (Glomerulopathy NEAR/3 fibronectin-deposits-1) OR (Glomerulopathy NEAR/3 fibronectin-deposits-2) OR (Glucose-transporter-type-1 NEAR/3 deficien* NEAR/3 syndrom*) OR (Glucose-galactose-malabsorption) OR (Glutamine NEAR/3 deficien* NEAR/3 congenital) OR (Glutaric-acidemia-type-I) OR (Glutaric-acidemia-type-II) OR (Glutaric-acidemia-type-III) OR (Glutathionuria) OR (Glycine-N-methyltransferase NEAR/3 deficien*) OR (Glycogen-storage NEAR/3 diseas*-type-0 NEAR/3 liver) OR (Glycogen-storage NEAR/3 diseas*-type-13) OR (Glycogen-storage NEAR/3 diseas*-type-1A) OR (Glycogen-storage NEAR/3 diseas*-type-1B) OR (Glycogen-storage NEAR/3 diseas*-type-3) OR (Glycogen-storage NEAR/3 diseas*-type-4) OR (Glycogen-storage NEAR/3 diseas*-type-5) OR (Glycogen-storage NEAR/3 diseas*-type-6) OR (GM1-gangliosidosis-type-1) OR (GM1-gangliosidosis-type-2) OR (GM1-gangliosidosis-type-3) OR (GM3-synthase NEAR/3 deficien*) OR (GMS NEAR/3 syndrom*) OR (Gnathodiaphyseal-dysplasia) OR (Goldberg-Shprintzen-megacolon NEAR/3 syndrom*) OR (Goldenhar NEAR/3 diseas*) OR (Goldmann-Favre NEAR/3 syndrom*) OR (Gomez-Lopez-Hernandez NEAR/3 syndrom*) OR (Gordon NEAR/3 syndrom*) OR (Gorlin-Chaudhry-Moss NEAR/3 syndrom*) OR (GOSR2-related-progressive-myoclonus-ataxia) OR (Gracile-bone-dysplasia) OR (GRACILE NEAR/3 syndrom*) OR (Graham-Boyle-Troxell NEAR/3 syndrom*) OR (Graham-Cox NEAR/3 syndrom*) OR (Graham-Little-Piccardi-Lassueur NEAR/3 syndrom*) OR (Grant NEAR/3 syndrom*) OR (Greenberg-dysplasia) OR (Greig-cephalopolysyndactyly NEAR/3 syndrom*) OR (Griscelli NEAR/3 syndrom*-type-1) OR (Griscelli NEAR/3 syndrom*-type-2) OR (Griscelli NEAR/3 syndrom*-type-3) OR (Groll-Hirschowitz NEAR/3 syndrom*) OR (Growth-hormone-insensitivity NEAR/3 immunodeficiency) OR (Grubben-de-Cock-Borghgraef NEAR/3 syndrom*) OR (GTP-cyclohydrolase-I NEAR/3 deficien*) OR (GTPCH1-deficient-DRD) OR (Guanidinoacetate-methyltransferase NEAR/3 deficien*) OR (Guizar-Vasquez-Sanchez-Manzano NEAR/3 syndrom*) OR (Gurrieri NEAR/3 syndrom*) OR (Gyrate-atrophy NEAR/3 choroid NEAR/3 retina) OR (Hailey-Hailey NEAR/3 diseas*) OR (Haim-Munk NEAR/3 syndrom*) OR (Hair NEAR/3 defect*-photosensitivity-intellectual-disability NEAR/3 syndrom*) OR (Hairy-elbows) OR (Halal NEAR/3 syndrom*) OR (Hall-Riggs NEAR/3 syndrom*) OR (Hallermann-Streiff NEAR/3 syndrom*) OR (Hamanishi-Ueba-Tsuji NEAR/3 syndrom*) OR (Hand NEAR/3 foot-deformity NEAR/3 flat-facies) OR (Hand-foot-uterus NEAR/3 syndrom*) OR (Hanhart NEAR/3 syndrom*) OR (Hard-skin NEAR/3 syndrom*-Parana) OR (Hardikar NEAR/3 syndrom*) OR (Harding-ataxia) OR (Harlequin-ichthyosis) OR (Harlequin NEAR/3 syndrom*) OR (Harrod-Doman-Keele NEAR/3 syndrom*) OR (Hartnup NEAR/3 diseas*) OR (Hawkinsinuria) OR (Heart NEAR/3 defect*-tongue-hamartoma-polysyndactyly NEAR/3 syndrom*) OR (Heart-hand NEAR/3 syndrom* NEAR/3 Slovenian) OR (Heart-hand NEAR/3 syndrom* NEAR/3 Spanish) OR (HEC NEAR/3 syndrom*) OR (Hemangiomatosis NEAR/3 familial-pulmonary-capillary) OR (Hemi-3 NEAR/3 syndrom*) OR (Hemifacial-hyperplasia-strabismus) OR (Hemifacial-microsomia) OR (Hemimegalencephaly) OR (Hepatic-lipase NEAR/3 deficien*) OR (Hepatic-venoocclusive NEAR/3 diseas* NEAR/3 immunodeficiency) OR (Hepatoerythropoietic-porphyria) OR (Hereditary-angiopathy NEAR/3 nephropathy NEAR/3 aneurysms NEAR/3 -muscle-cramps NEAR/3 syndrom*) OR (Hereditary-congenital-facial-paresis) OR (Hereditary-coproporphyria) OR (Hereditary-diffuse-leukoencephalopathy NEAR/3 spheroids) OR (Hereditary-fibrosing-poikiloderma NEAR/3 tendon-contractures NEAR/3 myopathy NEAR/3 -pulmonary-fibrosis) OR (Hereditary-fructose-intolerance) OR (Hereditary-geniospasm) OR (Hereditary-hyperekplexia) OR (Hereditary-keratitis) OR (Hereditary-koilonychia) OR (Hereditary-leiomyomatosis NEAR/3 renal-cell-cancer) OR (Hereditary-motor NEAR/3 sensory-neuropathy-type-5) OR (Hereditary-mucoepithelial-dysplasia) OR (Hereditary-multiple-osteochondromas) OR (Hereditary-neuralgic-amyotrophy) OR (Hereditary-neuropathy NEAR/3 liability-to-pressure-palsies) OR (Hereditary-pancreatitis) OR (Hereditary-proximal-myopathy NEAR/3 early-respiratory-failure) OR (Hereditary-sensorimotor-neuropathy NEAR/3 hyperelastic-skin) OR (Hereditary-sensory NEAR/3 autonomic-neuropathy-type-1E) OR (Hereditary-sensory NEAR/3 autonomic-neuropathy-type-2) OR (Hereditary-sensory NEAR/3 autonomic-neuropathy-type-7) OR (Hereditary-sensory NEAR/3 autonomic-neuropathy-type-V) OR (Hereditary-sensory-neuropathy-type-1) OR (Hereditary-vascular-retinopathy) OR (Hermansky-Pudlak NEAR/3 syndrom*) OR (Hernandez-Aguirre-Negrete NEAR/3 syndrom*) OR (Herpes-simplex-encephalitis) OR (Heterotaxy) OR (HIBCH NEAR/3 deficien*) OR (Hirschsprung NEAR/3 diseas*) OR (Hirschsprung NEAR/3 diseas*-type-d-brachydactyly) OR (His-bundle-tachycardia) OR (Histidinemia) OR (HMG-CoA-lyase NEAR/3 deficien*) OR (Holocarboxylase-synthetase NEAR/3 deficien*) OR (Holt-Oram NEAR/3 syndrom*) OR (Holzgreve NEAR/3 syndrom*) OR (Homocarnosinosis) OR (Homocystinuria-due-to-CBS NEAR/3 deficien*) OR (Homocystinuria-due-to-MTHFR NEAR/3 deficien*) OR (Horizontal-gaze-palsy NEAR/3 progressive-scoliosis) OR (HSD10 NEAR/3 diseas*) OR (Human-HOXA1 NEAR/3 syndrom*s) OR (Hunter-McAlpine NEAR/3 syndrom*) OR (Huntington NEAR/3 diseas*) OR (Hurler NEAR/3 syndrom*) OR (Hurler-Scheie NEAR/3 syndrom*) OR (Hutterite-cerebroosteonephrodysplasia NEAR/3 syndrom*) OR (Hyaline-fibromatosis NEAR/3 syndrom*) OR (Hydranencephaly) OR (Hydrocephalus-due-to-congenital-stenosis NEAR/3 aqueduct NEAR/3 sylvius) OR (Hydrocephalus-obesity-hypogonadism) OR (Hydrocephalus NEAR/3 costovertebral-dysplasia NEAR/3 -Sprengel-anomaly) OR (Hydrocephalus-cleft-palate-joint-contractures NEAR/3 syndrom*) OR (Hydroxykynureninuria) OR (Hyper-IgD NEAR/3 syndrom*) OR (Hyperbetaalaninemia) OR (Hyperbilirubinemia-transient-familial-neonatal) OR (Hyperferritinemia-cataract NEAR/3 syndrom*) OR (Hyperinsulinism-due-to-glucokinase NEAR/3 deficien*) OR (Hyperinsulinism-hyperammonemia NEAR/3 syndrom*) OR (Hyperkalemic-periodic-paralysis) OR (Hyperkeratosis-lenticularis-perstans) OR (Hyperlipidemia-type-3) OR (Hyperlipoproteinemia-type-5) OR (Hyperlysinemia) OR (Hypermethioninemia-due-to-S-adenosylhomocysteine-hydrolase NEAR/3 deficien*) OR (Hypermobile-Ehlers-Danlos NEAR/3 syndrom*) OR (Hyperostosis-corticalis-generalisata) OR (Hyperparathyroidism-jaw-tumor NEAR/3 syndrom*) OR (Hyperphenylalaninemia-due-to-dehydratase NEAR/3 deficien*) OR (Hyperprolinemia) OR (Hyperprolinemia-type-2) OR (Hypertelorism NEAR/3 tetralogy NEAR/3 Fallot) OR (Hyperthermia-induced NEAR/3 defect*s) OR (Hypertrichosis-lanuginosa-congenita) OR (Hypertrophic-neuropathy NEAR/3 Dejerine-Sottas) OR (Hypertryptophanemia) OR (Hypocalcemia NEAR/3 autosomal-dominant) OR (Hypochondroplasia) OR (Hypohidrotic-ectodermal-dysplasia-autosomal-recessive) OR (Hypohidrotic-ectodermal-dysplasia NEAR/3 hypothyroidism NEAR/3 ciliary-dyskinesia) OR (Hypokalemic-periodic-paralysis) OR (Hypomandibular-faciocranial-dysostosis) OR (Hypomelanosis NEAR/3 Ito) OR (Hypomyelination NEAR/3 congenital-cataract) OR (Hypomyelination NEAR/3 atrophy NEAR/3 basal-ganglia NEAR/3 cerebellum) OR (Hypoparathyroidism-intellectual-disability-dysmorphism NEAR/3 syndrom*) OR (Hypophosphatasia) OR (Hypophosphatemic-rickets) OR (Hypoplastic-left-heart NEAR/3 syndrom*) OR (Hypospadias-familial) OR (Hypospadias-intellectual-disability NEAR/3 Goldblatt-type NEAR/3 syndrom*) OR (Hypotelorism-cleft-palate-hypospadias) OR (Hypotonia NEAR/3 congenital-nystagmus NEAR/3 ataxia NEAR/3 abnormal-auditory-brainstem-response) OR (Hypotrichosis-simplex) OR (I-cell NEAR/3 diseas*) OR (ICF NEAR/3 syndrom*) OR (Ichthyosiform-erythroderma NEAR/3 corneal-involvement NEAR/3 deafness) OR (Ichthyosis-alopecia-eclabion-ectropion-intellectual-disability) OR (Ichthyosis-bullosa NEAR/3 Siemens) OR (Ichthyosis-cheek-eyebrow NEAR/3 syndrom*) OR (Ichthyosis-follicularis-atrichia-photophobia NEAR/3 syndrom*) OR (Ichthyosis-hystrix NEAR/3 Curth-Macklin) OR (Ichthyosis-lamellar-1) OR (Ichthyosis-lamellar-2) OR (Ichthyosis-lamellar-3) OR (Ichthyosis-lamellar NEAR/3 autosomal-dominant) OR (Ichthyosis-prematurity NEAR/3 syndrom*) OR (Ichthyosis-tapered-fingers-midline-groove-up) OR (Ichthyosis NEAR/3 hypotrichosis NEAR/3 autosomal-recessive) OR (Ichthyosis NEAR/3 leukocyte-vacuoles NEAR/3 alopecia NEAR/3 -sclerosing-cholangitis) OR (Ichthyosis-intellectual-disability-dwarfism-renal-impairment) OR (Idiopathic-basal-ganglia-calcification-childhood-onset) OR (Idiopathic-CD4-positive-T-lymphocytopenia) OR (Iida-Kannari NEAR/3 syndrom*) OR (IL12RB1 NEAR/3 deficien*) OR (IMAGe NEAR/3 syndrom*) OR (Iminoglycinuria) OR (Immune NEAR/3 defect*-due-to-absence NEAR/3 thymus) OR (Immunodeficiency NEAR/3 hyper-IgM-type-1) OR (Immunodeficiency NEAR/3 hyper-IgM-type-2) OR (Immunodeficiency NEAR/3 hyper-IgM-type-3) OR (Immunodeficiency NEAR/3 hyper-IgM-type-4) OR (Immunodeficiency NEAR/3 hyper-IgM-type-5) OR (Immunodysregulation NEAR/3 polyendocrinopathy NEAR/3 enteropathy-X-linked) OR (Imperforate-oropharynx-costo-vetebral-anomalies) OR (Inclusion-body-myopathy-2) OR (Inclusion-body-myopathy-3) OR (Inclusion-body-myopathy NEAR/3 early-onset-Paget NEAR/3 diseas* NEAR/3 frontotemporal-dementia) OR (Incontinentia-pigmenti) OR (Infantile-axonal-neuropathy) OR (Infantile-cerebellar-retinal-degeneration) OR (Infantile-choroidocerebral-calcification NEAR/3 syndrom*) OR (Infantile-histiocytoid-cardiomyopathy) OR (Infantile-liver-failure NEAR/3 syndrom*-1) OR (Infantile-myofibromatosis) OR (Infantile-neuroaxonal-dystroph*) OR (Infantile-onset-spinocerebellar-ataxia) OR (Infantile-spasms-broad-thumbs) OR (Infantile-onset-ascending-hereditary-spastic-paralysis) OR (Iniencephaly) OR (Insulin-like-growth-factor-1-resistance-to) OR (Insulin-like-growth-factor-I NEAR/3 deficien*) OR (Insulin-resistance-type-B) OR (Intellectual-deficit---short-stature---hypertelorism) OR (Intellectual-deficit-Buenos-Aires) OR (Intellectual-disability---athetosis---microphthalmia) OR (Intellectual-disability---hypoplastic-corpus-callosum---preauricular-tag) OR (Intellectual-disability-developmental-delay-contractures NEAR/3 syndrom*) OR (Intellectual-disability-dysmorphism-hypogonadism-diabetes-mellitus NEAR/3 syndrom*) OR (Intellectual-disability-severe-speech-delay-mild-dysmorphism NEAR/3 syndrom*) OR (Intellectual-disability-spasticity-ectrodactyly NEAR/3 syndrom*) OR (Intermediate-congenital-nemaline-myopathy) OR (Intermediate-severe-Salla NEAR/3 diseas*) OR (Intestinal-atresia-multiple) OR (Intrauterine-growth-retardation NEAR/3 increased-mitomycin-C-sensitivity) OR (IRAK-4 NEAR/3 deficien*) OR (Iridogoniodysgenesis NEAR/3 skeletal-anomalies) OR (Iris-hypoplasia NEAR/3 glaucoma) OR (Irons-Bhan NEAR/3 syndrom*) OR (IRVAN NEAR/3 syndrom*) OR (Isobutyryl-CoA-dehydrogenase NEAR/3 deficien*) OR (Isodicentric-chromosome-15 NEAR/3 syndrom*) OR (Isolated-ACTH NEAR/3 deficien*) OR (Isolated-anterior-cervical-hypertrichosis) OR (Isolated-congenital-megalocornea) OR (Isolated-ectopia-lentis) OR (Isolated-growth-hormone NEAR/3 deficien*-type-1A) OR (Isolated-growth-hormone NEAR/3 deficien*-type-1B) OR (Isolated-growth-hormone NEAR/3 deficien*-type-2) OR (Isolated-growth-hormone NEAR/3 deficien*-type-3) OR (Isotretinoin-embryopathy-like NEAR/3 syndrom*) OR (Isovaleric-acidemia) OR (Ivemark NEAR/3 syndrom*) OR (IVIC NEAR/3 syndrom*) OR (Jackson-Weiss NEAR/3 syndrom*) OR (Jansen-type-metaphyseal-chondrodysplasia) OR (Jejunal-atresia) OR (Jejunal-atresia NEAR/3 renal-adysplasia) OR (Jervell-Lange-Nielsen NEAR/3 syndrom*) OR (Jeune NEAR/3 syndrom*) OR (Johanson-Blizzard NEAR/3 syndrom*) OR (Johnson-Munson NEAR/3 syndrom*) OR (Johnson-neuroectodermal NEAR/3 syndrom*) OR (Johnston-Aarons-Schelley NEAR/3 syndrom*) OR (Jones NEAR/3 syndrom*) OR (Joubert NEAR/3 syndrom*) OR (Joubert NEAR/3 syndrom* NEAR/3 oculorenal-anomalies) OR (Juberg-Marsidi NEAR/3 syndrom*) OR (Juberg-Hayward NEAR/3 syndrom*) OR (Junctional-epidermolysis-bullosa-generalized-intermediate) OR (Junctional-epidermolysis-bullosa NEAR/3 Herlitz) OR (Juvenile-amyotrophic-lateral-sclerosis) OR (Juvenile-osteoporosis) OR (Juvenile-Paget NEAR/3 diseas*) OR (Juvenile-polyposis NEAR/3 syndrom*) OR (Juvenile-primary-lateral-sclerosis) OR (Juvenile-retinoschisis) OR (Juvenile-onset-dystonia) OR (Kabuki NEAR/3 syndrom*) OR (Kallmann NEAR/3 syndrom*) OR (Kallmann NEAR/3 syndrom*-1) OR (Kallmann NEAR/3 syndrom*-2) OR (Kaplan-Plauchu-Fitch NEAR/3 syndrom*) OR (Kapur-Toriello NEAR/3 syndrom*) OR (Karak NEAR/3 syndrom*) OR (Kartagener NEAR/3 syndrom*) OR (Kaufman-oculocerebrofacial NEAR/3 syndrom*) OR (KBG NEAR/3 syndrom*) OR (KCNQ2-Related-Disorders) OR (Kearns-Sayre NEAR/3 syndrom*) OR (Kennedy NEAR/3 diseas*) OR (Kenny-Caffey NEAR/3 syndrom*-type-1) OR (Kenny-Caffey NEAR/3 syndrom*-type-2) OR (Keratoderma-palmoplantar-deafness) OR (Keratoderma-palmoplantar-spastic-paralysis) OR (Keratoderma-palmoplantaris-transgrediens) OR (Keratolytic-winter-erythema) OR (Keratosis-follicularis-dwarfism NEAR/3 cerebral-atrophy) OR (Keratosis-follicularis-spinulosa-decalvans) OR (Kernicterus) OR (Keutel NEAR/3 syndrom*) OR (KID NEAR/3 syndrom*) OR (Kindler NEAR/3 syndrom*) OR (King-Denborough NEAR/3 syndrom*) OR (Kleeblattschaedel NEAR/3 syndrom*) OR (Kleine-Levin NEAR/3 syndrom*) OR (Kleiner-Holmes NEAR/3 syndrom*) OR (Klippel-Feil NEAR/3 syndrom*) OR (Kniest-dysplasia) OR (Kniest-like-dysplasia-lethal) OR (Kniest-like-dysplasia NEAR/3 pursed-lips NEAR/3 ectopia-lentis) OR (Knobloch NEAR/3 syndrom*) OR (Knuckle-pads NEAR/3 leuconychia NEAR/3 sensorineural-deafness) OR (Kohlschutter-Tonz NEAR/3 syndrom*) OR (Koone-Rizzo-Elias NEAR/3 syndrom*) OR (Kosztolanyi NEAR/3 syndrom*) OR (Kotzot-Richter NEAR/3 syndrom*) OR (Kowarski NEAR/3 syndrom*) OR (Kozlowski-Warren-Fisher NEAR/3 syndrom*) OR (Kozlowski-Krajewska NEAR/3 syndrom*) OR (Kuskokwim NEAR/3 diseas*) OR (Kyphomelic-dysplasia) OR (Kyphoscoliotic-Ehlers-Danlos NEAR/3 syndrom*) OR (L-2-hydroxyglutaric-aciduria) OR (L-arginine-glycine-amidinotransferase NEAR/3 deficien*) OR (Laband NEAR/3 syndrom*) OR (Lachiewicz-Sibley NEAR/3 syndrom*) OR (Lacrimo-auriculo-dento-digital NEAR/3 syndrom*) OR (Lafora NEAR/3 diseas*) OR (Laing-distal-myopathy) OR (Lambdoid-synostosis) OR (Lambert NEAR/3 syndrom*) OR (Lamellar-ichthyosis) OR (Landau-Kleffner NEAR/3 syndrom*) OR (Langer-mesomelic-dysplasia) OR (Laron NEAR/3 syndrom*) OR (Larsen NEAR/3 syndrom*) OR (Larsen-like NEAR/3 syndrom*) OR (Laryngomalacia) OR (Laryngoonychocutaneous NEAR/3 syndrom*) OR (Larynx-atresia) OR (Larynx NEAR/3 congenital-partial-atresia-of) OR (Late-onset-distal-myopathy NEAR/3 Markesbery-Griggs) OR (Late-onset-junctional-epidermolysis-bullosa) OR (Late-onset-localized-junctional-epidermolysis-bullosa-intellectual-disability NEAR/3 syndrom*) OR (Late-onset-retinal-degeneration) OR (Lateral-meningocele NEAR/3 syndrom*) OR (Lateral-semicircular-canal-malformation NEAR/3 familial NEAR/3 external NEAR/3 middle-ear-abnormalities) OR (Lathosterolosis) OR (Lattice-corneal-dystroph*-type-1) OR (Laurence-Moon NEAR/3 syndrom*) OR (Laurin-Sandrow NEAR/3 syndrom*) OR (LCHAD NEAR/3 deficien*) OR (Le-Marec-Bracq-Picaud NEAR/3 syndrom*) OR (Leber-congenital-amaurosis) OR (Leber-congenital-amaurosis-5) OR (Leber-hereditary-optic-neuropathy) OR (Leber-hereditary-optic-neuropathy NEAR/3 dystonia) OR (Left-ventricular-noncompaction) OR (Left-sided-gallbladder) OR (Legg-Calve-Perthes NEAR/3 diseas*) OR (Legius NEAR/3 syndrom*) OR (Leigh NEAR/3 syndrom*) OR (Leigh NEAR/3 syndrom* NEAR/3 French-Canadian) OR (Lelis NEAR/3 syndrom*) OR (Lennox-Gastaut NEAR/3 syndrom*) OR (Lenz-Majewski-hyperostotic-dwarfism) OR (Lenz-microphthalmia NEAR/3 syndrom*) OR (LEOPARD NEAR/3 syndrom*) OR (Leprechaunism) OR (Leri-pleonosteosis) OR (Leri-Weill-dyschondrosteosis) OR (Lethal-chondrodysplasia-Moerman) OR (Lethal-chondrodysplasia-Seller) OR (Lethal-congenital-contracture NEAR/3 syndrom*-1) OR (Lethal-congenital-contracture NEAR/3 syndrom*-2) OR (Lethal-congenital-contracture NEAR/3 syndrom*-3) OR (Lethal-short-limb-skeletal-dysplasia-Al-Gazali) OR (Leukocyte-adhesion NEAR/3 deficien*-type-1) OR (Leukodystroph*) OR (Leukoencephalopathy NEAR/3 dystonia NEAR/3 motor-neuropathy) OR (Leukoencephalopathy-palmoplantar-keratoderma) OR (Leukoencephalopathy NEAR/3 brain-stem NEAR/3 spinal-cord-involvement NEAR/3 lactate-elevation) OR (Leukoencephalopathy NEAR/3 thalamus NEAR/3 brainstem-involvement NEAR/3 high-lactate) OR (Leukoencephalopathy NEAR/3 vanishing-white-matter) OR (Leukoencephalopathy-spondylometaphyseal-dysplasia NEAR/3 syndrom*) OR (Leukonychia-totalis) OR (Levic-Stefanovic-Nikolic NEAR/3 syndrom*) OR (Lhermitte-Duclos NEAR/3 diseas*) OR (Li-Fraumeni NEAR/3 syndrom*) OR (Lichtenstein NEAR/3 syndrom*) OR (Ligneous-conjunctivitis) OR (Limb-deficiencies-distal NEAR/3 micrognathia) OR (Limb-body-wall-complex) OR (Limb-girdle-muscular-dystroph*-type-1A) OR (Limb-girdle-muscular-dystroph*-type-1B) OR (Limb-girdle-muscular-dystroph*-type-1C) OR (Limb-girdle-muscular-dystroph*-type-1D) OR (Limb-girdle-muscular-dystroph*-type-1E) OR (Limb-girdle-muscular-dystroph*-type-1F) OR (Limb-girdle-muscular-dystroph*-type-1G) OR (Limb-girdle-muscular-dystroph*-type-1H) OR (Limb-girdle-muscular-dystroph*-type-2A) OR (Limb-girdle-muscular-dystroph*-type-2B) OR (Limb-girdle-muscular-dystroph*-type-2E) OR (Limb-girdle-muscular-dystroph*-type-2F) OR (Limb-girdle-muscular-dystroph*-type-2H) OR (Limb-girdle-muscular-dystroph*-type-2I) OR (Limb-girdle-muscular-dystroph*-type-2J) OR (Limb-girdle-muscular-dystroph*-type-2K) OR (Limb-girdle-muscular-dystroph*-type-2L) OR (Limb-girdle-muscular-dystroph*-type-2M) OR (Limb-girdle-muscular-dystroph*-type-2N) OR (Limb-girdle-muscular-dystroph*-type-2O) OR (Limb-girdle-muscular-dystroph*-type-2P-type-1A) OR (Limb-girdle-muscular-dystroph*-type-2Q) OR (Limb-girdle-muscular-dystroph*-type-2S) OR (Limb-girdle-muscular-dystroph*-type-2T) OR (Limb-girdle-muscular-dystroph* NEAR/3 type-2C) OR (Limb-girdle-muscular-dystroph* NEAR/3 type-2D) OR (Limb-girdle-muscular-dystroph* NEAR/3 type-2G) OR (Limb-mammary NEAR/3 syndrom*) OR (Lin-Gettig NEAR/3 syndrom*) OR (Linear NEAR/3 whorled-nevoid-hypermelanosis) OR (Linear-nevus-sebaceous NEAR/3 syndrom*) OR (LIPE-related-familial-partial-lipodystroph*) OR (Lipodystroph* NEAR/3 familial-partial NEAR/3 type-5) OR (Lipoic-acid-synthetase NEAR/3 deficien*) OR (Lipoid-proteinosis NEAR/3 Urbach NEAR/3 Wiethe) OR (Localized-junctional-epidermolysis-bullosa NEAR/3 non-Herlitz) OR (Long-QT NEAR/3 syndrom*-1) OR (Loose-anagen-hair NEAR/3 syndrom*) OR (Lopes-Gorlin NEAR/3 syndrom*) OR (Lowe-oculocerebrorenal NEAR/3 syndrom*) OR (Lowry-Maclean NEAR/3 syndrom*) OR (Lowry-Wood NEAR/3 syndrom*) OR (LRBA NEAR/3 deficien*) OR (Lubinsky NEAR/3 syndrom*) OR (Lucey-Driscoll NEAR/3 syndrom*) OR (Lujan NEAR/3 syndrom*) OR (Lung-agenesis) OR (Lymphangioleiomyomatosis) OR (Lymphedema NEAR/3 microcephaly NEAR/3 chorioretinopathy NEAR/3 syndrom*) OR (Lysinuric-protein-intolerance) OR (Mac-Dermot-Winter NEAR/3 syndrom*) OR (Macrocephaly NEAR/3 benign-familial) OR (Macrocephaly-short-stature-paraplegia NEAR/3 syndrom*) OR (Macrodactyly NEAR/3 fingers) OR (Macrodactyly NEAR/3 toes) OR (Macroepiphyseal-dysplasia NEAR/3 osteoporosis NEAR/3 wrinkled-skin NEAR/3 -aged-appearance) OR (Macroglossia) OR (Macrosomia NEAR/3 lethal-microphthalmia) OR (Macrozoospermia) OR (Macular-dystroph* NEAR/3 corneal-type-1) OR (Macules-hereditary-congenital-hypopigmented NEAR/3 hyperpigmented) OR (Madokoro-Ohdo-Sonoda NEAR/3 syndrom*) OR (Male-pseudohermaphroditism-due-to NEAR/3 defect*ive-LH-molecule) OR (Male-pseudohermaphroditism-intellectual-disability NEAR/3 syndrom* NEAR/3 Verloes) OR (Malignant-Atrophic-Papulosis) OR (Malignant-hyperthermia) OR (Malignant-hyperthermia-arthrogryposis-torticollis) OR (Malignant-migrating-partial-seizures NEAR/3 infancy) OR (Malonyl-CoA-decarboxylase NEAR/3 deficien*) OR (MAN1B1-CDG) OR (Mandibuloacral-dysplasia NEAR/3 type-A-lipodystroph*) OR (Mandibuloacral-dysplasia NEAR/3 type-B-lipodystroph*) OR (Mandibulofacial-dysostosis NEAR/3 microcephaly) OR (Manitoba-oculotrichoanal NEAR/3 syndrom*) OR (Mannosidosis NEAR/3 beta-A NEAR/3 lysosomal) OR (Manouvrier NEAR/3 syndrom*) OR (Marden-Walker NEAR/3 syndrom*) OR (Marfan NEAR/3 syndrom*) OR (Marfanoid-habitus-autosomal-recessive-intellectual-disability NEAR/3 syndrom*) OR (Marie-Unna-congenital-hypotrichosis) OR (Marinesco-Sjogren NEAR/3 syndrom*) OR (Marshall NEAR/3 syndrom*) OR (Marshall-Smith NEAR/3 syndrom*) OR (Martsolf NEAR/3 syndrom*) OR (Mastocytosis-cutaneous NEAR/3 short-stature-conductive-hearing-loss NEAR/3 microtia) OR (Maternal-hyperphenylalaninemia) OR (Maternally-inherited-diabetes NEAR/3 deafness) OR (Maturity-onset-diabetes NEAR/3 young) OR (Maturity-onset-diabetes NEAR/3 young NEAR/3 type-1) OR (Maturity-onset-diabetes NEAR/3 young NEAR/3 type-2) OR (Maturity-onset-diabetes NEAR/3 young NEAR/3 type-3) OR (Maturity-onset-diabetes NEAR/3 young NEAR/3 type-4) OR (Maturity-onset-diabetes NEAR/3 young NEAR/3 type-5) OR (Maturity-onset-diabetes NEAR/3 young NEAR/3 type-6) OR (Maturity-onset-diabetes NEAR/3 young NEAR/3 type-7) OR (Maturity-onset-diabetes NEAR/3 young NEAR/3 type-8) OR (Maturity-onset-diabetes NEAR/3 young NEAR/3 type-9) OR (Maxillonasal-dysplasia NEAR/3 Binder) OR (McCune-Albright NEAR/3 syndrom*) OR (McDonough NEAR/3 syndrom*) OR (McKusick-Kaufman NEAR/3 syndrom*) OR (McPherson-Clemens NEAR/3 syndrom*) OR (Meacham-Winn-Culler NEAR/3 syndrom*) OR (Meckel NEAR/3 syndrom*) OR (MECP2 NEAR/3 duplicat* NEAR/3 syndrom*) OR (Medeira-Dennis-Donnai NEAR/3 syndrom*) OR (Median-cleft NEAR/3 upper-lip NEAR/3 polyps NEAR/3 facial-skin NEAR/3 nasal-mucosa) OR (Median-nodule NEAR/3 upper-lip) OR (Medium-chain-acyl-coenzyme-A-dehydrogenase NEAR/3 deficien*) OR (Medrano-Roldan NEAR/3 syndrom*) OR (Medulloblastoma) OR (Meesmann-corneal-dystroph*) OR (Megacystis-microcolon-intestinal-hypoperistalsis NEAR/3 syndrom*) OR (Megaduodenum) OR (megacystis) OR (Megaepiphyseal-dwarfism) OR (Megalencephalic-leukoencephalopathy NEAR/3 subcortical-cysts) OR (Megalencephaly-Polymicrogyria-Polydactyly-Hydrocephalus NEAR/3 syndrom*) OR (Megalocornea---spherophakia---secondary-glaucoma) OR (Megalocornea-intellectual-disability NEAR/3 syndrom*) OR (Megarbane-Jalkh NEAR/3 syndrom*) OR (Megarbane NEAR/3 syndrom*) OR (Mehes NEAR/3 syndrom*) OR (MEHMO NEAR/3 syndrom*) OR (Mehta-Lewis-Patton NEAR/3 syndrom*) OR (Meier-Gorlin NEAR/3 syndrom*) OR (Meige NEAR/3 syndrom*) OR (Meleda NEAR/3 diseas*) OR (Melnick-Needles NEAR/3 syndrom*) OR (Melorheostosis) OR (Melorheostosis NEAR/3 osteopoikilosis) OR (Meningocele) OR (Menkes NEAR/3 diseas*) OR (Mesomelia-synostoses NEAR/3 syndrom*) OR (Mesomelic-dwarfism-cleft-palate-camptodactyly) OR (Mesomelic-dwarfism NEAR/3 hypoplastic-tibia NEAR/3 radius) OR (Mesomelic-dysplasia-Kantaputra) OR (Mesomelic-dysplasia-Savarirayan) OR (Metacarpals-4 NEAR/3 5-fusion) OR (Metachondromatosis) OR (Metaphyseal-acroscyphodysplasia) OR (Metaphyseal-chondrodysplasia-Schmid) OR (Metaphyseal-chondrodysplasia-Spahr) OR (Metaphyseal-dysostosis-intellectual-disability-conductive-deafness NEAR/3 syndrom*) OR (Metaphyseal-dysplasia-maxillary-hypoplasia-brachydactyly) OR (Metaphyseal-dysplasia-without-hypotrichosis) OR (Metaphyseal-undermodeling NEAR/3 spondylar-dysplasia NEAR/3 -overgrowth) OR (Metatropic-dysplasia) OR (Methimazole-antenatal-exposure) OR (Methionine-adenosyltransferase NEAR/3 deficien*) OR (Methylmalonic-aciduria NEAR/3 cblA) OR (Methylmalonic-aciduria NEAR/3 cblB) OR (Mevalonic-aciduria) OR (MGAT2-CDG-) OR (CDG-IIa) OR (Micro NEAR/3 syndrom*) OR (Microbrachycephaly-ptosis-cleft-lip) OR (Microcephalic-osteodysplastic-primordial-dwarfism-type-1) OR (Microcephalic-osteodysplastic-primordial-dwarfism-type-2) OR (Microcephalic-primordial-dwarfism-Toriello) OR (Microcephalic-primordial-dwarfism NEAR/3 Montreal) OR (Microcephaly-autosomal-dominant) OR (Microcephaly-brain NEAR/3 defect*-spasticity-hypernatremia) OR (Microcephaly-cervical-spine-fusion-anomalies) OR (Microcephaly-deafness NEAR/3 syndrom*) OR (Microcephaly-glomerulonephritis-Marfanoid-habitus) OR (Microcephaly-microcornea NEAR/3 syndrom*-Seemanova) OR (Microcephaly-micropenis-convulsions) OR (Microcephaly-microphthalmos-blindness) OR (Microcephaly-nonsyndromal) OR (Microcephaly NEAR/3 seizures NEAR/3 -developmental-delay) OR (Microcephaly-albinism-digital-anomalies NEAR/3 syndrom*) OR (Microcephaly-cardiomyopathy) OR (Microduplication-Xp11.22-p11.23 NEAR/3 syndrom*) OR (Microgastria-limb-reduction NEAR/3 defect*) OR (Microhydranencephaly) OR (Microphthalmia-associated NEAR/3 colobomatous-cyst) OR (Microphthalmia-syndromic-10) OR (Microphthalmia-syndromic-4) OR (Microphthalmia-syndromic-5) OR (Microphthalmia-syndromic-6) OR (Microphthalmia-syndromic-8) OR (Microphthalmia-syndromic-9) OR (Microphthalmia NEAR/3 linear-skin NEAR/3 defect*s NEAR/3 syndrom*) OR (Microsomia-hemifacial-radial NEAR/3 defect*s) OR (Microtia-eye-coloboma NEAR/3 imperforation NEAR/3 nasolacrimal-duct) OR (Microtia NEAR/3 meatal-atresia NEAR/3 conductive-deafness) OR (Microtia-Anotia) OR (Microvillus-inclusion NEAR/3 diseas*) OR (Midphalangeal-hair) OR (Mild-phenylketonuria) OR (Miller NEAR/3 syndrom*) OR (Miller-Dieker NEAR/3 syndrom*) OR (Milner-Khallouf-Gibson NEAR/3 syndrom*) OR (Minicore-myopathy NEAR/3 external-ophthalmoplegia) OR (Minicore-myopathy NEAR/3 antenatal-onset NEAR/3 arthrogryposis) OR (Mitochondrial-complex-I NEAR/3 deficien*) OR (Mitochondrial-complex-II NEAR/3 deficien*) OR (Mitochondrial-complex-III NEAR/3 deficien*) OR (Mitochondrial-DNA-depletion NEAR/3 syndrom* NEAR/3 encephalomyopathic-form NEAR/3 methylmalonic-aciduria) OR (Mitochondrial-DNA-associated-Leigh NEAR/3 syndrom*) OR (Mitochondrial-encephalomyopathy-lactic-acidosis NEAR/3 stroke-like-episodes) OR (Mitochondrial-genetic-disorders) OR (Mitochondrial-Membrane-Protein-Associated-Neurodegeneration) OR (Mitochondrial-myopathy NEAR/3 diabetes) OR (Mitochondrial-myopathy NEAR/3 lactic-acidosis) OR (Mitochondrial-neurogastrointestinal-encephalopathy NEAR/3 syndrom*) OR (Mitochondrial-trifunctional-protein NEAR/3 deficien*) OR (Mitral-atresia) OR (Mitral-regurgitation NEAR/3 conductive-deafness NEAR/3 -fusion NEAR/3 cervical-vertebrae NEAR/3 carpal NEAR/3 tarsal-bones) OR (Mitral-valve-prolapse NEAR/3 familial NEAR/3 autosomal-dominant) OR (Miyoshi-myopathy) OR (Moebius NEAR/3 syndrom*) OR (MOGS-CDG-) OR (CDG-IIb) OR (Mohr-Tranebjaerg NEAR/3 syndrom*) OR (Moloney NEAR/3 syndrom*) OR (Molybdenum-cofactor NEAR/3 deficien*) OR (MOMO NEAR/3 syndrom*) OR (Monilethrix) OR (Monoamine-oxidase-A NEAR/3 deficien*) OR (Morgagni-Stewart-Morel NEAR/3 syndrom*) OR (MORM NEAR/3 syndrom*) OR (Morning-glory NEAR/3 syndrom*) OR (Morse-Rawnsley-Sargent NEAR/3 syndrom*) OR (Mosaic-variegated-aneuploidy NEAR/3 syndrom*) OR (Mounier-Kuhn NEAR/3 syndrom*) OR (Mousa-Al-din-Al-Nassar NEAR/3 syndrom*) OR (Mowat-Wilson NEAR/3 syndrom*) OR (Moyamoya NEAR/3 diseas*) OR (MPDU1-CDG-) OR (CDG-If) OR (MPV17-related-hepatocerebral-mitochondrial-DNA-depletion NEAR/3 syndrom*) OR (Muckle-Wells NEAR/3 syndrom*) OR (Mucolipidosis-III-alpha) OR (Mucolipidosis-III-beta) OR (Mucolipidosis-type-4) OR (Mucopolysaccharidosis-type-III) OR (Mucopolysaccharidosis-type-IIIA) OR (Mucopolysaccharidosis-type-IIIB) OR (Mucopolysaccharidosis-type-IIIC) OR (Mucopolysaccharidosis-type-IIID) OR (Mucopolysaccharidosis-type-IV) OR (Mucopolysaccharidosis-type-IVA) OR (Mucopolysaccharidosis-type-VII) OR (Muenke NEAR/3 syndrom*) OR (Muir-Torre NEAR/3 syndrom*) OR (Mulibrey-Nanism) OR (Muller-Barth-Menger NEAR/3 syndrom*) OR (Multicentric-carpotarsal-osteolysis NEAR/3 syndrom*) OR (Multicystic-renal-dysplasia NEAR/3 bilateral) OR (Multiple-cafe-au-lait-spots) OR (Multiple-congenital-anomalies-hypotonia-seizures NEAR/3 syndrom*) OR (Multiple-congenital-anomalies-hypotonia-seizures NEAR/3 syndrom*-type-2) OR (Multiple-epiphyseal-dysplasia-1) OR (Multiple-epiphyseal-dysplasia-2) OR (Multiple-epiphyseal-dysplasia-3) OR (Multiple-epiphyseal-dysplasia-4) OR (Multiple-epiphyseal-dysplasia-5) OR (Multiple-fibrofolliculoma-familial) OR (Multiple-pterygium NEAR/3 syndrom*-Escobar) OR (Multiple-pterygium NEAR/3 syndrom*-lethal) OR (Multiple-pterygium NEAR/3 syndrom*-X-linked) OR (Multiple-sulfatase NEAR/3 deficien*) OR (Multiple-symmetric-lipomatosis) OR (Multiple-synostoses NEAR/3 syndrom*-1) OR (Multiple-synostoses NEAR/3 syndrom*-2) OR (Multiple-system-atrophy) OR (Mungan NEAR/3 syndrom*) OR (MURCS-association) OR (Muscle-eye-brain NEAR/3 diseas*) OR (Muscular-atrophy-ataxia-retinitis-pigmentosa NEAR/3 diabetes-mellitus) OR (Muscular-dystroph*-white-matter-spongiosis) OR (Muscular-dystroph* NEAR/3 congenital NEAR/3 megaconial) OR (Muscular-phosphorylase-kinase NEAR/3 deficien*) OR (Musculocontractural-Ehlers-Danlos NEAR/3 syndrom*) OR (MYD88 NEAR/3 deficien*) OR (Myelocerebellar-disorder) OR (MYH7-related-scapuloperoneal-myopathy) OR (Myhre NEAR/3 syndrom*) OR (Myoclonic-epilepsy NEAR/3 ragged-red-fibers) OR (Myoclonus-cerebellar-ataxia-deafness) OR (Myoclonus-dystonia) OR (Myoglobinuria-recurrent) OR (Myokymia NEAR/3 neonatal-epilepsy) OR (Myopathic-carnitine NEAR/3 deficien*) OR (Myopathy NEAR/3 extrapyramidal-signs) OR (Myosin-storage-myopathy) OR (Myotonia-congenita) OR (Myotonic-dystroph*-type-1) OR (Myotonic-dystroph*-type-2) OR (N NEAR/3 syndrom*) OR (N-acetylglutamate-synthase NEAR/3 deficien*) OR (Naegeli NEAR/3 syndrom*) OR (Nager-acrofacial-dysostosis) OR (Naguib-Richieri-Costa NEAR/3 syndrom*) OR (Nail-dysplasia NEAR/3 isolated-congenital) OR (Nail-patella NEAR/3 syndrom*) OR (Nakajo-Nishimura NEAR/3 syndrom*) OR (Nakajo NEAR/3 syndrom*) OR (Nance-Horan NEAR/3 syndrom*) OR (Nasodigitoacoustic NEAR/3 syndrom*) OR (Nathalie NEAR/3 syndrom*) OR (Naxos NEAR/3 diseas*) OR (NBIA NEAR/3 DYT NEAR/3 PARK-PLA2G6) OR (Neonatal-adrenoleukodystroph*) OR (Neonatal-intrahepatic-cholestasis-caused-by-citrin NEAR/3 deficien*) OR (Neonatal-Onset-Multisystem-Inflammatory NEAR/3 diseas*) OR (Neonatal-progeroid NEAR/3 syndrom*) OR (Neonatal-severe-hyperparathyroidism) OR (Nephrogenic-diabetes-insipidus) OR (Nephrogenic-diabetes-insipidus-intracranial-calcification-facial-dysmorphism NEAR/3 syndrom*) OR (Nephropathy NEAR/3 deafness NEAR/3 -hyperparathyroidism) OR (Nestor-guillermo-progeria NEAR/3 syndrom*) OR (Netherton NEAR/3 syndrom*) OR (Neu-Laxova NEAR/3 syndrom*) OR (Neurofaciodigitorenal NEAR/3 syndrom*) OR (Neuroferritinopathy) OR (Neurofibromatosis-type-2) OR (Neurofibromatosis-Noonan NEAR/3 syndrom*) OR (Neuronal-ceroid-lipofuscinosis-10) OR (Neuronal-ceroid-lipofuscinosis-2) OR (Neuronal-ceroid-lipofuscinosis-3) OR (Neuronal-ceroid-lipofuscinosis-5) OR (Neuronal-ceroid-lipofuscinosis-6) OR (Neuronal-ceroid-lipofuscinosis-7) OR (Neuronal-ceroid-lipofuscinosis-9) OR (Neuronal-intranuclear-inclusion NEAR/3 diseas*) OR (Neuropathy-ataxia-retinitis-pigmentosa NEAR/3 syndrom*) OR (Neuropathy NEAR/3 congenital NEAR/3 arthrogryposis-multiplex) OR (Neuropathy NEAR/3 distal-hereditary-motor NEAR/3 Jerash) OR (Neuropathy NEAR/3 hereditary-motor NEAR/3 sensory NEAR/3 Okinawa) OR (Neuropathy NEAR/3 hereditary-motor NEAR/3 sensory NEAR/3 Russe) OR (Neutral-lipid-storage NEAR/3 diseas* NEAR/3 myopathy) OR (Neutrophil-specific-granule NEAR/3 deficien*) OR (Nevoid-basal-cell-carcinoma NEAR/3 syndrom*) OR (Nevus-comedonicus NEAR/3 syndrom*) OR (Nguyen NEAR/3 syndrom*) OR (Nicolaides-Baraitser NEAR/3 syndrom*) OR (Niemann-Pick NEAR/3 diseas*-type-A) OR (Niemann-Pick NEAR/3 diseas*-type-B) OR (Niemann-Pick NEAR/3 diseas*-type-C1) OR (Niemann-Pick NEAR/3 diseas*-type-C2) OR (Nievergelt NEAR/3 syndrom*) OR (Night-blindness-skeletal-anomalies-dysmorphism NEAR/3 syndrom*) OR (Nijmegen-breakage NEAR/3 syndrom*) OR (Nonbullous-congenital-ichthyosiform-erythroderma) OR (Nonsyndromic-hereditary-sensorineural-hearing-loss) OR (Noonan-like NEAR/3 syndrom* NEAR/3 loose-anagen-hair) OR (Norrie NEAR/3 diseas*) OR (North-Carolina-macular-dystroph*) OR (Northern-epilepsy) OR (Not-otherwise-specified-3-MGA-uria) OR (Obesity-due-to-congenital-leptin NEAR/3 deficien*) OR (Occipital-horn NEAR/3 syndrom*) OR (Ochoa NEAR/3 syndrom*) OR (Ocular-albinism-type-1) OR (Oculo-skeletal-renal NEAR/3 syndrom*) OR (Oculoauriculofrontonasal NEAR/3 syndrom*) OR (Oculocerebral NEAR/3 syndrom* NEAR/3 hypopigmentation) OR (Oculocerebrocutaneous NEAR/3 syndrom*) OR (Oculocutaneous-albinism-type-1) OR (Oculocutaneous-albinism-type-1B) OR (Oculocutaneous-albinism-type-2) OR (Oculocutaneous-albinism-type-3) OR (Oculodentodigital-dysplasia) OR (Oculoectodermal NEAR/3 syndrom*) OR (Oculofaciocardiodental NEAR/3 syndrom*) OR (Oculomaxillofacial-dysostosis) OR (Oculomotor-apraxia-Cogan) OR (Oculopharyngeal-muscular-dystroph*) OR (Oculopharyngodistal-myopathy) OR (Oculorenocerebellar NEAR/3 syndrom*) OR (Odonto-onycho-dysplasia NEAR/3 alopecia) OR (Odontoma-dysphagia NEAR/3 syndrom*) OR (Odontomicronychial-dysplasia) OR (Odontoonychodermal-dysplasia) OR (Odontotrichomelic NEAR/3 syndrom*) OR (Oguchi NEAR/3 diseas*) OR (Okamoto NEAR/3 syndrom*) OR (Oligodactyly-tetramelic-postaxial) OR (Oligomeganephronic-renal-hypoplasia) OR (Oliver NEAR/3 syndrom*) OR (Olivopontocerebellar-atrophy-deafness) OR (Ollier NEAR/3 diseas*) OR (Olmsted NEAR/3 syndrom*) OR (Omenn NEAR/3 syndrom*) OR (Omodysplasia-1) OR (Omodysplasia-2) OR (Omphalocele-cleft-palate NEAR/3 syndrom*-lethal) OR (Omphalocele NEAR/3 exstrophy NEAR/3 cloaca NEAR/3 imperforate-anus NEAR/3 -spinal NEAR/3 defect*s-complex) OR (Omphalomesenteric-cyst) OR (Onychodystroph*-anonychia) OR (Onychotrichodysplasia NEAR/3 neutropenia) OR (OPA3 NEAR/3 defect*) OR (OPHN1 NEAR/3 syndrom*) OR (Opsismodysplasia) OR (Optic-atrophy-1) OR (Optic-atrophy-2) OR (Optic-atrophy-polyneuropathy-deafness) OR (Ornithine-transcarbamylase NEAR/3 deficien*) OR (Ornithine-translocase NEAR/3 deficien* NEAR/3 syndrom*) OR (Orofaciodigital NEAR/3 syndrom*-1) OR (Orofaciodigital NEAR/3 syndrom*-10) OR (Orofaciodigital NEAR/3 syndrom*-11) OR (Orofaciodigital NEAR/3 syndrom*-12) OR (Orofaciodigital NEAR/3 syndrom*-13) OR (Orofaciodigital NEAR/3 syndrom*-2) OR (Orofaciodigital NEAR/3 syndrom*-3) OR (Orofaciodigital NEAR/3 syndrom*-4) OR (Orofaciodigital NEAR/3 syndrom*-5) OR (Orofaciodigital NEAR/3 syndrom*-6) OR (Orofaciodigital NEAR/3 syndrom*-8) OR (Orofaciodigital NEAR/3 syndrom*-9) OR (Orofaciodigital NEAR/3 syndrom*s) OR (Orthostatic-intolerance-due-to-NET NEAR/3 deficien*) OR (Oslam NEAR/3 syndrom*) OR (OSMED NEAR/3 syndrom*) OR (Ossicular-Malformations NEAR/3 familial) OR (Osteoarthropathy NEAR/3 fingers-familial) OR (Osteodysplasia-familial-Anderson) OR (Osteofibrous-dysplasia) OR (Osteogenesis-imperfecta) OR (Osteogenesis-imperfecta-type-I) OR (Osteogenesis-imperfecta-type-II) OR (Osteogenesis-imperfecta-type-III) OR (Osteogenesis-imperfecta-type-IV) OR (Osteogenesis-imperfecta-type-IX) OR (Osteogenesis-imperfecta-type-V) OR (Osteogenesis-imperfecta-type-VI) OR (Osteogenesis-imperfecta-type-VII) OR (Osteogenesis-imperfecta-type-VIII) OR (Osteoglophonic-dysplasia) OR (Osteomesopyknosis) OR (Osteopathia-striata NEAR/3 cranial-sclerosis) OR (Osteopathia-striata NEAR/3 pigmentary-dermopathy-including-white-forelock) OR (Osteopenia NEAR/3 sparse-hair) OR (Osteopetrosis) OR (Osteopetrosis NEAR/3 infantile-neuroaxonal-dystroph*) OR (Osteopetrosis-autosomal-dominant-type-1) OR (Osteopetrosis-autosomal-dominant-type-2) OR (Osteopetrosis-autosomal-recessive-1) OR (Osteopetrosis-autosomal-recessive-2) OR (Osteopetrosis-autosomal-recessive-3) OR (Osteopetrosis-autosomal-recessive-4) OR (Osteopetrosis-autosomal-recessive-5) OR (Osteopetrosis-autosomal-recessive-6) OR (Osteopetrosis-autosomal-recessive-7) OR (Osteopoikilosis NEAR/3 dacryocystitis) OR (Osteoporosis-oculocutaneous-hypopigmentation NEAR/3 syndrom*) OR (Osteoporosis-pseudoglioma NEAR/3 syndrom*) OR (Ostium-secundum-atrial-septal NEAR/3 defect*) OR (Oto-palato-digital NEAR/3 syndrom*-type-1) OR (Oto-palato-digital NEAR/3 syndrom*-type-2) OR (Otodental-dysplasia) OR (Otofaciocervical NEAR/3 syndrom*) OR (Otoonychoperoneal NEAR/3 syndrom*) OR (Ouvrier-Billson NEAR/3 syndrom*) OR (Pachydermoperiostosis) OR (Pachygyria) OR (Pachygyria NEAR/3 frontotemporal) OR (Pachygyria-intellectual-disability-epilepsy NEAR/3 syndrom*) OR (Pachyonychia-congenita) OR (Pacman-dysplasia) OR (PACS1-related NEAR/3 syndrom*) OR (PAGOD NEAR/3 syndrom*) OR (Pagon-Stephan NEAR/3 syndrom*) OR (Paine NEAR/3 syndrom*) OR (Palant-cleft-palate NEAR/3 syndrom*) OR (Palatopharyngeal-incompetence) OR (Pallidopyramidal NEAR/3 syndrom*) OR (Pallister-W NEAR/3 syndrom*) OR (Pallister-Hall NEAR/3 syndrom*) OR (Palmer-Pagon NEAR/3 syndrom*) OR (Palmoplantar-keratoderma-sclerodactyly NEAR/3 syndrom*) OR (Panhypopituitarism-X-linked) OR (Panostotic-fibrous-dysplasia) OR (Papillary-renal-cell-carcinoma) OR (Papillon-Lefevre NEAR/3 syndrom*) OR (Paraganglioma NEAR/3 gastric-stromal-sarcoma) OR (Paramyotonia-congenita) OR (PARC NEAR/3 syndrom*) OR (Parkinson NEAR/3 diseas*-type-9) OR (Paroxysmal-exertion-induced-dyskinesia) OR (Paroxysmal-extreme-pain-disorder) OR (Paroxysmal-kinesigenic-choreoathetosis) OR (Paroxysmal-ventricular-fibrillation) OR (Paroxysomal-nonkinesigenic-dyskinesia) OR (Partial-androgen-insensitivity NEAR/3 syndrom*) OR (Partington NEAR/3 syndrom*) OR (PASLI NEAR/3 diseas*) OR (Paternal-uniparental-disomy NEAR/3 chromosome-14) OR (Patterson-Stevenson-Fontaine NEAR/3 syndrom*) OR (PCDH19-related-female-limited-epilepsy) OR (Pectus-carinatum) OR (Pelger-Huet-anomaly) OR (Pelvic-dysplasia-arthrogryposis NEAR/3 lower-limbs) OR (Pendred NEAR/3 syndrom*) OR (Pentalogy NEAR/3 Cantrell) OR (Pentosuria) OR (Periodic-fever NEAR/3 aphthous-stomatitis NEAR/3 pharyngitis NEAR/3 adenitis) OR (Periodontal-Ehlers-Danlos NEAR/3 syndrom*) OR (Peripheral-resistance-to-thyroid-hormones) OR (Periventricular-heterotopia) OR (Perlman NEAR/3 syndrom*) OR (Permanent-neonatal-diabetes-mellitus) OR (Peroxisomal-biogenesis-disorders) OR (Perrault NEAR/3 syndrom*) OR (Perry NEAR/3 syndrom*) OR (Persistent-Mullerian-duct NEAR/3 syndrom*) OR (Peters-plus NEAR/3 syndrom*) OR (Peutz-Jeghers NEAR/3 syndrom*) OR (Pfeiffer-Mayer NEAR/3 syndrom*) OR (Pfeiffer-Palm-Teller NEAR/3 syndrom*) OR (Pfeiffer NEAR/3 syndrom*) OR (Pfeiffer-Tietze-Welte NEAR/3 syndrom*) OR (Pfeiffer-type-cardiocranial NEAR/3 syndrom*) OR (PGM1-CDG) OR (PGM3-CDG) OR (PHAVER NEAR/3 syndrom*) OR (Phenobarbital-antenatal-exposure) OR (Phocomelia-ectrodactyly-deafness-sinus-arrhythmia) OR (Phosphoglycerate-mutase NEAR/3 deficien*) OR (Phosphoserine-aminotransferase NEAR/3 deficien*) OR (Piebaldism) OR (Pierre-Robin-sequence) OR (Pierre-Robin-sequence NEAR/3 pectus-excavatum NEAR/3 rib NEAR/3 scapular-anomalies) OR (Pierson NEAR/3 syndrom*) OR (Pili-annulati) OR (Pili-torti) OR (Pili-torti-developmental-delay-neurological-abnormalities) OR (Pillay NEAR/3 syndrom*) OR (Pilodental-dysplasia NEAR/3 refractive-errors) OR (Pinheiro-Freire-Maia-Miranda NEAR/3 syndrom*) OR (Pitt-Hopkins NEAR/3 syndrom*) OR (Pitt-Hopkins-like NEAR/3 syndrom*) OR (Pituitary-dwarfism NEAR/3 large-sella-turcica) OR (Pituitary-hormone NEAR/3 deficien* NEAR/3 combined-3) OR (Pituitary-hormone NEAR/3 deficien* NEAR/3 combined-4) OR (Pituitary-stalk-interruption NEAR/3 syndrom*) OR (Pityriasis-rubra-pilaris) OR (Plagiocephaly) OR (Platyspondylic-lethal-skeletal-dysplasia-Torrance) OR (PMM2-CDG-) OR (CDG-Ia) OR (Poland NEAR/3 syndrom*) OR (POLR3-Related-Leukodystroph*) OR (Polycystic-kidneys NEAR/3 severe-infantile NEAR/3 tuberous-sclerosis) OR (Polycystic-lipomembranous-osteodysplasia NEAR/3 sclerosing-leukoencephalopathy) OR (Polycystic-liver NEAR/3 diseas*) OR (Polydactyly) OR (Polydactyly-myopia NEAR/3 syndrom*) OR (Polyneuropathy-intellectual-disability-acromicria-premature-menopause NEAR/3 syndrom*) OR (Polyosteolysis NEAR/3 hyperostosis NEAR/3 syndrom*) OR (Polyostotic-osteolytic-dysplasia NEAR/3 hereditary-expansile) OR (Polysyndactyly-cardiac-malformation) OR (Pontine-tegmental-cap-dysplasia) OR (Pontocerebellar-hypoplasia-type-1) OR (Pontocerebellar-hypoplasia-type-2) OR (Pontocerebellar-hypoplasia-type-3) OR (Pontocerebellar-hypoplasia-type-4) OR (Pontocerebellar-hypoplasia-type-5) OR (Pontocerebellar-hypoplasia-type-6) OR (Popliteal-pterygium NEAR/3 syndrom*) OR (Popliteal-pterygium NEAR/3 syndrom* NEAR/3 Bartsocas-Papas) OR (Porokeratosis NEAR/3 Mibelli) OR (Porokeratosis NEAR/3 disseminated-superficial-actinic-2) OR (Porphyria-cutanea-tarda) OR (Posterior-column-ataxia NEAR/3 retinitis-pigmentosa) OR (Postnatal-progressive-microcephaly NEAR/3 seizures NEAR/3 -brain-atrophy) OR (Potassium-aggravated-myotonia) OR (Potato-nose) OR (Potocki-Lupski NEAR/3 syndrom*) OR (Potter-sequence) OR (PPM-X NEAR/3 syndrom*) OR (Prader-Willi-habitus NEAR/3 osteopenia NEAR/3 -camptodactyly) OR (Prader-Willi NEAR/3 syndrom*) OR (Preaxial NEAR/3 deficien* NEAR/3 postaxial-polydactyly NEAR/3 hypospadias) OR (Preaxial-polydactyly-type-1) OR (Preaxial-polydactyly-type-2) OR (Preaxial-polydactyly-type-3) OR (Preaxial-polydactyly-type-4) OR (Pretibial-epidermolysis-bullosa) OR (Primary-basilar-impression) OR (Primary-carnitine NEAR/3 deficien*) OR (Primary-ciliary-dyskinesia) OR (Primary-Familial-Brain-Calcification) OR (Primary-hyperoxaluria-type-1) OR (Primary-hyperoxaluria-type-2) OR (Primary-hypomagnesemia NEAR/3 secondary-hypocalcemia) OR (Primary-lateral-sclerosis) OR (Primary-open-angle-glaucoma-juvenile-onset-1) OR (Primary-pigmented-nodular-adrenocortical NEAR/3 diseas*) OR (Primrose NEAR/3 syndrom*) OR (Progeria) OR (Progeroid-short-stature NEAR/3 pigmented-nevi) OR (Progeroid NEAR/3 syndrom*-Petty) OR (Prognathism-mandibular) OR (Progressive-bifocal-chorioretinal-atrophy) OR (Progressive-deafness NEAR/3 stapes-fixation) OR (Progressive-external-ophthalmoplegia NEAR/3 autosomal-recessive-1) OR (Progressive-familial-heart-block-type-1A) OR (Progressive-familial-heart-block-type-1B) OR (Progressive-familial-heart-block-type-2) OR (Progressive-familial-intrahepatic-cholestasis-1) OR (Progressive-familial-intrahepatic-cholestasis-type-2) OR (Progressive-familial-intrahepatic-cholestasis-type-3) OR (Progressive-non-fluent-aphasia) OR (Progressive-osseous-heteroplasia) OR (Progressive-pseudorheumatoid-dysplasia) OR (Proopiomelanocortin NEAR/3 deficien*) OR (Propionic-acidemia) OR (Proteus-like NEAR/3 syndrom*) OR (Proud NEAR/3 syndrom*) OR (Proximal-symphalangism) OR (Prune-belly NEAR/3 syndrom*) OR (Pseudo-Pelger-Huet-anomaly) OR (Pseudoachondroplasia) OR (Pseudoaminopterin NEAR/3 syndrom*) OR (Pseudocholinesterase NEAR/3 deficien*) OR (Pseudodiastrophic-dysplasia) OR (Pseudohypoaldosteronism-type-2) OR (Pseudohypoparathyroidism-type-1A) OR (Pseudohypoparathyroidism-type-1B) OR (Pseudohypoparathyroidism-type-1C) OR (Pseudohypoparathyroidism-type-2) OR (Pseudoneonatal-adrenoleukodystroph*) OR (Pseudoprogeria NEAR/3 syndrom*) OR (Pseudopseudohypoparathyroidism) OR (Pseudotrisomy-13 NEAR/3 syndrom*) OR (Pseudoxanthoma-elasticum NEAR/3 forme-fruste) OR (Ptosis-strabismus-ectopic-pupils) OR (Pulmonary-alveolar-microlithiasis) OR (Pulmonary-atresia NEAR/3 ventricular-septal NEAR/3 defect*) OR (Pulmonary-venoocclusive NEAR/3 diseas*) OR (Punctate-palmoplantar-keratoderma-type-2) OR (Punctate-palmoplantar-keratoderma-type-I) OR (Purine-nucleoside-phosphorylase NEAR/3 deficien*) OR (Pustulosis-palmaris-et-plantaris) OR (Pycnodysostosis) OR (Pyknoachondrogenesis) OR (Pyle NEAR/3 diseas*) OR (Pyogenic-arthritis NEAR/3 pyoderma-gangrenosum NEAR/3 acne) OR (Pyramidal-molars-abnormal-upper-lip NEAR/3 syndrom*) OR (Pyridoxal-5-phosphate-dependent-epilepsy) OR (Pyridoxine-dependent-epilepsy) OR (Pyruvate-dehydrogenase-phosphatase NEAR/3 deficien*) OR (Qazi-Markouizos NEAR/3 syndrom*) OR (Rabson-Mendenhall NEAR/3 syndrom*) OR (Radial NEAR/3 defect*-Robin-sequence) OR (Radial-ray-agenesis) OR (Radial-ray-hypoplasia-choanal-atresia) OR (Radio-renal NEAR/3 syndrom*) OR (Radioulnar-synostosis-microcephaly-scoliosis NEAR/3 syndrom*) OR (Radius-absent-anogenital-anomalies) OR (Raine NEAR/3 syndrom*) OR (Ramon NEAR/3 syndrom*) OR (Ramos-Arroyo-Clark NEAR/3 syndrom*) OR (Rapadilino NEAR/3 syndrom*) OR (Rapid-onset-dystonia-parkinsonism) OR (Rasmussen-Johnsen-Thomsen NEAR/3 syndrom*) OR (Reardon-Wilson-Cavanagh NEAR/3 syndrom*) OR (Recessive-dystrophic-epidermolysis-bullosa-generalized-other) OR (Reducing-body-myopathy) OR (Refsum NEAR/3 diseas*) OR (Refsum NEAR/3 diseas* NEAR/3 infantile-form) OR (Renal-agenesis) OR (Renal-coloboma NEAR/3 syndrom*) OR (Renal-dysplasia NEAR/3 retinal-pigmentary-dystroph* NEAR/3 cerebellar-ataxia NEAR/3 skeletal-dysplasia) OR (Renal-dysplasia-limb NEAR/3 defect*s NEAR/3 syndrom*) OR (Renal-glycosuria) OR (Renal-hypomagnesemia-2) OR (Renal-hypomagnesemia-6) OR (Renal-hypouricemia) OR (Renal-tubular-acidosis NEAR/3 deafness) OR (Renal-tubulopathy NEAR/3 diabetes-mellitus NEAR/3 -cerebellar-ataxia-due-to NEAR/3 duplicat* NEAR/3 mitochondrial-DNA) OR (Renier-Gabreels-Jasper NEAR/3 syndrom*) OR (Renpenning NEAR/3 syndrom*) OR (Reticular-dysgenesis) OR (Retinal-arterial-macroaneurysm NEAR/3 supravalvular-pulmonic-stenosis) OR (Retinal-cone-dystroph*-1) OR (Retinal-degeneration NEAR/3 nanophthalmos NEAR/3 cystic-macular-degeneration NEAR/3 -angle-closure-glaucoma) OR (Retinal-vasculopathy NEAR/3 cerebral-leukodystroph* NEAR/3 systemic-manifestations) OR (Retinitis-pigmentosa) OR (Retinitis-pigmentosa-intellectual-disability-deafness-hypogonadism NEAR/3 syndrom*) OR (Rett NEAR/3 syndrom*) OR (RFT1-CDG-) OR (CDG-In) OR (Rhizomelic-chondrodysplasia-punctata-type-1) OR (Rhizomelic-chondrodysplasia-punctata-type-2) OR (Rhizomelic-dysplasia-Patterson-Lowry) OR (Rhizomelic NEAR/3 syndrom*) OR (RHYNS NEAR/3 syndrom*) OR (Riboflavin-transporter NEAR/3 deficien*) OR (Richards-Rundle NEAR/3 syndrom*) OR (Richieri-Costa-Da-Silva NEAR/3 syndrom*) OR (Richieri-Costa-Pereira NEAR/3 syndrom*) OR (Right-ventricle-hypoplasia) OR (Rigid-spine NEAR/3 syndrom*) OR (Rippling-muscle NEAR/3 diseas*) OR (RNAse-T2-deficient-leukoencephalopathy) OR (Roberts NEAR/3 syndrom*) OR (Robinow NEAR/3 syndrom*) OR (Roch-Leri-mesosomatous-lipomatosis) OR (Rodrigues-blindness) OR (Roifman NEAR/3 syndrom*) OR (Rokitansky-sequence) OR (Rokitansky-Aschoff-sinuses NEAR/3 gallbladder) OR (Rombo NEAR/3 syndrom*) OR (Rommen-Mueller-Sybert NEAR/3 syndrom*) OR (Rothmund-Thomson NEAR/3 syndrom*) OR (Roussy-Levy NEAR/3 syndrom*) OR (Rozin-Hertz-Goodman NEAR/3 syndrom*) OR (RRM2B-related-mitochondrial-DNA-depletion NEAR/3 syndrom*) OR (Rud NEAR/3 syndrom*) OR (Russell-Silver NEAR/3 syndrom*) OR (Rutherfurd NEAR/3 syndrom*) OR (Ruvalcaba NEAR/3 syndrom*) OR (Ruzicka-Goerz-Anton NEAR/3 syndrom*) OR (Sabinas-brittle-hair NEAR/3 syndrom*) OR (Saccharopinuria) OR (Sacral-hemangiomas-multiple-congenital-abnormalities) OR (Sacral-meningocele-conotruncal-heart NEAR/3 defect*s) OR (Saethre-Chotzen NEAR/3 syndrom*) OR (Saito-Kuba-Tsuruta NEAR/3 syndrom*) OR (Sakoda-complex) OR (Salcedo NEAR/3 syndrom*) OR (Salla NEAR/3 diseas*) OR (Sarcosinemia) OR (Satoyoshi NEAR/3 syndrom*) OR (Saul-Wilkes-Stevenson NEAR/3 syndrom*) OR (Say-Barber-Miller NEAR/3 syndrom*) OR (Say-Meyer NEAR/3 syndrom*) OR (Say NEAR/3 syndrom*) OR (Say-Field-Coldwell NEAR/3 syndrom*) OR (Scalp NEAR/3 defect*s-postaxial-polydactyly) OR (Scalp-ear-nipple NEAR/3 syndrom*) OR (Scapuloperoneal NEAR/3 syndrom* NEAR/3 neurogenic NEAR/3 Kaeser) OR (SCARF NEAR/3 syndrom*) OR (Schaaf-Yang NEAR/3 syndrom*) OR (Schaap-Taylor-Baraitser NEAR/3 syndrom*) OR (Schaefer-Stein-Oshman NEAR/3 syndrom*) OR (Scheie NEAR/3 syndrom*) OR (Scheuermann NEAR/3 diseas*) OR (Schimke-immunoosseous-dysplasia) OR (Schindler NEAR/3 diseas*-type-1) OR (Schinzel-Giedion NEAR/3 syndrom*) OR (Schinzel-type-phocomelia) OR (Schisis-association) OR (Schizencephaly) OR (Schneckenbecken-dysplasia) OR (Scholte NEAR/3 syndrom*) OR (Schrander-Stumpel-Theunissen-Hulsmans NEAR/3 syndrom*) OR (Schwannomatosis) OR (Schwartz-Jampel NEAR/3 syndrom*) OR (Sclerosteosis) OR (SCOT NEAR/3 deficien*) OR (Scott-Bryant-Graham NEAR/3 syndrom*) OR (Sea-Blue-histiocytosis) OR (Seaver-Cassidy NEAR/3 syndrom*) OR (Sebaceous-gland-hyperplasia NEAR/3 familial-presenile) OR (Seckel-like NEAR/3 syndrom*-Majoor-Krakauer) OR (Seckel NEAR/3 syndrom*) OR (Segmentation NEAR/3 syndrom*-1) OR (Selective-IgM NEAR/3 deficien*) OR (Semantic-dementia) OR (Sengers NEAR/3 syndrom*) OR (Senior-Loken NEAR/3 syndrom*) OR (Sensory-ataxic-neuropathy NEAR/3 dysarthria NEAR/3 -ophthalmoparesis) OR (Sepiapterin-reductase NEAR/3 deficien*) OR (Septo-optic-dysplasia-spectrum) OR (Seres-Santamaria-Arimany-Muniz NEAR/3 syndrom*) OR (SERKAL NEAR/3 syndrom*) OR (SeSAME NEAR/3 syndrom*) OR (SETBP1-disorder) OR (Severe-achondroplasia NEAR/3 developmental-delay NEAR/3 acanthosis-nigricans) OR (Severe-combined-immunodeficiency) OR (Severe-combined-immunodeficiency-due-to-complete-RAG NEAR/3 deficien*) OR (Severe-congenital-nemaline-myopathy) OR (Severe-congenital-neutropenia-X-linked) OR (Severe-generalized-recessive-dystrophic-epidermolysis-bullosa) OR (Severe-intellectual-disability-progressive-spastic-diplegia NEAR/3 syndrom*) OR (Severe-X-linked-intellectual-disability NEAR/3 Gustavson) OR (Sheldon-Hall NEAR/3 syndrom*) OR (Short-rib-polydactyly NEAR/3 syndrom*-type-3) OR (Short-rib-polydactyly NEAR/3 syndrom*-type-1) OR (Short-rib-polydactyly NEAR/3 syndrom*-type-4) OR (Short-rib-polydactyly NEAR/3 syndrom* NEAR/3 Majewski) OR (Short-stature-deafness-neutrophil-dysfunction) OR (Short-stature NEAR/3 syndrom* NEAR/3 Brussels) OR (Short-stature-wormian-bones-dextrocardia) OR (Short-stature-craniofacial-anomalies-genital-hypoplasia NEAR/3 syndrom*) OR (SHORT NEAR/3 syndrom*) OR (Short-chain-acyl-CoA-dehydrogenase NEAR/3 deficien*) OR (Short-limb-skeletal-dysplasia NEAR/3 severe-combined-immunodeficiency) OR (Shprintzen-omphalocele NEAR/3 syndrom*) OR (Shprintzen-Goldberg-craniosynostosis NEAR/3 syndrom*) OR (Sialidosis-type-I) OR (Sialidosis NEAR/3 type-II) OR (Sialuria NEAR/3 French) OR (Siegler-Brewer-Carey NEAR/3 syndrom*) OR (Silengo-Lerone-Pelizza NEAR/3 syndrom*) OR (Sillence NEAR/3 syndrom*) OR (Simosa-cranio-facial NEAR/3 syndrom*) OR (Simpson-Golabi-Behmel NEAR/3 syndrom*) OR (Single-upper-central-incisor) OR (Singleton-Merten NEAR/3 syndrom*) OR (Sirenomelia) OR (Sitosterolemia) OR (Situs-inversus) OR (Situs-inversus-totalis NEAR/3 cystic-dysplasia NEAR/3 kidneys NEAR/3 pancreas) OR (Sjogren-Larsson NEAR/3 syndrom*) OR (Skeletal-dysplasia NEAR/3 San-Diego) OR (Skin-fragility-woolly-hair-palmoplantar-keratoderma NEAR/3 syndrom*) OR (SLC35A1-CDG-) OR (CDG-IIf) OR (SLC35A2-CDG) OR (SLC35C1-CDG-) OR (CDG-IIc) OR (Small-patella NEAR/3 syndrom*) OR (Smith-McCort-dysplasia) OR (Smith-Fineman-Myers NEAR/3 syndrom*) OR (Smith-Lemli-Opitz NEAR/3 syndrom*) OR (Snowflake-vitreoretinal-degeneration) OR (Snyder-Robinson NEAR/3 syndrom*) OR (Sonoda NEAR/3 syndrom*) OR (Sotos NEAR/3 syndrom*) OR (Spasmodic-dysphonia) OR (Spastic-ataxia-Charlevoix-Saguenay) OR (Spastic-paraplegia-1) OR (Spastic-paraplegia-10) OR (Spastic-paraplegia-11) OR (Spastic-paraplegia-12) OR (Spastic-paraplegia-13) OR (Spastic-paraplegia-14) OR (Spastic-paraplegia-15) OR (Spastic-paraplegia-16) OR (Spastic-paraplegia-17) OR (Spastic-paraplegia-18) OR (Spastic-paraplegia-19) OR (Spastic-paraplegia-2) OR (Spastic-paraplegia-23) OR (Spastic-paraplegia-24) OR (Spastic-paraplegia-25) OR (Spastic-paraplegia-26) OR (Spastic-paraplegia-29) OR (Spastic-paraplegia-3) OR (Spastic-paraplegia-31) OR (Spastic-paraplegia-32) OR (Spastic-paraplegia-4) OR (Spastic-paraplegia-51) OR (Spastic-paraplegia-5A) OR (Spastic-paraplegia-6) OR (Spastic-paraplegia-7) OR (Spastic-paraplegia-8) OR (Spastic-paraplegia-9) OR (Spastic-paraplegia-facial-cutaneous-lesions) OR (Spastic-paraplegia-epilepsy-intellectual-disability NEAR/3 syndrom*) OR (Spastic-paraplegia-glaucoma-intellectual-disability NEAR/3 syndrom*) OR (Spastic-tetraplegia-retinitis-pigmentosa-intellectual-disability NEAR/3 syndrom*) OR (Spastic-tetraplegia-thin-corpus-callosum-progressive-postnatal-microcephaly NEAR/3 syndrom*) OR (Specific-antibody NEAR/3 deficien*) OR (Spina-bifida) OR (Spinal-atrophy-ophthalmoplegia-pyramidal NEAR/3 syndrom*) OR (Spinal-muscular-atrophy-1) OR (Spinal-muscular-atrophy-type-2) OR (Spinal-muscular-atrophy-type-3) OR (Spinal-muscular-atrophy-progressive-myoclonic-epilepsy NEAR/3 syndrom*) OR (Spinocerebellar-ataxia-1) OR (Spinocerebellar-ataxia-10) OR (Spinocerebellar-ataxia-11) OR (Spinocerebellar-ataxia-12) OR (Spinocerebellar-ataxia-13) OR (Spinocerebellar-ataxia-14) OR (Spinocerebellar-ataxia-15) OR (Spinocerebellar-ataxia-17) OR (Spinocerebellar-ataxia-18) OR (Spinocerebellar-ataxia-19 NEAR/3 22) OR (Spinocerebellar-ataxia-2) OR (Spinocerebellar-ataxia-20) OR (Spinocerebellar-ataxia-21) OR (Spinocerebellar-ataxia-23) OR (Spinocerebellar-ataxia-25) OR (Spinocerebellar-ataxia-26) OR (Spinocerebellar-ataxia-27) OR (Spinocerebellar-ataxia-28) OR (Spinocerebellar-ataxia-29) OR (Spinocerebellar-ataxia-31) OR (Spinocerebellar-ataxia-34) OR (Spinocerebellar-ataxia-4) OR (Spinocerebellar-ataxia-5) OR (Spinocerebellar-ataxia-7) OR (Spinocerebellar-ataxia-8) OR (Spinocerebellar-ataxia-autosomal-recessive-3) OR (Spinocerebellar-ataxia-autosomal-recessive-4) OR (Spinocerebellar-ataxia-autosomal-recessive-5) OR (Spinocerebellar-ataxia-autosomal-recessive-7) OR (Spinocerebellar-ataxia-autosomal-recessive-8) OR (Spinocerebellar-ataxia-type-6) OR (Spinocerebellar-ataxia NEAR/3 axonal-neuropathy-type-1) OR (Spinocerebellar-ataxia NEAR/3 dysmorphism) OR (Spinocerebellar-ataxia-X-linked-type-3) OR (Spinocerebellar-ataxia-X-linked-type-4) OR (Spinocerebellar-degeneration NEAR/3 corneal-dystroph*) OR (Splenogonadal-fusion-limb NEAR/3 defect*s-micrognatia) OR (Split-hand-foot-malformation) OR (Split-hand-split-foot-nystagmus) OR (Split-hand-urinary-anomalies-spina-bifida) OR (Split-spinal-cord-malformation) OR (Spondylocamptodactyly) OR (Spondylocarpotarsal-synostosis NEAR/3 syndrom*) OR (Spondylocostal-dysostosis-1) OR (Spondylocostal-dysostosis-2) OR (Spondylocostal-dysostosis-3) OR (Spondylocostal-dysostosis-4) OR (Spondylocostal-dysostosis-5) OR (Spondylocostal-dysostosis-6) OR (Spondylodysplastic-Ehlers-Danlos NEAR/3 syndrom*) OR (Spondyloenchondrodysplasia NEAR/3 immune-dysregulation) OR (Spondyloepimetaphyseal-dysplasia-Genevieve) OR (Spondyloepimetaphyseal-dysplasia-joint-laxity) OR (Spondyloepimetaphyseal-dysplasia-Matrilin-3-related) OR (Spondyloepimetaphyseal-dysplasia-micromelic) OR (Spondyloepimetaphyseal-dysplasia-Missouri) OR (Spondyloepimetaphyseal-dysplasia-Shohat) OR (Spondyloepimetaphyseal-dysplasia-Sponastrime) OR (Spondyloepimetaphyseal-dysplasia-Strudwick) OR (Spondyloepimetaphyseal-dysplasia NEAR/3 hypotrichosis) OR (Spondyloepimetaphyseal-dysplasia NEAR/3 multiple-dislocations) OR (Spondyloepimetaphyseal-dysplasia-X-linked) OR (Spondyloepimetaphyseal-dysplasia NEAR/3 Aggrecan) OR (Spondyloepiphyseal-dysplasia-congenita) OR (Spondyloepiphyseal-dysplasia-Maroteaux) OR (Spondyloepiphyseal-dysplasia-tarda-X-linked) OR (Spondyloepiphyseal-dysplasia-brachydactyly NEAR/3 distinctive-speech) OR (Spondylometaepiphyseal-dysplasia-short-limb-hand) OR (Spondylometaphyseal-dysplasia-Algerian) OR (Spondylometaphyseal-dysplasia-corner-fracture) OR (Spondylometaphyseal-dysplasia-East-African) OR (Spondylometaphyseal-dysplasia-Sedaghatian) OR (Spondylometaphyseal-dysplasia-type-A4) OR (Spondylometaphyseal-dysplasia NEAR/3 bowed-forearms NEAR/3 facial-dysmorphism) OR (Spondylometaphyseal-dysplasia NEAR/3 cone-rod-dystroph*) OR (Spondylometaphyseal-dysplasia NEAR/3 dentinogenesis-imperfecta) OR (Spondylometaphyseal-dysplasia-X-linked) OR (Spondylometaphyseal-dysplasia NEAR/3 Kozlowski) OR (Spondyloperipheral-dysplasia) OR (Spondylospinal-thoracic-dysostosis) OR (Spondylothoracic-dysostosis) OR (Sprengel-deformity) OR (SRD5A3-CDG-) OR (CDG-Iq) OR (SSR4-CDG) OR (STAC3-Disorder) OR (Stalker-Chitayat NEAR/3 syndrom*) OR (STAR NEAR/3 syndrom*) OR (Stargardt NEAR/3 diseas*) OR (Steatocystoma-multiplex) OR (Steatocystoma-multiplex NEAR/3 natal-teeth) OR (Steinfeld NEAR/3 syndrom*) OR (Sternal-cleft) OR (Stickler NEAR/3 syndrom*) OR (Stiff-person NEAR/3 syndrom*) OR (Stiff-skin NEAR/3 syndrom*) OR (Stocco-dos-Santos NEAR/3 syndrom*) OR (Stoll-Alembik-Finck NEAR/3 syndrom*) OR (Striatonigral-degeneration-infantile) OR (Stuve-Wiedemann NEAR/3 syndrom*) OR (Subaortic-stenosis-short-stature NEAR/3 syndrom*) OR (Subcortical-band-heterotopia) OR (Succinic-semialdehyde-dehydrogenase NEAR/3 deficien*) OR (Sudden-infant-death NEAR/3 dysgenesis NEAR/3 testes NEAR/3 syndrom*) OR (Sugarman-brachydactyly) OR (Symphalangism NEAR/3 multiple-anomalies NEAR/3 hands NEAR/3 feet) OR (Syndactyly-Cenani-Lenz) OR (Syndactyly-type-1) OR (Syndactyly-type-3) OR (Syndactyly-type-5) OR (Syndactyly-type-9) OR (Syndactyly-polydactyly-earlobe NEAR/3 syndrom*) OR (Syndromic-microphthalmia NEAR/3 type-3) OR (Syndromic-X-linked-intellectual-disability-7) OR (Syngnathia-cleft-palate) OR (Syngnathia-multiple-anomalies) OR (Synovial-chondromatosis NEAR/3 familial NEAR/3 dwarfism) OR (Syringomyelia) OR (T-cell-immunodeficiency NEAR/3 congenital-alopecia NEAR/3 nail-dystroph*) OR (Tabatznik NEAR/3 syndrom*) OR (Talo-patello-scaphoid-osteolysis NEAR/3 synovitis NEAR/3 -short-fourth-metacarpals) OR (Talonavicular-coalition) OR (Tangier NEAR/3 diseas*) OR (TANGO2-Related-Metabolic-Encephalopathy NEAR/3 Arrhythmias) OR (TARP NEAR/3 syndrom*) OR (Tarsal-carpal-coalition NEAR/3 syndrom*) OR (Taurodontia NEAR/3 absent-teeth NEAR/3 sparse-hair NEAR/3 syndrom*) OR (Taurodontism) OR (Taurodontism NEAR/3 microdontia NEAR/3 -dens-invaginatus) OR (Teebi-Shaltout NEAR/3 syndrom*) OR (Tel-Hashomer-camptodactyly NEAR/3 syndrom*) OR (Telfer-Sugar-Jaeger NEAR/3 syndrom*) OR (Temple NEAR/3 syndrom*) OR (Temple-Baraitser NEAR/3 syndrom*) OR (Temporal-epilepsy NEAR/3 familial) OR (Temtamy-preaxial-brachydactyly NEAR/3 syndrom*) OR (Temtamy NEAR/3 syndrom*) OR (Tendons NEAR/3 extensor NEAR/3 fingers NEAR/3 anomalous-insertion-of) OR (Testotoxicosis) OR (Tethered-cord NEAR/3 syndrom*) OR (Tetra-amelia NEAR/3 syndrom*) OR (Tetraamelia NEAR/3 pulmonary-hypoplasia) OR (Tetraamelia-multiple-malformations NEAR/3 syndrom*) OR (Tetralogy NEAR/3 Fallot) OR (Tetralogy NEAR/3 fallot NEAR/3 glaucoma) OR (Tetramelic-monodactyly) OR (Tetraploidy) OR (Tetrasomy-21) OR (Thai-symphalangism NEAR/3 syndrom*) OR (Thakker-Donnai NEAR/3 syndrom*) OR (Thanatophoric-dysplasia-type-1) OR (Thanatophoric-dysplasia-type-2) OR (Thiopurine-S-methyltranferase NEAR/3 deficien*) OR (Thomas NEAR/3 syndrom*) OR (Thoracic-dysplasia-hydrocephalus NEAR/3 syndrom*) OR (Thoraco-abdominal-enteric NEAR/3 duplicat*) OR (Thumb-deformity) OR (Thumb-deformity NEAR/3 alopecia NEAR/3 pigmentation-anomaly) OR (Thumb-stiffness-brachydactyly-intellectual-disability NEAR/3 syndrom*) OR (Thymic-Renal-Anal-Lung-dysplasia) OR (Thyroid-dysgenesis) OR (Tibia-absent-polydactyly-arachnoid-cyst) OR (Tietz NEAR/3 syndrom*) OR (Tight-skin-contracture NEAR/3 syndrom* NEAR/3 lethal) OR (Timothy NEAR/3 syndrom*) OR (TMEM165-CDG-) OR (CDG-IIk) OR (Tollner-Horst-Manzke NEAR/3 syndrom*) OR (Tolosa-Hunt NEAR/3 syndrom*) OR (Tonoki NEAR/3 syndrom*) OR (Toriello-Carey NEAR/3 syndrom*) OR (Torticollis-keloids-cryptorchidism-renal-dysplasia) OR (Total-Hypotrichosis NEAR/3 Mari) OR (Townes-Brocks NEAR/3 syndrom*) OR (Tracheal-agenesis) OR (Tranebjaerg-Svejgaard NEAR/3 syndrom*) OR (Transaldolase NEAR/3 deficien*) OR (Transient-bullous-dermolysis NEAR/3 newborn) OR (Transient-infantile-liver-failure) OR (Transient-neonatal-diabetes-mellitus) OR (Treacher-Collins NEAR/3 syndrom*) OR (Treacher-Collins NEAR/3 syndrom*-3) OR (Tricho-dento-osseous NEAR/3 syndrom*) OR (Trichodental NEAR/3 syndrom*) OR (Trichohepatoenteric NEAR/3 syndrom*) OR (Trichomegaly NEAR/3 intellectual-disability NEAR/3 dwarfism NEAR/3 pigmentary-degeneration NEAR/3 retina) OR (Trichorhinophalangeal NEAR/3 syndrom*-type-1) OR (Trichorhinophalangeal NEAR/3 syndrom*-type-2) OR (Trichorhinophalangeal NEAR/3 syndrom*-type-3) OR (Trichothiodystroph*) OR (Tricuspid-atresia) OR (Trigonobrachycephaly NEAR/3 bulbous-bifid-nose NEAR/3 micrognathia NEAR/3 -abnormalities NEAR/3 hands NEAR/3 feet) OR (Trigonocephaly-bifid-nose-acral-anomalies) OR (Triphalangeal-thumbs-brachyectrodactyly) OR (Triple-A NEAR/3 syndrom*) OR (Trismus-pseudocamptodactyly NEAR/3 syndrom*) OR (Trisomy-18) OR (Trisomy-3-mosaicism) OR (Troyer NEAR/3 syndrom*) OR (Tryptophanuria NEAR/3 dwarfism) OR (Tubular-aggregate-myopathy) OR (Tucker NEAR/3 syndrom*) OR (Tufting-enteropathy) OR (Tukel NEAR/3 syndrom*) OR (Tumor-necrosis-factor-receptor-associated-periodic NEAR/3 syndrom*) OR (Twenty-nail-dystroph*) OR (Tylosis NEAR/3 esophageal-cancer) OR (Typical-congenital-nemaline-myopathy) OR (Tyrosine-hydroxylase NEAR/3 deficien*) OR (Tyrosine-oxidase-temporary NEAR/3 deficien*) OR (Tyrosinemia-type-1) OR (Tyrosinemia-type-2) OR (Tyrosinemia-type-3) OR (Ulerythema-ophryogenesis) OR (Ullrich-congenital-muscular-dystroph*) OR (Ulna NEAR/3 fibula NEAR/3 hypoplasia-of) OR (Ulna-hypoplasia-intellectual-disability NEAR/3 syndrom*) OR (Ulna-metaphyseal-dysplasia NEAR/3 syndrom*) OR (Ulnar-hypoplasia-lobster-claw-deformity NEAR/3 feet) OR (Ulnar-mammary NEAR/3 syndrom*) OR (Uncombable-hair NEAR/3 syndrom*) OR (Unverricht-Lundborg NEAR/3 diseas*) OR (Upington NEAR/3 diseas*) OR (Urachal-cyst) OR (Urogenital-adysplasia) OR (Usher NEAR/3 syndrom*-type-2A) OR (Usher NEAR/3 syndrom* NEAR/3 type-1) OR (UV-sensitive NEAR/3 syndrom*) OR (VACTERL-association) OR (VACTERL-association NEAR/3 hydrocephaly NEAR/3 X-linked) OR (VACTERL-hydrocephaly) OR (Vagina NEAR/3 absence-of) OR (Vagneur-Triolle-Ripert NEAR/3 syndrom*) OR (Van-Benthem-Driessen-Hanveld NEAR/3 syndrom*) OR (Van-Buchem NEAR/3 diseas*-type-2) OR (Van-Den-Bosch NEAR/3 syndrom*) OR (Van-den-Ende-Gupta NEAR/3 syndrom*) OR (Van-der-Woude NEAR/3 syndrom*) OR (Van-der-Woude NEAR/3 syndrom*-2) OR (Variegate-porphyria) OR (Vascular-hyalinosis) OR (Ventricular-extrasystoles NEAR/3 syncopal-episodes---perodactyly---Robin-sequence) OR (Verloes-Bourguignon NEAR/3 syndrom*) OR (Verloes-Van-Maldergem-Marneffe NEAR/3 syndrom*) OR (Verloove-Vanhorick-Brubakk NEAR/3 syndrom*) OR (Vibratory-urticaria) OR (Vici NEAR/3 syndrom*) OR (Viljoen-Kallis-Voges NEAR/3 syndrom*) OR (VLCAD NEAR/3 deficien*) OR (Vocal-cord-dysfunction-familial) OR (Vohwinkel NEAR/3 syndrom*) OR (Waardenburg NEAR/3 syndrom*-type-1) OR (Waardenburg NEAR/3 syndrom*-type-2) OR (Waardenburg NEAR/3 syndrom*-type-3) OR (Waardenburg NEAR/3 syndrom*-type-4) OR (Wagner NEAR/3 syndrom*) OR (WAGR NEAR/3 syndrom*) OR (Walker-Warburg NEAR/3 syndrom*) OR (Warfarin NEAR/3 syndrom*) OR (Warman-Mulliken-Hayward NEAR/3 syndrom*) OR (Weaver NEAR/3 syndrom*) OR (Weill-Marchesani NEAR/3 syndrom*) OR (Weissenbacher-Zweymuller NEAR/3 syndrom*) OR (Welander-distal-myopathy NEAR/3 Swedish) OR (Wells-Jankovic NEAR/3 syndrom*) OR (Werner NEAR/3 syndrom*) OR (West NEAR/3 syndrom*) OR (Weyers-acrofacial-dysostosis) OR (Weyers-ulnar-ray) OR (oligodactyly NEAR/3 syndrom*) OR (WHIM NEAR/3 syndrom*) OR (Whistling-face NEAR/3 syndrom* NEAR/3 recessive-form) OR (White-forelock NEAR/3 malformations) OR (White-matter-hypoplasia-corpus-callosum-agenesis-intellectual-disability NEAR/3 syndrom*) OR (White-sponge-nevus NEAR/3 cannon) OR (Wiedemann-Oldigs-Oppermann NEAR/3 syndrom*) OR (Wildervanck NEAR/3 syndrom*) OR (Wilms NEAR/3 tumor) OR (Wilson NEAR/3 diseas*) OR (Wilson-Turner NEAR/3 syndrom*) OR (Winchester NEAR/3 syndrom*) OR (Witkop NEAR/3 syndrom*) OR (Wolfram NEAR/3 syndrom*) OR (Wolman NEAR/3 diseas*) OR (Woodhouse-Sakati NEAR/3 syndrom*) OR (Woods-Black-Norbury NEAR/3 syndrom*) OR (Woolly-hair-hypotrichosis-everted-lower-lip NEAR/3 outstanding-ears) OR (Woolly-hair NEAR/3 syndrom*) OR (Worth-type-autosomal-dominant-osteosclerosis) OR (Wrinkly-skin NEAR/3 syndrom*) OR (X-linked-adrenal-hypoplasia-congenita) OR (X-linked-agammaglobulinemia) OR (X-linked-cardiac-valvular-dysplasia) OR (X-linked-cerebral-adrenoleukodystroph*) OR (X-linked-Charcot-Marie-Tooth NEAR/3 diseas*-type-1) OR (X-linked-Charcot-Marie-Tooth NEAR/3 diseas*-type-2) OR (X-linked-Charcot-Marie-Tooth NEAR/3 diseas*-type-3) OR (X-linked-Charcot-Marie-Tooth NEAR/3 diseas*-type-4) OR (X-linked-Charcot-Marie-Tooth NEAR/3 diseas*-type-5) OR (X-linked-Charcot-Marie-Tooth NEAR/3 diseas*-type-6) OR (X-linked-complicated-corpus-callosum-agenesis) OR (X-linked-complicated-spastic-paraplegia-type-1) OR (X-linked-congenital-generalized-hypertrichosis) OR (X-linked-congenital-stationary-night-blindness) OR (X-linked-creatine NEAR/3 deficien*) OR (X-linked-dominant-chondrodysplasia-punctata-2) OR (X-linked-dystonia-parkinsonism) OR (X-linked-hereditary-sensory NEAR/3 autonomic-neuropathy NEAR/3 deafness) OR (X-linked-hypohidrotic-ectodermal-dysplasia) OR (X-linked-hypophosphatemia) OR (X-linked-ichthyosis) OR (X-linked-intellectual-disability---corpus-callosum-agenesis---spastic-quadriparesis) OR (X-linked-intellectual-disability---short-stature---obesity) OR (X-linked-intellectual-disability NEAR/3 Abidi) OR (X-linked-intellectual-disability NEAR/3 Najm) OR (X-linked-intellectual-disability NEAR/3 Schimke) OR (X-linked-intellectual-disability NEAR/3 Siderius) OR (X-linked-intellectual-disability NEAR/3 Turner) OR (X-linked-intellectual-disability-dysmorphism-cerebral-atrophy NEAR/3 syndrom*) OR (X-linked-intellectual-disability-plagiocephaly NEAR/3 syndrom*) OR (X-linked-lissencephaly NEAR/3 abnormal-genitalia) OR (X-linked-lymphoproliferative NEAR/3 diseas*-due-to-SH2D1A NEAR/3 deficien*) OR (X-linked-lymphoproliferative NEAR/3 syndrom*) OR (X-linked-myopathy NEAR/3 excessive-autophagy) OR (X-linked-myotubular-myopathy) OR (X-linked-non-specific-intellectual-disability) OR (X-linked-periventricular-heterotopia) OR (X-linked-severe-combined-immunodeficiency) OR (X-linked-skeletal-dysplasia-intellectual-disability NEAR/3 syndrom*) OR (Xanthinuria-type-1) OR (Xanthinuria-type-2) OR (Xeroderma-pigmentosum) OR (XFE-progeroid NEAR/3 syndrom*) OR (Xia-Gibbs NEAR/3 syndrom*) OR (XK-aprosencephaly) OR (Xp22.3 NEAR/3 microdeletion* NEAR/3 syndrom*) OR (Yemenite-deaf-blind-hypopigmentation NEAR/3 syndrom*) OR (Yorifuji-Okuno NEAR/3 syndrom*) OR (Young NEAR/3 syndrom*) OR (Yunis-Varon NEAR/3 syndrom*) OR (Zadik-Barak-Levin NEAR/3 syndrom*) OR (ZAP-70 NEAR/3 deficien*) OR (Zazam-Sheriff-Phillips NEAR/3 syndrom*) OR (Zechi-Ceide NEAR/3 syndrom*) OR (Zellweger NEAR/3 syndrom*) OR (Zlotogora NEAR/3 syndrom*) OR (Zori-Stalker-Williams NEAR/3 syndrom*) OR (ZTTK NEAR/3 syndrom*) OR (Zunich-neuroectodermal NEAR/3 syndrom*) OR (Amyopathic-dermatomyositis) OR (Bizarre-parosteal-osteochondromatous-proliferation) OR (Classical-Ehlers-Danlos-syndrom*) OR (Ehlers-Danlos-syndrom*) OR (Eosinophilic-fasciitis) OR (Mixed-connective-tissue-diseas*) OR (Osteosclerosis NEAR/3 ichthyosis NEAR/3 premature-ovarian-failure) OR (Paget-disease NEAR/3 bone NEAR/3 familial) OR (Polymyositis) OR (Rheumatoid-factor-negative-juvenile-idiopathic-arthritis) OR (Ribbing-diseas*) OR (Temporomandibular-ankylosis) OR (Benign-recurrent-intrahepatic-cholestasis-1) OR (Benign-recurrent-intrahepatic-cholestasis-2) OR (Boerhaave-syndrom*) OR (Budd-Chiari-syndrom*) OR (Childhood-hepatocellular-carcinoma) OR (Chronic-hiccups) OR (Collagenous-gastritis) OR (Congenital NEAR/3 disorder* NEAR/3 glycosylation) OR (Cutaneous-photosensitivity NEAR/3 colitis NEAR/3 lethal) OR (Desmoplastic-small-round-cell-tumor) OR (Disseminated-peritoneal-leiomyomatosis) OR (Duodenal-ulcer-due-to-antral-G-cell-hyperfunction) OR (Eosinophilic-gastroenteritis) OR (Galactose-epimerase-deficiency) OR (Goblet-cell-carcinoid) OR (Hepatic-encephalopathy) OR (Hepatic-veno-occlusive-diseas*) OR (Hepatoblastoma) OR (Idiopathic-achalasia) OR (Intrahepatic-cholestasis NEAR/3 pregnancy) OR (Klatskin-tumor) OR (Malakoplakia) OR (Mallory-Weiss-syndrom*) OR (Menetrier-diseas*) OR (Necrotizing-enterocolitis) OR (Nodular-regenerative-hyperplasia) OR (Pancreatic-adenoma) OR (Pediatric-Crohn NEAR/3 diseas*) OR (Pediatric-ulcerative-colitis) OR (Primary-biliary-cholangitis) OR (Primary-sclerosing-cholangitis) OR (Progressive-familial-intrahepatic-cholestasis-4) OR (Pseudomyxoma-peritonei) OR (Renal-nutcracker-syndrom*) OR (Retroperitoneal-fibrosis) OR (Sandifer-syndrom*) OR (Sclerosing-mesenteritis) OR (Small-Intestinal-Adenocarcinoma) OR (Superior-mesenteric-artery-syndrom*) OR (VIPoma) OR (Watermelon-stomach) OR (Whipple-diseas*) OR (Zollinger-Ellison-syndrom*) OR (Auditory-neuropathy-spectrum-disorder*) OR (Cholesteatoma) OR (Fetal-indomethacin-syndrom*) OR (Fetal-minoxidil-syndrom*) OR (IgG4-related-dacryoadenitis NEAR/3 sialadenitis) OR (Mal-de-debarquement-syndrom*) OR (Recurrent-respiratory-papillomatosis) OR (Vestibulocochlear-dysfunction NEAR/3 progressive) OR (Acquired-generalized-lipodystrophy) OR (Acromegaly) OR (ACTH-secreting-pituitary-adenoma) OR (Ahumada-Del-Castillo-syndrom*) OR (Autoimmune-polyglandular-syndrome-type-3) OR (Central-nervous-system-germinoma) OR (Cushing NEAR/3 syndrom*) OR (Diencephalic-syndrom*) OR (Familial-chylomicronemia-syndrom*) OR (Gigantism) OR (Granulomatous-hypophysitis) OR (Growth-hormone-deficiency) OR (Holoprosencephaly) OR (Hyperadrenalism) OR (Hypoparathyroidism) OR (Hypopituitarism) OR (Iodine-antenatal-exposure) OR (Langerhans-cell-histiocytosis) OR (Meningioma) OR (Merkel-cell-carcinoma) OR (Optic-pathway-glioma) OR (Papillary-thyroid-carcinoma) OR (Parathyroid-carcinoma) OR (Primary-hyperparathyroidism) OR (Progressive-encephalomyelitis NEAR/3 rigidity NEAR/3 myoclonus) OR (Prolactinoma) OR (Pseudohypoparathyroidism) OR (Sheehan-syndrom*) OR (Small-cell-carcinoma NEAR/3 bladder) OR (Thyrotropin-deficiency NEAR/3 isolated) OR (Waterhouse-Friderichsen-syndrom*) OR (Achromatopsia-2) OR (Achromatopsia-3) OR (Aland-island-eye-diseas*) OR (Aniridia) OR (Anterior-ischemic-optic-neuropathy) OR (Anterior-uveitis) OR (Autosomal-recessive-bestrophinopathy) OR (Birdshot-chorioretinopathy) OR (Brown-syndrom*) OR (Cataract-Hutterite-type) OR (Cataract NEAR/3 posterior-polar NEAR/3 1) OR (Cataract NEAR/3 posterior-polar NEAR/3 3) OR (Cataract NEAR/3 posterior-polar NEAR/3 4) OR (Cataract NEAR/3 posterior-polar NEAR/3 5) OR (Centronuclear-myopathy) OR (Chandler NEAR/3 syndrom*) OR (Charles-Bonnet-syndrom*) OR (Chorioretinitis) OR (Cone-dystrophy-X-linked NEAR/3 tapetal-like-sheen) OR (Cone-rod-dystrophy-1) OR (Cone-rod-dystrophy-2) OR (Congenital-cystic-eye) OR (Corneal-dystrophy NEAR/3 Bowman-layer-type-1) OR (Dermoids NEAR/3 cornea) OR (Developmental-prosopagnosia) OR (Doyne-honeycomb-retinal-dystrophy) OR (Eales-diseas*) OR (Ectopia-lentis NEAR/3 isolated-autosomal-recessive) OR (Enthesitis-related-juvenile-idiopathic-arthritis) OR (Epithelial-basement-membrane-corneal-dystrophy) OR (Familial-isolated-hypoparathyroidism) OR (Fuchs-heterochromic-iridocyclitis) OR (Fundus-dystrophy NEAR/3 pseudoinflammatory NEAR/3 -Sorsby) OR (Groenouw-type-I-corneal-dystrophy) OR (Intraocular-melanoma) OR (Keratoconus) OR (Krabbe-diseas*) OR (Lattice-corneal-dystrophy-type-3A) OR (Leber-congenital-amaurosis-1) OR (Leber-congenital-amaurosis-10) OR (Leber-congenital-amaurosis-11) OR (Leber-congenital-amaurosis-12) OR (Leber-congenital-amaurosis-13) OR (Leber-congenital-amaurosis-14) OR (Leber-congenital-amaurosis-15) OR (Leber-congenital-amaurosis-16) OR (Leber-congenital-amaurosis-2) OR (Leber-congenital-amaurosis-3) OR (Leber-congenital-amaurosis-4) OR (Leber-congenital-amaurosis-6) OR (Leber-congenital-amaurosis-9) OR (Microcornea-posterior-megalolenticonus-persistent-fetal-vasculature-coloboma) OR (Microcornea-corectopia-macular-hypoplasia) OR (Microspherophakia NEAR/3 hernia) OR (Neuromyelitis-optica-spectrum-disorder*) OR (Neuronal-ceroid-lipofuscinosis) OR (Nystagmus-1 NEAR/3 congenital NEAR/3 X--linked) OR (Nystagmus-2 NEAR/3 congenital NEAR/3 autosomal-dominant) OR (O-Donnell-Pappas-syndrom*) OR (Ocular-neuromyotonia) OR (Opsoclonus-myoclonus-syndrom*) OR (Optic-atrophy-5) OR (Optic-atrophy-6) OR (Optic-neuritis) OR (Orbital-varix) OR (Panuveitis) OR (Pattern-dystrophy) OR (Pelizaeus-Merzbacher-diseas*) OR (Peters-anomaly) OR (Posterior-uveitis) OR (Pterygium NEAR/3 conjunctiva NEAR/3 cornea) OR (Punctate-inner-choroidopathy) OR (Reese-retinal-dysplasia) OR (Retinal-cone-dystrophy-2) OR (Retinal-cone-dystrophy-3A) OR (Retinal-cone-dystrophy-3B) OR (Retinal-cone-dystrophy-4) OR (Retinitis-pigmentosa-1) OR (Retinitis-Pigmentosa-11) OR (Retinitis-pigmentosa-12) OR (Retinitis-Pigmentosa-13) OR (Retinitis-Pigmentosa-14) OR (Retinitis-Pigmentosa-15) OR (Retinitis-Pigmentosa-17) OR (Retinitis-Pigmentosa-18) OR (Retinitis-Pigmentosa-19) OR (Retinitis-Pigmentosa-20) OR (Retinitis-Pigmentosa-22) OR (Retinitis-Pigmentosa-23) OR (Retinitis-Pigmentosa-24) OR (Retinitis-Pigmentosa-25) OR (Retinitis-Pigmentosa-26) OR (Retinitis-Pigmentosa-28) OR (Retinitis-pigmentosa-29) OR (Retinitis-pigmentosa-3) OR (Retinitis-Pigmentosa-30) OR (Retinitis-Pigmentosa-31) OR (Retinitis-Pigmentosa-32) OR (Retinitis-Pigmentosa-33) OR (Retinitis-Pigmentosa-34) OR (Retinitis-Pigmentosa-35) OR (Retinitis-Pigmentosa-36) OR (Retinitis-Pigmentosa-4) OR (Retinitis-Pigmentosa-41) OR (Retinitis-Pigmentosa-6) OR (Retinitis-Pigmentosa-7) OR (Retinitis-Pigmentosa-9) OR (Retinopathy NEAR/3 prematurity) OR (Ring-dermoid NEAR/3 cornea) OR (Sandhoff-diseas*) OR (Serpiginous-choroiditis) OR (Spondyloepiphyseal-dysplasia) OR (Superior-limbic-keratoconjunctivitis) OR (Tay-Sachs-diseas*) OR (Trachoma) OR (Tubulointerstitial-nephritis NEAR/3 uveitis) OR (Usher-syndrom*) OR (Usher-syndrome-type-3A) OR (Usher-syndrome NEAR/3 type-1B) OR (Usher-syndrome NEAR/3 type-1C) OR (Usher-syndrome NEAR/3 type-1D) OR (Usher-syndrome NEAR/3 type-1E) OR (Usher-syndrome NEAR/3 type-1F) OR (Usher-syndrome NEAR/3 type-2B) OR (Usher-syndrome NEAR/3 type-2C) OR (Uveal-diseases) OR (Vernal-keratoconjunctivitis) OR (Vogt-Koyanagi-Harada-diseas*) OR (Achard-Thiers-syndrom*) OR (Asherman NEAR/3 syndrom*) OR (Benign-mesonephroma) OR (Diabetic-mastopathy) OR (Diethylstilbestrol-syndrom*) OR (Extramammary-Paget-diseas*) OR (Fowler NEAR/3 syndrom*) OR (Granulomatous-lobular-mastitis) OR (HELLP-syndrom*) OR (Hydatidiform-mole) OR (Metaplastic-carcinoma NEAR/3 breast) OR (Ovarian-carcinosarcoma) OR (Paget-disease NEAR/3 breast) OR (Peripartum-cardiomyopathy) OR (Pruritic-urticarial-papules-plaques NEAR/3 pregnancy) OR (Uterine-Carcinosarcoma) OR (Aberrant-subclavian-artery) OR (Arrhythmogenic-right-ventricular-cardiomyopathy) OR (Baroreflex-failure) OR (Bidirectional-tachycardia) OR (Broken-heart-syndrom*) OR (Brugada-syndrome-3) OR (Brugada-syndrome-4) OR (Cardiac-hydatid-cysts NEAR/3 intracavitary-expansion) OR (Cardiac-rupture) OR (Chaotic-atrial-tachycardia) OR (Diffuse-cutaneous-systemic-sclerosis) OR (Familial-hypertrophic-cardiomyopathy) OR (Fibrocartilaginous-embolism) OR (Gaucher-diseas*) OR (Glycogen-storage-disease-type-2) OR (Intracranial-arteriovenous-malformation) OR (Kallikrein-hypertension) OR (Limited-cutaneous-systemic-sclerosis) OR (Limited-systemic-sclerosis) OR (Lymphocytic-vasculitis) OR (Neonatal-stroke) OR (Patent-ductus-arteriosus) OR (Patent-ductus-venosus) OR (Pulmonary-arterial-hypertension) OR (Pulmonary-valve-stenosis) OR (Pulmonic-stenosis) OR (Renoprival-hypertension) OR (Sudden-Arrhythmia-Death-syndrom*) OR (Duodenal-carcinoid-syndrom*) OR (Endolymphatic-sac-tumor) OR (Familial-adenomatous-polyposis) OR (Familial-isolated-pituitary-adenoma) OR (Hirschsprung-disease-ganglioneuroblastoma) OR (MYH-associated-polyposis) OR (Pheochromocytoma-islet-cell-tumor-syndrom*) OR (Premature-aging-Okamoto-type) OR (Stewart-Treves-syndrom*) OR (Adult-onset-immunodeficiency NEAR/3 anti-interferon-gamma-autoantibodies) OR (Allergic-bronchopulmonary-aspergillosis) OR (Amyloidosis-AA) OR (Amyloidosis-familial-visceral) OR (Autosomal-recessive-hyper-IgE-syndrom*) OR (Bronchiolitis-obliterans) OR (Chronic-graft-versus-host-diseas*) OR (Complement-component-8-deficiency-type-1) OR (Complement-component-8-deficiency-type-2) OR (Cryoglobulinemic-vasculitis) OR (Felty NEAR/3 syndrom*) OR (Hashimoto-encephalopathy) OR (Hyper-IgE-syndrom*) OR (Immune-dysfunction NEAR/3 T-cell-inactivation-due-to-calcium-entry-defect-1) OR (Immune-dysfunction NEAR/3 T-cell-inactivation-due-to-calcium-entry-defect-2) OR (Immunodeficiency NEAR/3 thymoma) OR (Immunodeficiency NEAR/3 anhidrotic-ectodermal-dysplasia) OR (Immunoglobulin-A-deficiency-2) OR (Lymphocytic-hypophysitis) OR (Melkersson-Rosenthal-syndrom*) OR (MHC-class-1-deficiency) OR (Multifocal-fibrosclerosis) OR (Neonatal-systemic-lupus-erythematosus) OR (Palindromic-rheumatism) OR (Pediatric-multiple-sclerosis) OR (Relapsing-polychondritis) OR (Schnitzler-syndrom*) OR (Severe-combined-immunodeficiency NEAR/3 sensitivity-to-ionizing-radiation) OR (Severe-combined-immunodeficiency NEAR/3 atypical) OR (Simple-cryoglobulinemia) OR (Stevens-Johnson-syndrom*) OR (toxic-epidermal-necrolysis) OR (X-linked-lymphoproliferative-syndrome-2) OR (Adult-onset-Still NEAR/3 diseas*) OR (Autosomal-dominant-tubulointerstitial-kidney-diseas*) OR (Autosomal-dominant-tubulointerstitial-kidney-disease-due-to-MUC1-mutations) OR (BK-virus-nephropathy) OR (Collecting-duct-carcinoma) OR (Cystinuria) OR (Dermatomyositis) OR (Dihydroxyadeninuria) OR (Fibrillary-glomerulonephritis) OR (Glomerulonephritis) OR (IgA-nephropathy) OR (Immunotactoid-glomerulopathy) OR (Infundibulopelvic-dysgenesis) OR (Juvenile-dermatomyositis) OR (Juvenile-polymyositis) OR (Lupus-nephritis) OR (Membranous-nephropathy) OR (Methylmalonic-acidemia) OR (Minimal-change-diseas*) OR (Nephrocalcinosis) OR (Nephronophthisis) OR (Polyomavirus-allograft-nephropathy) OR (Postorgasmic-illness-syndrom*) OR (Renal-medullary-carcinoma) OR (Renal-tubular-dysgenesis) OR (Testicular-seminoma) OR (Asbestosis) OR (Autoimmune-pulmonary-alveolar-proteinosis) OR (Beryllium-diseas*) OR (Bronchiolitis-obliterans-organizing-pneumonia) OR (Catamenial-pneumothorax) OR (Children NEAR/3 interstitial-lung-diseas*) OR (Chronic-thromboembolic-pulmonary-hypertension) OR (Coal-worker NEAR/3 pneumoconiosis) OR (Costocoracoid-ligament-congenitally-short) OR (Cryptogenic-organizing-pneumonia) OR (Diffuse-idiopathic-pulmonary-neuroendocrine-cell-hyperplasia) OR (Diffuse-panbronchiolitis) OR (Fibrosing-mediastinitis) OR (Idiopathic-pulmonary-fibrosis) OR (Idiopathic-pulmonary-hemosiderosis) OR (Kaolin-pneumoconiosis) OR (Meconium-aspiration-syndrom*) OR (Nocardiosis) OR (Pleuroparenchymal-fibroelastosis) OR (Psoriatic-juvenile-idiopathic-arthritis) OR (Pulmonary-sequestration) OR (Respiratory-distress-syndrome NEAR/3 infant) OR (Silicosis) OR (Systemic-onset-juvenile-idiopathic-arthritis) OR (Tracheobronchomalacia) OR (Tracheobronchopathia-osteoplastica) OR (Androgen-insensitivity-syndrom*) OR (Androgen-insensitivity-syndrome NEAR/3 mild) OR (Hansen NEAR/3 diseas*) OR (Prostatic-malacoplakia-associated NEAR/3 prostatic-abscess) OR (Sertoli-cell-only-syndrom*) OR (2-Hydroxyglutaric-aciduria) OR (Abdominal-obesity-metabolic-syndrom*) OR (Acetyl-CoA-acetyltransferase-2-deficiency) OR (Acetyl-carnitine-deficiency) OR (Apparent-mineralocorticoid-excess) OR (Bartter-syndrom*) OR (Bartter-syndrome-antenatal-type-1) OR (Bartter-syndrome-antenatal-type-2) OR (Central-diabetes-insipidus) OR (Chondrocalcinosis-1) OR (Chondrocalcinosis-due-to-apatite-crystal-deposition) OR (Citrulline-transport-defect) OR (Congenital-disorder NEAR/3 glycosylation-type-I) OR (Congenital-disorder NEAR/3 glycosylation-type-IIX) OR (CoQ-responsive-OXPHOS-deficiency) OR (Cytochrome-c-oxidase-deficiency) OR (Dipsogenic-diabetes-insipidus) OR (Fatal-infantile-encephalomyopathy) OR (Gestational-diabetes-insipidus) OR (Glutathione-synthetase-deficiency) OR (Glycogen-storage-disease-8) OR (Glycoproteinosis) OR (Hereditary-amyloidosis) OR (Homocysteinemia) OR (Hyperglycerolemia) OR (Hypolipoproteinemia) OR (Infantile-free-sialic-acid-storage-diseas*) OR (Krabbe-disease-atypical-due-to-Saposin-A-deficiency) OR (Lactate-dehydrogenase-A-deficiency) OR (Lactate-dehydrogenase-deficiency) OR (Leucine-sensitive-hypoglycemia NEAR/3 infancy) OR (Lipase-deficiency-combined) OR (Metachromatic-leukodystrophy) OR (Metachromatic-leukodystrophy-due-to-saposin-B-deficiency) OR (Morquio-syndrome-B) OR (Mucopolysaccharidosis-type-II) OR (Mucopolysaccharidosis-type-VI) OR (N-acetyltransferase-deficiency) OR (N-acetyl-alpha-D-galactosaminidase-deficiency-type-III) OR (Phosphoribosylpyrophosphate-synthetase-superactivity) OR (Primary-hyperoxaluria-type-3) OR (Pyruvate-carboxylase-deficiency) OR (Pyruvate-dehydrogenase-complex-deficiency) OR (Refsum-disease NEAR/3 increased-pipecolic-acidemia) OR (Rhizomelic-chondrodysplasia-punctata-type-3) OR (Tiglic-acidemia) OR (Transcobalamin-1-deficiency) OR (Trehalase-deficiency) OR (Trimethylaminuria) OR (Urea-cycle NEAR/3 disorder*) OR (Valinemia) OR (X-linked-adrenoleukodystrophy) OR (Ameloblastic-carcinoma) OR (Florid-cemento-osseous-dysplasia) OR (Gingival-fibromatosis NEAR/3 1) OR (Gingival-fibromatosis NEAR/3 2) OR (Gingival-fibromatosis NEAR/3 3) OR (Gingival-fibromatosis NEAR/3 4) OR (Hemifacial-myohyperplasia) OR (Oral-submucous-fibrosis) OR (Xanthogranulomatous-sialadenitis) OR (Acromesomelic-dysplasia) OR (Amyotrophy NEAR/3 neurogenic-scapuloperoneal NEAR/3 New-England-type) OR (Arthrogryposis-multiplex-congenita) OR (Axial-osteomalacia) OR (Baby-rattle-pelvic-dysplasia) OR (Cervical-dystonia) OR (Chondrosarcoma) OR (Chronic-recurrent-multifocal-osteomyelitis) OR (Coccygodynia) OR (Collagenopathy-type-2-alpha-1) OR (Congenital-radioulnar-synostosis) OR (Dysferlinopathy) OR (DYT-GNAL) OR (Erdheim-Chester-diseas*) OR (Ewing-sarcoma) OR (Familial-tumoral-calcinosis) OR (Freiberg NEAR/3 diseas*) OR (Giant-cell-tumor NEAR/3 bone) OR (Hyperphosphatemic-familial-tumoral-calcinosis) OR (Inclusion-body-myositis) OR (Iridogoniodysgenesis-type-1) OR (Kienbock NEAR/3 diseas*) OR (Kohler-diseas*) OR (Lambert-Eaton-myasthenic-syndrom*) OR (Levator-syndrom*) OR (Macrophagic-myofasciitis) OR (MAGIC-syndrom*) OR (Monomelic-amyotrophy) OR (Multiple-epiphyseal-dysplasia) OR (Muscular-dystrophy) OR (Myostatin-related-muscle-hypertrophy) OR (Myotonic-dystrophy) OR (Neurofibromatosis-type-1) OR (Normophosphatemic-familial-tumoral-calcinosis) OR (Osteochondritis-dissecans) OR (Osteodysplasty-precocious NEAR/3 Danks-Mayne NEAR/3 Kozlowski) OR (Osteosarcoma) OR (Pigmented-villonodular-synovitis) OR (Piriformis-syndrom*) OR (Pleoconial-myopathy NEAR/3 salt-craving) OR (Polycystic-bone-diseas*) OR (Pyoderma-gangrenosum) OR (Radio-ulnar-synostosis-type-1) OR (Radio-ulnar-synostosis-type-2) OR (Reactive-arthritis) OR (SAPHO-syndrom*) OR (Spheroid-body-myopathy) OR (Spinal-muscular-atrophy-Ryukyuan-type) OR (Spinal-muscular-atrophy-type-1 NEAR/3 congenital-bone-fractures) OR (Spinal-muscular-atrophy-type-4) OR (Spinal-muscular-atrophy NEAR/3 respiratory-distress-1) OR (Synovial-Chondromatosis) OR (Tarsal-tunnel-syndrom*) OR (Tietze-syndrom*) OR (Trochlea NEAR/3 humerus-aplasia-of) OR (Trochlear-dysplasia) OR (Undifferentiated-pleomorphic-sarcoma) OR (X-linked-dominant-scapuloperoneal-myopathy) OR (Absence NEAR/3 septum-pellucidum) OR (Adie-syndrom*) OR (Agnosia) OR (AIDS-Dementia-Complex) OR (Alzheimer-disease-type-4) OR (Alzheimer NEAR/3 disease NEAR/3 neurofibrillary-tangles) OR (Amyloid-neuropathy) OR (Amyotrophic-lateral-sclerosis) OR (Amyotrophic-lateral-sclerosis-type-6) OR (Amyotrophic-lateral-sclerosis-parkinsonism) OR (dementia-complex-1) OR (Anaplastic-astrocytoma) OR (Anaplastic-ganglioglioma) OR (Anaplastic-oligodendroglioma) OR (Antisynthetase-syndrom*) OR (Apraxia) OR (Arachnoiditis) OR (Autosomal-recessive-juvenile-Parkinson-diseas*) OR (Behavioral-variant NEAR/3 frontotemporal-dementia) OR (Bell NEAR/3 palsy) OR (Benign-rolandic-epilepsy) OR (Binswanger NEAR/3 diseas*) OR (Bobble-head-doll-syndrom*) OR (Brown-Sequard-syndrom*) OR (Camptocormism) OR (CANOMAD-syndrom*) OR (Central-neurocytoma) OR (Central-pain-syndrom*) OR (Cerebellar-degeneration) OR (Cerebral-palsy-ataxic) OR (Cerebral-palsy-athetoid) OR (Cerebral-palsy-spastic-hemiplegic) OR (Cerebral-palsy-spastic-monoplegic) OR (Cerebral-palsy-spastic-quadriplegic) OR (Cerebral-sclerosis-similar-to-Pelizaeus-Merzbacher-diseas*) OR (Cerebrospinal-fluid-leak) OR (Charcot-Marie-Tooth-diseas*) OR (Chiari-malformation-type-4) OR (Choroid-plexus-carcinoma) OR (Choroid-plexus-papilloma) OR (Chronic-lymphocytic-inflammation NEAR/3 pontine-perivascular-enhancement-responsive-to-steroids) OR (Coenzyme-Q10-deficiency) OR (Complex-regional-pain-syndrom*) OR (Creutzfeldt-Jakob-diseas*) OR (Cytomegalic-inclusion-diseas*) OR (Developmental-dysphasia-familial) OR (Dysautonomia-like-disorder*) OR (Dyssynergia-cerebellaris-myoclonica) OR (Eastern-equine-encephalitis) OR (Empty-sella-syndrom*) OR (Encephalitis-lethargica) OR (Ependymoma) OR (Epilepsy-occipital-calcifications) OR (Epilepsy-progressive-myoclonic-type-3) OR (Episodic-ataxia) OR (Familial-transthyretin-amyloidosis) OR (Frontotemporal-dementia) OR (Gangliocytoma) OR (Gerstmann-syndrom*) OR (Gliomatosis-cerebri) OR (Guillain-Barre-syndrom*) OR (Hemangioblastoma) OR (Hemicrania-continua) OR (Hereditary-spastic-paraplegia) OR (Herpes-zoster-oticus) OR (HTLV-1-associated-myelopathy) OR (tropical-spastic-paraparesis) OR (Hypothalamic-hamartomas) OR (Idiopathic-intracranial-hypertension) OR (Idiopathic-spinal-cord-herniation) OR (Intraneural-perineurioma) OR (Isaacs-syndrom*) OR (Juvenile-Huntington-diseas*) OR (Klumpke-paralysis) OR (Kuru) OR (Kuzniecky-Andermann-syndrom*) OR (La-Crosse-encephalitis) OR (Leukodystrophy NEAR/3 dysmyelinating NEAR/3 -spastic-paraparesis NEAR/3 dystonia) OR (Lewis-Sumner-syndrom*) OR (Limbic-encephalitis NEAR/3 LGI1-antibodies) OR (Lissencephaly-1) OR (Lissencephaly-X-linked) OR (Localized-hypertrophic-neuropathy) OR (Locked-in-syndrom*) OR (Logopenic-progressive-aphasia) OR (Macrothrombocytopenia-progressive-deafness) OR (Marchiafava-Bignami-diseas*) OR (Meralgia-paresthetica) OR (Microcephaly) OR (Migraine NEAR/3 brainstem-aura) OR (Miller-Fisher-syndrom*) OR (Morvan NEAR/3 fibrillary-chorea) OR (Multifocal-motor-neuropathy) OR (Myasthenia-gravis) OR (Myelomeningocele) OR (Narcolepsy) OR (Necrotizing-autoimmune-myopathy) OR (Neonatal-meningitis) OR (Neuroblastoma) OR (Neurocutaneous-melanosis) OR (Neuroleptic-malignant-syndrom*) OR (New-onset-refractory-status-epilepticus) OR (Non-24-hour-sleep-wake-disorder*) OR (Nondystrophic-myotonia) OR (Olfactory-neuroblastoma) OR (Oligoastrocytoma) OR (Oligodendroglioma) OR (Olivopontocerebellar-atrophy) OR (Painful-orbital NEAR/3 systemic-neurofibromas-marfanoid-habitus-syndrom*) OR (Pantothenate-kinase-associated-neurodegeneration) OR (Paralysis-agitans NEAR/3 juvenile NEAR/3 -Hunt) OR (Parkinson-disease-type-3) OR (Paroxysmal-hemicrania) OR (Parsonage-Turner-syndrom*) OR (Periventricular-leukomalacia) OR (Photosensitive-epilepsy) OR (Plasmacytoma) OR (Pleomorphic-xanthoastrocytoma) OR (Poliomyelitis) OR (Polyarteritis-nodosa) OR (Pontocerebellar-hypoplasia) OR (Post-Polio-syndrom*) OR (Posterior-column-ataxia) OR (Primary-amebic-meningoencephalitis) OR (Primary-melanoma NEAR/3 central-nervous-system) OR (Primary-orthostatic-tremor) OR (Primary-progressive-aphasia) OR (Progressive-bulbar-palsy) OR (Progressive-hemifacial-atrophy) OR (Pudendal-Neuralgia) OR (Pure-autonomic-failure) OR (Radiation-induced-brachial-plexopathy) OR (Rasmussen-encephalitis) OR (Restless-legs-syndrome NEAR/3 susceptibility-to NEAR/3 1) OR (Restless-legs-syndrome NEAR/3 susceptibility-to NEAR/3 2) OR (Restless-legs-syndrome NEAR/3 susceptibility-to NEAR/3 3) OR (Restless-legs-syndrome NEAR/3 susceptibility-to NEAR/3 4) OR (Restless-legs-syndrome NEAR/3 susceptibility-to NEAR/3 5) OR (Restless-legs-syndrome NEAR/3 susceptibility-to NEAR/3 6) OR (Reversible-cerebral-vasoconstriction-syndrom*) OR (Rhabdoid-tumor) OR (Shapiro-syndrom*) OR (Spastic-diplegia-cerebral-palsy) OR (Spastic-diplegia-infantile-type) OR (Spastic-paraplegia-39) OR (Spinal-meningioma) OR (Spinal-shock) OR (Spinocerebellar-ataxia) OR (Spinocerebellar-ataxia-3) OR (Spinocerebellar-ataxia-30) OR (Spinocerebellar-ataxia-9) OR (Spinocerebellar-ataxia-autosomal-recessive-6) OR (Spinocerebellar-ataxia-X-linked-type-2) OR (Status-epilepticus) OR (Subependymal-giant-cell-astrocytoma) OR (Subependymoma) OR (Symmetrical-thalamic-calcifications) OR (Tarlov-cysts) OR (Thyrotoxic-periodic-paralysis) OR (Transverse-myelitis) OR (Trichinosis) OR (Trigeminal-neuralgia) OR (Tumefactive-multiple-sclerosis) OR (Variant-Creutzfeldt-Jakob-diseas*) OR (Visual-snow-syndrom*) OR (Wernicke-Korsakoff-syndrom*) OR (Worster-Drought-syndrom*) OR (2-4-Dienoyl-CoA-reductase-deficiency) OR (21-hydroxylase-deficiency) OR (Carnitine-palmitoyltransferase-2-deficiency) OR (Citrullinemia-type-I) OR (Congenital-human-immunodeficiency-virus) OR (Congenital-hypothyroidism) OR (Glucose-6-phosphate-dehydrogenase-deficiency) OR (Maple-syrup-urine-diseas*) OR (Medium-chain-3-ketoacyl-coa-thiolase-deficiency) OR (Methylmalonic-acidemia NEAR/3 homocystinuria) OR (Mucopolysaccharidosis-type-I) OR (Phenylketonuria) OR (Tetrahydrobiopterin-deficiency) OR (Keshan-diseas*) OR (Rickets) OR (Scurvy) OR (Cutaneous-sclerosis) OR (Focal-task-specific-dystonia) OR (Abdominal-chemodectomas NEAR/3 cutaneous-angiolipomas) OR (Acrodermatitis) OR (Actinic-lichen-planus) OR (Adiposis-dolorosa) OR (Ainhum) OR (Annular-atrophic-lichen-planus) OR (Annular-lichen-planus) OR (Atrophic-lichen-planus) OR (Atrophoderma NEAR/3 Pasini NEAR/3 Pierini) OR (Atrophoderma-vermiculata) OR (Basaran-Yilmaz-syndrom*) OR (Becker NEAR/3 nevus) OR (Benign-eccrine-spiradenoma) OR (Brunsting-Perry-syndrom*) OR (Cheilitis-glandularis) OR (Chromhidrosis) OR (Corticosteroid-sensitive-aseptic-abscesses) OR (Cutaneous-collagenous-vasculopathy) OR (Cutaneous-polyarteritis-nodosa) OR (Cutis-verticis-gyrata) OR (Dermal-eccrine-cylindroma) OR (Dermatitis-herpetiformis) OR (Diffuse-dermal-angiomatosis) OR (Dystrophic-epidermolysis-bullosa) OR (Elastoderma) OR (Eosinophilic-pustular-folliculitis) OR (Epidermolysis-bullosa) OR (Epidermolysis-bullosa-acquisita) OR (Epidermolysis-bullosa-simplex) OR (Epidermolysis-bullosa-simplex NEAR/3 generalized) OR (Erythema-multiforme) OR (Erythema-nodosum NEAR/3 idiopathic) OR (Erythrokeratodermia-variabilis-et-progressiva) OR (Familial-dermographism) OR (Familial-multiple-trichodiscomas) OR (Febrile-Ulceronecrotic-Mucha-Habermann-diseas*) OR (Fox-Fordyce-diseas*) OR (Frontal-fibrosing-alopecia) OR (Granuloma-annulare) OR (Granulomatous-rosacea) OR (Guttate-psoriasis) OR (Halal-Setton-Wang-syndrom*) OR (Halo-nevus) OR (Hydroa-vacciniforme) OR (Hydroa-vacciniforme NEAR/3 familial) OR (Hypertrichosis-lanuginosa NEAR/3 acquired) OR (Hypohidrotic-ectodermal-dysplasia) OR (Ichthyosis-vulgaris) OR (Ichthyosis NEAR/3 acquired) OR (Junctional-epidermolysis-bullosa-inversa) OR (Keratosis-palmoplantaris-striata-1) OR (Keratosis-palmoplantaris-striata-3) OR (Kyrle-diseas*) OR (Lichen-planopilaris) OR (Lichen-planus-pemphigoides) OR (Lichen-planus-pigmentosus) OR (Lichen-sclerosus) OR (Linear-IgA-diseas*) OR (Linear-lichen-planus) OR (Linear-scleroderma) OR (Localized-scleroderma) OR (Lupus-erythematosus-tumidus) OR (Lymphocytic-infiltrate NEAR/3 Jessner) OR (Morphea) OR (Mucous-membrane-pemphigoid) OR (Multicentric-reticulohistiocytosis) OR (Necrobiotic-xanthogranuloma) OR (Nelson-syndrom*) OR (Nephrogenic-Systemic-Fibrosis) OR (Nodular-nonsuppurative-panniculitis) OR (Palmoplantar-keratoderma) OR (Parapsoriasis) OR (Peeling-skin-syndrom*) OR (Pemphigus-vulgaris) OR (Pigmented-purpuric-dermatosis) OR (Pityriasis-lichenoides) OR (Pityriasis-lichenoides-chronica) OR (Pityriasis-lichenoides-et-varioliformis-acuta) OR (Porokeratosis NEAR/3 disseminated-superficial-actinic-1) OR (Primary-cutaneous-amyloidosis) OR (Progestogen-hypersensitivity) OR (Pseudoainhum) OR (Pseudopelade NEAR/3 Brocq) OR (Quinquaud-folliculitis-decalvans) OR (Red-skin-pigment-anomaly NEAR/3 New-Guinea) OR (Rhabdomyomatous-mesenchymal-hamartoma) OR (Scleromyxedema) OR (Sjogren-Larsson-like-syndrom*) OR (Spitz-nevus) OR (Subcorneal-pustular-dermatosis) OR (Syringocystadenoma-papilliferum) OR (Systemic-scleroderma) OR (Trichostasis-spinulosa) OR (Wells-syndrom*) OR (Xanthoma-disseminatum) OR (Xeroderma-pigmentosum NEAR/3 variant-type)):ab,ti,kw,de)

**Medline 204**

(Disability-Adjusted Life Years/ OR (DALY OR DALYs OR ((disabil*) ADJ4 (adjust*) ADJ4 (life*) ADJ4 (year*)) OR YLL OR YLLs OR ((year*) ADJ2 (life*) ADJ (lost*)) OR YLD OR YLDs OR ((year*) ADJ3 (lived) ADJ3 (disabil*))).ab,ti,kf.) **AND** (Rare Diseases/ OR Hemophilia A/ OR Hemophilia B/ OR Sarcoidosis/ OR exp Heart Defects, Congenital/ OR (((rare OR orphan) ADJ3 (diseas* OR disorder* OR syndrom* OR condition*)) OR hemophilia* OR haemophilia* OR sarcoidosis* OR besnier-boeck* OR ((congenital) ADJ3 (heart* OR cardiac*) ADJ3 (diseas*))).ab,ti,kf. OR ((Addison ADJ3 disease) OR (Autoimmune-gastrointestinal-dysmotility) OR (Autoimmune-hemolytic-anemia) OR (Autoimmune-hepatitis) OR (Autoimmune-Inner-Ear ADJ3 diseas*) OR (Autoimmune-lymphoproliferative ADJ3 syndrom*) OR (Evans ADJ3 syndrom*) OR (Warm-antibody-hemolytic-anemia) OR (Conversion-disorder) OR (Gardner-Diamond ADJ3 syndrom*) OR (Mietens-Weber ADJ3 syndrom*) OR (Presenile-dementia ADJ3 -Kraepelin) OR (15q13#3-microdeletion ADJ3 syndrom*) OR (16p11#2-deletion ADJ3 syndrom*) OR (17q23#1q23#2-microdeletion ADJ3 syndrom*) OR (1q-duplication*) OR (1q21#1-microdeletion ADJ3 syndrom*) OR (22q11#2-deletion ADJ3 syndrom*) OR (22q11#2-duplication ADJ3 syndrom*) OR (2q23#1-microdeletion ADJ3 syndrom*) OR (2q37-deletion ADJ3 syndrom*) OR (47-XXX ADJ3 syndrom*) OR (47 ADJ3 XYY ADJ3 syndrom*) OR (49 ADJ3 XXXXX ADJ3 syndrom*) OR (Cat-eye ADJ3 syndrom*) OR (Chromosome-1 ADJ3 uniparental-disomy-1q12-q21) OR (Chromosome-10p-deletion) OR (Chromosome-10p-duplication) OR (Chromosome-10q-deletion) OR (Chromosome-10q-duplication) OR (Chromosome-11p-deletion) OR (Chromosome-11p-duplication) OR (Chromosome-11q-deletion) OR (Chromosome-11q-duplication) OR (Chromosome-12p-deletion) OR (Chromosome-12p-duplication) OR (Chromosome-12q-deletion) OR (Chromosome-12q-duplication) OR (Chromosome-13q-deletion) OR (Chromosome-13q-duplication) OR (Chromosome-14q-deletion) OR (Chromosome-14q-duplication) OR (Chromosome-15q-deletion) OR (Chromosome-15q-duplication) OR (Chromosome-16-trisomy) OR (Chromosome-16p-deletion) OR (Chromosome-16p-duplication) OR (Chromosome-16q-deletion) OR (Chromosome-17p-deletion) OR (Chromosome-17p-duplication) OR (Chromosome-17q-duplication) OR (Chromosome-18p-deletion) OR (Chromosome-18p-tetrasomy) OR (Chromosome-19p-deletion) OR (Chromosome-19p-duplication) OR (Chromosome-19q-deletion) OR (Chromosome-19q-duplication) OR (Chromosome-1p-deletion) OR (Chromosome-1p-duplication) OR (Chromosome-1p36-deletion ADJ3 syndrom*) OR (Chromosome-1q-deletion) OR (Chromosome-1q21#1-duplication ADJ3 syndrom*) OR (Chromosome-20-trisomy) OR (Chromosome-20p-deletion) OR (Chromosome-20p-duplication) OR (Chromosome-20q-deletion) OR (Chromosome-20q-duplication) OR (Chromosome-21q-deletion) OR (Chromosome-21q-duplication) OR (Chromosome-22q-deletion) OR (Chromosome-2p-deletion) OR (Chromosome-2p-duplication) OR (Chromosome-2q-deletion) OR (Chromosome-2q-duplication) OR (Chromosome-2q24-microdeletion ADJ3 syndrom*) OR (Chromosome-3p-duplication) OR (Chromosome-3p- ADJ3 syndrom*) OR (Chromosome-3q-deletion) OR (Chromosome-3q-duplication) OR (Chromosome-3q29-microduplication ADJ3 syndrom*) OR (Chromosome-4p-deletion) OR (Chromosome-4p-duplication) OR (Chromosome-4q-deletion) OR (Chromosome-4q-duplication) OR (Chromosome-5p-deletion) OR (Chromosome-5p-duplication) OR (Chromosome-5q-deletion) OR (Chromosome-5q-duplication) OR (Chromosome-6p-deletion) OR (Chromosome-6p-duplication) OR (Chromosome-6q-deletion) OR (Chromosome-6q-duplication) OR (Chromosome-6q25-microdeletion ADJ3 syndrom*) OR (Chromosome-7p-deletion) OR (Chromosome-7p-duplication) OR (Chromosome-7q-deletion) OR (Chromosome-7q-duplication) OR (Chromosome-8p-deletion) OR (Chromosome-8p-duplication) OR (Chromosome-8p23#1-deletion) OR (Chromosome-8q-deletion) OR (Chromosome-8q-duplication) OR (Chromosome-9p-deletion) OR (Chromosome-9p-duplication) OR (Chromosome-9q-deletion) OR (Chromosome-9q-duplication) OR (Chromosome-Xq-duplication) OR (Diploid-triploid-mosaicism) OR (Distal-chromosome-18q-deletion ADJ3 syndrom*) OR (Emanuel ADJ3 syndrom*) OR (Kleefstra ADJ3 syndrom*) OR (Koolen-de-Vries ADJ3 syndrom*) OR (Mosaic-monosomy-18) OR (Mosaic-monosomy-22) OR (Mosaic-trisomy-13) OR (Mosaic-trisomy-14) OR (Mosaic-trisomy-22) OR (Mosaic-trisomy-7) OR (Mosaic-trisomy-8) OR (Mosaic-trisomy-9) OR (Nablus-mask-like-facial ADJ3 syndrom*) OR (Pallister-Killian-mosaic ADJ3 syndrom*) OR (Partial-deletion ADJ3 the-short-arm ADJ3 chromosome-3) OR (Partial-deletion ADJ3 Y) OR (Potocki-Shaffer ADJ3 syndrom*) OR (Proximal-chromosome-18q-deletion ADJ3 syndrom*) OR (Recombinant-chromosome-8 ADJ3 syndrom*) OR (Ring-chromosome-1) OR (Ring-chromosome-10) OR (Ring-chromosome-11) OR (Ring-chromosome-12) OR (Ring-chromosome-13) OR (Ring-chromosome-14) OR (Ring-chromosome-15) OR (Ring-chromosome-16) OR (Ring-chromosome-17) OR (Ring-chromosome-18) OR (Ring-chromosome-19) OR (Ring-chromosome-2) OR (Ring-chromosome-20) OR (Ring-chromosome-21) OR (Ring-chromosome-22) OR (Ring-chromosome-3) OR (Ring-chromosome-4) OR (Ring-chromosome-5) OR (Ring-chromosome-6) OR (Ring-chromosome-7) OR (Ring-chromosome-8) OR (Ring-chromosome-9) OR (Smith-Magenis ADJ3 syndrom*) OR (Tetrasomy-9p) OR (Tetrasomy-X) OR (Triploidy) OR (Trisomy-13) OR (Trisomy-17-mosaicism) OR (Trisomy-2-mosaicism) OR (Turner ADJ3 syndrom*) OR (Wolf-Hirschhorn ADJ3 syndrom*) OR (X-linked-susceptibility-to-autism-4) OR (Y-chromosome-infertility) OR (5q ADJ3 syndrome*) OR (Aagenaes ADJ3 syndrome*) OR (Abdominal-aortic-aneurysm) OR (Abetalipoprotein*) OR (Acatalas*) OR (Aceruloplasmin*) OR (Acquir* ADJ3 agranulocytos*) OR (Acquir* ADJ3 hemophilia) OR (Acquir* ADJ3 hemophilia-A) OR (Acquir* ADJ3 pure-red-cell-aplasia) OR (Acquir* ADJ3 Von-Willebrand ADJ3 syndrome*) OR (Adenosine-Deaminase ADJ3 deficiency) OR (Adrenocortical-carcinoma) OR (Adult-T-cell-leukemia ADJ3 lymphoma) OR (Afibrinogen*) OR (ALK-histiocytos*) OR (Alpha-thalass*-x-linked-intellectual-disability ADJ3 syndrome*) OR (AML ADJ3 myelodysplasia-related-features) OR (Anemia ADJ3 Adenosine-triphosphatase ADJ3 deficienc*) OR (An*-sideroblastic ADJ3 spinocerebellar-ataxia) OR (Aneurysm ADJ3 sinus ADJ3 Valsalva) OR (Angioimmunoblastic-T-cell-lymphoma) OR (Angioma-hereditary-neurocutaneous) OR (Angioma-serpiginosum) OR (Antiphospholipid ADJ3 syndrome*) OR (Aplasia-cut*-congenita-intestinal-lymphangiectasia) OR (Aplastic-an*) OR (Arterial-calcification ADJ3 infancy) OR (Arterial-tortuosity ADJ3 syndrome*) OR (Atransferrin*) OR (Atypical-hemolytic-uremic ADJ3 syndrome*) OR (Autosomal ADJ3 recessive*-protein-C ADJ3 deficienc*) OR (Bannayan-Riley-Ruvalcaba ADJ3 syndrome*) OR (Behcet ADJ3 disease*) OR (Beta-thalass*) OR (Blastic-plasmacytoid-dendritic-cell) OR (Bleeding ADJ3 disorder* ADJ3 P2RY12-defect) OR (Bloom ADJ3 syndrome*) OR (Blue-rubber-bleb-nevus ADJ3 syndrome*) OR (Buerger ADJ3 disease*) OR (Burkitt-lymphoma) OR (Campomelia-Cumming ADJ3 type) OR (Castleman ADJ3 disease*) OR (Chediak-Higashi ADJ3 syndrome*) OR (Chromosome-17q11#2-deletion ADJ3 syndrome*) OR (Chronic-myeloid-leuk*) OR (Chylous-ascites) OR (CLOVES ADJ3 syndrome*) OR (Cobb ADJ3 syndrome*) OR (Cold-agglutinin ADJ3 disease*) OR (Congenital-amegakaryocytic-thrombocytopenia) OR (Congenital-analbumin*) OR (Congenital-dyserythropoietic-anemia ADJ3 type-1) OR (Congenital-dyserythropoietic-anemia ADJ3 type-2) OR (Congenital-dyserythropoietic-anemia ADJ3 type-3) OR (Congenital-erythropoietic-porphyria) OR (Congenital-myasthenic ADJ3 syndrome* ADJ3 episodic-apnea) OR (Congenital-pulmonary-lymphangiectasia) OR (Congenital-thrombotic-thrombocytopenic-purpura) OR (Cutaneous-mastocytoma) OR (Cut*-laxa ADJ3 autosomal* ADJ3 recessive* ADJ3 type-1) OR (Cut*-marmorata-telangiectatica-congenita) OR (Cyclic-neutropenia) OR (Cyclic-thrombocytopenia) OR (Cystic-medial-necrosis ADJ3 aorta) OR (Dahlberg-Borer-Newcomer ADJ3 syndrome*) OR (Deafness-lymphedema-leukemia ADJ3 syndrome*) OR (Dehydrated-hereditary-stomatocytos*) OR (Diamond-Blackfan-an*) OR (Diamond-Blackfan-an*-2) OR (Diamond-Blackfan-an*-3) OR (Dysfibrinogen*) OR (Dyskeratos*-congenita) OR (Dyskeratos*-congenita ADJ3 autosomal* ADJ3 dominant*) OR (Dyskeratos*-congenita ADJ3 autosomal* ADJ3 recessive*) OR (Dyskeratos*-congenita-X-linked) OR (Ehlers-Danlos ADJ3 syndrome*-dysfibronectinemic ADJ3 type) OR (Eosinophilic-granulomatosis ADJ3 polyangiit*) OR (Erythema-elevatum-diutinum) OR (Essential-thrombocyth*) OR (Extranodal-nasal-NK ADJ3 T-cell-lymphoma) OR (Fabry ADJ3 disease*) OR (Factor-V ADJ3 deficienc*) OR (Factor-VII ADJ3 deficienc*) OR (Factor-X ADJ3 deficienc*) OR (Factor-XI ADJ3 deficienc*) OR (Factor-XII ADJ3 deficienc*) OR (Factor-XIII ADJ3 deficienc*) OR (Familial-hyperthyroidism ADJ3 mutations ADJ3 TSH-receptor) OR (Familial-LCAT ADJ3 deficienc*) OR (Familial-platelet ADJ3 disorder* ADJ3 associated-myeloid-malignancy) OR (Familial-thoracic-aortic-aneurysm ADJ3 aortic-dissection) OR (Fanconi-an*) OR (Fetal ADJ3 neonatal-alloimmune-thrombocytopenia) OR (Follicular-lymphoma) OR (Genuine-diffuse-phlebectasia) OR (Giant-cell-arterit*) OR (Giant-platelet ADJ3 syndrome*) OR (Glanzmann-thrombasthenia) OR (Glucocorticoid-remediable-aldosteronism) OR (Glutamate-formiminotransferase ADJ3 deficienc*) OR (Glycogen-storage ADJ3 disease* ADJ3 type-12) OR (Glycogen-storage ADJ3 disease* ADJ3 type-7) OR (Glycoprotein-VI ADJ3 deficienc*) OR (Goodpasture ADJ3 syndrome*) OR (Gorham* ADJ3 disease*) OR (Granulomatosis ADJ3 polyangiit*) OR (Granulomatous-slack-skin ADJ3 disease*) OR (Gray-platelet ADJ3 syndrome*) OR (Hairy-cell-leuk*) OR (Hashimoto-Pritzker ADJ3 syndrome*) OR (Heinz-body-anemias) OR (Hemangioma-thrombocytopenia ADJ3 syndrome*) OR (Hemochromatosis ADJ3 type-2) OR (Hemochromatosis ADJ3 type-3) OR (Hemochromatosis ADJ3 type-4) OR (Hemoglobin-C ADJ3 disease*) OR (Hemoglobin-E ADJ3 disease*) OR (Hemoglobin-SC ADJ3 disease*) OR (Hemoglobin-SE ADJ3 disease*) OR (Hemolytic-an*-lethal-congenital-nonspherocytic ADJ3 genital ADJ3 other-abnormalities) OR (Hemolytic-uremic ADJ3 syndrome*) OR (Hemophilia-A) OR (Hemophilia-B) OR (Hemorrhagic-shock ADJ3 encephalopathy ADJ3 syndrome*) OR (Hennekam ADJ3 syndrome*) OR (Henoch-Schonlein-purpura) OR (Heparin-induced-thrombocytopenia) OR (Hereditary-antithrombin ADJ3 deficienc*) OR (Hereditary-elliptocytos*) OR (Hereditary-folate-malabsorption) OR (Hereditary-hemorrhagic-telangiectasia) OR (Hereditary-hemorrhagic-telangiectasia ADJ3 type-2) OR (Hereditary-hemorrhagic-telangiectasia ADJ3 type-3) OR (Hereditary-hemorrhagic-telangiectasia ADJ3 type-4) OR (Hereditary-lymphedema ADJ3 type-II) OR (Hereditary-methemoglobin*) OR (Hereditary-paraganglioma-pheochromocytoma) OR (Hereditary-spherocytos*) OR (Hermansky-Pudlak ADJ3 syndrome*-2) OR (High-molecular-weight-kininogen ADJ3 deficienc*) OR (Histiocytos*-lymphadenopathy ADJ3 syndrome*) OR (Hoyeraal-Hreidarsson ADJ3 syndrome*) OR (Hypercoagulability ADJ3 syndrome* ADJ3 glycosylphosphatidylinositol ADJ3 deficienc*) OR (Hypereosinophilic ADJ3 syndrome*) OR (Hypersensitivity-vasculit*) OR (Hypocomplementemic-urticarial-vasculit*) OR (Hypofibrinogenemia ADJ3 familial*) OR (Hypotrichos*-lymphedema-telangiectasia ADJ3 syndrome*) OR (Idiopathic-thrombocytopenic-purpura) OR (Imerslund-Grasbeck ADJ3 syndrome*) OR (Internal-carotid-agenes*) OR (Intrinsic-factor ADJ3 deficienc*) OR (Iron-refractory-iron ADJ3 deficienc*-an*) OR (Jacobsen ADJ3 syndrome*) OR (Juvenile-myelomonocytic-leuk*) OR (Juvenile-temporal-arterit*) OR (Kanzaki ADJ3 disease*) OR (Kaposi-sarcoma) OR (Kaposiform-Hemangioendothelioma) OR (Kaposiform-lymphangiomatos*) OR (Kawasaki ADJ3 disease*) OR (Klippel-Trenaunay ADJ3 syndrome*) OR (Langerhans-cell-sarcoma) OR (Lesch-Nyhan ADJ3 syndrome*) OR (Liddle ADJ3 syndrome*) OR (Lissencephaly-2) OR (Loeys-Dietz ADJ3 syndrome*) OR (Loeys-Dietz ADJ3 syndrome* ADJ3 type-1) OR (Loeys-Dietz ADJ3 syndrome* ADJ3 type-2) OR (Loeys-Dietz ADJ3 syndrome* ADJ3 type-3) OR (Loeys-Dietz ADJ3 syndrome* ADJ3 type-4) OR (Lymphedema ADJ3 cerebral-arteriovenous-anomaly) OR (Lymphedema-distichiasis ADJ3 syndrome*) OR (Lymphomatoid-papulos*) OR (Maffucci ADJ3 syndrome*) OR (Majeed ADJ3 syndrome*) OR (Mantle-cell-lymphoma) OR (McLeod-neuroacanthocytosis ADJ3 syndrome*) OR (Megalencephaly-capillary-malformation ADJ3 syndrome*) OR (Megaloblastic-anemia ADJ3 dihydrofolate-reductase ADJ3 deficienc*) OR (Methemoglobin*-beta-globin ADJ3 type) OR (Methylcobalamin ADJ3 deficienc*-cbl-G ADJ3 type) OR (Microcystic-lymphatic ADJ3 malformation*) OR (Microscopic-polyangiit*) OR (Milroy ADJ3 disease*) OR (Mitochondrial-myopathy ADJ3 sideroblastic-an*) OR (MPI-CDG-CDG-Ib) OR (Multicentric-Castleman ADJ3 disease*) OR (Multifocal-lymphangioendotheliomatosis ADJ3 thrombocytopenia) OR (Multiple-myeloma) OR (Multisystemic-smooth-muscle-dysfunction ADJ3 syndrome*) OR (Myelodysplastic ADJ3 syndrome* ADJ3 single-lineage-dysplasia) OR (Myelodysplastic ADJ3 syndrome*s) OR (Myeloid-sarcoma) OR (MYH9-related-thrombocytopenia) OR (Neonatal-hemochromatos*) OR (Neutropenia ADJ3 chronic* ADJ3 familial*) OR (Neutropenia-lethal-congenital ADJ3 eosinophilia) OR (Non-involuting-congenital-hemangioma) OR (Nonspherocytic-hemolytic-anemia ADJ3 hexokinase ADJ3 deficienc*) OR (Noonan ADJ3 syndrome*) OR (Orotic-aciduria ADJ3 type-1) OR (Overhydrated-hereditary-stomatocytos*) OR (Par*-Trousseau-thrombocytopenia) OR (Parkes-Weber ADJ3 syndrome*) OR (Paroxysmal-cold-hemoglobinuria) OR (Paroxysmal-nocturnal-hemoglobinuria) OR (Pearson ADJ3 syndrome*) OR (PEHO ADJ3 syndrome*) OR (PHACE ADJ3 syndrome*) OR (Pheochromocytoma) OR (Phosphoglycerate-kinase ADJ3 deficienc*) OR (Plasmablastic-lymphoma) OR (Plasminogen-activator-inhibitor ADJ3 type-1 ADJ3 deficienc*) OR (Platelet-storage-pool ADJ3 deficienc*) OR (Plummer-Vinson ADJ3 syndrome*) OR (POEMS ADJ3 syndrome*) OR (Poikiloderma ADJ3 neutropenia) OR (Polycyth*-vera) OR (Prekallikrein ADJ3 deficienc* ADJ3 congenital*) OR (Primary-angiitis ADJ3 central-nervous-system) OR (Primary-central-nervous-system-lymphoma) OR (Primary ADJ3 familial* ADJ3 congenital-polycyth*) OR (Primary-intestinal-lymphangiectasia) OR (Primary-myelofibros*) OR (Primary-release ADJ3 disorder* ADJ3 platelets) OR (Prolidase ADJ3 deficienc*) OR (Protein-S ADJ3 deficienc*) OR (Proteus ADJ3 syndrome*) OR (Prothrombin ADJ3 deficienc*) OR (Pseudo-Von-Willebrand ADJ3 disease*) OR (Pseudohyperkal*-Cardiff) OR (Pseudoxanthoma-elasticum) OR (Pulmonary-arterio-veinous-fistula) OR (Pulmonary-atresia ADJ3 intact-ventricular-septum) OR (Pulmonary-vein-stenos*) OR (Pyropoikilocytos*-hereditary) OR (Pyruvate-kinase ADJ3 deficienc*) OR (Quebec-platelet ADJ3 disorder*) OR (Red-cell-phospholipid-defect ADJ3 hemolys*) OR (Revesz ADJ3 syndrome*) OR (Reynolds ADJ3 syndrome*) OR (Rh ADJ3 deficienc* ADJ3 syndrome*) OR (Rosai-Dorfman ADJ3 disease*) OR (Rotor ADJ3 syndrome*) OR (Scott ADJ3 syndrome*) OR (Severe ADJ3 congenital*-neutropenia ADJ3 autosomal* ADJ3 dominant*) OR (Severe ADJ3 congenital*-neutropenia ADJ3 autosomal* ADJ3 recessive*-3) OR (Sezary ADJ3 syndrome*) OR (Shwachman-Diamond ADJ3 syndrome*) OR (Sickle-beta-thalass*) OR (Sickle-cell--hemoglobin-D ADJ3 disease*) OR (Sickle-cell-an*) OR (Sideroblastic-an*-pyridoxine-refractory ADJ3 autosomal* ADJ3 recessive*) OR (Sideroblastic-an*-pyridoxine-responsive ADJ3 autosomal* ADJ3 recessive*) OR (Slow-channel ADJ3 congenital*-myasthenic ADJ3 syndrome*) OR (Sneddon ADJ3 syndrome*) OR (Sturge-Weber ADJ3 syndrome*) OR (Supraumbilical-midabdominal-raphe ADJ3 facial-cavernous-hemangiomas) OR (Supravalvular-aortic-stenos*) OR (Susac ADJ3 syndrome*) OR (Swyer ADJ3 syndrome*) OR (Systemic-mastocytos*) OR (T-cell-large-granular-lymphocyte-leuk*) OR (T-cell ADJ3 histiocyte-rich-large-B-cell-lymphoma) OR (Takayasu-arterit*) OR (TAR ADJ3 syndrome*) OR (Thalass*) OR (Thiamine-responsive-megaloblastic-anemia ADJ3 syndrome*) OR (Thoracolaryngopelvic-dysplasia) OR (Thrombocytopathy-asplenia-mios*) OR (Thrombocytopenia-2) OR (Thrombocytopenia ADJ3 elevated-serum-IgA ADJ3 renal ADJ3 disease*) OR (Thrombomodulin-anomalies ADJ3 familial*) OR (Thrombotic-thrombocytopenic-purpura-acquired) OR (Transient-erythroblastopenia ADJ3 childhood) OR (Transient-myeloproliferative ADJ3 syndrome*) OR (Triosephosphate-isomerase ADJ3 deficienc*) OR (Tuberous-scleros*-complex) OR (Tufted-angioma) OR (Twin-to-twin-transfusion ADJ3 syndrome*) OR (Type-1-plasminogen ADJ3 deficienc*) OR (Unicentric-Castleman ADJ3 disease*) OR (Vascular-Ehlers-Danlos ADJ3 syndrome*) OR (Vein ADJ3 Galen-aneurysm) OR (Von-Hippel-Lindau ADJ3 disease*) OR (White-platelet ADJ3 syndrome*) OR (Williams ADJ3 syndrome*) OR (Wiskott-Aldrich ADJ3 syndrome*) OR (WT-limb-blood ADJ3 syndrome*) OR (Wyburn-Mason ADJ3 syndrome*) OR (X-linked-sideroblastic-an*) OR (X-linked-thrombocytopenia) OR (Yellow-nail ADJ3 syndrome*) OR (Parastremmatic-dwarfism) OR (Bilateral-parasagittal-parieto-occipital-polymicrogyria) OR (11-beta-hydroxylase ADJ3 deficien*) OR (12q14 ADJ3 microdeletion* ADJ3 syndrom*) OR (15q11#2 ADJ3 microdeletion*) OR (15q24 ADJ3 microdeletion* ADJ3 syndrom*) OR (16p13#11-microduplication ADJ3 syndrom*) OR (16q24#3 ADJ3 microdeletion* ADJ3 syndrom*) OR (17-alpha-hydroxylase ADJ3 deficien*) OR (17-beta-hydroxysteroid-dehydrogenase-3 ADJ3 deficien*) OR (17q12-deletion ADJ3 syndrom*) OR (17q12 ADJ3 duplicat*) OR (18-Hydroxylase ADJ3 deficien*) OR (19p13#12 ADJ3 microdeletion* ADJ3 syndrom*) OR (1q44 ADJ3 microdeletion* ADJ3 syndrom*) OR (2-methylbutyryl-CoA-dehydrogenase ADJ3 deficien*) OR (20p12#3 ADJ3 microdeletion* ADJ3 syndrom*) OR (22q13#3-deletion ADJ3 syndrom*) OR (2p15p16#1 ADJ3 microdeletion* ADJ3 syndrom*) OR (3-alpha-hydroxyacyl-CoA-dehydrogenase ADJ3 deficien*) OR (3-beta-hydroxysteroid-dehydrogenase ADJ3 deficien*) OR (3-Hydroxyisobutyric-aciduria) OR (3-methylcrotonyl-CoA-carboxylase ADJ3 deficien*) OR (3-methylglutaconyl-CoA-hydratase ADJ3 deficien*-) OR (AUH ADJ3 defect*) OR (3M ADJ3 syndrom*) OR (3MC ADJ3 syndrom*) OR (3q29 ADJ3 microdeletion* ADJ3 syndrom*) OR (46 ADJ3 XX-testicular-disorder ADJ3 sex-development) OR (48 ADJ3 XXXY ADJ3 syndrom*) OR (48 ADJ3 XYYY) OR (49 ADJ3 XXXXY ADJ3 syndrom*) OR (49 ADJ3 XXXYY ADJ3 syndrom*) OR (5-alpha-reductase ADJ3 deficien*) OR (5-oxoprolinase ADJ3 deficien*) OR (5q14#3 ADJ3 microdeletion* ADJ3 syndrom*) OR (6-pyruvoyl-tetrahydropterin-synthase ADJ3 deficien*) OR (7q11#23 ADJ3 duplicat* ADJ3 syndrom*) OR (8p23#1 ADJ3 duplicat* ADJ3 syndrom*) OR (8q12-microduplication ADJ3 syndrom*) OR (Aarskog ADJ3 syndrom*) OR (Ablepharon-macrostomia ADJ3 syndrom*) OR (ABri-amyloidosis) OR (Abruzzo-Erickson ADJ3 syndrom*) OR (Absence ADJ3 fingerprints-congenital-milia) OR (Absence ADJ3 gluteal-muscle) OR (Absence ADJ3 Tibia) OR (Absence ADJ3 tibia ADJ3 polydactyly) OR (Absent-breasts ADJ3 nipples) OR (Absent-patella) OR (Acalvaria) OR (Acanthosis-nigricans-muscle-cramps-acral-enlargement) OR (Acardia) OR (Accessory-deep-peroneal-nerve) OR (Accessory-pancreas) OR (Achalasia-microcephaly ADJ3 syndrom*) OR (Achard ADJ3 syndrom*) OR (Acheiropody) OR (Achondrogenesis) OR (Achondrogenesis-type-1A) OR (Achondrogenesis-type-1B) OR (Achondrogenesis-type-2) OR (Achondroplasia) OR (Acitretin-embryopathy) OR (Acral-dysostosis-dyserythropoiesis ADJ3 syndrom*) OR (Acral-peeling-skin ADJ3 syndrom*) OR (Acro-pectoro-renal-field ADJ3 defect*) OR (Acrocallosal ADJ3 syndrom* ADJ3 Schinzel) OR (Acrocapitofemoral-dysplasia) OR (Acrocephalopolydactyly) OR (Acrodermatitis-enteropathica) OR (Acrodysostosis) OR (Acrodysplasia-scoliosis) OR (Acrodysplasia ADJ3 ossification-abnormalities ADJ3 short-stature ADJ3 fibular-hypoplasia) OR (Acrofacial-dysostosis-Catania) OR (Acrofacial-dysostosis-Palagonia) OR (Acrofacial-dysostosis-Rodriguez) OR (Acrofrontofacionasal-dysostosis ADJ3 syndrom*) OR (Acrogeria ADJ3 Gottron) OR (Acrokeratoelastoidosis ADJ3 Costa) OR (Acromegaloid-facial-appearance ADJ3 syndrom*) OR (Acromegaloid-features ADJ3 overgrowth ADJ3 cleft-palate ADJ3 hernia) OR (Acromegaloid-hypertrichosis ADJ3 syndrom*) OR (Acromelic-frontonasal-dysostosis) OR (Acromesomelic-dysplasia-Campailla-Martinelli) OR (Acromesomelic-dysplasia-Hunter-Thompson) OR (Acromesomelic-dysplasia-Maroteaux) OR (Acromicric-dysplasia) OR (Acroosteolysis-dominant) OR (Acropectoral ADJ3 syndrom*) OR (Acropectorovertebral-dysplasia-F-form) OR (Acrorenal-mandibular ADJ3 syndrom*) OR (ACTH-independent-macronodular-adrenal-hyperplasia) OR (Adactylia-unilateral) OR (Adams-Oliver ADJ3 syndrom*) OR (ADCY5-related-dyskinesia) OR (Adenine-phosphoribosyltransferase ADJ3 deficien*) OR (Adenosine-deaminase ADJ3 deficien*) OR (Adenosine-monophosphate-deaminase-1 ADJ3 deficien*) OR (Adenylosuccinase ADJ3 deficien*) OR (Adermatoglyphia) OR (Adrenomyeloneuropathy) OR (Adrenomyodystroph*) OR (Adult-polyglucosan-body ADJ3 diseas*) OR (ADULT ADJ3 syndrom*) OR (Adult-onset-nemaline-myopathy) OR (Adult-onset-vitelliform-macular-dystroph*) OR (Advanced-sleep-phase ADJ3 syndrom* ADJ3 familial) OR (Agammaglobulinemia ADJ3 microcephaly ADJ3 -severe-dermatitis) OR (Agammaglobulinemia ADJ3 non-Bruton) OR (Agenesis ADJ3 dorsal-pancreas) OR (Agnathia-microstomia-synotia) OR (Aicardi ADJ3 syndrom*) OR (Aicardi-Goutieres ADJ3 syndrom*) OR (Akesson ADJ3 syndrom*) OR (Al-Gazali-Aziz-Salem ADJ3 syndrom*) OR (Al-Gazali-Khidr-Prem-Chandran ADJ3 syndrom*) OR (Al-Gazali-Sabrinathan-Nair ADJ3 syndrom*) OR (Al-Gazali ADJ3 syndrom*) OR (Al-Gazali-Donnai-Mueller ADJ3 syndrom*) OR (Alagille ADJ3 syndrom*) OR (Alaninuria ADJ3 microcephaly ADJ3 dwarfism ADJ3 enamel-hypoplasia ADJ3 diabetes-mellitus) OR (Albinism) OR (Albinism-deafness ADJ3 syndrom*) OR (Albinism-ocular-late-onset-sensorineural-deafness) OR (Albright ADJ3 -hereditary-osteodystroph*) OR (Alexander ADJ3 diseas*) OR (ALG1-CDG-) OR (CDG-Ik) OR (ALG11-CDG-) OR (CDG-Ip) OR (ALG12-CDG-) OR (CDG-Ig) OR (ALG13-CDG) OR (ALG2-CDG-) OR (CDG-Ii) OR (ALG3-CDG-) OR (CDG-Id) OR (ALG6-CDG-) OR (CDG-Ic) OR (ALG8-CDG-) OR (CDG-Ih) OR (ALG9-CDG-) OR (CDG-IL) OR (Alkaptonuria) OR (Allain-Babin-Demarquez ADJ3 syndrom*) OR (Allan-Herndon-Dudley ADJ3 syndrom*) OR (Alopecia-epilepsy-oligophrenia ADJ3 syndrom* ADJ3 Moynahan) OR (Alopecia-intellectual-disability ADJ3 syndrom*-2) OR (Alopecia-totalis) OR (Alopecia-universalis) OR (Alopecia-universalis-onychodystroph*-vitiligo) OR (Alopecia ADJ3 epilepsy ADJ3 pyorrhea ADJ3 mental-subnormality) OR (Alopecia-contractures-dwarfism-intellectual-disability ADJ3 syndrom*) OR (Alopecia-intellectual-disability ADJ3 syndrom*) OR (Alpers ADJ3 syndrom*) OR (Alpha-1-antitrypsin ADJ3 deficien*) OR (Alpha-ketoglutarate-dehydrogenase ADJ3 deficien*) OR (Alpha-mannosidosis) OR (Alport ADJ3 syndrom*) OR (Alstrom ADJ3 syndrom*) OR (Alternating-hemiplegia ADJ3 childhood) OR (Alveolar-capillary-dysplasia) OR (Amaurosis-congenita-cone-rod-type ADJ3 congenital-hypertrichosis) OR (Ambras ADJ3 syndrom*) OR (Amelogenesis-imperfecta) OR (Amelogenesis-imperfecta-hypoplastic ADJ3 hypomaturation-X-linked-1) OR (Amelogenesis-imperfecta-local-hypoplastic) OR (Amelogenesis-imperfecta-nephrocalcinosis) OR (Ameloonychohypohidrotic ADJ3 syndrom*) OR (Amino-aciduria ADJ3 mental ADJ3 deficien* ADJ3 dwarfism ADJ3 muscular-dystroph* ADJ3 osteoporosis ADJ3 acidosis) OR (Aminoacylase-1 ADJ3 deficien*) OR (Aminolevulinate-dehydratase ADJ3 deficien*-porphyria) OR (Amish-lethal-microcephaly) OR (Amish-Nemaline-Myopathy) OR (Amniotic-band ADJ3 syndrom*) OR (Amyloidosis-corneal) OR (Amyloidosis ADJ3 gingiva ADJ3 conjunctiva ADJ3 intellectual-disability) OR (Amyotonia-congenita) OR (Anal-sphincter-dysplasia) OR (Anauxetic-dysplasia) OR (Andermann ADJ3 syndrom*) OR (Andersen-Tawil ADJ3 syndrom*) OR (Anencephaly) OR (Angel-shaped-phalangoepiphyseal-dysplasia) OR (Angelman ADJ3 syndrom*) OR (Aniridia---ptosis---intellectual-disability---familial-obesity) OR (Aniridia-absent-patella) OR (Aniridia-renal-agenesis-psychomotor-retardation) OR (Ankyloblepharon-filiforme-adnatum-cleft-palate) OR (Ankyloblepharon-filiforme-imperforate-anus) OR (Ankyloblepharon-ectodermal ADJ3 defect*s-cleft-lip ADJ3 palate ADJ3 syndrom*) OR (Ankylosing-vertebral-hyperostosis ADJ3 tylosis) OR (Ankylosis ADJ3 teeth) OR (Annular-pancreas) OR (Anodontia) OR (Anomalous-origin ADJ3 right-pulmonary-artery-familial) OR (Anonychia-ectrodactyly) OR (Anonychia-onychodystroph* ADJ3 brachydactyly-type-B ADJ3 ectrodactyly) OR (Anonychia-onychodystroph* ADJ3 hypoplasia-) OR (Anonychia-onychodystroph*-absence ADJ3 distal-phalanges) OR (Anophthalmia-plus ADJ3 syndrom*) OR (Anophthalmos ADJ3 limb-anomalies) OR (Anorchia) OR (Antecubital-pterygium) OR (Anterior-segment-dysgenesis) OR (Antley-Bixler ADJ3 syndrom*) OR (Aortic-arch-anomaly---peculiar-facies---intellectual-disability) OR (Aortic-coarctation) OR (Aortopulmonary-window) OR (Apert ADJ3 syndrom*) OR (Aphalangia-partial ADJ3 syndactyly ADJ3 duplication ADJ3 metatarsal-IV) OR (Aplasia-cutis-congenita) OR (Aplasia-cutis-congenita ADJ3 limbs-recessive) OR (Arachnodactyly---intellectual-disability---dysmorphism) OR (Arachnoid-cysts) OR (AREDYLD) OR (Arginase ADJ3 deficien*) OR (Argininosuccinic-aciduria) OR (Arhinia-choanal-atresia-microphthalmia) OR (Aromatase ADJ3 deficien*) OR (Aromatase-excess ADJ3 syndrom*) OR (Aromatic-L-amino-acid-decarboxylase ADJ3 deficien*) OR (Arrhinia) OR (Arthrochalasia-Ehlers-Danlos ADJ3 syndrom*) OR (Arthrogryposis ADJ3 ectodermal-dysplasia) OR (Arthrogryposis-epileptic-seizures-migrational-brain-disorder) OR (Arthrogryposis-multiplex-congenita-neurogenic) OR (Arthrogryposis-multiplex-congenita-whistling-face) OR (Arthrogryposis-multiplex-congenita ADJ3 distal ADJ3 X-linked) OR (Arthrogryposis-renal-dysfunction-cholestasis ADJ3 syndrom*) OR (Arthrogryposis ADJ3 ectodermal-dysplasia ADJ3 cleft ADJ3 -developmental-delay) OR (Arthrogryposis-like-hand-anomaly ADJ3 sensorineural-deafness) OR (Arts ADJ3 syndrom*) OR (Ascher ADJ3 syndrom*) OR (Aspartylglycosaminuria) OR (Asternia) OR (Ataxia---hypogonadism---choroidal-dystroph*) OR (Ataxia-telangiectasia) OR (Ataxia ADJ3 oculomotor-apraxia-type-1) OR (Ataxia ADJ3 Oculomotor-Apraxia-Type-2) OR (Ataxia ADJ3 oculomotor-apraxia-type-4) OR (Ataxia ADJ3 vitamin-E ADJ3 deficien*) OR (Atelosteogenesis-type-1) OR (Atelosteogenesis-type-2) OR (Atelosteogenesis-type-3) OR (Atkin ADJ3 syndrom*) OR (Atresia ADJ3 small-intestine) OR (Atrial-myxoma ADJ3 familial) OR (Atrial-septal ADJ3 defect*-coronary-sinus) OR (Atrial-septal ADJ3 defect*-ostium-primum) OR (Atrial-septal ADJ3 defect*-sinus-venosus) OR (Atypical-Gaucher ADJ3 diseas*-due-to-saposin-C ADJ3 deficien*) OR (Atypical-Rett ADJ3 syndrom*) OR (Atypical-Werner ADJ3 syndrom*) OR (Auralcephalosyndactyly) OR (Auriculo-condylar ADJ3 syndrom*) OR (Auriculoosteodysplasia) OR (Ausems-Wittebol-Post-Hennekam ADJ3 syndrom*) OR (Autism ADJ3 port-wine-stain) OR (Autoimmune-lymphoproliferative ADJ3 syndrom*-due-to-CTLA4-haploinsuffiency) OR (Autoimmune-polyglandular ADJ3 syndrom*-type-1) OR (Autoimmune-polyglandular ADJ3 syndrom*-type-2) OR (Autosomal-dominant-Alport ADJ3 syndrom*) OR (Autosomal-dominant-centronuclear-myopathy) OR (Autosomal-dominant-cerebellar-ataxia ADJ3 deafness ADJ3 -narcolepsy) OR (Autosomal-dominant-Charcot-Marie-Tooth ADJ3 diseas*-type-2 ADJ3 giant-axons) OR (Autosomal-dominant-deafness-onychodystroph* ADJ3 syndrom*) OR (Autosomal-dominant-distal-renal-tubular-acidosis) OR (Autosomal-dominant-hyper-IgE ADJ3 syndrom*) OR (Autosomal-dominant-intermediate-Charcot-Marie-Tooth ADJ3 diseas*-type-A) OR (Autosomal-dominant-intermediate-Charcot-Marie-Tooth ADJ3 diseas*-type-B) OR (Autosomal-dominant-intermediate-Charcot-Marie-Tooth ADJ3 diseas*-type-C) OR (Autosomal-dominant-intermediate-Charcot-Marie-Tooth ADJ3 diseas*-type-D) OR (Autosomal-dominant-intermediate-Charcot-Marie-Tooth ADJ3 diseas*-type-E) OR (Autosomal-dominant-intermediate-Charcot-Marie-Tooth ADJ3 diseas*-type-F) OR (Autosomal-dominant-leukodystroph* ADJ3 autonomic ADJ3 diseas*) OR (Autosomal-dominant-multiple-pterygium ADJ3 syndrom*) OR (Autosomal-dominant-neuronal-ceroid-lipofuscinosis-4B) OR (Autosomal-dominant-nocturnal-frontal-lobe-epilepsy) OR (Autosomal-dominant-non-syndromic-intellectual-disability) OR (Autosomal-dominant-optic-atrophy ADJ3 cataract) OR (Autosomal-dominant-optic-atrophy-plus ADJ3 syndrom*) OR (Autosomal-dominant-palmoplantar-keratoderma ADJ3 congenital-alopecia) OR (Autosomal-dominant-partial-epilepsy ADJ3 auditory-features) OR (Autosomal-dominant-pseudohypoaldosteronism-type-1) OR (Autosomal-dominant-spinal-muscular-atrophy ADJ3 lower-extremity-predominant-1) OR (Autosomal-dominant-spondyloepiphyseal-dysplasia-tarda) OR (Autosomal-dominant-tubulointerstitial-kidney ADJ3 diseas*-due-to-REN-mutations) OR (Autosomal-dominant-tubulointerstitial-kidney ADJ3 diseas*-due-to-UMOD-mutations) OR (Autosomal-dominant-vitreoretinochoroidopathy) OR (Autosomal-erythropoietic-protoporphyria) OR (Autosomal-recessive-Alport ADJ3 syndrom*) OR (Autosomal-recessive-axonal-neuropathy ADJ3 neuromyotonia) OR (Autosomal-recessive-candidiasis-familial-chronic-mucocutaneous) OR (Autosomal-recessive-centronuclear-myopathy) OR (Autosomal-recessive-Charcot-Marie-Tooth ADJ3 diseas* ADJ3 hoarseness) OR (Autosomal-recessive-distal-osteolysis ADJ3 syndrom*) OR (Autosomal-recessive-early-onset-inflammatory-bowel ADJ3 diseas*) OR (Autosomal-recessive-intermediate-Charcot-Marie-Tooth ADJ3 diseas*-type-A) OR (Autosomal-recessive-intermediate-Charcot-Marie-Tooth ADJ3 diseas*-type-B) OR (Autosomal-recessive-neuronal-ceroid-lipofuscinosis-4A) OR (Autosomal-recessive-palmoplantar-keratoderma ADJ3 congenital-alopecia) OR (Autosomal-recessive-polycystic-kidney ADJ3 diseas*) OR (Autosomal-recessive-primary-microcephaly) OR (Autosomal-recessive-pseudohypoaldosteronism-type-1) OR (Autosomal-recessive-spastic-ataxia-4) OR (Autosomal-recessive-spastic-paraplegia-type-49) OR (Autosomal-recessive-spinocerebellar-ataxia-9) OR (Axenfeld-Rieger ADJ3 syndrom*) OR (Axial-mesodermal-dysplasia-spectrum) OR (Axial-spondylometaphyseal-dysplasia) OR (Ayazi ADJ3 syndrom*) OR (B4GALT1-CDG-) OR (CDG-IId) OR (Baetz-Greenwalt ADJ3 syndrom*) OR (Bagatelle-Cassidy ADJ3 syndrom*) OR (Baller-Gerold ADJ3 syndrom*) OR (Bamforth ADJ3 syndrom*) OR (Bangstad ADJ3 syndrom*) OR (Banki ADJ3 syndrom*) OR (Bantu-siderosis) OR (BAP1-tumor-predisposition ADJ3 syndrom*) OR (Baraitser-Winter ADJ3 syndrom*) OR (Barakat ADJ3 syndrom*) OR (Barber-Say ADJ3 syndrom*) OR (Bardet-Biedl ADJ3 syndrom*) OR (Bardet-Biedl ADJ3 syndrom*-1) OR (Bardet-Biedl ADJ3 syndrom*-10) OR (Bardet-Biedl ADJ3 syndrom*-11) OR (Bardet-Biedl ADJ3 syndrom*-12) OR (Bardet-Biedl ADJ3 syndrom*-2) OR (Bardet-Biedl ADJ3 syndrom*-3) OR (Bardet-Biedl ADJ3 syndrom*-4) OR (Bare-lymphocyte ADJ3 syndrom*-2) OR (Barraquer-Simons ADJ3 syndrom*) OR (Barth ADJ3 syndrom*) OR (Bartter ADJ3 syndrom*-type-3) OR (Bartter ADJ3 syndrom*-type-4) OR (Battaglia-Neri ADJ3 syndrom*) OR (Bazex-Dupre-Christol ADJ3 syndrom*) OR (Beare-Stevenson-cutis-gyrata ADJ3 syndrom*) OR (Becker-muscular-dystroph*) OR (Becker-nevus ADJ3 syndrom*) OR (Beckwith-Wiedemann ADJ3 syndrom*) OR (Beemer-Ertbruggen ADJ3 syndrom*) OR (Behr ADJ3 syndrom*) OR (Benallegue-Lacete ADJ3 syndrom*) OR (Benign-essential-blepharospasm) OR (Benign-familial-neonatal-epilepsy) OR (Benign-familial-neonatal-infantile-seizures) OR (Benign-hereditary-chorea) OR (Berk-Tabatznik ADJ3 syndrom*) OR (Best-vitelliform-macular-dystroph*) OR (Beta-ketothiolase ADJ3 deficien*) OR (Beta-Propeller-Protein-Associated-Neurodegeneration) OR (Bethlem-myopathy) OR (Beukes-familial-hip-dysplasia) OR (Biemond ADJ3 syndrom*) OR (Biemond ADJ3 syndrom*-2) OR (Bietti-crystalline-corneoretinal-dystroph*) OR (Bifid-nose) OR (Bifid-nose ADJ3 anorectal ADJ3 renal-anomalies) OR (Bilateral-frontal-polymicrogyria) OR (Bilateral-frontoparietal-polymicrogyria) OR (Bilateral-generalized-polymicrogyria) OR (Bilateral-perisylvian-polymicrogyria) OR (Bile-acid-synthesis ADJ3 defect* ADJ3 congenital ADJ3 4) OR (Biliary-atresia) OR (Biotin-thiamine-responsive-basal-ganglia ADJ3 diseas*) OR (Biotinidase ADJ3 deficien*) OR (Birk-Barel ADJ3 syndrom*) OR (Birt-Hogg-Dube ADJ3 syndrom*) OR (Bixler-Christian-Gorlin ADJ3 syndrom*) OR (Bjornstad ADJ3 syndrom*) OR (Blau ADJ3 syndrom*) OR (Blepharo-cheilo-odontic ADJ3 syndrom*) OR (Blepharonasofacial-malformation ADJ3 syndrom*) OR (Blepharophimosis ADJ3 ptosis ADJ3 syndactyly ADJ3 -short-stature) OR (Blepharoptosis-myopia-ectopia-lentis) OR (Blount ADJ3 diseas*) OR (Blue-cone-monochromatism) OR (Blue-diaper ADJ3 syndrom*) OR (BOD ADJ3 syndrom*) OR (Bohring-Opitz ADJ3 syndrom*) OR (Bone-dysplasia-Azouz) OR (Bone-dysplasia-lethal-Holmgren) OR (Book ADJ3 syndrom*) OR (Boomerang-dysplasia) OR (BOR-Duane-hydrocephalus-contiguous-gene ADJ3 syndrom*) OR (Borjeson-Forssman-Lehmann ADJ3 syndrom*) OR (Bork-Stender-Schmidt ADJ3 syndrom*) OR (Bowen-Conradi ADJ3 syndrom*) OR (Bowing ADJ3 legs ADJ3 anterior ADJ3 dwarfism) OR (Boylan-Dew-Greco ADJ3 syndrom*) OR (Brachioskeletogenital ADJ3 syndrom*) OR (Brachycephalofrontonasal-dysplasia) OR (Brachydactylous-dwarfism-Mseleni) OR (Brachydactyly-elbow-wrist-dysplasia) OR (Brachydactyly-long-thumb) OR (Brachydactyly-Mononen) OR (Brachydactyly-preaxial ADJ3 hallux-varus ADJ3 thumb-abduction) OR (Brachydactyly-tibial-hypoplasia) OR (Brachydactyly-type-A1) OR (Brachydactyly-type-A2) OR (Brachydactyly-type-A3) OR (Brachydactyly-type-A4) OR (Brachydactyly-type-A5) OR (Brachydactyly-type-A6) OR (Brachydactyly-type-A7) OR (Brachydactyly-type-B) OR (Brachydactyly-type-C) OR (Brachydactyly-type-E) OR (Brachydactyly-types-B ADJ3 E-combined) OR (Brachydactyly ADJ3 hypertension) OR (Brachydactyly-mesomelia-intellectual-disability-heart ADJ3 defect*s ADJ3 syndrom*) OR (Brachyolmia-type-3) OR (Brachyphalangy ADJ3 polydactyly ADJ3 -tibial-aplasia) OR (Brachyphalangy ADJ3 polydactyly ADJ3 -tibial-hypoplasia) OR (Bradyopsia) OR (Brain-dopamine-serotonin-vesicular-transport ADJ3 diseas*) OR (Brain-lung-thyroid ADJ3 syndrom*) OR (Branchial-arch ADJ3 syndrom*-X-linked) OR (Branchiooculofacial ADJ3 syndrom*) OR (Branchiootic ADJ3 syndrom*) OR (Branchiootorenal ADJ3 syndrom*) OR (BRCA1-hereditary-breast ADJ3 ovarian-cancer ADJ3 syndrom*) OR (BRCA2-hereditary-breast ADJ3 ovarian-cancer ADJ3 syndrom*) OR (Brittle-cornea ADJ3 syndrom*) OR (Brody-myopathy) OR (Bronchogenic-cyst) OR (Bronchopulmonary-dysplasia) OR (Brooks-Wisniewski-Brown ADJ3 syndrom*) OR (Bruck ADJ3 syndrom*-1) OR (Bruck ADJ3 syndrom*-2) OR (Brugada ADJ3 syndrom*) OR (Bullous-dystroph*-hereditary-macular) OR (Buschke-Ollendorff ADJ3 syndrom*) OR (C ADJ3 syndrom*) OR (C1q ADJ3 deficien*) OR (Cabezas ADJ3 syndrom*) OR (CAD-CDG) OR (CADASIL) OR (Caffey ADJ3 diseas*) OR (Calabro ADJ3 syndrom*) OR (Calloso-genital-dysplasia) OR (Camera-Marugo-Cohen ADJ3 syndrom*) OR (Campomelic-dysplasia) OR (Camptobrachydactyly) OR (Camptodactyly-arthropathy-coxa-vara-pericarditis ADJ3 syndrom*) OR (Camptodactyly ADJ3 syndrom*-Guadalajara-type-1) OR (Camptodactyly ADJ3 syndrom*-Guadalajara-type-2) OR (Camptodactyly ADJ3 syndrom*-Guadalajara-type-3) OR (Camptodactyly ADJ3 fibrous-tissue-hyperplasia ADJ3 -skeletal-dysplasia) OR (Camptodactyly ADJ3 tall-stature ADJ3 -hearing-loss ADJ3 syndrom*) OR (Camptodactyly-ichthyosis ADJ3 syndrom*) OR (Camptomelic ADJ3 syndrom*-long-limb) OR (Camurati-Engelmann ADJ3 diseas* ADJ3 type-2) OR (Camurati-Engelmann ADJ3 diseas*) OR (Canavan ADJ3 diseas*) OR (Cantu-Sanchez-Corona-Fragoso ADJ3 syndrom*) OR (Cantu ADJ3 syndrom*) OR (Cap-myopathy) OR (Carbamoyl-phosphate-synthetase-1 ADJ3 deficien*) OR (Carbonic-anhydrase-VA ADJ3 deficien*) OR (Cardiac-Valvular-Ehlers-Danlos ADJ3 syndrom*) OR (Cardioauditory ADJ3 syndrom* ADJ3 Sanchez-Cascos) OR (Cardioencephalomyopathy) OR (Cardiofaciocutaneous ADJ3 syndrom*) OR (Cardiomelic ADJ3 syndrom*-Stratton-Koehler) OR (Cardiomyopathy ADJ3 deafness-due-to-tRNA-lysine-gene-mutation) OR (Cardiomyopathy-cataract-hip-spine ADJ3 diseas*) OR (Cardiomyopathy-dilated ADJ3 woolly-hair ADJ3 keratoderma) OR (Cardioskeletal ADJ3 syndrom*-Kuwaiti) OR (Carey-Fineman-Ziter ADJ3 syndrom*) OR (Carney-complex) OR (Carney-triad) OR (Carnitine-palmitoyl-transferase-1A ADJ3 deficien*) OR (Carnitine-acylcarnitine-translocase ADJ3 deficien*) OR (Carnosinemia) OR (Caroli ADJ3 diseas*) OR (Carpenter ADJ3 syndrom*) OR (Carpotarsal-osteochondromatosis) OR (Cartilage-hair-hypoplasia) OR (Cataract-ataxia-deafness) OR (Cataract-congenital-Volkmann) OR (Cataract-microcornea ADJ3 syndrom*) OR (Cataract ADJ3 total-congenital) OR (Catatrichy) OR (Catecholaminergic-polymorphic-ventricular-tachycardia) OR (Catel-Manzke ADJ3 syndrom*) OR (Caudal-appendage-deafness) OR (Caudal-regression-sequence) OR (Central-core ADJ3 diseas*) OR (Cerebellar-ataxia ADJ3 hypogonadotropic-hypogonadism) OR (Cerebellar-ataxia-ectodermal-dysplasia) OR (Cerebellar-ataxia ADJ3 areflexia ADJ3 pes-cavus ADJ3 optic-atrophy ADJ3 sensorinural-hearing-loss) OR (Cerebellar-hypoplasia) OR (Cerebellar-hypoplasia-tapetoretinal-degeneration) OR (Cerebellar-hypoplasia ADJ3 endosteal-sclerosis) OR (Cerebelloparenchymal-disorder-3) OR (Cerebellum-agenesis-hydrocephaly) OR (Cerebral-autosomal-recessive-arteriopathy ADJ3 subcortical-infarcts ADJ3 leukoencephalopathy) OR (Cerebral-dysgenesis ADJ3 neuropathy ADJ3 ichthyosis ADJ3 -palmoplantar-keratoderma ADJ3 syndrom*) OR (Cerebral-folate ADJ3 deficien*) OR (Cerebral-gigantism-jaw-cysts) OR (Cerebro-costo-mandibular ADJ3 syndrom*) OR (Cerebro-facio-articular ADJ3 syndrom*) OR (Cerebro-oculo-facio-skeletal ADJ3 syndrom*) OR (Cerebrocostomandibular-like ADJ3 syndrom*) OR (Cerebrooculonasal ADJ3 syndrom*) OR (Cerebrotendinous-xanthomatosis) OR (Ceroid-lipofuscinosis-neuronal-1) OR (Cerulean-cataract) OR (Cervical-hypertrichosis-peripheral-neuropathy) OR (Chanarin-Dorfman ADJ3 syndrom*) OR (Char ADJ3 syndrom*) OR (Charcot-Marie-Tooth ADJ3 diseas*-type-1A) OR (Charcot-Marie-Tooth ADJ3 diseas*-type-1B) OR (Charcot-Marie-Tooth ADJ3 diseas*-type-1C) OR (Charcot-Marie-Tooth ADJ3 diseas*-type-1D) OR (Charcot-Marie-Tooth ADJ3 diseas*-type-1E) OR (Charcot-Marie-Tooth ADJ3 diseas*-type-1F) OR (Charcot-Marie-Tooth ADJ3 diseas*-type-2B) OR (Charcot-Marie-Tooth ADJ3 diseas*-type-2B1) OR (Charcot-Marie-Tooth ADJ3 diseas*-type-2B2) OR (Charcot-Marie-Tooth ADJ3 diseas*-type-2D) OR (Charcot-Marie-Tooth ADJ3 diseas*-type-2E) OR (Charcot-Marie-Tooth ADJ3 diseas*-type-2F) OR (Charcot-Marie-Tooth ADJ3 diseas*-type-2G) OR (Charcot-Marie-Tooth ADJ3 diseas*-type-2H) OR (Charcot-Marie-Tooth ADJ3 diseas*-type-2I) OR (Charcot-Marie-Tooth ADJ3 diseas*-type-2J) OR (Charcot-Marie-Tooth ADJ3 diseas*-type-2K) OR (Charcot-Marie-Tooth ADJ3 diseas*-type-2N) OR (Charcot-Marie-Tooth ADJ3 diseas*-type-2O) OR (Charcot-Marie-Tooth ADJ3 diseas*-type-2P) OR (Charcot-Marie-Tooth ADJ3 diseas*-type-2Q) OR (Charcot-Marie-Tooth ADJ3 diseas*-type-2R) OR (Charcot-Marie-Tooth ADJ3 diseas*-type-4A) OR (Charcot-Marie-Tooth ADJ3 diseas*-type-4B1) OR (Charcot-Marie-Tooth ADJ3 diseas*-type-4B2) OR (Charcot-Marie-Tooth ADJ3 diseas*-type-4C) OR (Charcot-Marie-Tooth ADJ3 diseas*-type-4D) OR (Charcot-Marie-Tooth ADJ3 diseas*-type-4E) OR (Charcot-Marie-Tooth ADJ3 diseas*-type-4H) OR (CHARGE ADJ3 syndrom*) OR (Charlie-M ADJ3 syndrom*) OR (Cherubism) OR (Chiari-malformation-type-2) OR (Chiari-malformation-type-3) OR (CHILD ADJ3 syndrom*) OR (Childhood-apraxia ADJ3 speech) OR (Childhood-encephalopathy-due-to-thiamine-pyrophosphokinase ADJ3 deficien*) OR (Childhood-hypophosphatasia) OR (Childhood-onset-nemaline-myopathy) OR (Chitayat-Meunier-Hodgkinson ADJ3 syndrom*) OR (Choanal-atresia-hearing-loss-cardiac ADJ3 defect*s-craniofacial-dysmorphism ADJ3 syndrom*) OR (Cholesteryl-ester-storage ADJ3 diseas*) OR (Chondrocalcinosis-2) OR (Chondrodysplasia-acromesomelic ADJ3 genital-anomalies) OR (Chondrodysplasia-Blomstrand) OR (Chondrodysplasia-calcificans-metaphysealis) OR (Chondrodysplasia-punctata-1 ADJ3 X-linked-recessive) OR (Chondrodysplasia-punctata-Sheffield) OR (Chondrodysplasia ADJ3 joint-dislocations ADJ3 GPAPP) OR (Chondrodysplasia ADJ3 Grebe) OR (Chordoma) OR (Chorea-acanthocytosis) OR (Choroidal-dystroph*-central-areolar) OR (Choroideremia) OR (Christianson ADJ3 syndrom*) OR (Chromosome-15 ADJ3 trisomy-mosaicism) OR (Chromosome-16p13#3-deletion ADJ3 syndrom*) OR (Chromosome-16p13#3 ADJ3 duplicat*) OR (Chromosome-17p13#1-deletion ADJ3 syndrom*) OR (Chromosome-17q-deletion) OR (Chromosome-18p ADJ3 duplicat*) OR (Chromosome-19q13#11-deletion ADJ3 syndrom*) OR (Chromosome-1q41-q42-deletion ADJ3 syndrom*) OR (Chromosome-21 ADJ3 uniparental-disomy) OR (Chronic-atypical-neutrophilic-dermatosis ADJ3 lipodystroph* ADJ3 elevated-temperature) OR (Chronic-granulomatous ADJ3 diseas*) OR (Chronic-progressive-external-ophthalmoplegia) OR (Chudley-Rozdilsky ADJ3 syndrom*) OR (Chylomicron-retention ADJ3 diseas*) OR (Chylothorax ADJ3 congenital) OR (Circumferential-skin-creases-Kunze) OR (Citrullinemia-type-II) OR (Clark-Baraitser ADJ3 syndrom*) OR (Clasped-thumbs ADJ3 congenital) OR (Classical-like-Ehlers-Danlos ADJ3 syndrom*) OR (Cleft-hand-absent-tibia) OR (Cleft-palate-short-stature-vertebral-anomalies) OR (Cleft-palate ADJ3 midfacial-hypoplasia ADJ3 triangular-facies ADJ3 -sensorineural-hearing-loss) OR (Cleidocranial-dysplasia) OR (Cleidocranial-dysplasia-recessive-form) OR (Cleidorhizomelic ADJ3 syndrom*) OR (Clouston ADJ3 syndrom*) OR (COACH ADJ3 syndrom*) OR (COASY-Protein-Associated-Neurodegeneration) OR (Coats ADJ3 diseas*) OR (Cockayne ADJ3 syndrom*) OR (Cockayne ADJ3 syndrom*-type-I) OR (Cockayne ADJ3 syndrom*-type-II) OR (Cockayne ADJ3 syndrom*-type-III) OR (CODAS ADJ3 syndrom*) OR (Coffin-Lowry ADJ3 syndrom*) OR (Coffin-Siris ADJ3 syndrom*) OR (COG1-CDG-) OR (CDG-IIg) OR (COG4-CDG-) OR (CDG-IIj) OR (COG5-CDG-) OR (CDG-IIi) OR (COG7-CDG-) OR (CDG-IIe) OR (COG8-CDG-) OR (CDG-IIh) OR (Cogan-Reese ADJ3 syndrom*) OR (Cohen ADJ3 syndrom*) OR (Cold-induced-sweating ADJ3 syndrom*) OR (Cole-Carpenter ADJ3 syndrom*) OR (Collins-Pope ADJ3 syndrom*) OR (Coloboma ADJ3 alar-nasal-cartilages ADJ3 telecanthus) OR (Coloboma ADJ3 macula) OR (Coloboma ADJ3 macula ADJ3 type-B-brachydactyly) OR (Colpocephaly) OR (Combined-immunodeficiency ADJ3 skin-granulomas) OR (Combined-oxidative-phosphorylation ADJ3 deficien*-16) OR (Combined-pituitary-hormone-deficiencies ADJ3 genetic-forms) OR (Common-variable-immunodeficiency) OR (Complement-component-2 ADJ3 deficien*) OR (Complete-androgen-insensitivity ADJ3 syndrom*) OR (Condensing-osteitis ADJ3 clavicle) OR (Conductive-deafness ADJ3 malformed-external-ear) OR (Cone-dystroph*) OR (Cone-rod-dystroph*) OR (Cone-rod-dystroph*-3) OR (Cone-rod-dystroph*-5) OR (Cone-rod-dystroph*-6) OR (Cone-rod-dystroph*-amelogenesis-imperfecta) OR (Cone-rod-dystroph*-X-linked-1) OR (Cone-rod-dystroph*-X-linked-2) OR (Cone-rod-dystroph*-X-linked-3) OR (Congenital-absence ADJ3 sternocleidomastoid-muscle) OR (Congenital-adrenal-hyperplasia-due-to-cytochrome-P450-oxidoreductase ADJ3 deficien*) OR (Congenital-anosmia) OR (Congenital-bilateral-absence ADJ3 vas-deferens) OR (Congenital-bile-acid-synthesis ADJ3 defect* ADJ3 type-1) OR (Congenital-bile-acid-synthesis ADJ3 defect* ADJ3 type-2) OR (Congenital-central-hypoventilation ADJ3 syndrom*) OR (Congenital-chloride-diarrhea) OR (Congenital-contractural-arachnodactyly) OR (Congenital-cytomegalovirus) OR (Congenital-deafness ADJ3 vitiligo ADJ3 achalasia) OR (Congenital-diaphragmatic-hernia) OR (Congenital-ectodermal-dysplasia ADJ3 hearing-loss) OR (Congenital-extrahepatic-portosystemic-shunt) OR (Congenital-femoral ADJ3 deficien*) OR (Congenital-fiber-type-disproportion) OR (Congenital-fibrosis ADJ3 extraocular-muscles) OR (Congenital-generalized-lipodystroph*) OR (Congenital-generalized-lipodystroph*-type-2) OR (Congenital-generalized-lipodystroph*-type-4) OR (Congenital-heart-block) OR (Congenital-hydrocephalus) OR (Congenital-hyperinsulinism) OR (Congenital-insensitivity-to-pain) OR (Congenital-insensitivity-to-pain ADJ3 anhidrosis) OR (Congenital-lactase ADJ3 deficien*) OR (Congenital-laryngeal-palsy) OR (Congenital-lipoid-adrenal-hyperplasia) OR (Congenital-lobar-emphysema) OR (Congenital-microcoria) OR (Congenital-mirror-movement-disorder) OR (Congenital-muscular-dystroph*-due-to-LMNA-mutation) OR (Congenital-muscular-dystroph*-type-1A) OR (Congenital-muscular-dystroph* ADJ3 integrin-alpha-7 ADJ3 deficien*) OR (Congenital-muscular-dystroph*-dystroglycanopathy ADJ3 intellectual-disability-) OR (Congenital-myasthenic ADJ3 syndrom*-associated ADJ3 acetylcholine-receptor ADJ3 deficien*) OR (Congenital-nephrotic ADJ3 syndrom*-Finnish) OR (Congenital-primary-aphakia) OR (Congenital-pseudoarthrosis) OR (Congenital-pulmonary-alveolar-proteinosis) OR (Congenital-rubella) OR (Congenital-sucrase-isomaltase ADJ3 deficien*) OR (Congenital-tracheal-stenosis) OR (Congenital-tracheomalacia) OR (Congenital-varicella ADJ3 syndrom*) OR (Congenital-vertical-talus) OR (Congenitally-corrected-transposition ADJ3 great-arteries) OR (Continuous-spike-wave-during-slow-sleep ADJ3 syndrom*) OR (Convulsions ADJ3 benign-familial-infantile ADJ3 1) OR (Copper ADJ3 deficien* ADJ3 familial-benign) OR (Cor-triatriatum-dexter) OR (Cor-triatriatum-sinister) OR (Corneal-dystroph* ADJ3 perceptive-deafness) OR (Corneal-dystroph*-Avellino) OR (Corneal-dystroph*-crystalline ADJ3 Schnyder) OR (Corneal-dystroph*-Thiel-Behnke) OR (Corneal-endothelial-dystroph*-type-2) OR (Corneal-hypesthesia ADJ3 familial) OR (Cornelia-de-Lange ADJ3 syndrom*) OR (Corneodermatoosseous ADJ3 syndrom*) OR (Corpus-callosum-agenesis-double-urinary-collecting) OR (Cortical-blindness-intellectual-disability-polydactyly ADJ3 syndrom*) OR (Cortical ADJ3 defect*s-wormian-bones ADJ3 dentinogenesis-imperfecta) OR (Cortical-dysgenesis ADJ3 pontocerebellar-hypoplasia-due-to-TUBB3-mutation) OR (Corticobasal-degeneration) OR (Corticosteroid-binding-globulin ADJ3 deficien*) OR (Costello ADJ3 syndrom*) OR (Cousin ADJ3 syndrom*) OR (Cowden ADJ3 syndrom*) OR (Crandall ADJ3 syndrom*) OR (Crane-Heise ADJ3 syndrom*) OR (Craniodiaphyseal-dysplasia) OR (Cranioectodermal-dysplasia) OR (Craniofacial-deafness-hand ADJ3 syndrom*) OR (Craniofacial-dysostosis ADJ3 diaphyseal-hyperplasia) OR (Craniofacial-dyssynostosis) OR (Craniofrontonasal-dysplasia) OR (Craniometaphyseal-dysplasia ADJ3 autosomal-dominant) OR (Craniometaphyseal-dysplasia ADJ3 autosomal-recessive) OR (Craniopharyngioma) OR (Craniorachischisis) OR (Craniosynostosis) OR (Craniosynostosis ADJ3 anal-anomalies ADJ3 -porokeratosis) OR (Craniotelencephalic-dysplasia) OR (Cri-du-chat ADJ3 syndrom*) OR (Crigler-Najjar ADJ3 syndrom* ADJ3 type-1) OR (Crigler-Najjar ADJ3 syndrom*-type-2) OR (Crome ADJ3 syndrom*) OR (Cronkhite-Canada ADJ3 diseas*) OR (Crouzon ADJ3 syndrom*) OR (Crumpled-helices ADJ3 small-mouth) OR (Cryptomicrotia-brachydactyly ADJ3 syndrom*) OR (Cryptophthalmos) OR (Culler-Jones ADJ3 syndrom*) OR (Curly-hair-acral-keratoderma-caries ADJ3 syndrom*) OR (Currarino-triad) OR (Curry-Jones ADJ3 syndrom*) OR (Cutis-laxa ADJ3 autosomal-dominant) OR (Cylindrical-spirals-myopathy) OR (Cyprus-facial-neuromusculoskeletal ADJ3 syndrom*) OR (Cystic-fibrosis) OR (Cystic-hygroma) OR (Cystinosis) OR (Czech-dysplasia-metatarsal) OR (D-ercole ADJ3 syndrom*) OR (D-2-hydroxyglutaric-aciduria) OR (D-bifunctional-protein ADJ3 deficien*) OR (D-glycericacidemia) OR (Daentl-Towsend-Siegel ADJ3 syndrom*) OR (Daish-Hardman-Lamont ADJ3 syndrom*) OR (Dandy-Walker-complex) OR (Dandy-Walker-cyst ADJ3 Renal-Hepatic-Pancreatic-dysplasia) OR (Dandy-Walker-like-malformation ADJ3 atrioventricular-septal ADJ3 defect*) OR (Dandy-Walker-malformation ADJ3 intellectual-disability ADJ3 basal-ganglia ADJ3 diseas* ADJ3 seizures) OR (Dandy-Walker-malformation ADJ3 nasopharyngeal-teratoma ADJ3 diaphragmatic-hernia) OR (Dandy-Walker-malformation ADJ3 postaxial-polydactyly) OR (Dandy-Walker-malformation ADJ3 sagittal-craniosynostosis ADJ3 hydrocephalus) OR (Daneman-Davy-Mancer ADJ3 syndrom*) OR (Danon ADJ3 diseas*) OR (Darier ADJ3 diseas*) OR (Dauwerse-Peters ADJ3 syndrom*) OR (Davenport-Donlan ADJ3 syndrom*) OR (DCMA ADJ3 syndrom*) OR (DDOST-CDG-) OR (CDG-Ir) OR (De-Barsy ADJ3 syndrom*) OR (De-Sanctis-Cacchione ADJ3 syndrom*) OR (DEAF1-associated-disorders) OR (Deafness ADJ3 myopia ADJ3 syndrom*) OR (Deafness-conductive-ptosis-skeletal-anomalies) OR (Deafness-enamel-hypoplasia-nail ADJ3 defect*s) OR (Deafness-hypogonadism ADJ3 syndrom*) OR (Deafness-oligodontia ADJ3 syndrom*) OR (Deafness ADJ3 labyrinthine-aplasia-microtia ADJ3 microdontia-) OR (LAMM) OR (Deafness ADJ3 autosomal-dominant-nonsyndromic-sensorineural-17) OR (Deafness ADJ3 autosomal-dominant-nonsyndromic-sensorineural-22) OR (Deafness ADJ3 autosomal-dominant-nonsyndromic-sensorineural-23) OR (Deafness ADJ3 autosomal-dominant-nonsyndromic-sensorineural-24) OR (Deafness ADJ3 autosomal-dominant-nonsyndromic-sensorineural-3) OR (Deafness ADJ3 autosomal-dominant-nonsyndromic-sensorineural-53) OR (Deafness ADJ3 autosomal-recessive-51) OR (Deafness ADJ3 dystonia ADJ3 -cerebral-hypomyelination) OR (Deafness ADJ3 epiphyseal-dysplasia ADJ3 short-stature) OR (Deafness ADJ3 X-linked-2) OR (Deafness-infertility ADJ3 syndrom*) OR (Deficiency ADJ3 interleukin-1-receptor-antagonist) OR (Delayed-membranous-cranial-ossification) OR (Dendritic-cell ADJ3 monocyte ADJ3 B-lymphocyte ADJ3 -natural-killer-lymphocyte ADJ3 deficien*) OR (Dense-deposit ADJ3 diseas*) OR (Dentatorubral-pallidoluysian-atrophy) OR (Dentin-dysplasia-sclerotic-bones) OR (Dentin-dysplasia ADJ3 coronal) OR (Dentin-dysplasia ADJ3 type-1) OR (Dentinogenesis-imperfecta) OR (Dentinogenesis-imperfecta-type-2) OR (Dentinogenesis-imperfecta-type-3) OR (Denys-Drash ADJ3 syndrom*) OR (Dermatofibrosarcoma-protuberans) OR (Dermatoosteolysis-Kirghizian) OR (Dermatopathia-pigmentosa-reticularis) OR (Dermatosparaxis-Ehlers-Danlos ADJ3 syndrom*) OR (Dermochondrocorneal-dystroph* ADJ3 Francois) OR (Dermoodontodysplasia) OR (Desbuquois ADJ3 syndrom*) OR (Desmoid-tumor) OR (Desmosterolosis) OR (Devriendt ADJ3 syndrom*) OR (Dextrocardia) OR (Dextrocardia ADJ3 unusual-facies ADJ3 microphthalmia) OR (DFNB1) OR (Diaphyseal-medullary-stenosis ADJ3 malignant-fibrous-histiocytoma) OR (Diastrophic-dysplasia) OR (Dicarboxylic-aminoaciduria) OR (Dihydrolipoamide-dehydrogenase ADJ3 deficien*) OR (Dihydropteridine-reductase ADJ3 deficien*) OR (Dihydropyrimidinase ADJ3 deficien*) OR (Dilated-cardiomyopathy) OR (Dilated-cardiomyopathy ADJ3 hypergonadotropic-hypogonadism) OR (Diphallia) OR (Disseminated-superficial-actinic-porokeratosis) OR (Distal-arthrogryposis-type-1) OR (Distal-arthrogryposis-type-5) OR (Distal-arthrogryposis-type-5D) OR (Distal-arthrogryposis ADJ3 hypopituitarism ADJ3 intellectual-disability ADJ3 facial-anomalies) OR (Distal-myopathy ADJ3 vocal-cord-weakness) OR (DK-phocomelia ADJ3 syndrom*) OR (DOLK-CDG-) OR (CDG-Im) OR (Dominant-dystrophic-epidermolysis-bullosa) OR (Donnai-Barrow ADJ3 syndrom*) OR (DOOR ADJ3 syndrom*) OR (Dopa-responsive-dystonia) OR (Dopamine-beta-hydroxylase ADJ3 deficien*) OR (Dopamine-transporter ADJ3 deficien* ADJ3 syndrom*) OR (Dowling-Degos ADJ3 diseas*) OR (DPAGT1-CDG-) OR (CDG-Ij) OR (DPM1-CDG-) OR (CDG-Ie) OR (DPM2-CDG) OR (DPM3-CDG-) OR (CDG-Io) OR (Drachtman-Weinblatt-Sitarz ADJ3 syndrom*) OR (Dravet ADJ3 syndrom*) OR (Duane ADJ3 syndrom*) OR (Duane-radial-ray ADJ3 syndrom*) OR (Dubin-Johnson ADJ3 syndrom*) OR (Dubowitz ADJ3 syndrom*) OR (Duchenne-muscular-dystroph*) OR (Duodenal-atresia) OR (Duplication ADJ3 urethra) OR (Dwarfism-familial ADJ3 muscle-spasms) OR (Dwarfism-Levi) OR (Dwarfism ADJ3 low-birth-weight-type ADJ3 unresponsiveness-to-growth-hormone) OR (Dwarfism ADJ3 proportionate ADJ3 hip-dislocation) OR (Dyggve-Melchior-Clausen ADJ3 syndrom*) OR (Dykes-Markes-Harper ADJ3 syndrom*) OR (Dyschondrosteosis-nephritis) OR (Dyschromatosis-symmetrica-hereditaria-1) OR (Dyschromatosis-universalis-hereditaria) OR (Dysequilibrium ADJ3 syndrom*) OR (Dysosteosclerosis) OR (Dysplasia-epiphysealis-hemimelica) OR (Dyssegmental-dysplasia ADJ3 glaucoma) OR (Dyssegmental-dysplasia-Rolland-Desbuquois) OR (Dyssegmental-dysplasia-Silverman-Handmaker) OR (Dystelephalangy) OR (Dystonia-2 ADJ3 torsion ADJ3 autosomal-recessive) OR (DYT-PRKRA) OR (DYT-THAP1) OR (DYT-TOR1A) OR (DYT-TUBB4A) OR (Early-Infantile-Epileptic-Encephalopathy) OR (Early-infantile-epileptic-encephalopathy-25) OR (Early-onset-anterior-polar-cataract) OR (Early-onset-autosomal-dominant-Alzheimer ADJ3 diseas*) OR (Early-onset-parkinsonism-intellectual-disability ADJ3 syndrom*) OR (Early-onset-zonular-cataract) OR (Ebstein ADJ3 -anomaly) OR (Ectodermal-dysplasia) OR (Ectodermal-dysplasia-skin-fragility ADJ3 syndrom*) OR (Ectodermal-dysplasia-trichoodontoonychial) OR (Ectodermal-dysplasia ADJ3 natal-teeth-Turnpenny) OR (Ectodermal-dysplasia ADJ3 hidrotic ADJ3 Christianson-Fourie) OR (Ectodermal-dysplasia ADJ3 sensorineural-hearing-loss ADJ3 -distinctive-facial-features) OR (EEC ADJ3 syndrom*) OR (EEM ADJ3 syndrom*) OR (Eisenmenger ADJ3 syndrom*) OR (Elastosis-perforans-serpiginosa) OR (Ellis-Yale-Winter ADJ3 syndrom*) OR (Ellis-Van-Creveld ADJ3 syndrom*) OR (Encephalocele) OR (Encephalocraniocutaneous-lipomatosis) OR (Encephalopathy-due-to-prosaposin ADJ3 deficien*) OR (Encephalopathy-intracranial-calcification-growth-hormone ADJ3 deficien*-microcephaly-retinal-degeneration) OR (Epidermodysplasia-verruciformis) OR (Epidermolysa-bullosa-simplex ADJ3 muscular-dystroph*) OR (Epidermolysis-bullosa-simplex ADJ3 mottled-pigmentation) OR (Epidermolysis-bullosa-simplex ADJ3 Dowling-Meara) OR (Epidermolysis-bullosa-simplex ADJ3 localized) OR (Epidermolysis-bullosa-simplex ADJ3 Ogna) OR (Epidermolysis-bullosa ADJ3 lethal-acantholytic) OR (Epidermolytic-ichthyosis) OR (Epidermolytic-palmoplantar-keratoderma) OR (Epilepsy-juvenile-absence) OR (Epilepsy ADJ3 myoclonic-atonic-seizures) OR (Epiphyseal-dysplasia-hearing-loss-dysmorphism) OR (Epiphyseal-dysplasia-multiple ADJ3 early-onset-diabetes-mellitus) OR (Episodic-ataxia ADJ3 nystagmus) OR (Ermine-phenotype) OR (Erythrokeratoderma-en-cocardes) OR (Erythromelalgia) OR (Erythropoietic-uroporphyria-associated ADJ3 myeloid-malignancy) OR (Esophageal-atresia) OR (Ethylmalonic-encephalopathy) OR (Eunuchoidism-familial-hypogonadotropic) OR (Exstrophy ADJ3 bladder) OR (FACES ADJ3 syndrom*) OR (Facial-ectodermal-dysplasia) OR (Facial-onset-sensory ADJ3 motor-neuronopathy) OR (Facio-thoraco-genital ADJ3 syndrom*) OR (Faciocardiorenal ADJ3 syndrom*) OR (Facioscapulohumeral-muscular-dystroph*) OR (Fallot-complex ADJ3 severe-mental ADJ3 growth-retardation) OR (Familial-amyloidosis ADJ3 Finnish) OR (Familial-atrial-fibrillation) OR (Familial-avascular-necrosis ADJ3 femoral-head) OR (Familial-bilateral-striatal-necrosis) OR (Familial-caudal-dysgenesis) OR (Familial-cold-autoinflammatory ADJ3 syndrom*) OR (Familial-congenital-palsy ADJ3 trochlear-nerve) OR (Familial-cutaneous-collagenoma) OR (Familial-dilated-cardiomyopathy) OR (Familial-dysautonomia) OR (Familial-encephalopathy ADJ3 neuroserpin-inclusion-bodies) OR (Familial-exudative-vitreoretinopathy) OR (Familial-focal-epilepsy ADJ3 variable-foci) OR (Familial-glucocorticoid ADJ3 deficien*) OR (Familial-HDL ADJ3 deficien*) OR (Familial-hemiplegic-migraine) OR (Familial-hemophagocytic-lymphohistiocytosis) OR (Familial-hyperaldosteronism-type-2) OR (Familial-hyperaldosteronism-type-III) OR (Familial-hypocalciuric-hypercalcemia-type-1) OR (Familial-hypocalciuric-hypercalcemia-type-2) OR (Familial-hypocalciuric-hypercalcemia-type-3) OR (Familial-infantile-convulsions ADJ3 paroxysmal-choreoathetosis) OR (Familial-joint-instability ADJ3 syndrom*) OR (Familial-lipoprotein-lipase ADJ3 deficien*) OR (Familial-Mediterranean-fever) OR (Familial-multiple-lipomatosis) OR (Familial-osteochondritis-dissecans) OR (Familial-partial-lipodystroph*-associated ADJ3 PLIN1-mutations) OR (Familial-partial-lipodystroph*-associated ADJ3 PPARG-mutations) OR (Familial-partial-lipodystroph*-due-to-AKT2-mutations) OR (Familial-partial-lipodystroph*-type-2) OR (Familial-partial-lipodystroph*-type-Kobberling) OR (Familial-porencephaly) OR (Familial-progressive-cardiac-conduction ADJ3 defect*) OR (Familial-reactive-perforating-collagenosis) OR (Familial-thyroglossal-duct-cyst) OR (Familial-visceral-myopathy ADJ3 external-ophthalmoplegia) OR (Familiar-hemiplegic-migraine) OR (Sporadic-hemiplegic-migraine) OR (Fanconi-Bickel ADJ3 syndrom*) OR (Fanconi ADJ3 syndrom*) OR (Farber ADJ3 diseas*) OR (Fatal-familial-insomnia) OR (Fatty-acid-hydroxylase-associated-neurodegeneration) OR (Faye-Petersen-Ward-Carey ADJ3 syndrom*) OR (FBXL4-related-encephalomyopathic-mitochondrial-DNA-depletion ADJ3 syndrom*) OR (Feigenbaum-Bergeron-Richardson ADJ3 syndrom*) OR (Feingold ADJ3 syndrom*) OR (Femoral-facial ADJ3 syndrom*) OR (Femur-bifid ADJ3 monodactylous-ectrodactyly) OR (Femur-fibula-ulna ADJ3 syndrom*) OR (Fertile-eunuch ADJ3 syndrom*) OR (Fetal-akinesia-deformation-sequence) OR (Fetal-aminopterin ADJ3 syndrom*) OR (Fetal-cystic-hygroma) OR (Fetal-hydantoin ADJ3 syndrom*) OR (Fetal-methylmercury ADJ3 syndrom*) OR (Fetal-retinoid ADJ3 syndrom*) OR (Fetal-thalidomide ADJ3 syndrom*) OR (Fetal-valproate ADJ3 syndrom*) OR (FG ADJ3 syndrom*) OR (FG ADJ3 syndrom*-2) OR (FG ADJ3 syndrom*-3) OR (Fibro-adipose-vascular-anomaly) OR (Fibrochondrogenesis) OR (Fibrodysplasia-ossificans-progressiva) OR (Fibrous-dysplasia) OR (Fibular-aplasia-ectrodactyly) OR (Fibular-aplasia ADJ3 tibial-campomelia ADJ3 -oligosyndactyly ADJ3 syndrom*) OR (Fibular-hemimelia) OR (Fibular-hypoplasia ADJ3 complex-brachydactyly) OR (Filippi ADJ3 syndrom*) OR (Fine-Lubinsky ADJ3 syndrom*) OR (Fingerprint-body-myopathy) OR (Fish-eye ADJ3 diseas*) OR (Fitzsimmons ADJ3 syndrom*) OR (Fitzsimmons-Walson-Mellor ADJ3 syndrom*) OR (Fitzsimmons-Guilbert ADJ3 syndrom*) OR (Floating-Harbor ADJ3 syndrom*) OR (Flynn-Aird ADJ3 syndrom*) OR (Focal-cortical-dysplasia ADJ3 Taylor) OR (Focal-dermal-hypoplasia) OR (Focal-facial-dermal-dysplasia) OR (Focal-segmental-glomerulosclerosis) OR (Follicle-stimulating-hormone ADJ3 deficien* ADJ3 isolated) OR (Fountain ADJ3 syndrom*) OR (FOXG1 ADJ3 syndrom*) OR (Fragile-X ADJ3 syndrom*) OR (Fragile-XE ADJ3 syndrom*) OR (Frank-Ter-Haar ADJ3 syndrom*) OR (Fraser ADJ3 syndrom*) OR (Frasier ADJ3 syndrom*) OR (Free-sialic-acid-storage ADJ3 diseas*) OR (Freeman-Sheldon ADJ3 syndrom*) OR (Frias ADJ3 syndrom*) OR (Friedreich-ataxia) OR (Frints-De-Smet-Fabry-Fryns ADJ3 syndrom*) OR (Frontofacionasal-dysplasia) OR (Frontometaphyseal-dysplasia) OR (Frontonasal-dysplasia) OR (Frontonasal-dysplasia ADJ3 alopecia ADJ3 genital-anomaly) OR (Frontonasal-dysplasia-severe-microphthalmia-severe-facial-clefting ADJ3 syndrom*) OR (Frontorhiny) OR (Frontotemporal-dementia ADJ3 ubiquitin-positive) OR (Froster-Huch ADJ3 syndrom*) OR (Fructose-1-6-bisphosphatase ADJ3 deficien*) OR (Fryns-Hofkens-Fabry ADJ3 syndrom*) OR (Fryns ADJ3 syndrom*) OR (Fucosidosis) OR (Fuhrmann ADJ3 syndrom*) OR (Fukuyama-type-muscular-dystroph*) OR (Fumarase ADJ3 deficien*) OR (Fused-mandibular-incisors) OR (Galactokinase ADJ3 deficien*) OR (Galactosemia) OR (Galactosialidosis) OR (Galloway-Mowat ADJ3 syndrom*) OR (Game-Friedman-Paradice ADJ3 syndrom*) OR (Gamma-aminobutyric-acid-transaminase ADJ3 deficien*) OR (Gamma-cystathionase ADJ3 deficien*) OR (GAPO ADJ3 syndrom*) OR (Gardner ADJ3 syndrom*) OR (Garret-Tripp ADJ3 syndrom*) OR (Gastrocutaneous ADJ3 syndrom*) OR (Gastrointestinal-Stromal-Tumors) OR (Gastroschisis) OR (GATAD2B-associated-neurodevelopmental-disorder) OR (Gaucher ADJ3 diseas*---ophthalmoplegia---cardiovascular-calcification) OR (Gaucher ADJ3 diseas*-perinatal-lethal) OR (Gaucher ADJ3 diseas*-type-1) OR (Gaucher ADJ3 diseas*-type-2) OR (Gaucher ADJ3 diseas*-type-3) OR (Gay-Feinmesser-Cohen ADJ3 syndrom*) OR (Geleophysic-dwarfism) OR (Gemignani ADJ3 syndrom*) OR (Generalized-junctional-epidermolysis-bullosa ADJ3 non-Herlitz) OR (Generalized-pustular-psoriasis) OR (Genito-palato-cardiac ADJ3 syndrom*) OR (Genitopatellar ADJ3 syndrom*) OR (Genoa ADJ3 syndrom*) OR (Genochondromatosis) OR (Genu-valgum ADJ3 st-Helena-familial) OR (Geroderma-osteodysplastica) OR (Gerstmann-Straussler-Scheinker ADJ3 diseas*) OR (Gestational-trophoblastic-tumor) OR (Ghosal-hematodiaphyseal-dysplasia ADJ3 syndrom*) OR (Ghose-Sachdev-Kumar ADJ3 syndrom*) OR (Giant-axonal-neuropathy) OR (Giant-congenital-nevus) OR (Gillespie ADJ3 syndrom*) OR (Gingival-fibromatosis ADJ3 distinctive-facies) OR (Gingival-fibromatosis ADJ3 hypertrichosis) OR (Gitelman ADJ3 syndrom*) OR (Glaucoma-sleep-apnea) OR (Glaucoma ADJ3 Ectopia ADJ3 Microspherophakia ADJ3 Stiff-joints ADJ3 Short-stature ADJ3 syndrom*) OR (Globozoospermia) OR (Glomerulonephritis ADJ3 sparse-hair ADJ3 telangiectases) OR (Glomerulopathy ADJ3 fibronectin-deposits-1) OR (Glomerulopathy ADJ3 fibronectin-deposits-2) OR (Glucose-transporter-type-1 ADJ3 deficien* ADJ3 syndrom*) OR (Glucose-galactose-malabsorption) OR (Glutamine ADJ3 deficien* ADJ3 congenital) OR (Glutaric-acidemia-type-I) OR (Glutaric-acidemia-type-II) OR (Glutaric-acidemia-type-III) OR (Glutathionuria) OR (Glycine-N-methyltransferase ADJ3 deficien*) OR (Glycogen-storage ADJ3 diseas*-type-0 ADJ3 liver) OR (Glycogen-storage ADJ3 diseas*-type-13) OR (Glycogen-storage ADJ3 diseas*-type-1A) OR (Glycogen-storage ADJ3 diseas*-type-1B) OR (Glycogen-storage ADJ3 diseas*-type-3) OR (Glycogen-storage ADJ3 diseas*-type-4) OR (Glycogen-storage ADJ3 diseas*-type-5) OR (Glycogen-storage ADJ3 diseas*-type-6) OR (GM1-gangliosidosis-type-1) OR (GM1-gangliosidosis-type-2) OR (GM1-gangliosidosis-type-3) OR (GM3-synthase ADJ3 deficien*) OR (GMS ADJ3 syndrom*) OR (Gnathodiaphyseal-dysplasia) OR (Goldberg-Shprintzen-megacolon ADJ3 syndrom*) OR (Goldenhar ADJ3 diseas*) OR (Goldmann-Favre ADJ3 syndrom*) OR (Gomez-Lopez-Hernandez ADJ3 syndrom*) OR (Gordon ADJ3 syndrom*) OR (Gorlin-Chaudhry-Moss ADJ3 syndrom*) OR (GOSR2-related-progressive-myoclonus-ataxia) OR (Gracile-bone-dysplasia) OR (GRACILE ADJ3 syndrom*) OR (Graham-Boyle-Troxell ADJ3 syndrom*) OR (Graham-Cox ADJ3 syndrom*) OR (Graham-Little-Piccardi-Lassueur ADJ3 syndrom*) OR (Grant ADJ3 syndrom*) OR (Greenberg-dysplasia) OR (Greig-cephalopolysyndactyly ADJ3 syndrom*) OR (Griscelli ADJ3 syndrom*-type-1) OR (Griscelli ADJ3 syndrom*-type-2) OR (Griscelli ADJ3 syndrom*-type-3) OR (Groll-Hirschowitz ADJ3 syndrom*) OR (Growth-hormone-insensitivity ADJ3 immunodeficiency) OR (Grubben-de-Cock-Borghgraef ADJ3 syndrom*) OR (GTP-cyclohydrolase-I ADJ3 deficien*) OR (GTPCH1-deficient-DRD) OR (Guanidinoacetate-methyltransferase ADJ3 deficien*) OR (Guizar-Vasquez-Sanchez-Manzano ADJ3 syndrom*) OR (Gurrieri ADJ3 syndrom*) OR (Gyrate-atrophy ADJ3 choroid ADJ3 retina) OR (Hailey-Hailey ADJ3 diseas*) OR (Haim-Munk ADJ3 syndrom*) OR (Hair ADJ3 defect*-photosensitivity-intellectual-disability ADJ3 syndrom*) OR (Hairy-elbows) OR (Halal ADJ3 syndrom*) OR (Hall-Riggs ADJ3 syndrom*) OR (Hallermann-Streiff ADJ3 syndrom*) OR (Hamanishi-Ueba-Tsuji ADJ3 syndrom*) OR (Hand ADJ3 foot-deformity ADJ3 flat-facies) OR (Hand-foot-uterus ADJ3 syndrom*) OR (Hanhart ADJ3 syndrom*) OR (Hard-skin ADJ3 syndrom*-Parana) OR (Hardikar ADJ3 syndrom*) OR (Harding-ataxia) OR (Harlequin-ichthyosis) OR (Harlequin ADJ3 syndrom*) OR (Harrod-Doman-Keele ADJ3 syndrom*) OR (Hartnup ADJ3 diseas*) OR (Hawkinsinuria) OR (Heart ADJ3 defect*-tongue-hamartoma-polysyndactyly ADJ3 syndrom*) OR (Heart-hand ADJ3 syndrom* ADJ3 Slovenian) OR (Heart-hand ADJ3 syndrom* ADJ3 Spanish) OR (HEC ADJ3 syndrom*) OR (Hemangiomatosis ADJ3 familial-pulmonary-capillary) OR (Hemi-3 ADJ3 syndrom*) OR (Hemifacial-hyperplasia-strabismus) OR (Hemifacial-microsomia) OR (Hemimegalencephaly) OR (Hepatic-lipase ADJ3 deficien*) OR (Hepatic-venoocclusive ADJ3 diseas* ADJ3 immunodeficiency) OR (Hepatoerythropoietic-porphyria) OR (Hereditary-angiopathy ADJ3 nephropathy ADJ3 aneurysms ADJ3 -muscle-cramps ADJ3 syndrom*) OR (Hereditary-congenital-facial-paresis) OR (Hereditary-coproporphyria) OR (Hereditary-diffuse-leukoencephalopathy ADJ3 spheroids) OR (Hereditary-fibrosing-poikiloderma ADJ3 tendon-contractures ADJ3 myopathy ADJ3 -pulmonary-fibrosis) OR (Hereditary-fructose-intolerance) OR (Hereditary-geniospasm) OR (Hereditary-hyperekplexia) OR (Hereditary-keratitis) OR (Hereditary-koilonychia) OR (Hereditary-leiomyomatosis ADJ3 renal-cell-cancer) OR (Hereditary-motor ADJ3 sensory-neuropathy-type-5) OR (Hereditary-mucoepithelial-dysplasia) OR (Hereditary-multiple-osteochondromas) OR (Hereditary-neuralgic-amyotrophy) OR (Hereditary-neuropathy ADJ3 liability-to-pressure-palsies) OR (Hereditary-pancreatitis) OR (Hereditary-proximal-myopathy ADJ3 early-respiratory-failure) OR (Hereditary-sensorimotor-neuropathy ADJ3 hyperelastic-skin) OR (Hereditary-sensory ADJ3 autonomic-neuropathy-type-1E) OR (Hereditary-sensory ADJ3 autonomic-neuropathy-type-2) OR (Hereditary-sensory ADJ3 autonomic-neuropathy-type-7) OR (Hereditary-sensory ADJ3 autonomic-neuropathy-type-V) OR (Hereditary-sensory-neuropathy-type-1) OR (Hereditary-vascular-retinopathy) OR (Hermansky-Pudlak ADJ3 syndrom*) OR (Hernandez-Aguirre-Negrete ADJ3 syndrom*) OR (Herpes-simplex-encephalitis) OR (Heterotaxy) OR (HIBCH ADJ3 deficien*) OR (Hirschsprung ADJ3 diseas*) OR (Hirschsprung ADJ3 diseas*-type-d-brachydactyly) OR (His-bundle-tachycardia) OR (Histidinemia) OR (HMG-CoA-lyase ADJ3 deficien*) OR (Holocarboxylase-synthetase ADJ3 deficien*) OR (Holt-Oram ADJ3 syndrom*) OR (Holzgreve ADJ3 syndrom*) OR (Homocarnosinosis) OR (Homocystinuria-due-to-CBS ADJ3 deficien*) OR (Homocystinuria-due-to-MTHFR ADJ3 deficien*) OR (Horizontal-gaze-palsy ADJ3 progressive-scoliosis) OR (HSD10 ADJ3 diseas*) OR (Human-HOXA1 ADJ3 syndrom*s) OR (Hunter-McAlpine ADJ3 syndrom*) OR (Huntington ADJ3 diseas*) OR (Hurler ADJ3 syndrom*) OR (Hurler-Scheie ADJ3 syndrom*) OR (Hutterite-cerebroosteonephrodysplasia ADJ3 syndrom*) OR (Hyaline-fibromatosis ADJ3 syndrom*) OR (Hydranencephaly) OR (Hydrocephalus-due-to-congenital-stenosis ADJ3 aqueduct ADJ3 sylvius) OR (Hydrocephalus-obesity-hypogonadism) OR (Hydrocephalus ADJ3 costovertebral-dysplasia ADJ3 Sprengel-anomaly) OR (Hydrocephalus-cleft-palate-joint-contractures ADJ3 syndrom*) OR (Hydroxykynureninuria) OR (Hyper-IgD ADJ3 syndrom*) OR (Hyperbetaalaninemia) OR (Hyperbilirubinemia-transient-familial-neonatal) OR (Hyperferritinemia-cataract ADJ3 syndrom*) OR (Hyperinsulinism-due-to-glucokinase ADJ3 deficien*) OR (Hyperinsulinism-hyperammonemia ADJ3 syndrom*) OR (Hyperkalemic-periodic-paralysis) OR (Hyperkeratosis-lenticularis-perstans) OR (Hyperlipidemia-type-3) OR (Hyperlipoproteinemia-type-5) OR (Hyperlysinemia) OR (Hypermethioninemia-due-to-S-adenosylhomocysteine-hydrolase ADJ3 deficien*) OR (Hypermobile-Ehlers-Danlos ADJ3 syndrom*) OR (Hyperostosis-corticalis-generalisata) OR (Hyperparathyroidism-jaw-tumor ADJ3 syndrom*) OR (Hyperphenylalaninemia-due-to-dehydratase ADJ3 deficien*) OR (Hyperprolinemia) OR (Hyperprolinemia-type-2) OR (Hypertelorism ADJ3 tetralogy ADJ3 Fallot) OR (Hyperthermia-induced ADJ3 defect*s) OR (Hypertrichosis-lanuginosa-congenita) OR (Hypertrophic-neuropathy ADJ3 Dejerine-Sottas) OR (Hypertryptophanemia) OR (Hypocalcemia ADJ3 autosomal-dominant) OR (Hypochondroplasia) OR (Hypohidrotic-ectodermal-dysplasia-autosomal-recessive) OR (Hypohidrotic-ectodermal-dysplasia ADJ3 hypothyroidism ADJ3 ciliary-dyskinesia) OR (Hypokalemic-periodic-paralysis) OR (Hypomandibular-faciocranial-dysostosis) OR (Hypomelanosis ADJ3 Ito) OR (Hypomyelination ADJ3 congenital-cataract) OR (Hypomyelination ADJ3 atrophy ADJ3 basal-ganglia ADJ3 cerebellum) OR (Hypoparathyroidism-intellectual-disability-dysmorphism ADJ3 syndrom*) OR (Hypophosphatasia) OR (Hypophosphatemic-rickets) OR (Hypoplastic-left-heart ADJ3 syndrom*) OR (Hypospadias-familial) OR (Hypospadias-intellectual-disability ADJ3 Goldblatt-type ADJ3 syndrom*) OR (Hypotelorism-cleft-palate-hypospadias) OR (Hypotonia ADJ3 congenital-nystagmus ADJ3 ataxia ADJ3 abnormal-auditory-brainstem-response) OR (Hypotrichosis-simplex) OR (I-cell ADJ3 diseas*) OR (ICF ADJ3 syndrom*) OR (Ichthyosiform-erythroderma ADJ3 corneal-involvement ADJ3 deafness) OR (Ichthyosis-alopecia-eclabion-ectropion-intellectual-disability) OR (Ichthyosis-bullosa ADJ3 Siemens) OR (Ichthyosis-cheek-eyebrow ADJ3 syndrom*) OR (Ichthyosis-follicularis-atrichia-photophobia ADJ3 syndrom*) OR (Ichthyosis-hystrix ADJ3 Curth-Macklin) OR (Ichthyosis-lamellar-1) OR (Ichthyosis-lamellar-2) OR (Ichthyosis-lamellar-3) OR (Ichthyosis-lamellar ADJ3 autosomal-dominant) OR (Ichthyosis-prematurity ADJ3 syndrom*) OR (Ichthyosis-tapered-fingers-midline-groove-up) OR (Ichthyosis ADJ3 hypotrichosis ADJ3 autosomal-recessive) OR (Ichthyosis ADJ3 leukocyte-vacuoles ADJ3 alopecia ADJ3 -sclerosing-cholangitis) OR (Ichthyosis-intellectual-disability-dwarfism-renal-impairment) OR (Idiopathic-basal-ganglia-calcification-childhood-onset) OR (Idiopathic-CD4-positive-T-lymphocytopenia) OR (Iida-Kannari ADJ3 syndrom*) OR (IL12RB1 ADJ3 deficien*) OR (IMAGe ADJ3 syndrom*) OR (Iminoglycinuria) OR (Immune ADJ3 defect*-due-to-absence ADJ3 thymus) OR (Immunodeficiency ADJ3 hyper-IgM-type-1) OR (Immunodeficiency ADJ3 hyper-IgM-type-2) OR (Immunodeficiency ADJ3 hyper-IgM-type-3) OR (Immunodeficiency ADJ3 hyper-IgM-type-4) OR (Immunodeficiency ADJ3 hyper-IgM-type-5) OR (Immunodysregulation ADJ3 polyendocrinopathy ADJ3 enteropathy-X-linked) OR (Imperforate-oropharynx-costo-vetebral-anomalies) OR (Inclusion-body-myopathy-2) OR (Inclusion-body-myopathy-3) OR (Inclusion-body-myopathy ADJ3 early-onset-Paget ADJ3 diseas* ADJ3 frontotemporal-dementia) OR (Incontinentia-pigmenti) OR (Infantile-axonal-neuropathy) OR (Infantile-cerebellar-retinal-degeneration) OR (Infantile-choroidocerebral-calcification ADJ3 syndrom*) OR (Infantile-histiocytoid-cardiomyopathy) OR (Infantile-liver-failure ADJ3 syndrom*-1) OR (Infantile-myofibromatosis) OR (Infantile-neuroaxonal-dystroph*) OR (Infantile-onset-spinocerebellar-ataxia) OR (Infantile-spasms-broad-thumbs) OR (Infantile-onset-ascending-hereditary-spastic-paralysis) OR (Iniencephaly) OR (Insulin-like-growth-factor-1-resistance-to) OR (Insulin-like-growth-factor-I ADJ3 deficien*) OR (Insulin-resistance-type-B) OR (Intellectual-deficit---short-stature---hypertelorism) OR (Intellectual-deficit-Buenos-Aires) OR (Intellectual-disability---athetosis---microphthalmia) OR (Intellectual-disability---hypoplastic-corpus-callosum---preauricular-tag) OR (Intellectual-disability-developmental-delay-contractures ADJ3 syndrom*) OR (Intellectual-disability-dysmorphism-hypogonadism-diabetes-mellitus ADJ3 syndrom*) OR (Intellectual-disability-severe-speech-delay-mild-dysmorphism ADJ3 syndrom*) OR (Intellectual-disability-spasticity-ectrodactyly ADJ3 syndrom*) OR (Intermediate-congenital-nemaline-myopathy) OR (Intermediate-severe-Salla ADJ3 diseas*) OR (Intestinal-atresia-multiple) OR (Intrauterine-growth-retardation ADJ3 increased-mitomycin-C-sensitivity) OR (IRAK-4 ADJ3 deficien*) OR (Iridogoniodysgenesis ADJ3 skeletal-anomalies) OR (Iris-hypoplasia ADJ3 glaucoma) OR (Irons-Bhan ADJ3 syndrom*) OR (IRVAN ADJ3 syndrom*) OR (Isobutyryl-CoA-dehydrogenase ADJ3 deficien*) OR (Isodicentric-chromosome-15 ADJ3 syndrom*) OR (Isolated-ACTH ADJ3 deficien*) OR (Isolated-anterior-cervical-hypertrichosis) OR (Isolated-congenital-megalocornea) OR (Isolated-ectopia-lentis) OR (Isolated-growth-hormone ADJ3 deficien*-type-1A) OR (Isolated-growth-hormone ADJ3 deficien*-type-1B) OR (Isolated-growth-hormone ADJ3 deficien*-type-2) OR (Isolated-growth-hormone ADJ3 deficien*-type-3) OR (Isotretinoin-embryopathy-like ADJ3 syndrom*) OR (Isovaleric-acidemia) OR (Ivemark ADJ3 syndrom*) OR (IVIC ADJ3 syndrom*) OR (Jackson-Weiss ADJ3 syndrom*) OR (Jansen-type-metaphyseal-chondrodysplasia) OR (Jejunal-atresia) OR (Jejunal-atresia ADJ3 renal-adysplasia) OR (Jervell-Lange-Nielsen ADJ3 syndrom*) OR (Jeune ADJ3 syndrom*) OR (Johanson-Blizzard ADJ3 syndrom*) OR (Johnson-Munson ADJ3 syndrom*) OR (Johnson-neuroectodermal ADJ3 syndrom*) OR (Johnston-Aarons-Schelley ADJ3 syndrom*) OR (Jones ADJ3 syndrom*) OR (Joubert ADJ3 syndrom*) OR (Joubert ADJ3 syndrom* ADJ3 oculorenal-anomalies) OR (Juberg-Marsidi ADJ3 syndrom*) OR (Juberg-Hayward ADJ3 syndrom*) OR (Junctional-epidermolysis-bullosa-generalized-intermediate) OR (Junctional-epidermolysis-bullosa ADJ3 Herlitz) OR (Juvenile-amyotrophic-lateral-sclerosis) OR (Juvenile-osteoporosis) OR (Juvenile-Paget ADJ3 diseas*) OR (Juvenile-polyposis ADJ3 syndrom*) OR (Juvenile-primary-lateral-sclerosis) OR (Juvenile-retinoschisis) OR (Juvenile-onset-dystonia) OR (Kabuki ADJ3 syndrom*) OR (Kallmann ADJ3 syndrom*) OR (Kallmann ADJ3 syndrom*-1) OR (Kallmann ADJ3 syndrom*-2) OR (Kaplan-Plauchu-Fitch ADJ3 syndrom*) OR (Kapur-Toriello ADJ3 syndrom*) OR (Karak ADJ3 syndrom*) OR (Kartagener ADJ3 syndrom*) OR (Kaufman-oculocerebrofacial ADJ3 syndrom*) OR (KBG ADJ3 syndrom*) OR (KCNQ2-Related-Disorders) OR (Kearns-Sayre ADJ3 syndrom*) OR (Kennedy ADJ3 diseas*) OR (Kenny-Caffey ADJ3 syndrom*-type-1) OR (Kenny-Caffey ADJ3 syndrom*-type-2) OR (Keratoderma-palmoplantar-deafness) OR (Keratoderma-palmoplantar-spastic-paralysis) OR (Keratoderma-palmoplantaris-transgrediens) OR (Keratolytic-winter-erythema) OR (Keratosis-follicularis-dwarfism ADJ3 cerebral-atrophy) OR (Keratosis-follicularis-spinulosa-decalvans) OR (Kernicterus) OR (Keutel ADJ3 syndrom*) OR (KID ADJ3 syndrom*) OR (Kindler ADJ3 syndrom*) OR (King-Denborough ADJ3 syndrom*) OR (Kleeblattschaedel ADJ3 syndrom*) OR (Kleine-Levin ADJ3 syndrom*) OR (Kleiner-Holmes ADJ3 syndrom*) OR (Klippel-Feil ADJ3 syndrom*) OR (Kniest-dysplasia) OR (Kniest-like-dysplasia-lethal) OR (Kniest-like-dysplasia ADJ3 pursed-lips ADJ3 ectopia-lentis) OR (Knobloch ADJ3 syndrom*) OR (Knuckle-pads ADJ3 leuconychia ADJ3 sensorineural-deafness) OR (Kohlschutter-Tonz ADJ3 syndrom*) OR (Koone-Rizzo-Elias ADJ3 syndrom*) OR (Kosztolanyi ADJ3 syndrom*) OR (Kotzot-Richter ADJ3 syndrom*) OR (Kowarski ADJ3 syndrom*) OR (Kozlowski-Warren-Fisher ADJ3 syndrom*) OR (Kozlowski-Krajewska ADJ3 syndrom*) OR (Kuskokwim ADJ3 diseas*) OR (Kyphomelic-dysplasia) OR (Kyphoscoliotic-Ehlers-Danlos ADJ3 syndrom*) OR (L-2-hydroxyglutaric-aciduria) OR (L-arginine-glycine-amidinotransferase ADJ3 deficien*) OR (Laband ADJ3 syndrom*) OR (Lachiewicz-Sibley ADJ3 syndrom*) OR (Lacrimo-auriculo-dento-digital ADJ3 syndrom*) OR (Lafora ADJ3 diseas*) OR (Laing-distal-myopathy) OR (Lambdoid-synostosis) OR (Lambert ADJ3 syndrom*) OR (Lamellar-ichthyosis) OR (Landau-Kleffner ADJ3 syndrom*) OR (Langer-mesomelic-dysplasia) OR (Laron ADJ3 syndrom*) OR (Larsen ADJ3 syndrom*) OR (Larsen-like ADJ3 syndrom*) OR (Laryngomalacia) OR (Laryngoonychocutaneous ADJ3 syndrom*) OR (Larynx-atresia) OR (Larynx ADJ3 congenital-partial-atresia-of) OR (Late-onset-distal-myopathy ADJ3 Markesbery-Griggs) OR (Late-onset-junctional-epidermolysis-bullosa) OR (Late-onset-localized-junctional-epidermolysis-bullosa-intellectual-disability ADJ3 syndrom*) OR (Late-onset-retinal-degeneration) OR (Lateral-meningocele ADJ3 syndrom*) OR (Lateral-semicircular-canal-malformation ADJ3 familial ADJ3 external ADJ3 middle-ear-abnormalities) OR (Lathosterolosis) OR (Lattice-corneal-dystroph*-type-1) OR (Laurence-Moon ADJ3 syndrom*) OR (Laurin-Sandrow ADJ3 syndrom*) OR (LCHAD ADJ3 deficien*) OR (Le-Marec-Bracq-Picaud ADJ3 syndrom*) OR (Leber-congenital-amaurosis) OR (Leber-congenital-amaurosis-5) OR (Leber-hereditary-optic-neuropathy) OR (Leber-hereditary-optic-neuropathy ADJ3 dystonia) OR (Left-ventricular-noncompaction) OR (Left-sided-gallbladder) OR (Legg-Calve-Perthes ADJ3 diseas*) OR (Legius ADJ3 syndrom*) OR (Leigh ADJ3 syndrom*) OR (Leigh ADJ3 syndrom* ADJ3 French-Canadian) OR (Lelis ADJ3 syndrom*) OR (Lennox-Gastaut ADJ3 syndrom*) OR (Lenz-Majewski-hyperostotic-dwarfism) OR (Lenz-microphthalmia ADJ3 syndrom*) OR (LEOPARD ADJ3 syndrom*) OR (Leprechaunism) OR (Leri-pleonosteosis) OR (Leri-Weill-dyschondrosteosis) OR (Lethal-chondrodysplasia-Moerman) OR (Lethal-chondrodysplasia-Seller) OR (Lethal-congenital-contracture ADJ3 syndrom*-1) OR (Lethal-congenital-contracture ADJ3 syndrom*-2) OR (Lethal-congenital-contracture ADJ3 syndrom*-3) OR (Lethal-short-limb-skeletal-dysplasia-Al-Gazali) OR (Leukocyte-adhesion ADJ3 deficien*-type-1) OR (Leukodystroph*) OR (Leukoencephalopathy ADJ3 dystonia ADJ3 motor-neuropathy) OR (Leukoencephalopathy-palmoplantar-keratoderma) OR (Leukoencephalopathy ADJ3 brain-stem ADJ3 spinal-cord-involvement ADJ3 lactate-elevation) OR (Leukoencephalopathy ADJ3 thalamus ADJ3 brainstem-involvement ADJ3 high-lactate) OR (Leukoencephalopathy ADJ3 vanishing-white-matter) OR (Leukoencephalopathy-spondylometaphyseal-dysplasia ADJ3 syndrom*) OR (Leukonychia-totalis) OR (Levic-Stefanovic-Nikolic ADJ3 syndrom*) OR (Lhermitte-Duclos ADJ3 diseas*) OR (Li-Fraumeni ADJ3 syndrom*) OR (Lichtenstein ADJ3 syndrom*) OR (Ligneous-conjunctivitis) OR (Limb-deficiencies-distal ADJ3 micrognathia) OR (Limb-body-wall-complex) OR (Limb-girdle-muscular-dystroph*-type-1A) OR (Limb-girdle-muscular-dystroph*-type-1B) OR (Limb-girdle-muscular-dystroph*-type-1C) OR (Limb-girdle-muscular-dystroph*-type-1D) OR (Limb-girdle-muscular-dystroph*-type-1E) OR (Limb-girdle-muscular-dystroph*-type-1F) OR (Limb-girdle-muscular-dystroph*-type-1G) OR (Limb-girdle-muscular-dystroph*-type-1H) OR (Limb-girdle-muscular-dystroph*-type-2A) OR (Limb-girdle-muscular-dystroph*-type-2B) OR (Limb-girdle-muscular-dystroph*-type-2E) OR (Limb-girdle-muscular-dystroph*-type-2F) OR (Limb-girdle-muscular-dystroph*-type-2H) OR (Limb-girdle-muscular-dystroph*-type-2I) OR (Limb-girdle-muscular-dystroph*-type-2J) OR (Limb-girdle-muscular-dystroph*-type-2K) OR (Limb-girdle-muscular-dystroph*-type-2L) OR (Limb-girdle-muscular-dystroph*-type-2M) OR (Limb-girdle-muscular-dystroph*-type-2N) OR (Limb-girdle-muscular-dystroph*-type-2O) OR (Limb-girdle-muscular-dystroph*-type-2P-type-1A) OR (Limb-girdle-muscular-dystroph*-type-2Q) OR (Limb-girdle-muscular-dystroph*-type-2S) OR (Limb-girdle-muscular-dystroph*-type-2T) OR (Limb-girdle-muscular-dystroph* ADJ3 type-2C) OR (Limb-girdle-muscular-dystroph* ADJ3 type-2D) OR (Limb-girdle-muscular-dystroph* ADJ3 type-2G) OR (Limb-mammary ADJ3 syndrom*) OR (Lin-Gettig ADJ3 syndrom*) OR (Linear ADJ3 whorled-nevoid-hypermelanosis) OR (Linear-nevus-sebaceous ADJ3 syndrom*) OR (LIPE-related-familial-partial-lipodystroph*) OR (Lipodystroph* ADJ3 familial-partial ADJ3 type-5) OR (Lipoic-acid-synthetase ADJ3 deficien*) OR (Lipoid-proteinosis ADJ3 Urbach ADJ3 Wiethe) OR (Localized-junctional-epidermolysis-bullosa ADJ3 non-Herlitz) OR (Long-QT ADJ3 syndrom*-1) OR (Loose-anagen-hair ADJ3 syndrom*) OR (Lopes-Gorlin ADJ3 syndrom*) OR (Lowe-oculocerebrorenal ADJ3 syndrom*) OR (Lowry-Maclean ADJ3 syndrom*) OR (Lowry-Wood ADJ3 syndrom*) OR (LRBA ADJ3 deficien*) OR (Lubinsky ADJ3 syndrom*) OR (Lucey-Driscoll ADJ3 syndrom*) OR (Lujan ADJ3 syndrom*) OR (Lung-agenesis) OR (Lymphangioleiomyomatosis) OR (Lymphedema ADJ3 microcephaly ADJ3 chorioretinopathy ADJ3 syndrom*) OR (Lysinuric-protein-intolerance) OR (Mac-Dermot-Winter ADJ3 syndrom*) OR (Macrocephaly ADJ3 benign-familial) OR (Macrocephaly-short-stature-paraplegia ADJ3 syndrom*) OR (Macrodactyly ADJ3 fingers) OR (Macrodactyly ADJ3 toes) OR (Macroepiphyseal-dysplasia ADJ3 osteoporosis ADJ3 wrinkled-skin ADJ3 -aged-appearance) OR (Macroglossia) OR (Macrosomia ADJ3 lethal-microphthalmia) OR (Macrozoospermia) OR (Macular-dystroph* ADJ3 corneal-type-1) OR (Macules-hereditary-congenital-hypopigmented ADJ3 hyperpigmented) OR (Madokoro-Ohdo-Sonoda ADJ3 syndrom*) OR (Male-pseudohermaphroditism-due-to ADJ3 defect*ive-LH-molecule) OR (Male-pseudohermaphroditism-intellectual-disability ADJ3 syndrom* ADJ3 Verloes) OR (Malignant-Atrophic-Papulosis) OR (Malignant-hyperthermia) OR (Malignant-hyperthermia-arthrogryposis-torticollis) OR (Malignant-migrating-partial-seizures ADJ3 infancy) OR (Malonyl-CoA-decarboxylase ADJ3 deficien*) OR (MAN1B1-CDG) OR (Mandibuloacral-dysplasia ADJ3 type-A-lipodystroph*) OR (Mandibuloacral-dysplasia ADJ3 type-B-lipodystroph*) OR (Mandibulofacial-dysostosis ADJ3 microcephaly) OR (Manitoba-oculotrichoanal ADJ3 syndrom*) OR (Mannosidosis ADJ3 beta-A ADJ3 lysosomal) OR (Manouvrier ADJ3 syndrom*) OR (Marden-Walker ADJ3 syndrom*) OR (Marfan ADJ3 syndrom*) OR (Marfanoid-habitus-autosomal-recessive-intellectual-disability ADJ3 syndrom*) OR (Marie-Unna-congenital-hypotrichosis) OR (Marinesco-Sjogren ADJ3 syndrom*) OR (Marshall ADJ3 syndrom*) OR (Marshall-Smith ADJ3 syndrom*) OR (Martsolf ADJ3 syndrom*) OR (Mastocytosis-cutaneous ADJ3 short-stature-conductive-hearing-loss ADJ3 microtia) OR (Maternal-hyperphenylalaninemia) OR (Maternally-inherited-diabetes ADJ3 deafness) OR (Maturity-onset-diabetes ADJ3 young) OR (Maturity-onset-diabetes ADJ3 young ADJ3 type-1) OR (Maturity-onset-diabetes ADJ3 young ADJ3 type-2) OR (Maturity-onset-diabetes ADJ3 young ADJ3 type-3) OR (Maturity-onset-diabetes ADJ3 young ADJ3 type-4) OR (Maturity-onset-diabetes ADJ3 young ADJ3 type-5) OR (Maturity-onset-diabetes ADJ3 young ADJ3 type-6) OR (Maturity-onset-diabetes ADJ3 young ADJ3 type-7) OR (Maturity-onset-diabetes ADJ3 young ADJ3 type-8) OR (Maturity-onset-diabetes ADJ3 young ADJ3 type-9) OR (Maxillonasal-dysplasia ADJ3 Binder) OR (McCune-Albright ADJ3 syndrom*) OR (McDonough ADJ3 syndrom*) OR (McKusick-Kaufman ADJ3 syndrom*) OR (McPherson-Clemens ADJ3 syndrom*) OR (Meacham-Winn-Culler ADJ3 syndrom*) OR (Meckel ADJ3 syndrom*) OR (MECP2 ADJ3 duplicat* ADJ3 syndrom*) OR (Medeira-Dennis-Donnai ADJ3 syndrom*) OR (Median-cleft ADJ3 upper-lip ADJ3 polyps ADJ3 facial-skin ADJ3 nasal-mucosa) OR (Median-nodule ADJ3 upper-lip) OR (Medium-chain-acyl-coenzyme-A-dehydrogenase ADJ3 deficien*) OR (Medrano-Roldan ADJ3 syndrom*) OR (Medulloblastoma) OR (Meesmann-corneal-dystroph*) OR (Megacystis-microcolon-intestinal-hypoperistalsis ADJ3 syndrom*) OR (Megaduodenum) OR (megacystis) OR (Megaepiphyseal-dwarfism) OR (Megalencephalic-leukoencephalopathy ADJ3 subcortical-cysts) OR (Megalencephaly-Polymicrogyria-Polydactyly-Hydrocephalus ADJ3 syndrom*) OR (Megalocornea---spherophakia---secondary-glaucoma) OR (Megalocornea-intellectual-disability ADJ3 syndrom*) OR (Megarbane-Jalkh ADJ3 syndrom*) OR (Megarbane ADJ3 syndrom*) OR (Mehes ADJ3 syndrom*) OR (MEHMO ADJ3 syndrom*) OR (Mehta-Lewis-Patton ADJ3 syndrom*) OR (Meier-Gorlin ADJ3 syndrom*) OR (Meige ADJ3 syndrom*) OR (Meleda ADJ3 diseas*) OR (Melnick-Needles ADJ3 syndrom*) OR (Melorheostosis) OR (Melorheostosis ADJ3 osteopoikilosis) OR (Meningocele) OR (Menkes ADJ3 diseas*) OR (Mesomelia-synostoses ADJ3 syndrom*) OR (Mesomelic-dwarfism-cleft-palate-camptodactyly) OR (Mesomelic-dwarfism ADJ3 hypoplastic-tibia ADJ3 radius) OR (Mesomelic-dysplasia-Kantaputra) OR (Mesomelic-dysplasia-Savarirayan) OR (Metacarpals-4 ADJ3 5-fusion) OR (Metachondromatosis) OR (Metaphyseal-acroscyphodysplasia) OR (Metaphyseal-chondrodysplasia-Schmid) OR (Metaphyseal-chondrodysplasia-Spahr) OR (Metaphyseal-dysostosis-intellectual-disability-conductive-deafness ADJ3 syndrom*) OR (Metaphyseal-dysplasia-maxillary-hypoplasia-brachydactyly) OR (Metaphyseal-dysplasia-without-hypotrichosis) OR (Metaphyseal-undermodeling ADJ3 spondylar-dysplasia ADJ3 -overgrowth) OR (Metatropic-dysplasia) OR (Methimazole-antenatal-exposure) OR (Methionine-adenosyltransferase ADJ3 deficien*) OR (Methylmalonic-aciduria ADJ3 cblA) OR (Methylmalonic-aciduria ADJ3 cblB) OR (Mevalonic-aciduria) OR (MGAT2-CDG-) OR (CDG-IIa) OR (Micro ADJ3 syndrom*) OR (Microbrachycephaly-ptosis-cleft-lip) OR (Microcephalic-osteodysplastic-primordial-dwarfism-type-1) OR (Microcephalic-osteodysplastic-primordial-dwarfism-type-2) OR (Microcephalic-primordial-dwarfism-Toriello) OR (Microcephalic-primordial-dwarfism ADJ3 Montreal) OR (Microcephaly-autosomal-dominant) OR (Microcephaly-brain ADJ3 defect*-spasticity-hypernatremia) OR (Microcephaly-cervical-spine-fusion-anomalies) OR (Microcephaly-deafness ADJ3 syndrom*) OR (Microcephaly-glomerulonephritis-Marfanoid-habitus) OR (Microcephaly-microcornea ADJ3 syndrom*-Seemanova) OR (Microcephaly-micropenis-convulsions) OR (Microcephaly-microphthalmos-blindness) OR (Microcephaly-nonsyndromal) OR (Microcephaly ADJ3 seizures ADJ3 -developmental-delay) OR (Microcephaly-albinism-digital-anomalies ADJ3 syndrom*) OR (Microcephaly-cardiomyopathy) OR (Microduplication-Xp11#22-p11#23 ADJ3 syndrom*) OR (Microgastria-limb-reduction ADJ3 defect*) OR (Microhydranencephaly) OR (Microphthalmia-associated ADJ3 colobomatous-cyst) OR (Microphthalmia-syndromic-10) OR (Microphthalmia-syndromic-4) OR (Microphthalmia-syndromic-5) OR (Microphthalmia-syndromic-6) OR (Microphthalmia-syndromic-8) OR (Microphthalmia-syndromic-9) OR (Microphthalmia ADJ3 linear-skin ADJ3 defect*s ADJ3 syndrom*) OR (Microsomia-hemifacial-radial ADJ3 defect*s) OR (Microtia-eye-coloboma ADJ3 imperforation ADJ3 nasolacrimal-duct) OR (Microtia ADJ3 meatal-atresia ADJ3 conductive-deafness) OR (Microtia-Anotia) OR (Microvillus-inclusion ADJ3 diseas*) OR (Midphalangeal-hair) OR (Mild-phenylketonuria) OR (Miller ADJ3 syndrom*) OR (Miller-Dieker ADJ3 syndrom*) OR (Milner-Khallouf-Gibson ADJ3 syndrom*) OR (Minicore-myopathy ADJ3 external-ophthalmoplegia) OR (Minicore-myopathy ADJ3 antenatal-onset ADJ3 arthrogryposis) OR (Mitochondrial-complex-I ADJ3 deficien*) OR (Mitochondrial-complex-II ADJ3 deficien*) OR (Mitochondrial-complex-III ADJ3 deficien*) OR (Mitochondrial-DNA-depletion ADJ3 syndrom* ADJ3 encephalomyopathic-form ADJ3 methylmalonic-aciduria) OR (Mitochondrial-DNA-associated-Leigh ADJ3 syndrom*) OR (Mitochondrial-encephalomyopathy-lactic-acidosis ADJ3 stroke-like-episodes) OR (Mitochondrial-genetic-disorders) OR (Mitochondrial-Membrane-Protein-Associated-Neurodegeneration) OR (Mitochondrial-myopathy ADJ3 diabetes) OR (Mitochondrial-myopathy ADJ3 lactic-acidosis) OR (Mitochondrial-neurogastrointestinal-encephalopathy ADJ3 syndrom*) OR (Mitochondrial-trifunctional-protein ADJ3 deficien*) OR (Mitral-atresia) OR (Mitral-regurgitation ADJ3 conductive-deafness ADJ3 -fusion ADJ3 cervical-vertebrae ADJ3 carpal ADJ3 tarsal-bones) OR (Mitral-valve-prolapse ADJ3 familial ADJ3 autosomal-dominant) OR (Miyoshi-myopathy) OR (Moebius ADJ3 syndrom*) OR (MOGS-CDG-) OR (CDG-IIb) OR (Mohr-Tranebjaerg ADJ3 syndrom*) OR (Moloney ADJ3 syndrom*) OR (Molybdenum-cofactor ADJ3 deficien*) OR (MOMO ADJ3 syndrom*) OR (Monilethrix) OR (Monoamine-oxidase-A ADJ3 deficien*) OR (Morgagni-Stewart-Morel ADJ3 syndrom*) OR (MORM ADJ3 syndrom*) OR (Morning-glory ADJ3 syndrom*) OR (Morse-Rawnsley-Sargent ADJ3 syndrom*) OR (Mosaic-variegated-aneuploidy ADJ3 syndrom*) OR (Mounier-Kuhn ADJ3 syndrom*) OR (Mousa-Al-din-Al-Nassar ADJ3 syndrom*) OR (Mowat-Wilson ADJ3 syndrom*) OR (Moyamoya ADJ3 diseas*) OR (MPDU1-CDG-) OR (CDG-If) OR (MPV17-related-hepatocerebral-mitochondrial-DNA-depletion ADJ3 syndrom*) OR (Muckle-Wells ADJ3 syndrom*) OR (Mucolipidosis-III-alpha) OR (Mucolipidosis-III-beta) OR (Mucolipidosis-type-4) OR (Mucopolysaccharidosis-type-III) OR (Mucopolysaccharidosis-type-IIIA) OR (Mucopolysaccharidosis-type-IIIB) OR (Mucopolysaccharidosis-type-IIIC) OR (Mucopolysaccharidosis-type-IIID) OR (Mucopolysaccharidosis-type-IV) OR (Mucopolysaccharidosis-type-IVA) OR (Mucopolysaccharidosis-type-VII) OR (Muenke ADJ3 syndrom*) OR (Muir-Torre ADJ3 syndrom*) OR (Mulibrey-Nanism) OR (Muller-Barth-Menger ADJ3 syndrom*) OR (Multicentric-carpotarsal-osteolysis ADJ3 syndrom*) OR (Multicystic-renal-dysplasia ADJ3 bilateral) OR (Multiple-cafe-au-lait-spots) OR (Multiple-congenital-anomalies-hypotonia-seizures ADJ3 syndrom*) OR (Multiple-congenital-anomalies-hypotonia-seizures ADJ3 syndrom*-type-2) OR (Multiple-epiphyseal-dysplasia-1) OR (Multiple-epiphyseal-dysplasia-2) OR (Multiple-epiphyseal-dysplasia-3) OR (Multiple-epiphyseal-dysplasia-4) OR (Multiple-epiphyseal-dysplasia-5) OR (Multiple-fibrofolliculoma-familial) OR (Multiple-pterygium ADJ3 syndrom*-Escobar) OR (Multiple-pterygium ADJ3 syndrom*-lethal) OR (Multiple-pterygium ADJ3 syndrom*-X-linked) OR (Multiple-sulfatase ADJ3 deficien*) OR (Multiple-symmetric-lipomatosis) OR (Multiple-synostoses ADJ3 syndrom*-1) OR (Multiple-synostoses ADJ3 syndrom*-2) OR (Multiple-system-atrophy) OR (Mungan ADJ3 syndrom*) OR (MURCS-association) OR (Muscle-eye-brain ADJ3 diseas*) OR (Muscular-atrophy-ataxia-retinitis-pigmentosa ADJ3 diabetes-mellitus) OR (Muscular-dystroph*-white-matter-spongiosis) OR (Muscular-dystroph* ADJ3 congenital ADJ3 megaconial) OR (Muscular-phosphorylase-kinase ADJ3 deficien*) OR (Musculocontractural-Ehlers-Danlos ADJ3 syndrom*) OR (MYD88 ADJ3 deficien*) OR (Myelocerebellar-disorder) OR (MYH7-related-scapuloperoneal-myopathy) OR (Myhre ADJ3 syndrom*) OR (Myoclonic-epilepsy ADJ3 ragged-red-fibers) OR (Myoclonus-cerebellar-ataxia-deafness) OR (Myoclonus-dystonia) OR (Myoglobinuria-recurrent) OR (Myokymia ADJ3 neonatal-epilepsy) OR (Myopathic-carnitine ADJ3 deficien*) OR (Myopathy ADJ3 extrapyramidal-signs) OR (Myosin-storage-myopathy) OR (Myotonia-congenita) OR (Myotonic-dystroph*-type-1) OR (Myotonic-dystroph*-type-2) OR (N ADJ3 syndrom*) OR (N-acetylglutamate-synthase ADJ3 deficien*) OR (Naegeli ADJ3 syndrom*) OR (Nager-acrofacial-dysostosis) OR (Naguib-Richieri-Costa ADJ3 syndrom*) OR (Nail-dysplasia ADJ3 isolated-congenital) OR (Nail-patella ADJ3 syndrom*) OR (Nakajo-Nishimura ADJ3 syndrom*) OR (Nakajo ADJ3 syndrom*) OR (Nance-Horan ADJ3 syndrom*) OR (Nasodigitoacoustic ADJ3 syndrom*) OR (Nathalie ADJ3 syndrom*) OR (Naxos ADJ3 diseas*) OR (NBIA ADJ3 DYT ADJ3 PARK-PLA2G6) OR (Neonatal-adrenoleukodystroph*) OR (Neonatal-intrahepatic-cholestasis-caused-by-citrin ADJ3 deficien*) OR (Neonatal-Onset-Multisystem-Inflammatory ADJ3 diseas*) OR (Neonatal-progeroid ADJ3 syndrom*) OR (Neonatal-severe-hyperparathyroidism) OR (Nephrogenic-diabetes-insipidus) OR (Nephrogenic-diabetes-insipidus-intracranial-calcification-facial-dysmorphism ADJ3 syndrom*) OR (Nephropathy ADJ3 deafness ADJ3 -hyperparathyroidism) OR (Nestor-guillermo-progeria ADJ3 syndrom*) OR (Netherton ADJ3 syndrom*) OR (Neu-Laxova ADJ3 syndrom*) OR (Neurofaciodigitorenal ADJ3 syndrom*) OR (Neuroferritinopathy) OR (Neurofibromatosis-type-2) OR (Neurofibromatosis-Noonan ADJ3 syndrom*) OR (Neuronal-ceroid-lipofuscinosis-10) OR (Neuronal-ceroid-lipofuscinosis-2) OR (Neuronal-ceroid-lipofuscinosis-3) OR (Neuronal-ceroid-lipofuscinosis-5) OR (Neuronal-ceroid-lipofuscinosis-6) OR (Neuronal-ceroid-lipofuscinosis-7) OR (Neuronal-ceroid-lipofuscinosis-9) OR (Neuronal-intranuclear-inclusion ADJ3 diseas*) OR (Neuropathy-ataxia-retinitis-pigmentosa ADJ3 syndrom*) OR (Neuropathy ADJ3 congenital ADJ3 arthrogryposis-multiplex) OR (Neuropathy ADJ3 distal-hereditary-motor ADJ3 Jerash) OR (Neuropathy ADJ3 hereditary-motor ADJ3 sensory ADJ3 Okinawa) OR (Neuropathy ADJ3 hereditary-motor ADJ3 sensory ADJ3 Russe) OR (Neutral-lipid-storage ADJ3 diseas* ADJ3 myopathy) OR (Neutrophil-specific-granule ADJ3 deficien*) OR (Nevoid-basal-cell-carcinoma ADJ3 syndrom*) OR (Nevus-comedonicus ADJ3 syndrom*) OR (Nguyen ADJ3 syndrom*) OR (Nicolaides-Baraitser ADJ3 syndrom*) OR (Niemann-Pick ADJ3 diseas*-type-A) OR (Niemann-Pick ADJ3 diseas*-type-B) OR (Niemann-Pick ADJ3 diseas*-type-C1) OR (Niemann-Pick ADJ3 diseas*-type-C2) OR (Nievergelt ADJ3 syndrom*) OR (Night-blindness-skeletal-anomalies-dysmorphism ADJ3 syndrom*) OR (Nijmegen-breakage ADJ3 syndrom*) OR (Nonbullous-congenital-ichthyosiform-erythroderma) OR (Nonsyndromic-hereditary-sensorineural-hearing-loss) OR (Noonan-like ADJ3 syndrom* ADJ3 loose-anagen-hair) OR (Norrie ADJ3 diseas*) OR (North-Carolina-macular-dystroph*) OR (Northern-epilepsy) OR (Not-otherwise-specified-3-MGA-uria) OR (Obesity-due-to-congenital-leptin ADJ3 deficien*) OR (Occipital-horn ADJ3 syndrom*) OR (Ochoa ADJ3 syndrom*) OR (Ocular-albinism-type-1) OR (Oculo-skeletal-renal ADJ3 syndrom*) OR (Oculoauriculofrontonasal ADJ3 syndrom*) OR (Oculocerebral ADJ3 syndrom* ADJ3 hypopigmentation) OR (Oculocerebrocutaneous ADJ3 syndrom*) OR (Oculocutaneous-albinism-type-1) OR (Oculocutaneous-albinism-type-1B) OR (Oculocutaneous-albinism-type-2) OR (Oculocutaneous-albinism-type-3) OR (Oculodentodigital-dysplasia) OR (Oculoectodermal ADJ3 syndrom*) OR (Oculofaciocardiodental ADJ3 syndrom*) OR (Oculomaxillofacial-dysostosis) OR (Oculomotor-apraxia-Cogan) OR (Oculopharyngeal-muscular-dystroph*) OR (Oculopharyngodistal-myopathy) OR (Oculorenocerebellar ADJ3 syndrom*) OR (Odonto-onycho-dysplasia ADJ3 alopecia) OR (Odontoma-dysphagia ADJ3 syndrom*) OR (Odontomicronychial-dysplasia) OR (Odontoonychodermal-dysplasia) OR (Odontotrichomelic ADJ3 syndrom*) OR (Oguchi ADJ3 diseas*) OR (Okamoto ADJ3 syndrom*) OR (Oligodactyly-tetramelic-postaxial) OR (Oligomeganephronic-renal-hypoplasia) OR (Oliver ADJ3 syndrom*) OR (Olivopontocerebellar-atrophy-deafness) OR (Ollier ADJ3 diseas*) OR (Olmsted ADJ3 syndrom*) OR (Omenn ADJ3 syndrom*) OR (Omodysplasia-1) OR (Omodysplasia-2) OR (Omphalocele-cleft-palate ADJ3 syndrom*-lethal) OR (Omphalocele ADJ3 exstrophy ADJ3 cloaca ADJ3 imperforate-anus ADJ3 -spinal ADJ3 defect*s-complex) OR (Omphalomesenteric-cyst) OR (Onychodystroph*-anonychia) OR (Onychotrichodysplasia ADJ3 neutropenia) OR (OPA3 ADJ3 defect*) OR (OPHN1 ADJ3 syndrom*) OR (Opsismodysplasia) OR (Optic-atrophy-1) OR (Optic-atrophy-2) OR (Optic-atrophy-polyneuropathy-deafness) OR (Ornithine-transcarbamylase ADJ3 deficien*) OR (Ornithine-translocase ADJ3 deficien* ADJ3 syndrom*) OR (Orofaciodigital ADJ3 syndrom*-1) OR (Orofaciodigital ADJ3 syndrom*-10) OR (Orofaciodigital ADJ3 syndrom*-11) OR (Orofaciodigital ADJ3 syndrom*-12) OR (Orofaciodigital ADJ3 syndrom*-13) OR (Orofaciodigital ADJ3 syndrom*-2) OR (Orofaciodigital ADJ3 syndrom*-3) OR (Orofaciodigital ADJ3 syndrom*-4) OR (Orofaciodigital ADJ3 syndrom*-5) OR (Orofaciodigital ADJ3 syndrom*-6) OR (Orofaciodigital ADJ3 syndrom*-8) OR (Orofaciodigital ADJ3 syndrom*-9) OR (Orofaciodigital ADJ3 syndrom*s) OR (Orthostatic-intolerance-due-to-NET ADJ3 deficien*) OR (Oslam ADJ3 syndrom*) OR (OSMED ADJ3 syndrom*) OR (Ossicular-Malformations ADJ3 familial) OR (Osteoarthropathy ADJ3 fingers-familial) OR (Osteodysplasia-familial-Anderson) OR (Osteofibrous-dysplasia) OR (Osteogenesis-imperfecta) OR (Osteogenesis-imperfecta-type-I) OR (Osteogenesis-imperfecta-type-II) OR (Osteogenesis-imperfecta-type-III) OR (Osteogenesis-imperfecta-type-IV) OR (Osteogenesis-imperfecta-type-IX) OR (Osteogenesis-imperfecta-type-V) OR (Osteogenesis-imperfecta-type-VI) OR (Osteogenesis-imperfecta-type-VII) OR (Osteogenesis-imperfecta-type-VIII) OR (Osteoglophonic-dysplasia) OR (Osteomesopyknosis) OR (Osteopathia-striata ADJ3 cranial-sclerosis) OR (Osteopathia-striata ADJ3 pigmentary-dermopathy-including-white-forelock) OR (Osteopenia ADJ3 sparse-hair) OR (Osteopetrosis) OR (Osteopetrosis ADJ3 infantile-neuroaxonal-dystroph*) OR (Osteopetrosis-autosomal-dominant-type-1) OR (Osteopetrosis-autosomal-dominant-type-2) OR (Osteopetrosis-autosomal-recessive-1) OR (Osteopetrosis-autosomal-recessive-2) OR (Osteopetrosis-autosomal-recessive-3) OR (Osteopetrosis-autosomal-recessive-4) OR (Osteopetrosis-autosomal-recessive-5) OR (Osteopetrosis-autosomal-recessive-6) OR (Osteopetrosis-autosomal-recessive-7) OR (Osteopoikilosis ADJ3 dacryocystitis) OR (Osteoporosis-oculocutaneous-hypopigmentation ADJ3 syndrom*) OR (Osteoporosis-pseudoglioma ADJ3 syndrom*) OR (Ostium-secundum-atrial-septal ADJ3 defect*) OR (Oto-palato-digital ADJ3 syndrom*-type-1) OR (Oto-palato-digital ADJ3 syndrom*-type-2) OR (Otodental-dysplasia) OR (Otofaciocervical ADJ3 syndrom*) OR (Otoonychoperoneal ADJ3 syndrom*) OR (Ouvrier-Billson ADJ3 syndrom*) OR (Pachydermoperiostosis) OR (Pachygyria) OR (Pachygyria ADJ3 frontotemporal) OR (Pachygyria-intellectual-disability-epilepsy ADJ3 syndrom*) OR (Pachyonychia-congenita) OR (Pacman-dysplasia) OR (PACS1-related ADJ3 syndrom*) OR (PAGOD ADJ3 syndrom*) OR (Pagon-Stephan ADJ3 syndrom*) OR (Paine ADJ3 syndrom*) OR (Palant-cleft-palate ADJ3 syndrom*) OR (Palatopharyngeal-incompetence) OR (Pallidopyramidal ADJ3 syndrom*) OR (Pallister-W ADJ3 syndrom*) OR (Pallister-Hall ADJ3 syndrom*) OR (Palmer-Pagon ADJ3 syndrom*) OR (Palmoplantar-keratoderma-sclerodactyly ADJ3 syndrom*) OR (Panhypopituitarism-X-linked) OR (Panostotic-fibrous-dysplasia) OR (Papillary-renal-cell-carcinoma) OR (Papillon-Lefevre ADJ3 syndrom*) OR (Paraganglioma ADJ3 gastric-stromal-sarcoma) OR (Paramyotonia-congenita) OR (PARC ADJ3 syndrom*) OR (Parkinson ADJ3 diseas*-type-9) OR (Paroxysmal-exertion-induced-dyskinesia) OR (Paroxysmal-extreme-pain-disorder) OR (Paroxysmal-kinesigenic-choreoathetosis) OR (Paroxysmal-ventricular-fibrillation) OR (Paroxysomal-nonkinesigenic-dyskinesia) OR (Partial-androgen-insensitivity ADJ3 syndrom*) OR (Partington ADJ3 syndrom*) OR (PASLI ADJ3 diseas*) OR (Paternal-uniparental-disomy ADJ3 chromosome-14) OR (Patterson-Stevenson-Fontaine ADJ3 syndrom*) OR (PCDH19-related-female-limited-epilepsy) OR (Pectus-carinatum) OR (Pelger-Huet-anomaly) OR (Pelvic-dysplasia-arthrogryposis ADJ3 lower-limbs) OR (Pendred ADJ3 syndrom*) OR (Pentalogy ADJ3 Cantrell) OR (Pentosuria) OR (Periodic-fever ADJ3 aphthous-stomatitis ADJ3 pharyngitis ADJ3 adenitis) OR (Periodontal-Ehlers-Danlos ADJ3 syndrom*) OR (Peripheral-resistance-to-thyroid-hormones) OR (Periventricular-heterotopia) OR (Perlman ADJ3 syndrom*) OR (Permanent-neonatal-diabetes-mellitus) OR (Peroxisomal-biogenesis-disorders) OR (Perrault ADJ3 syndrom*) OR (Perry ADJ3 syndrom*) OR (Persistent-Mullerian-duct ADJ3 syndrom*) OR (Peters-plus ADJ3 syndrom*) OR (Peutz-Jeghers ADJ3 syndrom*) OR (Pfeiffer-Mayer ADJ3 syndrom*) OR (Pfeiffer-Palm-Teller ADJ3 syndrom*) OR (Pfeiffer ADJ3 syndrom*) OR (Pfeiffer-Tietze-Welte ADJ3 syndrom*) OR (Pfeiffer-type-cardiocranial ADJ3 syndrom*) OR (PGM1-CDG) OR (PGM3-CDG) OR (PHAVER ADJ3 syndrom*) OR (Phenobarbital-antenatal-exposure) OR (Phocomelia-ectrodactyly-deafness-sinus-arrhythmia) OR (Phosphoglycerate-mutase ADJ3 deficien*) OR (Phosphoserine-aminotransferase ADJ3 deficien*) OR (Piebaldism) OR (Pierre-Robin-sequence) OR (Pierre-Robin-sequence ADJ3 pectus-excavatum ADJ3 rib ADJ3 scapular-anomalies) OR (Pierson ADJ3 syndrom*) OR (Pili-annulati) OR (Pili-torti) OR (Pili-torti-developmental-delay-neurological-abnormalities) OR (Pillay ADJ3 syndrom*) OR (Pilodental-dysplasia ADJ3 refractive-errors) OR (Pinheiro-Freire-Maia-Miranda ADJ3 syndrom*) OR (Pitt-Hopkins ADJ3 syndrom*) OR (Pitt-Hopkins-like ADJ3 syndrom*) OR (Pituitary-dwarfism ADJ3 large-sella-turcica) OR (Pituitary-hormone ADJ3 deficien* ADJ3 combined-3) OR (Pituitary-hormone ADJ3 deficien* ADJ3 combined-4) OR (Pituitary-stalk-interruption ADJ3 syndrom*) OR (Pityriasis-rubra-pilaris) OR (Plagiocephaly) OR (Platyspondylic-lethal-skeletal-dysplasia-Torrance) OR (PMM2-CDG-) OR (CDG-Ia) OR (Poland ADJ3 syndrom*) OR (POLR3-Related-Leukodystroph*) OR (Polycystic-kidneys ADJ3 severe-infantile ADJ3 tuberous-sclerosis) OR (Polycystic-lipomembranous-osteodysplasia ADJ3 sclerosing-leukoencephalopathy) OR (Polycystic-liver ADJ3 diseas*) OR (Polydactyly) OR (Polydactyly-myopia ADJ3 syndrom*) OR (Polyneuropathy-intellectual-disability-acromicria-premature-menopause ADJ3 syndrom*) OR (Polyosteolysis ADJ3 hyperostosis ADJ3 syndrom*) OR (Polyostotic-osteolytic-dysplasia ADJ3 hereditary-expansile) OR (Polysyndactyly-cardiac-malformation) OR (Pontine-tegmental-cap-dysplasia) OR (Pontocerebellar-hypoplasia-type-1) OR (Pontocerebellar-hypoplasia-type-2) OR (Pontocerebellar-hypoplasia-type-3) OR (Pontocerebellar-hypoplasia-type-4) OR (Pontocerebellar-hypoplasia-type-5) OR (Pontocerebellar-hypoplasia-type-6) OR (Popliteal-pterygium ADJ3 syndrom*) OR (Popliteal-pterygium ADJ3 syndrom* ADJ3 Bartsocas-Papas) OR (Porokeratosis ADJ3 Mibelli) OR (Porokeratosis ADJ3 disseminated-superficial-actinic-2) OR (Porphyria-cutanea-tarda) OR (Posterior-column-ataxia ADJ3 retinitis-pigmentosa) OR (Postnatal-progressive-microcephaly ADJ3 seizures ADJ3 -brain-atrophy) OR (Potassium-aggravated-myotonia) OR (Potato-nose) OR (Potocki-Lupski ADJ3 syndrom*) OR (Potter-sequence) OR (PPM-X ADJ3 syndrom*) OR (Prader-Willi-habitus ADJ3 osteopenia ADJ3 -camptodactyly) OR (Prader-Willi ADJ3 syndrom*) OR (Preaxial ADJ3 deficien* ADJ3 postaxial-polydactyly ADJ3 hypospadias) OR (Preaxial-polydactyly-type-1) OR (Preaxial-polydactyly-type-2) OR (Preaxial-polydactyly-type-3) OR (Preaxial-polydactyly-type-4) OR (Pretibial-epidermolysis-bullosa) OR (Primary-basilar-impression) OR (Primary-carnitine ADJ3 deficien*) OR (Primary-ciliary-dyskinesia) OR (Primary-Familial-Brain-Calcification) OR (Primary-hyperoxaluria-type-1) OR (Primary-hyperoxaluria-type-2) OR (Primary-hypomagnesemia ADJ3 secondary-hypocalcemia) OR (Primary-lateral-sclerosis) OR (Primary-open-angle-glaucoma-juvenile-onset-1) OR (Primary-pigmented-nodular-adrenocortical ADJ3 diseas*) OR (Primrose ADJ3 syndrom*) OR (Progeria) OR (Progeroid-short-stature ADJ3 pigmented-nevi) OR (Progeroid ADJ3 syndrom*-Petty) OR (Prognathism-mandibular) OR (Progressive-bifocal-chorioretinal-atrophy) OR (Progressive-deafness ADJ3 stapes-fixation) OR (Progressive-external-ophthalmoplegia ADJ3 autosomal-recessive-1) OR (Progressive-familial-heart-block-type-1A) OR (Progressive-familial-heart-block-type-1B) OR (Progressive-familial-heart-block-type-2) OR (Progressive-familial-intrahepatic-cholestasis-1) OR (Progressive-familial-intrahepatic-cholestasis-type-2) OR (Progressive-familial-intrahepatic-cholestasis-type-3) OR (Progressive-non-fluent-aphasia) OR (Progressive-osseous-heteroplasia) OR (Progressive-pseudorheumatoid-dysplasia) OR (Proopiomelanocortin ADJ3 deficien*) OR (Propionic-acidemia) OR (Proteus-like ADJ3 syndrom*) OR (Proud ADJ3 syndrom*) OR (Proximal-symphalangism) OR (Prune-belly ADJ3 syndrom*) OR (Pseudo-Pelger-Huet-anomaly) OR (Pseudoachondroplasia) OR (Pseudoaminopterin ADJ3 syndrom*) OR (Pseudocholinesterase ADJ3 deficien*) OR (Pseudodiastrophic-dysplasia) OR (Pseudohypoaldosteronism-type-2) OR (Pseudohypoparathyroidism-type-1A) OR (Pseudohypoparathyroidism-type-1B) OR (Pseudohypoparathyroidism-type-1C) OR (Pseudohypoparathyroidism-type-2) OR (Pseudoneonatal-adrenoleukodystroph*) OR (Pseudoprogeria ADJ3 syndrom*) OR (Pseudopseudohypoparathyroidism) OR (Pseudotrisomy-13 ADJ3 syndrom*) OR (Pseudoxanthoma-elasticum ADJ3 forme-fruste) OR (Ptosis-strabismus-ectopic-pupils) OR (Pulmonary-alveolar-microlithiasis) OR (Pulmonary-atresia ADJ3 ventricular-septal ADJ3 defect*) OR (Pulmonary-venoocclusive ADJ3 diseas*) OR (Punctate-palmoplantar-keratoderma-type-2) OR (Punctate-palmoplantar-keratoderma-type-I) OR (Purine-nucleoside-phosphorylase ADJ3 deficien*) OR (Pustulosis-palmaris-et-plantaris) OR (Pycnodysostosis) OR (Pyknoachondrogenesis) OR (Pyle ADJ3 diseas*) OR (Pyogenic-arthritis ADJ3 pyoderma-gangrenosum ADJ3 acne) OR (Pyramidal-molars-abnormal-upper-lip ADJ3 syndrom*) OR (Pyridoxal-5-phosphate-dependent-epilepsy) OR (Pyridoxine-dependent-epilepsy) OR (Pyruvate-dehydrogenase-phosphatase ADJ3 deficien*) OR (Qazi-Markouizos ADJ3 syndrom*) OR (Rabson-Mendenhall ADJ3 syndrom*) OR (Radial ADJ3 defect*-Robin-sequence) OR (Radial-ray-agenesis) OR (Radial-ray-hypoplasia-choanal-atresia) OR (Radio-renal ADJ3 syndrom*) OR (Radioulnar-synostosis-microcephaly-scoliosis ADJ3 syndrom*) OR (Radius-absent-anogenital-anomalies) OR (Raine ADJ3 syndrom*) OR (Ramon ADJ3 syndrom*) OR (Ramos-Arroyo-Clark ADJ3 syndrom*) OR (Rapadilino ADJ3 syndrom*) OR (Rapid-onset-dystonia-parkinsonism) OR (Rasmussen-Johnsen-Thomsen ADJ3 syndrom*) OR (Reardon-Wilson-Cavanagh ADJ3 syndrom*) OR (Recessive-dystrophic-epidermolysis-bullosa-generalized-other) OR (Reducing-body-myopathy) OR (Refsum ADJ3 diseas*) OR (Refsum ADJ3 diseas* ADJ3 infantile-form) OR (Renal-agenesis) OR (Renal-coloboma ADJ3 syndrom*) OR (Renal-dysplasia ADJ3 retinal-pigmentary-dystroph* ADJ3 cerebellar-ataxia ADJ3 skeletal-dysplasia) OR (Renal-dysplasia-limb ADJ3 defect*s ADJ3 syndrom*) OR (Renal-glycosuria) OR (Renal-hypomagnesemia-2) OR (Renal-hypomagnesemia-6) OR (Renal-hypouricemia) OR (Renal-tubular-acidosis ADJ3 deafness) OR (Renal-tubulopathy ADJ3 diabetes-mellitus ADJ3 -cerebellar-ataxia-due-to ADJ3 duplicat* ADJ3 mitochondrial-DNA) OR (Renier-Gabreels-Jasper ADJ3 syndrom*) OR (Renpenning ADJ3 syndrom*) OR (Reticular-dysgenesis) OR (Retinal-arterial-macroaneurysm ADJ3 supravalvular-pulmonic-stenosis) OR (Retinal-cone-dystroph*-1) OR (Retinal-degeneration ADJ3 nanophthalmos ADJ3 cystic-macular-degeneration ADJ3 -angle-closure-glaucoma) OR (Retinal-vasculopathy ADJ3 cerebral-leukodystroph* ADJ3 systemic-manifestations) OR (Retinitis-pigmentosa) OR (Retinitis-pigmentosa-intellectual-disability-deafness-hypogonadism ADJ3 syndrom*) OR (Rett ADJ3 syndrom*) OR (RFT1-CDG-) OR (CDG-In) OR (Rhizomelic-chondrodysplasia-punctata-type-1) OR (Rhizomelic-chondrodysplasia-punctata-type-2) OR (Rhizomelic-dysplasia-Patterson-Lowry) OR (Rhizomelic ADJ3 syndrom*) OR (RHYNS ADJ3 syndrom*) OR (Riboflavin-transporter ADJ3 deficien*) OR (Richards-Rundle ADJ3 syndrom*) OR (Richieri-Costa-Da-Silva ADJ3 syndrom*) OR (Richieri-Costa-Pereira ADJ3 syndrom*) OR (Right-ventricle-hypoplasia) OR (Rigid-spine ADJ3 syndrom*) OR (Rippling-muscle ADJ3 diseas*) OR (RNAse-T2-deficient-leukoencephalopathy) OR (Roberts ADJ3 syndrom*) OR (Robinow ADJ3 syndrom*) OR (Roch-Leri-mesosomatous-lipomatosis) OR (Rodrigues-blindness) OR (Roifman ADJ3 syndrom*) OR (Rokitansky-sequence) OR (Rokitansky-Aschoff-sinuses ADJ3 gallbladder) OR (Rombo ADJ3 syndrom*) OR (Rommen-Mueller-Sybert ADJ3 syndrom*) OR (Rothmund-Thomson ADJ3 syndrom*) OR (Roussy-Levy ADJ3 syndrom*) OR (Rozin-Hertz-Goodman ADJ3 syndrom*) OR (RRM2B-related-mitochondrial-DNA-depletion ADJ3 syndrom*) OR (Rud ADJ3 syndrom*) OR (Russell-Silver ADJ3 syndrom*) OR (Rutherfurd ADJ3 syndrom*) OR (Ruvalcaba ADJ3 syndrom*) OR (Ruzicka-Goerz-Anton ADJ3 syndrom*) OR (Sabinas-brittle-hair ADJ3 syndrom*) OR (Saccharopinuria) OR (Sacral-hemangiomas-multiple-congenital-abnormalities) OR (Sacral-meningocele-conotruncal-heart ADJ3 defect*s) OR (Saethre-Chotzen ADJ3 syndrom*) OR (Saito-Kuba-Tsuruta ADJ3 syndrom*) OR (Sakoda-complex) OR (Salcedo ADJ3 syndrom*) OR (Salla ADJ3 diseas*) OR (Sarcosinemia) OR (Satoyoshi ADJ3 syndrom*) OR (Saul-Wilkes-Stevenson ADJ3 syndrom*) OR (Say-Barber-Miller ADJ3 syndrom*) OR (Say-Meyer ADJ3 syndrom*) OR (Say ADJ3 syndrom*) OR (Say-Field-Coldwell ADJ3 syndrom*) OR (Scalp ADJ3 defect*s-postaxial-polydactyly) OR (Scalp-ear-nipple ADJ3 syndrom*) OR (Scapuloperoneal ADJ3 syndrom* ADJ3 neurogenic ADJ3 Kaeser) OR (SCARF ADJ3 syndrom*) OR (Schaaf-Yang ADJ3 syndrom*) OR (Schaap-Taylor-Baraitser ADJ3 syndrom*) OR (Schaefer-Stein-Oshman ADJ3 syndrom*) OR (Scheie ADJ3 syndrom*) OR (Scheuermann ADJ3 diseas*) OR (Schimke-immunoosseous-dysplasia) OR (Schindler ADJ3 diseas*-type-1) OR (Schinzel-Giedion ADJ3 syndrom*) OR (Schinzel-type-phocomelia) OR (Schisis-association) OR (Schizencephaly) OR (Schneckenbecken-dysplasia) OR (Scholte ADJ3 syndrom*) OR (Schrander-Stumpel-Theunissen-Hulsmans ADJ3 syndrom*) OR (Schwannomatosis) OR (Schwartz-Jampel ADJ3 syndrom*) OR (Sclerosteosis) OR (SCOT ADJ3 deficien*) OR (Scott-Bryant-Graham ADJ3 syndrom*) OR (Sea-Blue-histiocytosis) OR (Seaver-Cassidy ADJ3 syndrom*) OR (Sebaceous-gland-hyperplasia ADJ3 familial-presenile) OR (Seckel-like ADJ3 syndrom*-Majoor-Krakauer) OR (Seckel ADJ3 syndrom*) OR (Segmentation ADJ3 syndrom*-1) OR (Selective-IgM ADJ3 deficien*) OR (Semantic-dementia) OR (Sengers ADJ3 syndrom*) OR (Senior-Loken ADJ3 syndrom*) OR (Sensory-ataxic-neuropathy ADJ3 dysarthria ADJ3 -ophthalmoparesis) OR (Sepiapterin-reductase ADJ3 deficien*) OR (Septo-optic-dysplasia-spectrum) OR (Seres-Santamaria-Arimany-Muniz ADJ3 syndrom*) OR (SERKAL ADJ3 syndrom*) OR (SeSAME ADJ3 syndrom*) OR (SETBP1-disorder) OR (Severe-achondroplasia ADJ3 developmental-delay ADJ3 acanthosis-nigricans) OR (Severe-combined-immunodeficiency) OR (Severe-combined-immunodeficiency-due-to-complete-RAG ADJ3 deficien*) OR (Severe-congenital-nemaline-myopathy) OR (Severe-congenital-neutropenia-X-linked) OR (Severe-generalized-recessive-dystrophic-epidermolysis-bullosa) OR (Severe-intellectual-disability-progressive-spastic-diplegia ADJ3 syndrom*) OR (Severe-X-linked-intellectual-disability ADJ3 Gustavson) OR (Sheldon-Hall ADJ3 syndrom*) OR (Short-rib-polydactyly ADJ3 syndrom*-type-3) OR (Short-rib-polydactyly ADJ3 syndrom*-type-1) OR (Short-rib-polydactyly ADJ3 syndrom*-type-4) OR (Short-rib-polydactyly ADJ3 syndrom* ADJ3 Majewski) OR (Short-stature-deafness-neutrophil-dysfunction) OR (Short-stature ADJ3 syndrom* ADJ3 Brussels) OR (Short-stature-wormian-bones-dextrocardia) OR (Short-stature-craniofacial-anomalies-genital-hypoplasia ADJ3 syndrom*) OR (SHORT ADJ3 syndrom*) OR (Short-chain-acyl-CoA-dehydrogenase ADJ3 deficien*) OR (Short-limb-skeletal-dysplasia ADJ3 severe-combined-immunodeficiency) OR (Shprintzen-omphalocele ADJ3 syndrom*) OR (Shprintzen-Goldberg-craniosynostosis ADJ3 syndrom*) OR (Sialidosis-type-I) OR (Sialidosis ADJ3 type-II) OR (Sialuria ADJ3 French) OR (Siegler-Brewer-Carey ADJ3 syndrom*) OR (Silengo-Lerone-Pelizza ADJ3 syndrom*) OR (Sillence ADJ3 syndrom*) OR (Simosa-cranio-facial ADJ3 syndrom*) OR (Simpson-Golabi-Behmel ADJ3 syndrom*) OR (Single-upper-central-incisor) OR (Singleton-Merten ADJ3 syndrom*) OR (Sirenomelia) OR (Sitosterolemia) OR (Situs-inversus) OR (Situs-inversus-totalis ADJ3 cystic-dysplasia ADJ3 kidneys ADJ3 pancreas) OR (Sjogren-Larsson ADJ3 syndrom*) OR (Skeletal-dysplasia ADJ3 San-Diego) OR (Skin-fragility-woolly-hair-palmoplantar-keratoderma ADJ3 syndrom*) OR (SLC35A1-CDG-) OR (CDG-IIf) OR (SLC35A2-CDG) OR (SLC35C1-CDG-) OR (CDG-IIc) OR (Small-patella ADJ3 syndrom*) OR (Smith-McCort-dysplasia) OR (Smith-Fineman-Myers ADJ3 syndrom*) OR (Smith-Lemli-Opitz ADJ3 syndrom*) OR (Snowflake-vitreoretinal-degeneration) OR (Snyder-Robinson ADJ3 syndrom*) OR (Sonoda ADJ3 syndrom*) OR (Sotos ADJ3 syndrom*) OR (Spasmodic-dysphonia) OR (Spastic-ataxia-Charlevoix-Saguenay) OR (Spastic-paraplegia-1) OR (Spastic-paraplegia-10) OR (Spastic-paraplegia-11) OR (Spastic-paraplegia-12) OR (Spastic-paraplegia-13) OR (Spastic-paraplegia-14) OR (Spastic-paraplegia-15) OR (Spastic-paraplegia-16) OR (Spastic-paraplegia-17) OR (Spastic-paraplegia-18) OR (Spastic-paraplegia-19) OR (Spastic-paraplegia-2) OR (Spastic-paraplegia-23) OR (Spastic-paraplegia-24) OR (Spastic-paraplegia-25) OR (Spastic-paraplegia-26) OR (Spastic-paraplegia-29) OR (Spastic-paraplegia-3) OR (Spastic-paraplegia-31) OR (Spastic-paraplegia-32) OR (Spastic-paraplegia-4) OR (Spastic-paraplegia-51) OR (Spastic-paraplegia-5A) OR (Spastic-paraplegia-6) OR (Spastic-paraplegia-7) OR (Spastic-paraplegia-8) OR (Spastic-paraplegia-9) OR (Spastic-paraplegia-facial-cutaneous-lesions) OR (Spastic-paraplegia-epilepsy-intellectual-disability ADJ3 syndrom*) OR (Spastic-paraplegia-glaucoma-intellectual-disability ADJ3 syndrom*) OR (Spastic-tetraplegia-retinitis-pigmentosa-intellectual-disability ADJ3 syndrom*) OR (Spastic-tetraplegia-thin-corpus-callosum-progressive-postnatal-microcephaly ADJ3 syndrom*) OR (Specific-antibody ADJ3 deficien*) OR (Spina-bifida) OR (Spinal-atrophy-ophthalmoplegia-pyramidal ADJ3 syndrom*) OR (Spinal-muscular-atrophy-1) OR (Spinal-muscular-atrophy-type-2) OR (Spinal-muscular-atrophy-type-3) OR (Spinal-muscular-atrophy-progressive-myoclonic-epilepsy ADJ3 syndrom*) OR (Spinocerebellar-ataxia-1) OR (Spinocerebellar-ataxia-10) OR (Spinocerebellar-ataxia-11) OR (Spinocerebellar-ataxia-12) OR (Spinocerebellar-ataxia-13) OR (Spinocerebellar-ataxia-14) OR (Spinocerebellar-ataxia-15) OR (Spinocerebellar-ataxia-17) OR (Spinocerebellar-ataxia-18) OR (Spinocerebellar-ataxia-19 ADJ3 22) OR (Spinocerebellar-ataxia-2) OR (Spinocerebellar-ataxia-20) OR (Spinocerebellar-ataxia-21) OR (Spinocerebellar-ataxia-23) OR (Spinocerebellar-ataxia-25) OR (Spinocerebellar-ataxia-26) OR (Spinocerebellar-ataxia-27) OR (Spinocerebellar-ataxia-28) OR (Spinocerebellar-ataxia-29) OR (Spinocerebellar-ataxia-31) OR (Spinocerebellar-ataxia-34) OR (Spinocerebellar-ataxia-4) OR (Spinocerebellar-ataxia-5) OR (Spinocerebellar-ataxia-7) OR (Spinocerebellar-ataxia-8) OR (Spinocerebellar-ataxia-autosomal-recessive-3) OR (Spinocerebellar-ataxia-autosomal-recessive-4) OR (Spinocerebellar-ataxia-autosomal-recessive-5) OR (Spinocerebellar-ataxia-autosomal-recessive-7) OR (Spinocerebellar-ataxia-autosomal-recessive-8) OR (Spinocerebellar-ataxia-type-6) OR (Spinocerebellar-ataxia ADJ3 axonal-neuropathy-type-1) OR (Spinocerebellar-ataxia ADJ3 dysmorphism) OR (Spinocerebellar-ataxia-X-linked-type-3) OR (Spinocerebellar-ataxia-X-linked-type-4) OR (Spinocerebellar-degeneration ADJ3 corneal-dystroph*) OR (Splenogonadal-fusion-limb ADJ3 defect*s-micrognatia) OR (Split-hand-foot-malformation) OR (Split-hand-split-foot-nystagmus) OR (Split-hand-urinary-anomalies-spina-bifida) OR (Split-spinal-cord-malformation) OR (Spondylocamptodactyly) OR (Spondylocarpotarsal-synostosis ADJ3 syndrom*) OR (Spondylocostal-dysostosis-1) OR (Spondylocostal-dysostosis-2) OR (Spondylocostal-dysostosis-3) OR (Spondylocostal-dysostosis-4) OR (Spondylocostal-dysostosis-5) OR (Spondylocostal-dysostosis-6) OR (Spondylodysplastic-Ehlers-Danlos ADJ3 syndrom*) OR (Spondyloenchondrodysplasia ADJ3 immune-dysregulation) OR (Spondyloepimetaphyseal-dysplasia-Genevieve) OR (Spondyloepimetaphyseal-dysplasia-joint-laxity) OR (Spondyloepimetaphyseal-dysplasia-Matrilin-3-related) OR (Spondyloepimetaphyseal-dysplasia-micromelic) OR (Spondyloepimetaphyseal-dysplasia-Missouri) OR (Spondyloepimetaphyseal-dysplasia-Shohat) OR (Spondyloepimetaphyseal-dysplasia-Sponastrime) OR (Spondyloepimetaphyseal-dysplasia-Strudwick) OR (Spondyloepimetaphyseal-dysplasia ADJ3 hypotrichosis) OR (Spondyloepimetaphyseal-dysplasia ADJ3 multiple-dislocations) OR (Spondyloepimetaphyseal-dysplasia-X-linked) OR (Spondyloepimetaphyseal-dysplasia ADJ3 Aggrecan) OR (Spondyloepiphyseal-dysplasia-congenita) OR (Spondyloepiphyseal-dysplasia-Maroteaux) OR (Spondyloepiphyseal-dysplasia-tarda-X-linked) OR (Spondyloepiphyseal-dysplasia-brachydactyly ADJ3 distinctive-speech) OR (Spondylometaepiphyseal-dysplasia-short-limb-hand) OR (Spondylometaphyseal-dysplasia-Algerian) OR (Spondylometaphyseal-dysplasia-corner-fracture) OR (Spondylometaphyseal-dysplasia-East-African) OR (Spondylometaphyseal-dysplasia-Sedaghatian) OR (Spondylometaphyseal-dysplasia-type-A4) OR (Spondylometaphyseal-dysplasia ADJ3 bowed-forearms ADJ3 facial-dysmorphism) OR (Spondylometaphyseal-dysplasia ADJ3 cone-rod-dystroph*) OR (Spondylometaphyseal-dysplasia ADJ3 dentinogenesis-imperfecta) OR (Spondylometaphyseal-dysplasia-X-linked) OR (Spondylometaphyseal-dysplasia ADJ3 Kozlowski) OR (Spondyloperipheral-dysplasia) OR (Spondylospinal-thoracic-dysostosis) OR (Spondylothoracic-dysostosis) OR (Sprengel-deformity) OR (SRD5A3-CDG-) OR (CDG-Iq) OR (SSR4-CDG) OR (STAC3-Disorder) OR (Stalker-Chitayat ADJ3 syndrom*) OR (STAR ADJ3 syndrom*) OR (Stargardt ADJ3 diseas*) OR (Steatocystoma-multiplex) OR (Steatocystoma-multiplex ADJ3 natal-teeth) OR (Steinfeld ADJ3 syndrom*) OR (Sternal-cleft) OR (Stickler ADJ3 syndrom*) OR (Stiff-person ADJ3 syndrom*) OR (Stiff-skin ADJ3 syndrom*) OR (Stocco-dos-Santos ADJ3 syndrom*) OR (Stoll-Alembik-Finck ADJ3 syndrom*) OR (Striatonigral-degeneration-infantile) OR (Stuve-Wiedemann ADJ3 syndrom*) OR (Subaortic-stenosis-short-stature ADJ3 syndrom*) OR (Subcortical-band-heterotopia) OR (Succinic-semialdehyde-dehydrogenase ADJ3 deficien*) OR (Sudden-infant-death ADJ3 dysgenesis ADJ3 testes ADJ3 syndrom*) OR (Sugarman-brachydactyly) OR (Symphalangism ADJ3 multiple-anomalies ADJ3 hands ADJ3 feet) OR (Syndactyly-Cenani-Lenz) OR (Syndactyly-type-1) OR (Syndactyly-type-3) OR (Syndactyly-type-5) OR (Syndactyly-type-9) OR (Syndactyly-polydactyly-earlobe ADJ3 syndrom*) OR (Syndromic-microphthalmia ADJ3 type-3) OR (Syndromic-X-linked-intellectual-disability-7) OR (Syngnathia-cleft-palate) OR (Syngnathia-multiple-anomalies) OR (Synovial-chondromatosis ADJ3 familial ADJ3 dwarfism) OR (Syringomyelia) OR (T-cell-immunodeficiency ADJ3 congenital-alopecia ADJ3 nail-dystroph*) OR (Tabatznik ADJ3 syndrom*) OR (Talo-patello-scaphoid-osteolysis ADJ3 synovitis ADJ3 -short-fourth-metacarpals) OR (Talonavicular-coalition) OR (Tangier ADJ3 diseas*) OR (TANGO2-Related-Metabolic-Encephalopathy ADJ3 Arrhythmias) OR (TARP ADJ3 syndrom*) OR (Tarsal-carpal-coalition ADJ3 syndrom*) OR (Taurodontia ADJ3 absent-teeth ADJ3 sparse-hair ADJ3 syndrom*) OR (Taurodontism) OR (Taurodontism ADJ3 microdontia ADJ3 -dens-invaginatus) OR (Teebi-Shaltout ADJ3 syndrom*) OR (Tel-Hashomer-camptodactyly ADJ3 syndrom*) OR (Telfer-Sugar-Jaeger ADJ3 syndrom*) OR (Temple ADJ3 syndrom*) OR (Temple-Baraitser ADJ3 syndrom*) OR (Temporal-epilepsy ADJ3 familial) OR (Temtamy-preaxial-brachydactyly ADJ3 syndrom*) OR (Temtamy ADJ3 syndrom*) OR (Tendons ADJ3 extensor ADJ3 fingers ADJ3 anomalous-insertion-of) OR (Testotoxicosis) OR (Tethered-cord ADJ3 syndrom*) OR (Tetra-amelia ADJ3 syndrom*) OR (Tetraamelia ADJ3 pulmonary-hypoplasia) OR (Tetraamelia-multiple-malformations ADJ3 syndrom*) OR (Tetralogy ADJ3 Fallot) OR (Tetralogy ADJ3 fallot ADJ3 glaucoma) OR (Tetramelic-monodactyly) OR (Tetraploidy) OR (Tetrasomy-21) OR (Thai-symphalangism ADJ3 syndrom*) OR (Thakker-Donnai ADJ3 syndrom*) OR (Thanatophoric-dysplasia-type-1) OR (Thanatophoric-dysplasia-type-2) OR (Thiopurine-S-methyltranferase ADJ3 deficien*) OR (Thomas ADJ3 syndrom*) OR (Thoracic-dysplasia-hydrocephalus ADJ3 syndrom*) OR (Thoraco-abdominal-enteric ADJ3 duplicat*) OR (Thumb-deformity) OR (Thumb-deformity ADJ3 alopecia ADJ3 pigmentation-anomaly) OR (Thumb-stiffness-brachydactyly-intellectual-disability ADJ3 syndrom*) OR (Thymic-Renal-Anal-Lung-dysplasia) OR (Thyroid-dysgenesis) OR (Tibia-absent-polydactyly-arachnoid-cyst) OR (Tietz ADJ3 syndrom*) OR (Tight-skin-contracture ADJ3 syndrom* ADJ3 lethal) OR (Timothy ADJ3 syndrom*) OR (TMEM165-CDG-) OR (CDG-IIk) OR (Tollner-Horst-Manzke ADJ3 syndrom*) OR (Tolosa-Hunt ADJ3 syndrom*) OR (Tonoki ADJ3 syndrom*) OR (Toriello-Carey ADJ3 syndrom*) OR (Torticollis-keloids-cryptorchidism-renal-dysplasia) OR (Total-Hypotrichosis ADJ3 Mari) OR (Townes-Brocks ADJ3 syndrom*) OR (Tracheal-agenesis) OR (Tranebjaerg-Svejgaard ADJ3 syndrom*) OR (Transaldolase ADJ3 deficien*) OR (Transient-bullous-dermolysis ADJ3 newborn) OR (Transient-infantile-liver-failure) OR (Transient-neonatal-diabetes-mellitus) OR (Treacher-Collins ADJ3 syndrom*) OR (Treacher-Collins ADJ3 syndrom*-3) OR (Tricho-dento-osseous ADJ3 syndrom*) OR (Trichodental ADJ3 syndrom*) OR (Trichohepatoenteric ADJ3 syndrom*) OR (Trichomegaly ADJ3 intellectual-disability ADJ3 dwarfism ADJ3 pigmentary-degeneration ADJ3 retina) OR (Trichorhinophalangeal ADJ3 syndrom*-type-1) OR (Trichorhinophalangeal ADJ3 syndrom*-type-2) OR (Trichorhinophalangeal ADJ3 syndrom*-type-3) OR (Trichothiodystroph*) OR (Tricuspid-atresia) OR (Trigonobrachycephaly ADJ3 bulbous-bifid-nose ADJ3 micrognathia ADJ3 -abnormalities ADJ3 hands ADJ3 feet) OR (Trigonocephaly-bifid-nose-acral-anomalies) OR (Triphalangeal-thumbs-brachyectrodactyly) OR (Triple-A ADJ3 syndrom*) OR (Trismus-pseudocamptodactyly ADJ3 syndrom*) OR (Trisomy-18) OR (Trisomy-3-mosaicism) OR (Troyer ADJ3 syndrom*) OR (Tryptophanuria ADJ3 dwarfism) OR (Tubular-aggregate-myopathy) OR (Tucker ADJ3 syndrom*) OR (Tufting-enteropathy) OR (Tukel ADJ3 syndrom*) OR (Tumor-necrosis-factor-receptor-associated-periodic ADJ3 syndrom*) OR (Twenty-nail-dystroph*) OR (Tylosis ADJ3 esophageal-cancer) OR (Typical-congenital-nemaline-myopathy) OR (Tyrosine-hydroxylase ADJ3 deficien*) OR (Tyrosine-oxidase-temporary ADJ3 deficien*) OR (Tyrosinemia-type-1) OR (Tyrosinemia-type-2) OR (Tyrosinemia-type-3) OR (Ulerythema-ophryogenesis) OR (Ullrich-congenital-muscular-dystroph*) OR (Ulna ADJ3 fibula ADJ3 hypoplasia-of) OR (Ulna-hypoplasia-intellectual-disability ADJ3 syndrom*) OR (Ulna-metaphyseal-dysplasia ADJ3 syndrom*) OR (Ulnar-hypoplasia-lobster-claw-deformity ADJ3 feet) OR (Ulnar-mammary ADJ3 syndrom*) OR (Uncombable-hair ADJ3 syndrom*) OR (Unverricht-Lundborg ADJ3 diseas*) OR (Upington ADJ3 diseas*) OR (Urachal-cyst) OR (Urogenital-adysplasia) OR (Usher ADJ3 syndrom*-type-2A) OR (Usher ADJ3 syndrom* ADJ3 type-1) OR (UV-sensitive ADJ3 syndrom*) OR (VACTERL-association) OR (VACTERL-association ADJ3 hydrocephaly ADJ3 X-linked) OR (VACTERL-hydrocephaly) OR (Vagina ADJ3 absence-of) OR (Vagneur-Triolle-Ripert ADJ3 syndrom*) OR (Van-Benthem-Driessen-Hanveld ADJ3 syndrom*) OR (Van-Buchem ADJ3 diseas*-type-2) OR (Van-Den-Bosch ADJ3 syndrom*) OR (Van-den-Ende-Gupta ADJ3 syndrom*) OR (Van-der-Woude ADJ3 syndrom*) OR (Van-der-Woude ADJ3 syndrom*-2) OR (Variegate-porphyria) OR (Vascular-hyalinosis) OR (Ventricular-extrasystoles ADJ3 syncopal-episodes---perodactyly---Robin-sequence) OR (Verloes-Bourguignon ADJ3 syndrom*) OR (Verloes-Van-Maldergem-Marneffe ADJ3 syndrom*) OR (Verloove-Vanhorick-Brubakk ADJ3 syndrom*) OR (Vibratory-urticaria) OR (Vici ADJ3 syndrom*) OR (Viljoen-Kallis-Voges ADJ3 syndrom*) OR (VLCAD ADJ3 deficien*) OR (Vocal-cord-dysfunction-familial) OR (Vohwinkel ADJ3 syndrom*) OR (Waardenburg ADJ3 syndrom*-type-1) OR (Waardenburg ADJ3 syndrom*-type-2) OR (Waardenburg ADJ3 syndrom*-type-3) OR (Waardenburg ADJ3 syndrom*-type-4) OR (Wagner ADJ3 syndrom*) OR (WAGR ADJ3 syndrom*) OR (Walker-Warburg ADJ3 syndrom*) OR (Warfarin ADJ3 syndrom*) OR (Warman-Mulliken-Hayward ADJ3 syndrom*) OR (Weaver ADJ3 syndrom*) OR (Weill-Marchesani ADJ3 syndrom*) OR (Weissenbacher-Zweymuller ADJ3 syndrom*) OR (Welander-distal-myopathy ADJ3 Swedish) OR (Wells-Jankovic ADJ3 syndrom*) OR (Werner ADJ3 syndrom*) OR (West ADJ3 syndrom*) OR (Weyers-acrofacial-dysostosis) OR (Weyers-ulnar-ray) OR (oligodactyly ADJ3 syndrom*) OR (WHIM ADJ3 syndrom*) OR (Whistling-face ADJ3 syndrom* ADJ3 recessive-form) OR (White-forelock ADJ3 malformations) OR (White-matter-hypoplasia-corpus-callosum-agenesis-intellectual-disability ADJ3 syndrom*) OR (White-sponge-nevus ADJ3 cannon) OR (Wiedemann-Oldigs-Oppermann ADJ3 syndrom*) OR (Wildervanck ADJ3 syndrom*) OR (Wilms ADJ3 tumor) OR (Wilson ADJ3 diseas*) OR (Wilson-Turner ADJ3 syndrom*) OR (Winchester ADJ3 syndrom*) OR (Witkop ADJ3 syndrom*) OR (Wolfram ADJ3 syndrom*) OR (Wolman ADJ3 diseas*) OR (Woodhouse-Sakati ADJ3 syndrom*) OR (Woods-Black-Norbury ADJ3 syndrom*) OR (Woolly-hair-hypotrichosis-everted-lower-lip ADJ3 outstanding-ears) OR (Woolly-hair ADJ3 syndrom*) OR (Worth-type-autosomal-dominant-osteosclerosis) OR (Wrinkly-skin ADJ3 syndrom*) OR (X-linked-adrenal-hypoplasia-congenita) OR (X-linked-agammaglobulinemia) OR (X-linked-cardiac-valvular-dysplasia) OR (X-linked-cerebral-adrenoleukodystroph*) OR (X-linked-Charcot-Marie-Tooth ADJ3 diseas*-type-1) OR (X-linked-Charcot-Marie-Tooth ADJ3 diseas*-type-2) OR (X-linked-Charcot-Marie-Tooth ADJ3 diseas*-type-3) OR (X-linked-Charcot-Marie-Tooth ADJ3 diseas*-type-4) OR (X-linked-Charcot-Marie-Tooth ADJ3 diseas*-type-5) OR (X-linked-Charcot-Marie-Tooth ADJ3 diseas*-type-6) OR (X-linked-complicated-corpus-callosum-agenesis) OR (X-linked-complicated-spastic-paraplegia-type-1) OR (X-linked-congenital-generalized-hypertrichosis) OR (X-linked-congenital-stationary-night-blindness) OR (X-linked-creatine ADJ3 deficien*) OR (X-linked-dominant-chondrodysplasia-punctata-2) OR (X-linked-dystonia-parkinsonism) OR (X-linked-hereditary-sensory ADJ3 autonomic-neuropathy ADJ3 deafness) OR (X-linked-hypohidrotic-ectodermal-dysplasia) OR (X-linked-hypophosphatemia) OR (X-linked-ichthyosis) OR (X-linked-intellectual-disability---corpus-callosum-agenesis---spastic-quadriparesis) OR (X-linked-intellectual-disability---short-stature---obesity) OR (X-linked-intellectual-disability ADJ3 Abidi) OR (X-linked-intellectual-disability ADJ3 Najm) OR (X-linked-intellectual-disability ADJ3 Schimke) OR (X-linked-intellectual-disability ADJ3 Siderius) OR (X-linked-intellectual-disability ADJ3 Turner) OR (X-linked-intellectual-disability-dysmorphism-cerebral-atrophy ADJ3 syndrom*) OR (X-linked-intellectual-disability-plagiocephaly ADJ3 syndrom*) OR (X-linked-lissencephaly ADJ3 abnormal-genitalia) OR (X-linked-lymphoproliferative ADJ3 diseas*-due-to-SH2D1A ADJ3 deficien*) OR (X-linked-lymphoproliferative ADJ3 syndrom*) OR (X-linked-myopathy ADJ3 excessive-autophagy) OR (X-linked-myotubular-myopathy) OR (X-linked-non-specific-intellectual-disability) OR (X-linked-periventricular-heterotopia) OR (X-linked-severe-combined-immunodeficiency) OR (X-linked-skeletal-dysplasia-intellectual-disability ADJ3 syndrom*) OR (Xanthinuria-type-1) OR (Xanthinuria-type-2) OR (Xeroderma-pigmentosum) OR (XFE-progeroid ADJ3 syndrom*) OR (Xia-Gibbs ADJ3 syndrom*) OR (XK-aprosencephaly) OR (Xp22#3 ADJ3 microdeletion* ADJ3 syndrom*) OR (Yemenite-deaf-blind-hypopigmentation ADJ3 syndrom*) OR (Yorifuji-Okuno ADJ3 syndrom*) OR (Young ADJ3 syndrom*) OR (Yunis-Varon ADJ3 syndrom*) OR (Zadik-Barak-Levin ADJ3 syndrom*) OR (ZAP-70 ADJ3 deficien*) OR (Zazam-Sheriff-Phillips ADJ3 syndrom*) OR (Zechi-Ceide ADJ3 syndrom*) OR (Zellweger ADJ3 syndrom*) OR (Zlotogora ADJ3 syndrom*) OR (Zori-Stalker-Williams ADJ3 syndrom*) OR (ZTTK ADJ3 syndrom*) OR (Zunich-neuroectodermal ADJ3 syndrom*) OR (Amyopathic-dermatomyositis) OR (Bizarre-parosteal-osteochondromatous-proliferation) OR (Classical-Ehlers-Danlos-syndrom*) OR (Ehlers-Danlos-syndrom*) OR (Eosinophilic-fasciitis) OR (Mixed-connective-tissue-diseas*) OR (Osteosclerosis ADJ3 ichthyosis ADJ3 premature-ovarian-failure) OR (Paget-disease ADJ3 bone ADJ3 familial) OR (Polymyositis) OR (Rheumatoid-factor-negative-juvenile-idiopathic-arthritis) OR (Ribbing-diseas*) OR (Temporomandibular-ankylosis) OR (Benign-recurrent-intrahepatic-cholestasis-1) OR (Benign-recurrent-intrahepatic-cholestasis-2) OR (Boerhaave-syndrom*) OR (Budd-Chiari-syndrom*) OR (Childhood-hepatocellular-carcinoma) OR (Chronic-hiccups) OR (Collagenous-gastritis) OR (Congenital ADJ3 disorder* ADJ3 glycosylation) OR (Cutaneous-photosensitivity ADJ3 colitis ADJ3 lethal) OR (Desmoplastic-small-round-cell-tumor) OR (Disseminated-peritoneal-leiomyomatosis) OR (Duodenal-ulcer-due-to-antral-G-cell-hyperfunction) OR (Eosinophilic-gastroenteritis) OR (Galactose-epimerase-deficiency) OR (Goblet-cell-carcinoid) OR (Hepatic-encephalopathy) OR (Hepatic-veno-occlusive-diseas*) OR (Hepatoblastoma) OR (Idiopathic-achalasia) OR (Intrahepatic-cholestasis ADJ3 pregnancy) OR (Klatskin-tumor) OR (Malakoplakia) OR (Mallory-Weiss-syndrom*) OR (Menetrier-diseas*) OR (Necrotizing-enterocolitis) OR (Nodular-regenerative-hyperplasia) OR (Pancreatic-adenoma) OR (Pediatric-Crohn ADJ3 diseas*) OR (Pediatric-ulcerative-colitis) OR (Primary-biliary-cholangitis) OR (Primary-sclerosing-cholangitis) OR (Progressive-familial-intrahepatic-cholestasis-4) OR (Pseudomyxoma-peritonei) OR (Renal-nutcracker-syndrom*) OR (Retroperitoneal-fibrosis) OR (Sandifer-syndrom*) OR (Sclerosing-mesenteritis) OR (Small-Intestinal-Adenocarcinoma) OR (Superior-mesenteric-artery-syndrom*) OR (VIPoma) OR (Watermelon-stomach) OR (Whipple-diseas*) OR (Zollinger-Ellison-syndrom*) OR (Auditory-neuropathy-spectrum-disorder*) OR (Cholesteatoma) OR (Fetal-indomethacin-syndrom*) OR (Fetal-minoxidil-syndrom*) OR (IgG4-related-dacryoadenitis ADJ3 sialadenitis) OR (Mal-de-debarquement-syndrom*) OR (Recurrent-respiratory-papillomatosis) OR (Vestibulocochlear-dysfunction ADJ3 progressive) OR (Acquired-generalized-lipodystrophy) OR (Acromegaly) OR (ACTH-secreting-pituitary-adenoma) OR (Ahumada-Del-Castillo-syndrom*) OR (Autoimmune-polyglandular-syndrome-type-3) OR (Central-nervous-system-germinoma) OR (Cushing ADJ3 syndrom*) OR (Diencephalic-syndrom*) OR (Familial-chylomicronemia-syndrom*) OR (Gigantism) OR (Granulomatous-hypophysitis) OR (Growth-hormone-deficiency) OR (Holoprosencephaly) OR (Hyperadrenalism) OR (Hypoparathyroidism) OR (Hypopituitarism) OR (Iodine-antenatal-exposure) OR (Langerhans-cell-histiocytosis) OR (Meningioma) OR (Merkel-cell-carcinoma) OR (Optic-pathway-glioma) OR (Papillary-thyroid-carcinoma) OR (Parathyroid-carcinoma) OR (Primary-hyperparathyroidism) OR (Progressive-encephalomyelitis ADJ3 rigidity ADJ3 myoclonus) OR (Prolactinoma) OR (Pseudohypoparathyroidism) OR (Sheehan-syndrom*) OR (Small-cell-carcinoma ADJ3 bladder) OR (Thyrotropin-deficiency ADJ3 isolated) OR (Waterhouse-Friderichsen-syndrom*) OR (Achromatopsia-2) OR (Achromatopsia-3) OR (Aland-island-eye-diseas*) OR (Aniridia) OR (Anterior-ischemic-optic-neuropathy) OR (Anterior-uveitis) OR (Autosomal-recessive-bestrophinopathy) OR (Birdshot-chorioretinopathy) OR (Brown-syndrom*) OR (Cataract-Hutterite-type) OR (Cataract ADJ3 posterior-polar ADJ3 1) OR (Cataract ADJ3 posterior-polar ADJ3 3) OR (Cataract ADJ3 posterior-polar ADJ3 4) OR (Cataract ADJ3 posterior-polar ADJ3 5) OR (Centronuclear-myopathy) OR (Chandler ADJ3 syndrom*) OR (Charles-Bonnet-syndrom*) OR (Chorioretinitis) OR (Cone-dystrophy-X-linked ADJ3 tapetal-like-sheen) OR (Cone-rod-dystrophy-1) OR (Cone-rod-dystrophy-2) OR (Congenital-cystic-eye) OR (Corneal-dystrophy ADJ3 Bowman-layer-type-1) OR (Dermoids ADJ3 cornea) OR (Developmental-prosopagnosia) OR (Doyne-honeycomb-retinal-dystrophy) OR (Eales-diseas*) OR (Ectopia-lentis ADJ3 isolated-autosomal-recessive) OR (Enthesitis-related-juvenile-idiopathic-arthritis) OR (Epithelial-basement-membrane-corneal-dystrophy) OR (Familial-isolated-hypoparathyroidism) OR (Fuchs-heterochromic-iridocyclitis) OR (Fundus-dystrophy ADJ3 pseudoinflammatory ADJ3 -Sorsby) OR (Groenouw-type-I-corneal-dystrophy) OR (Intraocular-melanoma) OR (Keratoconus) OR (Krabbe-diseas*) OR (Lattice-corneal-dystrophy-type-3A) OR (Leber-congenital-amaurosis-1) OR (Leber-congenital-amaurosis-10) OR (Leber-congenital-amaurosis-11) OR (Leber-congenital-amaurosis-12) OR (Leber-congenital-amaurosis-13) OR (Leber-congenital-amaurosis-14) OR (Leber-congenital-amaurosis-15) OR (Leber-congenital-amaurosis-16) OR (Leber-congenital-amaurosis-2) OR (Leber-congenital-amaurosis-3) OR (Leber-congenital-amaurosis-4) OR (Leber-congenital-amaurosis-6) OR (Leber-congenital-amaurosis-9) OR (Microcornea-posterior-megalolenticonus-persistent-fetal-vasculature-coloboma) OR (Microcornea-corectopia-macular-hypoplasia) OR (Microspherophakia ADJ3 hernia) OR (Neuromyelitis-optica-spectrum-disorder*) OR (Neuronal-ceroid-lipofuscinosis) OR (Nystagmus-1 ADJ3 congenital ADJ3 X--linked) OR (Nystagmus-2 ADJ3 congenital ADJ3 autosomal-dominant) OR (O-Donnell-Pappas-syndrom*) OR (Ocular-neuromyotonia) OR (Opsoclonus-myoclonus-syndrom*) OR (Optic-atrophy-5) OR (Optic-atrophy-6) OR (Optic-neuritis) OR (Orbital-varix) OR (Panuveitis) OR (Pattern-dystrophy) OR (Pelizaeus-Merzbacher-diseas*) OR (Peters-anomaly) OR (Posterior-uveitis) OR (Pterygium ADJ3 conjunctiva ADJ3 cornea) OR (Punctate-inner-choroidopathy) OR (Reese-retinal-dysplasia) OR (Retinal-cone-dystrophy-2) OR (Retinal-cone-dystrophy-3A) OR (Retinal-cone-dystrophy-3B) OR (Retinal-cone-dystrophy-4) OR (Retinitis-pigmentosa-1) OR (Retinitis-Pigmentosa-11) OR (Retinitis-pigmentosa-12) OR (Retinitis-Pigmentosa-13) OR (Retinitis-Pigmentosa-14) OR (Retinitis-Pigmentosa-15) OR (Retinitis-Pigmentosa-17) OR (Retinitis-Pigmentosa-18) OR (Retinitis-Pigmentosa-19) OR (Retinitis-Pigmentosa-20) OR (Retinitis-Pigmentosa-22) OR (Retinitis-Pigmentosa-23) OR (Retinitis-Pigmentosa-24) OR (Retinitis-Pigmentosa-25) OR (Retinitis-Pigmentosa-26) OR (Retinitis-Pigmentosa-28) OR (Retinitis-pigmentosa-29) OR (Retinitis-pigmentosa-3) OR (Retinitis-Pigmentosa-30) OR (Retinitis-Pigmentosa-31) OR (Retinitis-Pigmentosa-32) OR (Retinitis-Pigmentosa-33) OR (Retinitis-Pigmentosa-34) OR (Retinitis-Pigmentosa-35) OR (Retinitis-Pigmentosa-36) OR (Retinitis-Pigmentosa-4) OR (Retinitis-Pigmentosa-41) OR (Retinitis-Pigmentosa-6) OR (Retinitis-Pigmentosa-7) OR (Retinitis-Pigmentosa-9) OR (Retinopathy ADJ3 prematurity) OR (Ring-dermoid ADJ3 cornea) OR (Sandhoff-diseas*) OR (Serpiginous-choroiditis) OR (Spondyloepiphyseal-dysplasia) OR (Superior-limbic-keratoconjunctivitis) OR (Tay-Sachs-diseas*) OR (Trachoma) OR (Tubulointerstitial-nephritis ADJ3 uveitis) OR (Usher-syndrom*) OR (Usher-syndrome-type-3A) OR (Usher-syndrome ADJ3 type-1B) OR (Usher-syndrome ADJ3 type-1C) OR (Usher-syndrome ADJ3 type-1D) OR (Usher-syndrome ADJ3 type-1E) OR (Usher-syndrome ADJ3 type-1F) OR (Usher-syndrome ADJ3 type-2B) OR (Usher-syndrome ADJ3 type-2C) OR (Uveal-diseases) OR (Vernal-keratoconjunctivitis) OR (Vogt-Koyanagi-Harada-diseas*) OR (Achard-Thiers-syndrom*) OR (Asherman ADJ3 syndrom*) OR (Benign-mesonephroma) OR (Diabetic-mastopathy) OR (Diethylstilbestrol-syndrom*) OR (Extramammary-Paget-diseas*) OR (Fowler ADJ3 syndrom*) OR (Granulomatous-lobular-mastitis) OR (HELLP-syndrom*) OR (Hydatidiform-mole) OR (Metaplastic-carcinoma ADJ3 breast) OR (Ovarian-carcinosarcoma) OR (Paget-disease ADJ3 breast) OR (Peripartum-cardiomyopathy) OR (Pruritic-urticarial-papules-plaques ADJ3 pregnancy) OR (Uterine-Carcinosarcoma) OR (Aberrant-subclavian-artery) OR (Arrhythmogenic-right-ventricular-cardiomyopathy) OR (Baroreflex-failure) OR (Bidirectional-tachycardia) OR (Broken-heart-syndrom*) OR (Brugada-syndrome-3) OR (Brugada-syndrome-4) OR (Cardiac-hydatid-cysts ADJ3 intracavitary-expansion) OR (Cardiac-rupture) OR (Chaotic-atrial-tachycardia) OR (Diffuse-cutaneous-systemic-sclerosis) OR (Familial-hypertrophic-cardiomyopathy) OR (Fibrocartilaginous-embolism) OR (Gaucher-diseas*) OR (Glycogen-storage-disease-type-2) OR (Intracranial-arteriovenous-malformation) OR (Kallikrein-hypertension) OR (Limited-cutaneous-systemic-sclerosis) OR (Limited-systemic-sclerosis) OR (Lymphocytic-vasculitis) OR (Neonatal-stroke) OR (Patent-ductus-arteriosus) OR (Patent-ductus-venosus) OR (Pulmonary-arterial-hypertension) OR (Pulmonary-valve-stenosis) OR (Pulmonic-stenosis) OR (Renoprival-hypertension) OR (Sudden-Arrhythmia-Death-syndrom*) OR (Duodenal-carcinoid-syndrom*) OR (Endolymphatic-sac-tumor) OR (Familial-adenomatous-polyposis) OR (Familial-isolated-pituitary-adenoma) OR (Hirschsprung-disease-ganglioneuroblastoma) OR (MYH-associated-polyposis) OR (Pheochromocytoma-islet-cell-tumor-syndrom*) OR (Premature-aging-Okamoto-type) OR (Stewart-Treves-syndrom*) OR (Adult-onset-immunodeficiency ADJ3 anti-interferon-gamma-autoantibodies) OR (Allergic-bronchopulmonary-aspergillosis) OR (Amyloidosis-AA) OR (Amyloidosis-familial-visceral) OR (Autosomal-recessive-hyper-IgE-syndrom*) OR (Bronchiolitis-obliterans) OR (Chronic-graft-versus-host-diseas*) OR (Complement-component-8-deficiency-type-1) OR (Complement-component-8-deficiency-type-2) OR (Cryoglobulinemic-vasculitis) OR (Felty ADJ3 syndrom*) OR (Hashimoto-encephalopathy) OR (Hyper-IgE-syndrom*) OR (Immune-dysfunction ADJ3 T-cell-inactivation-due-to-calcium-entry-defect-1) OR (Immune-dysfunction ADJ3 T-cell-inactivation-due-to-calcium-entry-defect-2) OR (Immunodeficiency ADJ3 thymoma) OR (Immunodeficiency ADJ3 anhidrotic-ectodermal-dysplasia) OR (Immunoglobulin-A-deficiency-2) OR (Lymphocytic-hypophysitis) OR (Melkersson-Rosenthal-syndrom*) OR (MHC-class-1-deficiency) OR (Multifocal-fibrosclerosis) OR (Neonatal-systemic-lupus-erythematosus) OR (Palindromic-rheumatism) OR (Pediatric-multiple-sclerosis) OR (Relapsing-polychondritis) OR (Schnitzler-syndrom*) OR (Severe-combined-immunodeficiency ADJ3 sensitivity-to-ionizing-radiation) OR (Severe-combined-immunodeficiency ADJ3 atypical) OR (Simple-cryoglobulinemia) OR (Stevens-Johnson-syndrom*) OR (toxic-epidermal-necrolysis) OR (X-linked-lymphoproliferative-syndrome-2) OR (Adult-onset-Still ADJ3 diseas*) OR (Autosomal-dominant-tubulointerstitial-kidney-diseas*) OR (Autosomal-dominant-tubulointerstitial-kidney-disease-due-to-MUC1-mutations) OR (BK-virus-nephropathy) OR (Collecting-duct-carcinoma) OR (Cystinuria) OR (Dermatomyositis) OR (Dihydroxyadeninuria) OR (Fibrillary-glomerulonephritis) OR (Glomerulonephritis) OR (IgA-nephropathy) OR (Immunotactoid-glomerulopathy) OR (Infundibulopelvic-dysgenesis) OR (Juvenile-dermatomyositis) OR (Juvenile-polymyositis) OR (Lupus-nephritis) OR (Membranous-nephropathy) OR (Methylmalonic-acidemia) OR (Minimal-change-diseas*) OR (Nephrocalcinosis) OR (Nephronophthisis) OR (Polyomavirus-allograft-nephropathy) OR (Postorgasmic-illness-syndrom*) OR (Renal-medullary-carcinoma) OR (Renal-tubular-dysgenesis) OR (Testicular-seminoma) OR (Asbestosis) OR (Autoimmune-pulmonary-alveolar-proteinosis) OR (Beryllium-diseas*) OR (Bronchiolitis-obliterans-organizing-pneumonia) OR (Catamenial-pneumothorax) OR (Children ADJ3 interstitial-lung-diseas*) OR (Chronic-thromboembolic-pulmonary-hypertension) OR (Coal-worker ADJ3 pneumoconiosis) OR (Costocoracoid-ligament-congenitally-short) OR (Cryptogenic-organizing-pneumonia) OR (Diffuse-idiopathic-pulmonary-neuroendocrine-cell-hyperplasia) OR (Diffuse-panbronchiolitis) OR (Fibrosing-mediastinitis) OR (Idiopathic-pulmonary-fibrosis) OR (Idiopathic-pulmonary-hemosiderosis) OR (Kaolin-pneumoconiosis) OR (Meconium-aspiration-syndrom*) OR (Nocardiosis) OR (Pleuroparenchymal-fibroelastosis) OR (Psoriatic-juvenile-idiopathic-arthritis) OR (Pulmonary-sequestration) OR (Respiratory-distress-syndrome ADJ3 infant) OR (Silicosis) OR (Systemic-onset-juvenile-idiopathic-arthritis) OR (Tracheobronchomalacia) OR (Tracheobronchopathia-osteoplastica) OR (Androgen-insensitivity-syndrom*) OR (Androgen-insensitivity-syndrome ADJ3 mild) OR (Hansen ADJ3 diseas*) OR (Prostatic-malacoplakia-associated ADJ3 prostatic-abscess) OR (Sertoli-cell-only-syndrom*) OR (2-Hydroxyglutaric-aciduria) OR (Abdominal-obesity-metabolic-syndrom*) OR (Acetyl-CoA-acetyltransferase-2-deficiency) OR (Acetyl-carnitine-deficiency) OR (Apparent-mineralocorticoid-excess) OR (Bartter-syndrom*) OR (Bartter-syndrome-antenatal-type-1) OR (Bartter-syndrome-antenatal-type-2) OR (Central-diabetes-insipidus) OR (Chondrocalcinosis-1) OR (Chondrocalcinosis-due-to-apatite-crystal-deposition) OR (Citrulline-transport-defect) OR (Congenital-disorder ADJ3 glycosylation-type-I) OR (Congenital-disorder ADJ3 glycosylation-type-IIX) OR (CoQ-responsive-OXPHOS-deficiency) OR (Cytochrome-c-oxidase-deficiency) OR (Dipsogenic-diabetes-insipidus) OR (Fatal-infantile-encephalomyopathy) OR (Gestational-diabetes-insipidus) OR (Glutathione-synthetase-deficiency) OR (Glycogen-storage-disease-8) OR (Glycoproteinosis) OR (Hereditary-amyloidosis) OR (Homocysteinemia) OR (Hyperglycerolemia) OR (Hypolipoproteinemia) OR (Infantile-free-sialic-acid-storage-diseas*) OR (Krabbe-disease-atypical-due-to-Saposin-A-deficiency) OR (Lactate-dehydrogenase-A-deficiency) OR (Lactate-dehydrogenase-deficiency) OR (Leucine-sensitive-hypoglycemia ADJ3 infancy) OR (Lipase-deficiency-combined) OR (Metachromatic-leukodystrophy) OR (Metachromatic-leukodystrophy-due-to-saposin-B-deficiency) OR (Morquio-syndrome-B) OR (Mucopolysaccharidosis-type-II) OR (Mucopolysaccharidosis-type-VI) OR (N-acetyltransferase-deficiency) OR (N-acetyl-alpha-D-galactosaminidase-deficiency-type-III) OR (Phosphoribosylpyrophosphate-synthetase-superactivity) OR (Primary-hyperoxaluria-type-3) OR (Pyruvate-carboxylase-deficiency) OR (Pyruvate-dehydrogenase-complex-deficiency) OR (Refsum-disease ADJ3 increased-pipecolic-acidemia) OR (Rhizomelic-chondrodysplasia-punctata-type-3) OR (Tiglic-acidemia) OR (Transcobalamin-1-deficiency) OR (Trehalase-deficiency) OR (Trimethylaminuria) OR (Urea-cycle ADJ3 disorder*) OR (Valinemia) OR (X-linked-adrenoleukodystrophy) OR (Ameloblastic-carcinoma) OR (Florid-cemento-osseous-dysplasia) OR (Gingival-fibromatosis ADJ3 1) OR (Gingival-fibromatosis ADJ3 2) OR (Gingival-fibromatosis ADJ3 3) OR (Gingival-fibromatosis ADJ3 4) OR (Hemifacial-myohyperplasia) OR (Oral-submucous-fibrosis) OR (Xanthogranulomatous-sialadenitis) OR (Acromesomelic-dysplasia) OR (Amyotrophy ADJ3 neurogenic-scapuloperoneal ADJ3 New-England-type) OR (Arthrogryposis-multiplex-congenita) OR (Axial-osteomalacia) OR (Baby-rattle-pelvic-dysplasia) OR (Cervical-dystonia) OR (Chondrosarcoma) OR (Chronic-recurrent-multifocal-osteomyelitis) OR (Coccygodynia) OR (Collagenopathy-type-2-alpha-1) OR (Congenital-radioulnar-synostosis) OR (Dysferlinopathy) OR (DYT-GNAL) OR (Erdheim-Chester-diseas*) OR (Ewing-sarcoma) OR (Familial-tumoral-calcinosis) OR (Freiberg ADJ3 diseas*) OR (Giant-cell-tumor ADJ3 bone) OR (Hyperphosphatemic-familial-tumoral-calcinosis) OR (Inclusion-body-myositis) OR (Iridogoniodysgenesis-type-1) OR (Kienbock ADJ3 diseas*) OR (Kohler-diseas*) OR (Lambert-Eaton-myasthenic-syndrom*) OR (Levator-syndrom*) OR (Macrophagic-myofasciitis) OR (MAGIC-syndrom*) OR (Monomelic-amyotrophy) OR (Multiple-epiphyseal-dysplasia) OR (Muscular-dystrophy) OR (Myostatin-related-muscle-hypertrophy) OR (Myotonic-dystrophy) OR (Neurofibromatosis-type-1) OR (Normophosphatemic-familial-tumoral-calcinosis) OR (Osteochondritis-dissecans) OR (Osteodysplasty-precocious ADJ3 Danks-Mayne ADJ3 Kozlowski) OR (Osteosarcoma) OR (Pigmented-villonodular-synovitis) OR (Piriformis-syndrom*) OR (Pleoconial-myopathy ADJ3 salt-craving) OR (Polycystic-bone-diseas*) OR (Pyoderma-gangrenosum) OR (Radio-ulnar-synostosis-type-1) OR (Radio-ulnar-synostosis-type-2) OR (Reactive-arthritis) OR (SAPHO-syndrom*) OR (Spheroid-body-myopathy) OR (Spinal-muscular-atrophy-Ryukyuan-type) OR (Spinal-muscular-atrophy-type-1 ADJ3 congenital-bone-fractures) OR (Spinal-muscular-atrophy-type-4) OR (Spinal-muscular-atrophy ADJ3 respiratory-distress-1) OR (Synovial-Chondromatosis) OR (Tarsal-tunnel-syndrom*) OR (Tietze-syndrom*) OR (Trochlea ADJ3 humerus-aplasia-of) OR (Trochlear-dysplasia) OR (Undifferentiated-pleomorphic-sarcoma) OR (X-linked-dominant-scapuloperoneal-myopathy) OR (Absence ADJ3 septum-pellucidum) OR (Adie-syndrom*) OR (Agnosia) OR (AIDS-Dementia-Complex) OR (Alzheimer-disease-type-4) OR (Alzheimer ADJ3 disease ADJ3 neurofibrillary-tangles) OR (Amyloid-neuropathy) OR (Amyotrophic-lateral-sclerosis) OR (Amyotrophic-lateral-sclerosis-type-6) OR (Amyotrophic-lateral-sclerosis-parkinsonism) OR (dementia-complex-1) OR (Anaplastic-astrocytoma) OR (Anaplastic-ganglioglioma) OR (Anaplastic-oligodendroglioma) OR (Antisynthetase-syndrom*) OR (Apraxia) OR (Arachnoiditis) OR (Autosomal-recessive-juvenile-Parkinson-diseas*) OR (Behavioral-variant ADJ3 frontotemporal-dementia) OR (Bell ADJ3 palsy) OR (Benign-rolandic-epilepsy) OR (Binswanger ADJ3 diseas*) OR (Bobble-head-doll-syndrom*) OR (Brown-Sequard-syndrom*) OR (Camptocormism) OR (CANOMAD-syndrom*) OR (Central-neurocytoma) OR (Central-pain-syndrom*) OR (Cerebellar-degeneration) OR (Cerebral-palsy-ataxic) OR (Cerebral-palsy-athetoid) OR (Cerebral-palsy-spastic-hemiplegic) OR (Cerebral-palsy-spastic-monoplegic) OR (Cerebral-palsy-spastic-quadriplegic) OR (Cerebral-sclerosis-similar-to-Pelizaeus-Merzbacher-diseas*) OR (Cerebrospinal-fluid-leak) OR (Charcot-Marie-Tooth-diseas*) OR (Chiari-malformation-type-4) OR (Choroid-plexus-carcinoma) OR (Choroid-plexus-papilloma) OR (Chronic-lymphocytic-inflammation ADJ3 pontine-perivascular-enhancement-responsive-to-steroids) OR (Coenzyme-Q10-deficiency) OR (Complex-regional-pain-syndrom*) OR (Creutzfeldt-Jakob-diseas*) OR (Cytomegalic-inclusion-diseas*) OR (Developmental-dysphasia-familial) OR (Dysautonomia-like-disorder*) OR (Dyssynergia-cerebellaris-myoclonica) OR (Eastern-equine-encephalitis) OR (Empty-sella-syndrom*) OR (Encephalitis-lethargica) OR (Ependymoma) OR (Epilepsy-occipital-calcifications) OR (Epilepsy-progressive-myoclonic-type-3) OR (Episodic-ataxia) OR (Familial-transthyretin-amyloidosis) OR (Frontotemporal-dementia) OR (Gangliocytoma) OR (Gerstmann-syndrom*) OR (Gliomatosis-cerebri) OR (Guillain-Barre-syndrom*) OR (Hemangioblastoma) OR (Hemicrania-continua) OR (Hereditary-spastic-paraplegia) OR (Herpes-zoster-oticus) OR (HTLV-1-associated-myelopathy) OR (tropical-spastic-paraparesis) OR (Hypothalamic-hamartomas) OR (Idiopathic-intracranial-hypertension) OR (Idiopathic-spinal-cord-herniation) OR (Intraneural-perineurioma) OR (Isaacs-syndrom*) OR (Juvenile-Huntington-diseas*) OR (Klumpke-paralysis) OR (Kuru) OR (Kuzniecky-Andermann-syndrom*) OR (La-Crosse-encephalitis) OR (Leukodystrophy ADJ3 dysmyelinating ADJ3 -spastic-paraparesis ADJ3 dystonia) OR (Lewis-Sumner-syndrom*) OR (Limbic-encephalitis ADJ3 LGI1-antibodies) OR (Lissencephaly-1) OR (Lissencephaly-X-linked) OR (Localized-hypertrophic-neuropathy) OR (Locked-in-syndrom*) OR (Logopenic-progressive-aphasia) OR (Macrothrombocytopenia-progressive-deafness) OR (Marchiafava-Bignami-diseas*) OR (Meralgia-paresthetica) OR (Microcephaly) OR (Migraine ADJ3 brainstem-aura) OR (Miller-Fisher-syndrom*) OR (Morvan ADJ3 fibrillary-chorea) OR (Multifocal-motor-neuropathy) OR (Myasthenia-gravis) OR (Myelomeningocele) OR (Narcolepsy) OR (Necrotizing-autoimmune-myopathy) OR (Neonatal-meningitis) OR (Neuroblastoma) OR (Neurocutaneous-melanosis) OR (Neuroleptic-malignant-syndrom*) OR (New-onset-refractory-status-epilepticus) OR (Non-24-hour-sleep-wake-disorder*) OR (Nondystrophic-myotonia) OR (Olfactory-neuroblastoma) OR (Oligoastrocytoma) OR (Oligodendroglioma) OR (Olivopontocerebellar-atrophy) OR (Painful-orbital ADJ3 systemic-neurofibromas-marfanoid-habitus-syndrom*) OR (Pantothenate-kinase-associated-neurodegeneration) OR (Paralysis-agitans ADJ3 juvenile ADJ3 -Hunt) OR (Parkinson-disease-type-3) OR (Paroxysmal-hemicrania) OR (Parsonage-Turner-syndrom*) OR (Periventricular-leukomalacia) OR (Photosensitive-epilepsy) OR (Plasmacytoma) OR (Pleomorphic-xanthoastrocytoma) OR (Poliomyelitis) OR (Polyarteritis-nodosa) OR (Pontocerebellar-hypoplasia) OR (Post-Polio-syndrom*) OR (Posterior-column-ataxia) OR (Primary-amebic-meningoencephalitis) OR (Primary-melanoma ADJ3 central-nervous-system) OR (Primary-orthostatic-tremor) OR (Primary-progressive-aphasia) OR (Progressive-bulbar-palsy) OR (Progressive-hemifacial-atrophy) OR (Pudendal-Neuralgia) OR (Pure-autonomic-failure) OR (Radiation-induced-brachial-plexopathy) OR (Rasmussen-encephalitis) OR (Restless-legs-syndrome ADJ3 susceptibility-to ADJ3 1) OR (Restless-legs-syndrome ADJ3 susceptibility-to ADJ3 2) OR (Restless-legs-syndrome ADJ3 susceptibility-to ADJ3 3) OR (Restless-legs-syndrome ADJ3 susceptibility-to ADJ3 4) OR (Restless-legs-syndrome ADJ3 susceptibility-to ADJ3 5) OR (Restless-legs-syndrome ADJ3 susceptibility-to ADJ3 6) OR (Reversible-cerebral-vasoconstriction-syndrom*) OR (Rhabdoid-tumor) OR (Shapiro-syndrom*) OR (Spastic-diplegia-cerebral-palsy) OR (Spastic-diplegia-infantile-type) OR (Spastic-paraplegia-39) OR (Spinal-meningioma) OR (Spinal-shock) OR (Spinocerebellar-ataxia) OR (Spinocerebellar-ataxia-3) OR (Spinocerebellar-ataxia-30) OR (Spinocerebellar-ataxia-9) OR (Spinocerebellar-ataxia-autosomal-recessive-6) OR (Spinocerebellar-ataxia-X-linked-type-2) OR (Status-epilepticus) OR (Subependymal-giant-cell-astrocytoma) OR (Subependymoma) OR (Symmetrical-thalamic-calcifications) OR (Tarlov-cysts) OR (Thyrotoxic-periodic-paralysis) OR (Transverse-myelitis) OR (Trichinosis) OR (Trigeminal-neuralgia) OR (Tumefactive-multiple-sclerosis) OR (Variant-Creutzfeldt-Jakob-diseas*) OR (Visual-snow-syndrom*) OR (Wernicke-Korsakoff-syndrom*) OR (Worster-Drought-syndrom*) OR (2-4-Dienoyl-CoA-reductase-deficiency) OR (21-hydroxylase-deficiency) OR (Carnitine-palmitoyltransferase-2-deficiency) OR (Citrullinemia-type-I) OR (Congenital-human-immunodeficiency-virus) OR (Congenital-hypothyroidism) OR (Glucose-6-phosphate-dehydrogenase-deficiency) OR (Maple-syrup-urine-diseas*) OR (Medium-chain-3-ketoacyl-coa-thiolase-deficiency) OR (Methylmalonic-acidemia ADJ3 homocystinuria) OR (Mucopolysaccharidosis-type-I) OR (Phenylketonuria) OR (Tetrahydrobiopterin-deficiency) OR (Keshan-diseas*) OR (Rickets) OR (Scurvy) OR (Cutaneous-sclerosis) OR (Focal-task-specific-dystonia) OR (Abdominal-chemodectomas ADJ3 cutaneous-angiolipomas) OR (Acrodermatitis) OR (Actinic-lichen-planus) OR (Adiposis-dolorosa) OR (Ainhum) OR (Annular-atrophic-lichen-planus) OR (Annular-lichen-planus) OR (Atrophic-lichen-planus) OR (Atrophoderma ADJ3 Pasini ADJ3 Pierini) OR (Atrophoderma-vermiculata) OR (Basaran-Yilmaz-syndrom*) OR (Becker ADJ3 nevus) OR (Benign-eccrine-spiradenoma) OR (Brunsting-Perry-syndrom*) OR (Cheilitis-glandularis) OR (Chromhidrosis) OR (Corticosteroid-sensitive-aseptic-abscesses) OR (Cutaneous-collagenous-vasculopathy) OR (Cutaneous-polyarteritis-nodosa) OR (Cutis-verticis-gyrata) OR (Dermal-eccrine-cylindroma) OR (Dermatitis-herpetiformis) OR (Diffuse-dermal-angiomatosis) OR (Dystrophic-epidermolysis-bullosa) OR (Elastoderma) OR (Eosinophilic-pustular-folliculitis) OR (Epidermolysis-bullosa) OR (Epidermolysis-bullosa-acquisita) OR (Epidermolysis-bullosa-simplex) OR (Epidermolysis-bullosa-simplex ADJ3 generalized) OR (Erythema-multiforme) OR (Erythema-nodosum ADJ3 idiopathic) OR (Erythrokeratodermia-variabilis-et-progressiva) OR (Familial-dermographism) OR (Familial-multiple-trichodiscomas) OR (Febrile-Ulceronecrotic-Mucha-Habermann-diseas*) OR (Fox-Fordyce-diseas*) OR (Frontal-fibrosing-alopecia) OR (Granuloma-annulare) OR (Granulomatous-rosacea) OR (Guttate-psoriasis) OR (Halal-Setton-Wang-syndrom*) OR (Halo-nevus) OR (Hydroa-vacciniforme) OR (Hydroa-vacciniforme ADJ3 familial) OR (Hypertrichosis-lanuginosa ADJ3 acquired) OR (Hypohidrotic-ectodermal-dysplasia) OR (Ichthyosis-vulgaris) OR (Ichthyosis ADJ3 acquired) OR (Junctional-epidermolysis-bullosa-inversa) OR (Keratosis-palmoplantaris-striata-1) OR (Keratosis-palmoplantaris-striata-3) OR (Kyrle-diseas*) OR (Lichen-planopilaris) OR (Lichen-planus-pemphigoides) OR (Lichen-planus-pigmentosus) OR (Lichen-sclerosus) OR (Linear-IgA-diseas*) OR (Linear-lichen-planus) OR (Linear-scleroderma) OR (Localized-scleroderma) OR (Lupus-erythematosus-tumidus) OR (Lymphocytic-infiltrate ADJ3 Jessner) OR (Morphea) OR (Mucous-membrane-pemphigoid) OR (Multicentric-reticulohistiocytosis) OR (Necrobiotic-xanthogranuloma) OR (Nelson-syndrom*) OR (Nephrogenic-Systemic-Fibrosis) OR (Nodular-nonsuppurative-panniculitis) OR (Palmoplantar-keratoderma) OR (Parapsoriasis) OR (Peeling-skin-syndrom*) OR (Pemphigus-vulgaris) OR (Pigmented-purpuric-dermatosis) OR (Pityriasis-lichenoides) OR (Pityriasis-lichenoides-chronica) OR (Pityriasis-lichenoides-et-varioliformis-acuta) OR (Porokeratosis ADJ3 disseminated-superficial-actinic-1) OR (Primary-cutaneous-amyloidosis) OR (Progestogen-hypersensitivity) OR (Pseudoainhum) OR (Pseudopelade ADJ3 Brocq) OR (Quinquaud-folliculitis-decalvans) OR (Red-skin-pigment-anomaly ADJ3 New-Guinea) OR (Rhabdomyomatous-mesenchymal-hamartoma) OR (Scleromyxedema) OR (Sjogren-Larsson-like-syndrom*) OR (Spitz-nevus) OR (Subcorneal-pustular-dermatosis) OR (Syringocystadenoma-papilliferum) OR (Systemic-scleroderma) OR (Trichostasis-spinulosa) OR (Wells-syndrom*) OR (Xanthoma-disseminatum) OR (Xeroderma-pigmentosum ADJ3 variant-type)).ab,ti,kf,sh,rs.)

### Grey literature search

| **Recommendation** | **Website/database** | **Search Term** |
| --- | --- | --- |
| Cochrane and Paez 2017 | Wonder | NA |
|  | scopus | ( TITLE-ABS-KEY ( rare  AND  disease )  AND  TITLE-ABS-KEY ( disability-adjusted  AND  life  AND  years ) )  AND  PUBYEAR  >  1989 |
|  | Clinicaltrials.gov | rare diseases |
|  | google scholar | "daly" burden "rare disease" -infection -cancer -tumor -myeloid |
|  | worldwidescience.org | “rare disease” and burden |
|  | grey literature report | “rare disease” and burden |
|  | University of Michigan Library, Grey Literature Overview | Daly/ yll/ yld |
|  | MDPI | burden |
|  | ProQuest | Burden AND “rare disease” AND daly |
|  | Worldcat | burden AND "rare disease" |
|  | OAIster | kw:(burden) AND ti:(rare disease) NOT au:(cancer) NOT kw:(infection) |
|  | DANS | Burden AND rare |
| The Canadian Agency for Drugs & Technologies in Health (CADTH) | The International Network of Agencies for Health Technology Assessment (INAHTA) | “burden” |
|  | Agency for Healthcare Research and Quality (AHRQ) | “burden” |
|  | Ideas database (IDEAS) | “Burden rare disease” |
|  | Value in Health | “Burden rare disease” |
|  | University of York: center for reviews and dissemination (CRD) | “burden” |
|  | Bandolier | Burden rare disease |
|  | Health systems evidence | Burden rare |
|  | National Institute for Health and Care Excellence (NICE) | Burden rare |
|  | National Institute for Health and Care Excellence (TRIP) | "rare disease" AND burden NOT cancer |
|  | Lund University Libraries (DOAJ) | Burden rare disease |
| Populational health and/or rare diseases focused websites | WHO | “burden”/ “effectiveness” |
|  | EMA | “burden” AND “rare disease” |
|  | FDA | "premature mortality" "Disability-adjusted life years" YLL YLD |
|  | NORD | burden |
|  | EURORDIS | burden |
|  | MetabERN | burden |
|  | IRDiRC | burden |

## Complete version of the data extraction

The complete version of the data extraction of burden of chronic non-communicable rare disease studies is provided as an excel file format attachment, under the name “Complete version of the data extraction”. The complete version of the data extraction includes the items presented in the manuscript and additional items that were not mentioned.

The data extraction form was based on a previous systematic review (Charalampous et al 2022). However, to better suit the present study, which focuses on a different group of diseases, some items were excluded while additional items were added. The following items were maintained: *Title, Author(s), Year, Funding body, Country(s)/region, Reference year, data source mortality/YLL, Data source incidence/prevalence/YLD, Internal consistency, Use of DisMod, Perspective of YLD estimates, Life Expectancy for YLL, Study developed own DWs, Source of DW, Severity distribution, Comorbidity adjustment, Age weighting, Time discounting, Uncertainty analysis and Sensitivity analysis.* Moreover*,* the following items were add in addition: *Reference time period, Was full text available?, Disease(s) included, Corresponding disease group, Epidemiological metric unit, Epidemiological estimate, Crude DALY, Crude YLL, Crude YLD, DALYs crude rate, DALY per incident case* and *DALY per prevalent case.*

## Definitions of the items included in the data extraction

| General information | Author(s) | List of author(s) with the use of **APA** style**.** | |
| --- | --- | --- | --- |
|  | Reference time period | Period from when the observations were taken, expressed in dates. | |
|  | Geographic coverage | City, country, or geographic region from where the observations were retrieved. | |
|  | Was full text available? | Yes or No. Full-text paper of each identified study was searched in peer-review databases (Embase, Medline, and Pubmed), and gray literature databases (google scholar). The study was included as an abstract when its full text was not found. | |
|  | Year of publication | The year that the selected BoD study was published. | |
|  | Funding  *(source)* | Yes or no. If funding was provided, the source was provided and categorized as a non-profit organization (NPO), research fellowships and/or pharmaceuticals industry. | |
| Disease information | Disease(s) included | Name of the disease according to the paper | |
|  | Disease(s) classification | Disease(s) were categorized according to the chapter names of the 11th revision of the International Statistical Classification of Diseases and Related Health Problems (ICD-11)- see table 1. | |
| Data input sources | Data source mortality/YLL | *If not reported- NR*  *Relevant sources for mortality data:* National statistics, disease registries, survey data, vital registration systems, verbal autopsies, hospital records, literature, etc. | |
|  | Data source incidence/prevalence/YLD | *If not reported-NR*  *Relevant data sources for morbidity data:* Literature, disease registries, patient registries, survey datasets, surveillance systems, hospital records, etc | |
| YLD methods | Perspective of YLD estimates | - *Prevalence-based* perspective takes point prevalence measures of disability, adjusted for seasonal variation.   *Incidence-based* perspective captures the BoD in new diagnostic cases during a reference time-period and links all possible sequelae in future through an outcome tree or disease progression model. | |
|  | Study developed own DWs? | [Yes/No]  If yes, the quality of life questionnaire used in the respective study or by the study from which such data was extracted was reported. If not, the source of DWs was reported.  *Relevant sources*: GBD DWs, Dutch DWs, Empirical DWs etc. | |
|  | Study used DWs from other disease? | [Yes/No]  If study use a DWs set developed for a disease, under the assumption both diseases have similar manifestations and progression. | |
|  | DW: severity distribution | The proportion of cases with e.g., mild, moderate or severe health state^1^ of a specific outcome for which separate DWs are available.  [Yes/No]  *Was a severity distribution used/reported by the authors?* [Global/National]   - ^1^: a health state reflects a combination of signs or symptoms that result in a certain amount of health loss | |
|  | Comorbidity adjustment (YLD calculation) | [Yes/No]  Adjustment of YLD data for comorbidity^2^  ^2^: multiple conditions co-existing in one individual | |
| YLL methods | Life-expectancy source | The life table that was used to assess YLL  *Relevant life-tables*: Aspirational standard life tables, i.e., WHO standard life table, GBD standard life table OR National life tables (i.e., country-specific) | |
| General BoD methods | Internal consistency | [Yes/No]  *Were adjustments made to ensure that the sum of cause‐specific mortality or impairments equals all‐cause mortality or impairments?* | |
|  | Use of DisMod | [Yes/No]  DisMod is s a software tool that may be used to check the consistency of estimates of incidence, prevalence, duration and case fatality for diseases.  *Did the authors mention the use of DisMoD?* | |
|  | Age weighting and discounting rate | [Yes/No]  By incorporating age-weighting into DALY implies that the value of life depends on age; a lower weight of healthy life years lived is given at younger and at older ages – known as ‘non-uniform DALY’ | |
|  | Time discounting and discounting rate | [Yes/No]  Time-discounting discounts future years of healthy life lived using a rate of 3% or an alternative set of 0% | |
|  | Uncertainty analysis | [Yes/No]  An estimation of range or distribution of uncertainty in estimates based on an assessment of the uncertainty or confidence intervals for all data and parameter inputs  *Relevant methods of uncertainty in DALY calculations:* Parameter uncertainty, Structural or model uncertainty, Methodological uncertainty | |
|  | Sensitivity analysis | [Yes/No]  Analysis of how the impact of uncertainties of one or more input variables can lead to uncertainties in data inputs or assumptions | |
| DALY per case estimation | Epidemiological metric unit | | Epidemiological metric used to estimate YLD, such as incidence or prevalence. Expressed in number of cases in the study sample or disease rate in the population |
|  | Epidemiological estimate | | Number of cases or disease rate applied to YLD calculation. |
|  | Crude DALY | | Absolute number of DALYs calculated in the study population. |
|  | Crude YLL | | Absolute number of YLLs calculated in the study population. |
|  | Crude YLD | | Absolute number of YLDs calculated in the study population. |
|  | Crude DALY rate | | Absolute number of DALYs calculated per 100 000 people. |
|  | DALY per incident case | | The average healthy-years of life of live lost during a lifetime of an individual with a disease. |
|  | DALY per prevalent case | | The average healthy-years of life of live lost per year lived by the individual with the disease. It includes information on years of life lost due to premature mortality, which is the reason why the DALY per prevalent case, as an annual estimate, can still account for more than a single year of healthy-years of life lost, annually. |
| BoD: burden of disease; DALY: disability adjusted life years; DW: disability weight; GBD: Global Burden of Disease; YLD: years lost due to disability; YLL: years of life lost due to premature mortality; WHO: World Health Organisation | | | |

## The 11th revision of the International Statistical Classification of Diseases and Related Health Problems (ICD-11)

Table 1 Overview of ICD-11 chapters

| **Chapter** | **Matching the search criteria** |
| --- | --- |
| Chapter 01 – Infectious diseases | ☓ |
| Chapter 02 – Neoplasms | ☓ |
| Chapter 03 – Diseases of the blood and bloodforming organs | ✓ |
| Chapter 04 – Disorders of the immune system | ✓ |
| Chapter 05 – Conditions related to sexual health | ✓ |
| Chapter 06 – Endocrine, nutritional and metabolic diseases | ✓ |
| Chapter 07 – Mental and behavioural disorders | ✓ |
| Chapter 08 – Sleep – Wake disorders | ✓ |
| Chapter 09 – Diseases of the nervous system | ✓ |
| Chapter 10 – Diseases of the eye and adnexa | ✓ |
| Chapter 11 - Diseases of the ear and mastoid process | ✓ |
| Chapter 12 – Diseases of the circulatory system | ✓ |
| Chapter 13 – Diseases of the respiratory system | ✓ |
| Chapter 14 – Diseases of the digestive system | ✓ |
| Chapter 15 – Diseases of the skin | ✓ |
| Chapter 16 – Diseases of the musculoskeletal system and connective tissue | ✓ |
| Chapter 17 – Diseases of the genitourinary system | ✓ |
| Chapter 18 – Pregnancy, childbirth and the puerperium | ✓ |
| Chapter 19 – Certain conditions originating in the perinatal period | ✓ |
| Chapter 20 – Developmental anomalies | ✓ |
| Chapter 21 – Symptoms, signs, clinical forms, and abnormal clinical and laboratory findings, not elsewhere classified | ☓ |
| Chapter 22 – Injury, poisoning and certain other consequences of external causes | ☓ |
| Chapter 23 – External causes of morbidity and mortality | ☓ |
| Chapter 24 – Factors influencing health status and contact with health services | ☓ |
| Chapter 25 – Codes for special purposes | ☓ |
| Chapter 26 –Extension Codes | ☓ |
| Chapter 27 – Traditional Medicine | ☓ |

# Chapter 2

## Quality of reporting assessment of included studies with full-text available

|  |  | Abolhassani et al | Café et al | Chung et al | GBD 2016 MND collaborators | GBD 2019 Disease and injuries collaborators | Guojun et al | Henrard et al. | Inês et al | Kansal et al | Liu et al | Odnoletkova et al. | Siddiqi et al | Villaverde-Hueso et al |
| --- | --- | --- | --- | --- | --- | --- | --- | --- | --- | --- | --- | --- | --- | --- |
| A | Objectives and funding |  | | | | | | | | | | | | |
| 1 | Define the indicator(s), populations (including age, sex, and geographic entities), and time period(s) for which estimates were made. |  |  |  |  |  |  |  |  |  |  |  |  |  |
| 2 | List the funding sources for the work. |  |  |  |  |  |  |  |  |  |  |  |  |  |
| B | Data inputs |  | | | | | | | | | | | | |
| *B.1* | *For all data inputs from multiple sources that are synthesised as part of the study:* |  | | | | | | | | | | | | |
| 3 | Describe how the data were identified and how the data were accessed. |  |  |  |  |  |  |  |  |  |  |  |  |  |
| 4 | Specify the inclusion and exclusion criteria. Identify all ad-hoc exclusions. |  |  |  |  |  |  |  |  |  |  |  |  |  |
| 5 | Provide information about all included data sources and their main characteristics. For each data source used, report reference information or contact name/institution, population represented, data collection method, year(s) of data collection, sex and age range, diagnostic criteria or measurement method, and sample size, as relevant. |  |  |  |  |  |  |  |  |  |  |  |  |  |
| 6 | Identify and describe any categories of input data that have potentially important biases (eg, based on characteristics listed in item 5). |  |  |  |  |  |  |  |  |  |  |  |  |  |
| *B.2* | *For data inputs that contribute to the analysis but were not synthesised as part of the study:* |  | | | | | | | | | | | | |
| 7 | Describe and give sources for any other data inputs. |  |  |  |  |  |  |  |  |  |  |  |  |  |
| *B.3* | *For all data inputs:* |  | | | | | | | | | | | | |
| 8 | Provide all data inputs in a file format from which data can be efficiently extracted (eg, a spreadsheet rather than a PDF), including all relevant meta-data listed in item 5. For any data inputs that cannot be shared because of ethical or legal reasons, such as third-party ownership, provide a contact name or the name of the institution that retains the right to the data. |  |  |  |  |  |  |  |  |  |  |  |  |  |
| C | Data analysis |  | | | | | | | | | | | | |
| 9 | Provide a conceptual overview of the data analysis method. A diagram may be helpful. |  |  |  |  |  |  |  |  |  |  |  |  |  |
| 10 | Provide a detailed description of all steps of the analysis, including mathematical formulae. This description should cover, as relevant, data cleaning, data pre-processing, data adjustments and weighting of data sources, and mathematical or statistical model(s). |  |  |  |  |  |  |  |  |  |  |  |  |  |
| 11 | Describe how candidate models were evaluated and how the final model(s) were selected. |  |  |  |  |  |  |  |  |  |  |  |  |  |
| 12 | Provide the results of an evaluation of model performance, if done, as well as the results of any relevant sensitivity analysis. |  |  |  |  |  |  |  |  |  |  |  |  |  |
| 13 | Describe methods of calculating uncertainty of the estimates. State which sources of uncertainty were, and were not, accounted for in the uncertainty analysis. |  |  |  |  |  |  |  |  |  |  |  |  |  |
| 14 | State how analytical or statistical source code used to generate estimates can be accessed. |  |  |  |  |  |  |  |  |  |  |  |  |  |
| D | Results and discussion |  | | | | | | | | | | | | |
| 15 | Provide published estimates in a file format from which data can be efficiently extracted. |  |  |  |  |  |  |  |  |  |  |  |  |  |
| 16 | Report a quantitative measure of the uncertainty of the estimates (eg, uncertainty intervals). |  |  |  |  |  |  |  |  |  |  |  |  |  |
| 17 | Interpret results in light of existing evidence. If updating a previous set of estimates, describe the reasons for changes in estimates. |  |  |  |  |  |  |  |  |  |  |  |  |  |
| 18 | Discuss limitations of the estimates. Include a discussion of any modelling assumptions or data limitations that aff ect interpretation of the estimates. |  |  |  |  |  |  |  |  |  |  |  |  |  |
|  | **According to GATHER (%)** | **55,6** | **66,8** | **72,2** | **100,0** | **100,0** | **61,1** | **72,2** | **61,1** | **72,2** | **77,8** | **83,3** | **61,1** | **72,2** |

*Note: A light green box implies that the study checked the corresponding item; a white box implies that the study did not check the corresponding item; a grey colour is used to indicate a box that does not involve an item as it corresponds to a title/subtitle of the checklist chapter OR not applicable item for the respective study design (kansal et al performed a meta-analysis)*

# Chapter 3

## The Preferred Reporting Items for Systematic Reviews and Meta-Analyses (PRISMA) checklist

### Abstract checklist

| **Section and Topic** | **Item #** | **Checklist item** | **Reported (Yes/No)** |
| --- | --- | --- | --- |
| **TITLE** | | |  |
| Title | 1 | Identify the report as a systematic review. | Yes |
| **BACKGROUND** | | |  |
| Objectives | 2 | Provide an explicit statement of the main objective(s) or question(s) the review addresses. | Yes |
| **METHODS** | | |  |
| Eligibility criteria | 3 | Specify the inclusion and exclusion criteria for the review. | Yes |
| Information sources | 4 | Specify the information sources (e.g. databases, registers) used to identify studies and the date when each was last searched. | Yes |
| Risk of bias | 5 | Specify the methods used to assess risk of bias in the included studies. | No |
| Synthesis of results | 6 | Specify the methods used to present and synthesise results. | Yes |
| **RESULTS** | | |  |
| Included studies | 7 | Give the total number of included studies and participants and summarise relevant characteristics of studies. | Yes |
| Synthesis of results | 8 | Present results for main outcomes, preferably indicating the number of included studies and participants for each. If meta-analysis was done, report the summary estimate and confidence/credible interval. If comparing groups, indicate the direction of the effect (i.e. which group is favoured). | Yes |
| **DISCUSSION** | | |  |
| Limitations of evidence | 9 | Provide a brief summary of the limitations of the evidence included in the review (e.g. study risk of bias, inconsistency and imprecision). | No |
| Interpretation | 10 | Provide a general interpretation of the results and important implications. | Yes |
| **OTHER** | | |  |
| Funding | 11 | Specify the primary source of funding for the review. | Lines 459-460; funding |
| Registration | 12 | Provide the register name and registration number. | Lines 102-104; methods |

### PRISMA 2020 checklist

| **Section and Topic** | **Item #** | **Checklist item** | **Location where item is reported** |
| --- | --- | --- | --- |
| **TITLE** | | |  |
| Title | 1 | Identify the report as a systematic review. | Line 1 |
| **ABSTRACT** | | |  |
| Abstract | 2 | See the PRISMA 2020 for Abstracts checklist. | Lines 23-43 |
| **INTRODUCTION** | | |  |
| Rationale | 3 | Describe the rationale for the review in the context of existing knowledge. | Lines 76-89 |
| Objectives | 4 | Provide an explicit statement of the objective(s) or question(s) the review addresses. | Lines 89-97 |
| **METHODS** | | |  |
| Eligibility criteria | 5 | Specify the inclusion and exclusion criteria for the review and how studies were grouped for the syntheses. | Lines 126-147; inclusion and exclusion criteria |
| Information sources | 6 | Specify all databases, registers, websites, organisations, reference lists and other sources searched or consulted to identify studies. Specify the date when each source was last searched or consulted. | Lines 106-124; search strategy and data sources |
| Search strategy | 7 | Present the full search strategies for all databases, registers and websites, including any filters and limits used. | Supplementary material; search strategy |
| Selection process | 8 | Specify the methods used to decide whether a study met the inclusion criteria of the review, including how many reviewers screened each record and each report retrieved, whether they worked independently, and if applicable, details of automation tools used in the process. | Lines 149-158; data screening and extraction |
| Data collection process | 9 | Specify the methods used to collect data from reports, including how many reviewers collected data from each report, whether they worked independently, any processes for obtaining or confirming data from study investigators, and if applicable, details of automation tools used in the process. | Lines 149-158; data screening and extraction |
| Data items | 10a | List and define all outcomes for which data were sought. Specify whether all results that were compatible with each outcome domain in each study were sought (e.g. for all measures, time points, analyses), and if not, the methods used to decide which results to collect. | Lines 160-173; data synthesis |
|  | 10b | List and define all other variables for which data were sought (e.g. participant and intervention characteristics, funding sources). Describe any assumptions made about any missing or unclear information. | Lines 160-173; data synthesis |
| Study risk of bias assessment | 11 | Specify the methods used to assess risk of bias in the included studies, including details of the tool(s) used, how many reviewers assessed each study and whether they worked independently, and if applicable, details of automation tools used in the process. | Lines 190-194; quality of reporting assessment |
| Effect measures | 12 | Specify for each outcome the effect measure(s) (e.g. risk ratio, mean difference) used in the synthesis or presentation of results. | Lines 175-188; DALY per case calculation |
| Synthesis methods | 13a | Describe the processes used to decide which studies were eligible for each synthesis (e.g. tabulating the study intervention characteristics and comparing against the planned groups for each synthesis (item #5)). | Lines 160-173; data synthesis |
|  | 13b | Describe any methods required to prepare the data for presentation or synthesis, such as handling of missing summary statistics, or data conversions. | NA |
|  | 13c | Describe any methods used to tabulate or visually display results of individual studies and syntheses. | Lines 172-173; data synthesis |
|  | 13d | Describe any methods used to synthesize results and provide a rationale for the choice(s). If meta-analysis was performed, describe the model(s), method(s) to identify the presence and extent of statistical heterogeneity, and software package(s) used. | Supplementary material, Definitions of the items included in the data extraction |
|  | 13e | Describe any methods used to explore possible causes of heterogeneity among study results (e.g. subgroup analysis, meta-regression). | NA |
|  | 13f | Describe any sensitivity analyses conducted to assess robustness of the synthesized results. | NA |
| Reporting bias assessment | 14 | Describe any methods used to assess risk of bias due to missing results in a synthesis (arising from reporting biases). | Lines 190-194; quality of reporting assessment |
| Certainty assessment | 15 | Describe any methods used to assess certainty (or confidence) in the body of evidence for an outcome. | NA |
| **RESULTS** | | |  |
| Study selection | 16a | Describe the results of the search and selection process, from the number of records identified in the search to the number of studies included in the review, ideally using a flow diagram. | Line 198-209 & Figure 1; literature search |
|  | 16b | Cite studies that might appear to meet the inclusion criteria, but which were excluded, and explain why they were excluded. | Line 198-209 & Figure 1; literature search |
| Study characteristics | 17 | Cite each included study and present its characteristics. | Line 211- 251 & Table 1 & Figure 2, 3 and 4; study characteristics |
| Risk of bias in studies | 18 | Present assessments of risk of bias for each included study. | Supplementary material; quality of reporting assessment of included studies with full-text |
| Results of individual studies | 19 | For all outcomes, present, for each study: (a) summary statistics for each group (where appropriate) and (b) an effect estimate and its precision (e.g. confidence/credible interval), ideally using structured tables or plots. | Supplementary material, Complete version data extraction |
| Results of syntheses | 20a | For each synthesis, briefly summarise the characteristics and risk of bias among contributing studies. | Line 211- 251 & Table 1 & Figure 2, 3, 4 and 5 & line 300-323; study characteristics & DALY per case & quality of reporting assessment |
|  | 20b | Present results of all statistical syntheses conducted. If meta-analysis was done, present for each the summary estimate and its precision (e.g. confidence/credible interval) and measures of statistical heterogeneity. If comparing groups, describe the direction of the effect. | NA |
|  | 20c | Present results of all investigations of possible causes of heterogeneity among study results. | NA |
|  | 20d | Present results of all sensitivity analyses conducted to assess the robustness of the synthesized results. | NA |
| Reporting biases | 21 | Present assessments of risk of bias due to missing results (arising from reporting biases) for each synthesis assessed. | Lines 316-323 & supplementary material; quality of reporting assessment |
| Certainty of evidence | 22 | Present assessments of certainty (or confidence) in the body of evidence for each outcome assessed. | NA |
| **DISCUSSION** | | |  |
| Discussion | 23a | Provide a general interpretation of the results in the context of other evidence. | Lines 326-422; discussion |
|  | 23b | Discuss any limitations of the evidence included in the review. | Lines 424-435; strengths and limitations |
|  | 23c | Discuss any limitations of the review processes used. | NA |
|  | 23d | Discuss implications of the results for practice, policy, and future research. | Lines 326-422 & lines 437-446; discussion & conclusion |
| **OTHER INFORMATION** | | |  |
| Registration and protocol | 24a | Provide registration information for the review, including register name and registration number, or state that the review was not registered. | Lines 102-104; methods |
|  | 24b | Indicate where the review protocol can be accessed, or state that a protocol was not prepared. | Lines 102-104; methods |
|  | 24c | Describe and explain any amendments to information provided at registration or in the protocol. | NA |
| Support | 25 | Describe sources of financial or non-financial support for the review, and the role of the funders or sponsors in the review. | Lines 459-460; funding |
| Competing interests | 26 | Declare any competing interests of review authors. | Lines 455-457; competing interests |
| Availability of data, code and other materials | 27 | Report which of the following are publicly available and where they can be found: template data collection forms; data extracted from included studies; data used for all analyses; analytic code; any other materials used in the review. | Lines 523-531; Additional files |

# Chapter 4

## References

### Reference list of the included burden chronic non-communicable rare disease studies

1. Abolhassani H, Aghamohammadi A, Abolhassani F, Eftekhar H, Heidarnia M, Rezaei N. Health policy for common variable immunodeficiency: Burden of the disease. J Invest Allergol Clin Immunol. 2011;21(6):454-8.

2. Acuna P, Go C. Disability-adjusted life years (DALY) in x-linked dystonia parkinsonism. Mov Disord. 2017;32:789.

3. Ana Cristina Silva Pinto M, PhD , Fernando Ferreira Costa, MD, PhD , Sandra Fatima Menosi Gualandro, MD, PhD , Patricia Belintani Blum Fonseca , Carolina Tosin Bueno,, Cançado PaRD. 3409 Burden of Sickle Cell Disease: A Brazilian Societal Perspective Analysis. 2020.

4. Café A, Carvalho M, Crato M, Faria M, Kjollerstrom P, Oliveira C, et al. Haemophilia A: Health and economic burden of a rare disease in Portugal. Orphanet J Rare Dis. 2019;14(1).

5. Chung SE, Cheong HK, Park JH, Kim HJ. Burden of disease of multiple sclerosis in Korea. Epidemiol Health. 2012;34:e2012008.

6. Costa J, Coelho T, Moreno T, Negrão L, Ribeiro J, Santos M, et al. POSA102 Burden of Disease and Cost of Illness of Spinal Muscular Atrophy in Portugal. Value Health. 2022;25(1):S53.

7. Guojun T, Yan X, Weizhi W, Lihua W, Chunyang L, Xinghu Z, et al. A multicenter study to evaluate the disease burden and health economics of inpatients with multiple sclerosis in China. Mult Scler Relat Disord. 2022;60:103732.

8 DI Collaborators, GBD. Global burden of 369 diseases and injuries in 204 countries and territories, 1990-2019: a systematic analysis for the Global Burden of Disease Study 2019. Lancet. 2020;396(10258):1204-22.

9. Henrard S, Devleesschauwer B, Beutels P, Callens M, De Smet F, Hermans C, et al. The health and economic burden of haemophilia in Belgium: A rare, expensive and challenging disease. Orphanet J Rare Dis. 2014;9(1).

10. Inês M, Coelho T, Conceição I, Landeiro F, de Carvalho M, Costa J. Societal costs and burden of hereditary transthyretin amyloidosis polyneuropathy. Amyloid. 2020;27(2):89-96.

11. Janphram C, Worawichawong S, Boongird S, Udomsubpayakul U, Assanatham M, Kitiyakara C. Year of life lost due to premature death from glomerulonephritis in Thailand. J Am Soc Nephrol. 2021;32:491-2.

12. Kansal K, Mareddy M, Sloane K, Minc A, Rabins P, McGready J, et al. Meta-analysis of survival duration and years of life lost in frontotemporal dementia. 2015;27:S138-S9.

13. Liu X, Cui Y, Han J. Estimating epidemiological data of Multiple sclerosis using hospitalized data in Shandong Province, China Dr. Segolene Ayme. Orphanet J Rare Dis. 2016;11(1).

14. MND Collaborators, GBD. Global, regional, and national burden of motor neuron diseases 1990-2016: a systematic analysis for the Global Burden of Disease Study 2016. Lancet Neurol. 2018;17(12):1083-97.

15. Odnoletkova I, Kindle G, Quinti I, Grimbacher B, Knerr V, Gathmann B, et al. The burden of common variable immunodeficiency disorders: A retrospective analysis of the European Society for Immunodeficiency (ESID) registry data 11 Medical and Health Sciences 1117 Public Health and Health Services. Orphanet J Rare Dis. 2018;13(1).

16. Siddiqi AeA, Ebrahim SH, Soucie JM, Parker CS, Atrash HK. Burden of Disease Resulting from Hemophilia in the U.S. Am J Prev Med. 2010;38(4 SUPPL.):S482-S8.

17. Villaquiran-Torres C, Dueñas-Villamil R, Taborda-Restrepo A, Rojas-López K, Chamorro-Velásquez C, Londoño-Trujillo D. Burden of pulmonary arterial hypertension and chronic thromboembolic pulmonary hypertension in a developing country (colombia-south america). Am J Respir Crit Care Med. 2021;203(9).

18. Villaverde-Hueso A, Sánchez-Valle E, Álvarez E, Morant C, Carreira PE, Martín-Arribas MC, et al. Estimating the burden of scleroderma disease in Spain. J Rheumatol. 2007;34(11):2236-42.

### Additional sources

### Rare diseases list sources: search strategy

| ***Website*** | ***URL_Link*** |
| --- | --- |
| Orphanet | <https://www.orpha.net/consor/cgi-bin/Disease_Search_List.php?lng=EN>) |
| Genetic and Rare Diseases information center (GARD) | <https://rarediseases.org/gard-rare-disease/?page=3> |

#### GBD study estimates: DALY per case calculation

| ***Disease*** | ***URL_Link*** |
| --- | --- |
| Down syndrome | <https://www.healthdata.org/results/gbd_summaries/2019/down-syndrome-level-4-cause> |
| Kilnefelter syndrome | <https://www.healthdata.org/results/gbd_summaries/2019/klinefelter-syndrome-level-4-cause> |
| Motor neuron diseases | <https://www.healthdata.org/results/gbd_summaries/2019/motor-neuron-disease-level-3-cause> |
| Multiple sclerosis | <https://www.healthdata.org/results/gbd_summaries/2019/multiple-sclerosis-level-3-cause> |
| Neural tube defects | <https://www.healthdata.org/results/gbd_summaries/2019/neural-tube-defects-level-4-cause> |
| Orofacial cleft | <https://www.healthdata.org/results/gbd_summaries/2019/orofacial-clefts-level-4-cause> |
| Turner syndrome | <https://www.healthdata.org/results/gbd_summaries/2019/turner-syndrome-level-4-cause> |
| Thalassemia | <https://www.healthdata.org/results/gbd_summaries/2019/thalassemias-level-4-cause> |
| Sickle cell disease | <https://www.healthdata.org/results/gbd_summaries/2019/sickle-cell-disorders-level-4-cause> |
